# *De Novo* Design of Glycan Foldamers with Programmable Tertiary Structure

**DOI:** 10.1021/jacs.5c20415

**Published:** 2026-01-29

**Authors:** Yadiel Vázquez-Mena, Nishu Yadav, Martin Rosenthal, Yu Ogawa, Martina Delbianco

**Affiliations:** † University of Grenoble Alps, CNRS, CERMAV, 38000 Grenoble, France; ‡ Department of Biomolecular Systems, Max Planck Institute of Colloids and Interfaces, Am Mühlenberg 1 14476 Potsdam, Germany; § Department of Chemistry and Biochemistry, Freie Universität Berlin, Arnimallee 22 14195 Berlin, Germany; ∥ Faculty of Chemistry, 26657 KU Leuven, Celestijnenlaan 200F, Box 2404 B-3001 Leuven, Belgium; ⊥ Department of Sustainable and Bioinspired Materials, Max Planck Institute of Colloids and Interfaces, Am Mühlenberg 1 14476 Potsdam, Germany

## Abstract

*De novo* molecular design has yielded proteins and peptides with structures and functions beyond those found in nature. Despite the potential for glycans to form a broader scope of well-defined tertiary architectures, owing to the numerous conjugation sites and stereocenters, no one has yet built glycans with targeted structures and functions from scratch. Here, we designed glycan sequences that fold into programmable 3D architectures. Starting from first-principles, we create a linear glycan that spontaneously adopts a rigid tertiary structure not reported for natural glycans. Considering stereochemical and spatial orientation, we identify a rigid trisaccharide turn unit that programs backbone directionality, driving folding into antiparallel geometry. The combination of this turn unit with multiple cellulose-like strands completes our design, stabilizing a tertiary sheet-like folding, as confirmed by nuclear magnetic resonance spectroscopy and small-angle X-ray scattering (SAXS). To quantitatively evaluate the conformational landscape of our glycans in aqueous solution, we built a semiautomated protocol that integrates SAXS data with molecular dynamics simulations, demonstrating further the effectiveness of our design principles. This is an important step to design and control conformation populations, not just single structures in the solid state or of unknown prevalence in the solution phase. Together, these results show that glycans can be programmed to adopt rigid tertiary structures on demand, opening new avenues for *de novo* glycan-based architectures in synthetic glycobiology, catalysis, and materials science.

## Graphical abstract

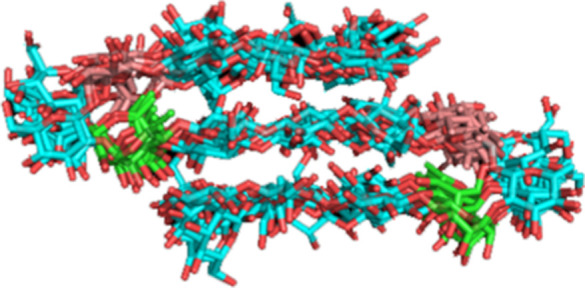

## Introduction

The ability to design molecules with programmable 3D structures has enabled the rational creation of systems with specific functions. By understanding and manipulating the principles that govern molecular folding and interactions, engineer peptides,^1^ proteins,^2–4^ and synthetic molecules^5^ were crafted with defined shapes and properties. While traditional approaches relied on modifying natural structures, the field of *de novo* protein design starts from first-principles,^6,7^ generating entirely new proteins. Unprecedented control over stability, folding, and activity led to the development of novel enzymes,^8,9^ self-assembling nanostructures,^10^ and therapeutic candidates^11^ that were previously unattainable using natural protein scaffolds.

The breakthrough in *de novo* protein design greatly benefitted from foundational studies on minimalistic secondary structures.^12^ By systematically exploring the principles governing α-helices,^13,14^ β-sheets,^15,16^ and turns,^17^ these early efforts provided crucial insights into the relationship between amino acid sequence, local structure, and overall stability. Minimalistic peptide model systems, particularly hairpins (Figure 1), have been instrumental in elucidating the fundamental principles governing peptide and protein folding.^12^ These constructs retain critical structural elements while minimizing complexity, enabling a precise examination of local conformational preferences, folding energies, and nucleation events. Building on these concepts, multiple-sheet systems were developed to probe how strand rigidity, hydrogen bonding, and side-chain interactions dictate conformational stability and organization.^18–20^ The resulting architectures begin to exhibit the spatial curvature and hydrophobic core typical of tertiary folds and are defined “minimal tertiary units”.^16,19,21,22^

**Figure 1:**
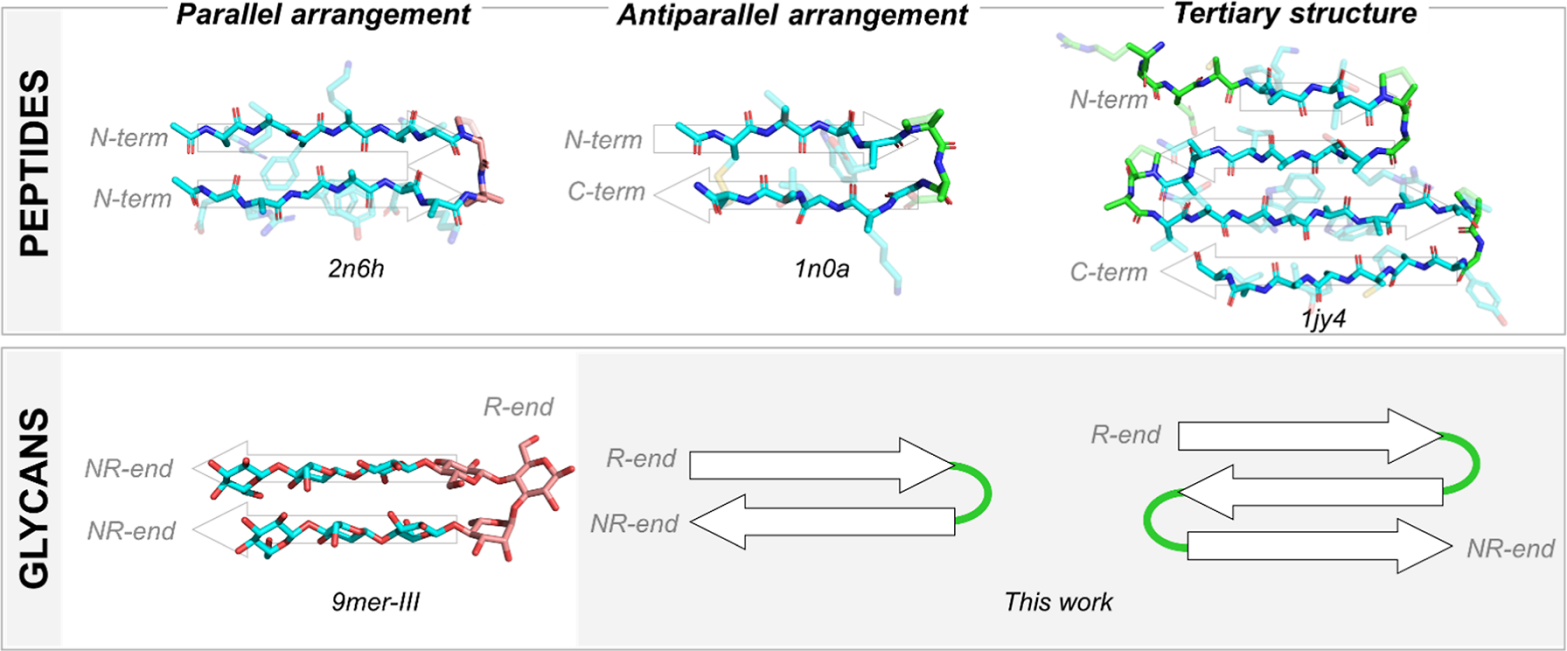
Peptide 3D arrangements and analogous glycan geometries: parallel, antiparallel, and tertiary sheet-like motifs. Our prior minimal branched oligosaccharide (**9mer-III**) formed a parallel hairpin, but parallel strand directionality prevents assembly into multistrand tertiary folds. Antiparallel register permits chains to fold back and form a tertiary structure. Realizing such tertiary architecture requires the *de novo* design of a synthetic turn unit (this work). *N*-term: peptide *N*-terminus; *C*-term: peptide *C*-terminus; NR-end: nonreducing end of the glycan; *R*-end: reducing end of the glycan. The compound nomenclature is based on the PDB IDs for the peptides (**2n6h** (2-stranded parallel β-sheet peptide); **1n0a** (2-stranded antiparallel β-sheet peptide); **1jy4** (4-stranded antiparallel β-sheet peptide)) and on Yadav et al. for the parallel glycan hairpin **9mer-III**.

Similar to peptides, glycans are capable of forming ordered architectures ranging from helices^23^ and sheets^24,25^ to complex multiscale assemblies, such as in plant cell walls.^26^ This structural versatility underlies many of their biological functions, like structural support,^26^ energy storage,^27^ and cell signaling,^28^ and offers an intriguing parallel to the design principles explored in proteins. Rational approaches to design glycans could unlock new possibilities in biomaterials and synthetic biology.^29^ However, the structural complexity of natural polysaccharides, combined with the time-consuming and challenging synthesis of well-defined oligomers, has significantly limited systematic structural studies in this field.

We have recently reported the minimalistic design of a branched oligosaccharide capable of folding into a parallel hairpin-like structure (Figure 1), a secondary structure not yet discovered in nature.^30,31^ However, this arrangement is not suitable to increase glycan complexity toward a tertiary motif due to the directionality of the growing glycan strands (i.e., pointing in the same direction). By contrast, an antiparallel geometry allows the polymer to fold back on itself, ultimately allowing for the creation of a tertiary motif that include multiple strands and turn units, such as those observed in peptides (Figure 1). Key to the antiparallel sheet-like conformation is the creation of an engineered, non-natural turn unit that programs backbone directionality. This geometry, to the best of our knowledge, is not available in nature, demanding careful consideration of both the stereochemistry and spatial orientation.

Here, we report the *de novo* design of a glycan turn for the construction of an antiparallel sheet-like geometry, culminating in a three-stranded system adopting a tertiary organization. Automated glycan assembly^32^ (AGA) allowed us to access a series of self-folding glycan sequences that were analyzed with nuclear magnetic resonance (NMR) spectroscopy, providing unambiguous evidence of their folded conformations. Moreover, we present a protocol based on small-angle X-ray scattering (SAXS) aided by molecular dynamics (MD) simulations for comprehensive conformational analysis, enabling quantitative assessment of folded populations in aqueous solution. Together, these methods provide unambiguous evidence of defined folded states in aqueous solution, establishing a framework for the *de novo* design of tertiary glycan architectures.

## Results and Discussion

### *De Novo* Design of an Antiparallel Glycan Turn Unit

Central to the hairpin and sheet architectures is the turn unit, which facilitates the connection of two strands (parallel hairpin) or reversal of chain direction (antiparallel hairpin) and guarantees strand alignment through intramolecular hydrogen bonding.^17^ Peptide models have shown that the key to designing turn motifs is the incorporation of a semirigid structure with a preformed conformation that enforces the necessary bend.^17^ Additionally, stabilizing interactions between the ends of the turn, such as hydrogen bonds, are essential for maintaining the desired geometry.^15,17^

Similar features are also stabilizing the natural Lewis *x* (Le^*x*^) trisaccharide and the biomimetic turn **3mer-III**, employed for the construction of a parallel glycan hairpin (Figure 2a).^30,31^ Le^*x*^ adopts a semirigid closed conformation stabilized by hydrophobic interactions between the methyl group of l-Fuc and the β-face of d-Gal^33^ (Figure 2a). A nonconventional CH···O hydrogen bond has been proposed between the H-5 of l-Fuc and O-5 atom of d-Gal, conferring additional stability to the folded structure.^34–36^ **3mer-III** is based on the same backbone, but with engineered orientation of certain hydroxyl groups,^30^ to allow for the introduction of cellulose strands, toward a parallel hairpin geometry (**9mer-III**). A similar geometry is not available in the glycan portfolio of linear trisaccharides, demanding the design of an antiparallel semirigid glycan framework from scratch.

**Figure 2:**
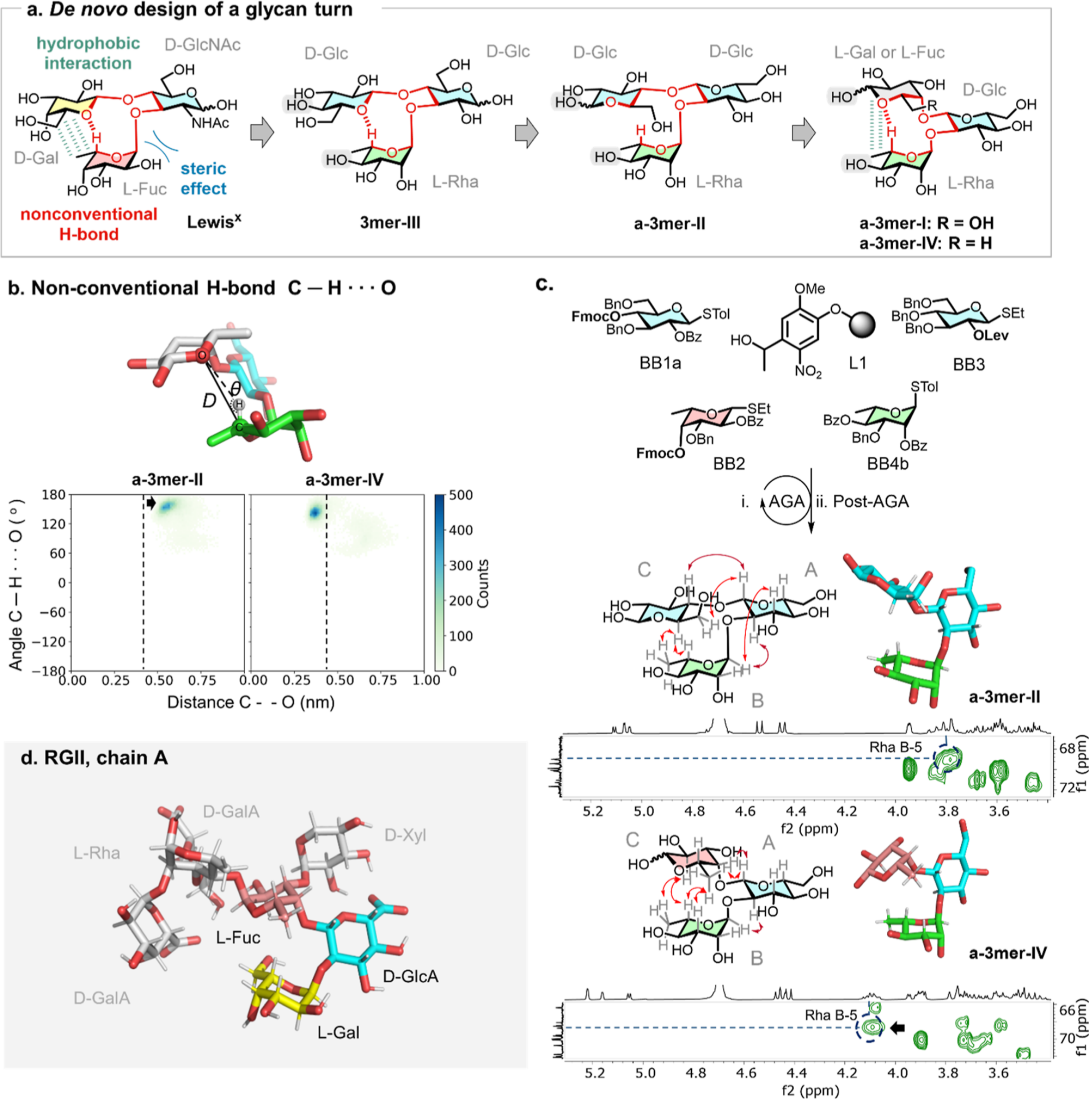
a) Design of an antiparallel trisaccharide turn unit. (b) C--O distance versus C–H···O angle plot extracted from MD simulations to evaluate the possibility of a nonconventional hydrogen bond stabilizing the turn unit. The results support a favorable interaction for **a-3mer-IV** but not for **a-3mer-II**. (c) Synthesis of the glycan turn units via AGA using protected monosaccharide building blocks (BBs). The chemical shift of Rha-5 suggested the presence of a nonconventional CH···O hydrogen bond in **a-3mer-IV** but not in **a**-**3mer-II**. Experimental NOEs (red arrows) from NOESY NMR experiments confirmed the closed conformation of ****a**-3mer-IV** and a more open conformation for **a-3mer-II**. (d) Structure of natural glycan from rhamnogalacturonan II (RGII) chain A. The highlighted trisaccharide motif corresponds to the backbone reproduced in our *de novo* design.

For our *de novo* design, we placed an β-d-Glc residue as the core of the turn motif. The equatorial orientation of the hydroxyl groups at positions C-1 and C-2 enforced a bend, mimicking the orientation of the hydroxyl groups (at positions C-3 and C-4) in the branching d-Glc unit in **3mer-III** (Figure 2a). Position C-2 was glycosylated with an α-l-Rha. This choice was made to introduce a hydrophobic interaction between the methyl group of l-Rha and the reducing-end residue installed at C-1 of the d-Glc unit, analogous to the stabilizing interaction seen in the Le^*x*^ motif. Initially, we explored the possibility of a d-Glc residue at the reducing end, forming a β­(1,4) linkage with the central d-Glc core to complete the turn structure (**a-3mer-II**, where a- indicates the antiparallel orientation). This framework features 11 atoms between the H-5 of l-Rha and the O-5 of d-Glc. While this differs from the 10-membered ring system commonly found in natural motifs (e.g., β-turn, Le^*x*^), it was shown that 11-membered hydrogen-bonded rings can support antiparallel hairpin formation in peptides.^37^ A more critical issue was the orientation of the O-5 atom of the reducing end d-Glc in **a-3mer-II**, which pointed upward, preventing orbital overlap with H-5 of L-Rha. Thus, we replaced the reducing-end d-Glc residue with l-pyranoses that have an axial orientation of the C-4 hydroxyl group such as l-Gal (**a-3mer-I**) or l-Fuc (**a-3mer-IV**). In these trisaccharides, the O-5 of the reducing-end unit adopted a favorable orientation for a potential interaction with H-5 of l-Rha, thereby restoring the possibility of a stabilizing CH···O hydrogen bond.

Atomistic MD simulations were performed to evaluate the propensity of the designed glycans to adopt the turn conformation. Each structure was subjected to a 500 ns simulation using a refined variant of the GLYCAM06 ^38^ carbohydrate force field. To minimize nonspecific interactions between monomer units, the systems were solvated using the TIP5P^39^ water model. A comparison of the conformational predictions for **a-3mer-II** and **a-3mer-IV** revealed substantial differences in their overall conformations, as evidenced by the radius of gyration plots (Supplementary 5.2). Negligible differences were detected between **a-3mer-IV** (containing l-Fuc) and **a-3mer-I** (containing l-Gal) (Supplementary 5.2). This observation is in line with the hypothesis that, in many plant glycan motifs, replacement of l-Fuc by l-Gal does not significantly alter the overall three-dimensional structure of the glycans.^40,41^ Therefore, from now on, we will focus our discussion on **a-3mer-IV**, a more accessible target, lacking the unnatural l-Gal unit. The full characterization of **a-3mer-I** can be found in Supplements 5.2 and 5.3.

To verify the possibility of hydrogen bonding between the reducing and nonreducing monosaccharides, a 2D plot donor–acceptor distance versus angle was generated (Figure 2b). This analysis focused on nonconventional hydrogen bonds, with optimal interactions defined by C--O distances shorter than 0.4 nm and C–H···O angles greater than 150°.^42–44^ The result confirmed that the dominant population of **a-3mer-IV** falls within this favorable range, whereas that of **a-3mer-II** is entirely out of it. Therefore, the MD results supported our hypothesis that an unconventional hydrogen bond stabilizes **a-3mer-IV**, but not **a-3mer-II**.

These two turn units were synthesized using AGA from protected monosaccharide building blocks (Figure 2c; for details see Supplementary 4.5.1 and 4.5.2). Cycles of the glycosylation and deprotection afforded the protected trisaccharides. Post-AGA steps included solid-phase methanolysis, photocleavage from the solid support, and hydrogenolysis, revealing the target structures. The chemical shift deviation of Rha-5 was analyzed as an indicator of the hypothetic nonconventional hydrogen bond^34,35,45^ holding the two ends in the antiparallel orientation. Indeed, NMR analysis showed a substantial downfield shift for Rha-5 of **a-3mer-IV** (Δδ = 0.31 ppm) compared to **a-3mer-II**, in which no hydrogen bond can occur (Figure 2c).

NOESY experiments were performed to evaluate the differences in the overall shapes of the two turn units (supplementary 6.2 and 6.3). For both trisaccharides, the spatial proximity between protons was confirmed by a set of NOE cross-peaks (Figure 2c). For **a-3mer-II**, typical NOEs were detected between protons across the glycosidic linkage (Glc A-1/Glc C-4 and Rha B-1/Glc A-2), along with inter-residue cross-peaks involving a proton adjacent to Glc C-4 (Rha B-5/Glc C-5 and Rha B-6/Glc C-5). These results support the semiopen conformation predicted by MD, where only protons on one side of the Glc C ring are in proximity to the Rha B residue. In contrast, **a-3mer-IV** exhibited multiple key NOEs between Fuc C and Rha B (Rha B-5/Fuc C-6, Rha B-3/Fuc C-6, Rha B-5/Fuc C-2, and Rha B-6/Fuc C-2), besides the typical NOEs between protons across glycosidic bonds (Glc A-1/Fuc C-4 and Rha B-1/Glc A-2), indicating a more compact conformation.

A literature survey revealed no reports of the natural occurrence of this exact sequence. However, we identified a trisaccharide fragment α-l-Galp-(1→2)-β-d-GlcpA-(1→4)-l-FucpOH in the chain A of rhamnogalacturonan II sharing the same backbone connectivity (see Figure 2d and Supplementary 5.4).^40^ Notably, NMR studies indicated that the residues at the ends of this natural trisaccharide are in close spatial proximity, in agreements with our findings.^46^ Our conformational characterization could provide valuable insights into how such secondary structural elements influence the biological functions of complex glycans.

### Building a Glycan Antiparallel Hairpin with the Designed Turn Unit

The rigid and compact conformation of our *de novo*-designed trisaccharide motivated us to pursue the construction of a more complex secondary structure, such as an antiparallel hairpin (Figure 3a), the first step toward a tertiary sheet-like geometry. We previously demonstrated that the stacking of cellulose strands stabilized folding of a parallel hairpin.^30,31^ We reasoned that an antiparallel arrangement of cellulose strands could have a similar effect, mimicking the packing of the cellulose II allomorph.^47^ Cellulose II is the thermodynamically stable allomorph of cellulose and crystallizes in a monoclinic, two-chain unit cell arranged in an antiparallel fashion.^47^ Hydrophobic face-to-face packing results in an antiparallel sheet arrangement, offering a suitable system to achieve our target geometries (Figure 3a).

**Figure 3:**
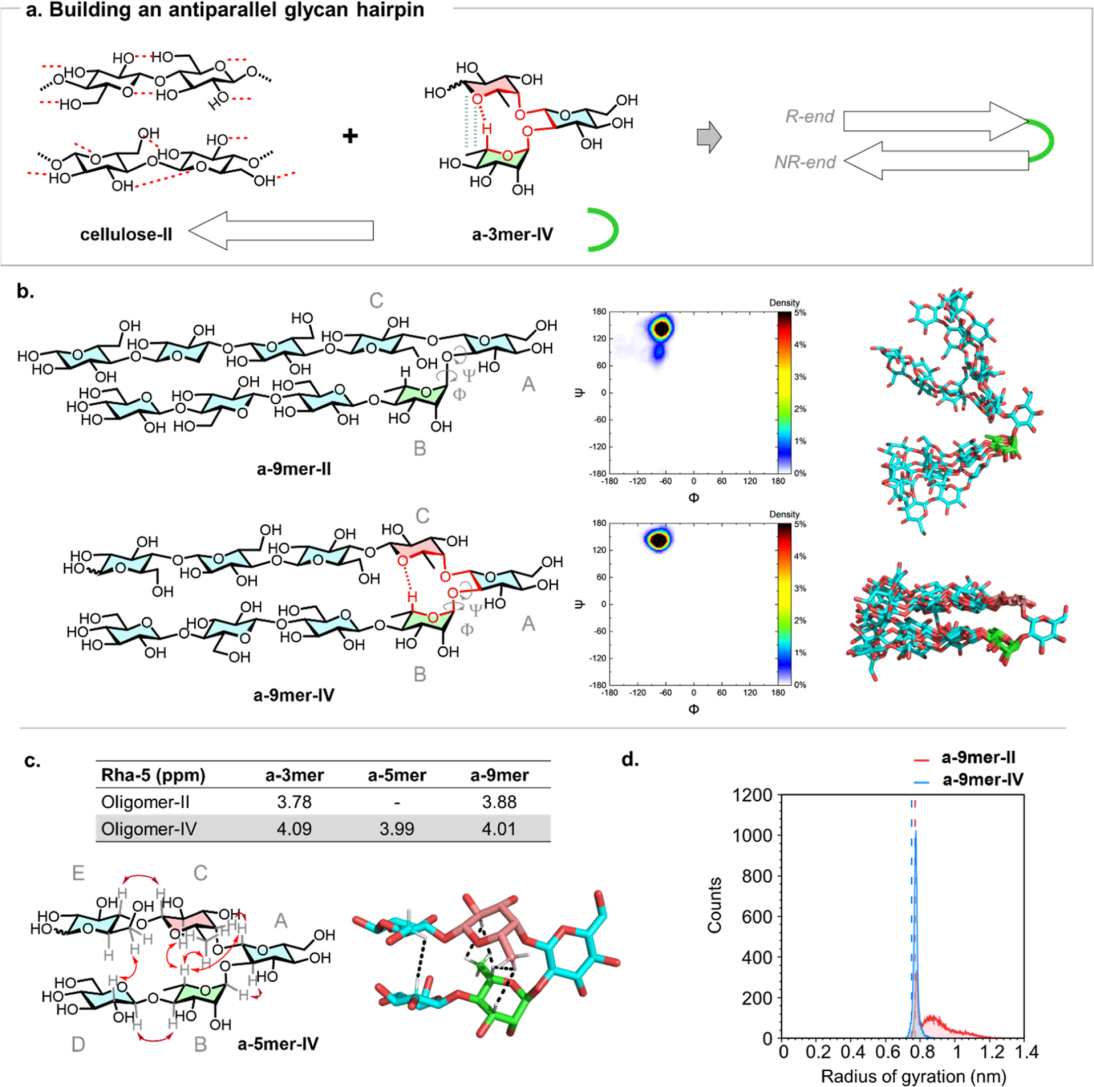
a) Schematic representation of the design strategy to obtain an antiparallel glycan hairpin utilizing the semirigid trisaccharide **a-3mer-IV**. (b) Chemical structure and representative conformations of **a-9mer-II** and **a-9mer-IV**. Ramachandran plots for the glycosidic bond between Rha B and Glc A in both hairpins. The MD results highlighted clear conformational differences between the two hairpins, showing that **a-9mer-IV** preferentially adopted a rigid hairpin conformation while **a-9mer-II** explored more conformational states. (c) Chemical shift of Rha-5 as an indicator of the presence of the nonconventional hydrogen bonding in oligomers based on **a-3mer-IV**. Experimental NOEs detected for **a-5mer-IV,** confirming its hairpin conformation. (d) Comparison of radius of gyration profiles obtained from MD simulations and experimental values measured by SAXS (dashed lines).

Both turn units (**a-3mer-II** and **a-3mer-IV**) were elongated with cellulose chains to give **a-9mer-II** and **a-9mer-IV**, respectively. MD simulations were performed to analyze the conformational preference of these two molecules prior to synthesis. In general, **a-9mer-IV** exhibited a more compact conformation and reduced flexibility compared to **a-9mer-II** (Supplementary 5.8–5.10). The analysis of the glycosidic linkage dihedral angles (Φ: O5′-C1′-O2-C2, Ψ: C1′-O2-C2-C1) within the turn region revealed that **a-9mer-IV** predominantly occupies a single conformational state (Figure 3b). In contrast, **a-9mer-II** displays two distinct conformational populations at the α-l-Rha-(1-2)-β-d-Glc glycosidic linkage. Inter-residue distance analysis supported the presence of a hairpin-like structure for **a-9mer-IV** for the majority of the simulation time; in contrast, **a-9mer-II** adopted predominantly an open conformation (Supplementary 5.8, 5.9, and 5.11). Taken together, MD results suggested a greater conformational rigidity for **a-9mer-IV** compared to the more flexible **a-9mer-II**, supporting the effectiveness of the designed turn in promoting conformational stability.

All target structures were prepared by AGA, following the same workflow described for the turn units (Supplementary 4.5). The collection included **a-5mer-IV**, a shorter sequence with minimal strands’ length of one Glc residue. According to MD simulations (supplementary 5.5 and 5.6), **a-5mer-IV** adopted the hairpin conformation and therefore offered a simplified model for comprehensive structural characterization using standard NMR techniques.

The chemical shift of Rha-5 was monitored as a qualitative reporter of the closed conformation of the turn units within the extended sequences (Figure 3c). All structures based on the **a-3mer-IV** turn (oligomer-IV series) were characterized by a downfield shift of Rha-5, suggesting the presence of the nonconventional hydrogen bond. A minor downfield shift was observed upon extension of the more flexible **a-3mer-II**, suggesting that cellulose strands can engage in some strand–strand interactions, promoting a closer conformation of the turn unit in **a-9mer-II** compared to that in **a-3mer-II**. Still, the chemical shift of Rha-5 remained far from the values of the more rigid oligomer-IV series.

We then proceeded with the analysis of proton proximities through detection of NOE couplings for **a-5mer-IV** (Supplementary 6.4). Several cross-peaks around the glycosidic linkages were identified in the 2D ROESY spectra. Due to the inherently weak NOE signals in a molecule of intermediate size and for protons separated by near 4 Å, which approach the sensitivity limit of the technique,^48^ we focused on key protons using 1D selective ROESY experiments (Supplementary 6.4 Figures S33 and S34). Selective irradiation of the Rha B-5 proton confirmed that the turn region adopted a conformation similar to that of the compact trisaccharide (Figure 3c). Additionally, irradiation of Glc D-2 provided evidence that the terminal residues were in close spatial proximity, further supporting that **a-5mer-IV** adopted a hairpin-like geometry.

The complete NMR signal assignment of the more complex structures was not feasible due to the substantial chemical shift degeneracy of the Glc residues. Nevertheless, guided by the analysis of **a-5mer-IV**, it was possible to assign signals corresponding to key residues of **a-9mer-IV** (Supplementary 6.6). 2D-NOESY spectra confirmed that the turn motif retained a compact conformation with key NOEs observed between Rha B-5 and Fuc C-6, as well as between Rha B-6 and Fuc C-2 (Supplementary 6.6 Figure S39).

To quickly analyze the overall shape of our molecules in solution, we turned our attention to SAXS, a robust scattering technique that provides essential structural parameters, such as the radius of gyration (Rg) of molecules. The Rg for dispersed glycans was determined using the Guinier approximation.^49^ We measured experimental Rg values of 7.7 Å for **a-9mer-II** and 7.5 Å for **a-9mer-IV**, in good agreement with those predicted by MD simulations for the two glycans (Figure 3d). Overall, our analysis supported the presence of a folded structure for **a-9mer-IV** and a less compact conformation for **a-9mer-II**.

### From a Hairpin to a Tertiary Sheet-like Architecture

To increase the complexity of glycan structures accessible via *de novo* design, we targeted a glycan sheet-like architecture (**ttt-15mer-IV**, Figure 4a, *n* = 3, where ttt denotes a three-strand glycan system composed of three trimers, i.e., a tritri-tri arrangement). This target required the insertion of an additional turn unit and a strand to our hairpin motif. In this arrangement, an odd number of d-Glc residues in the central strand is essential for achieving proper packing, as shown by representative snapshots from MD simulations of three systems with different strand lengths (Figure 4a). Thus, we fixed the inner cellulose-like core to three Glc units per strand.

**Figure 4:**
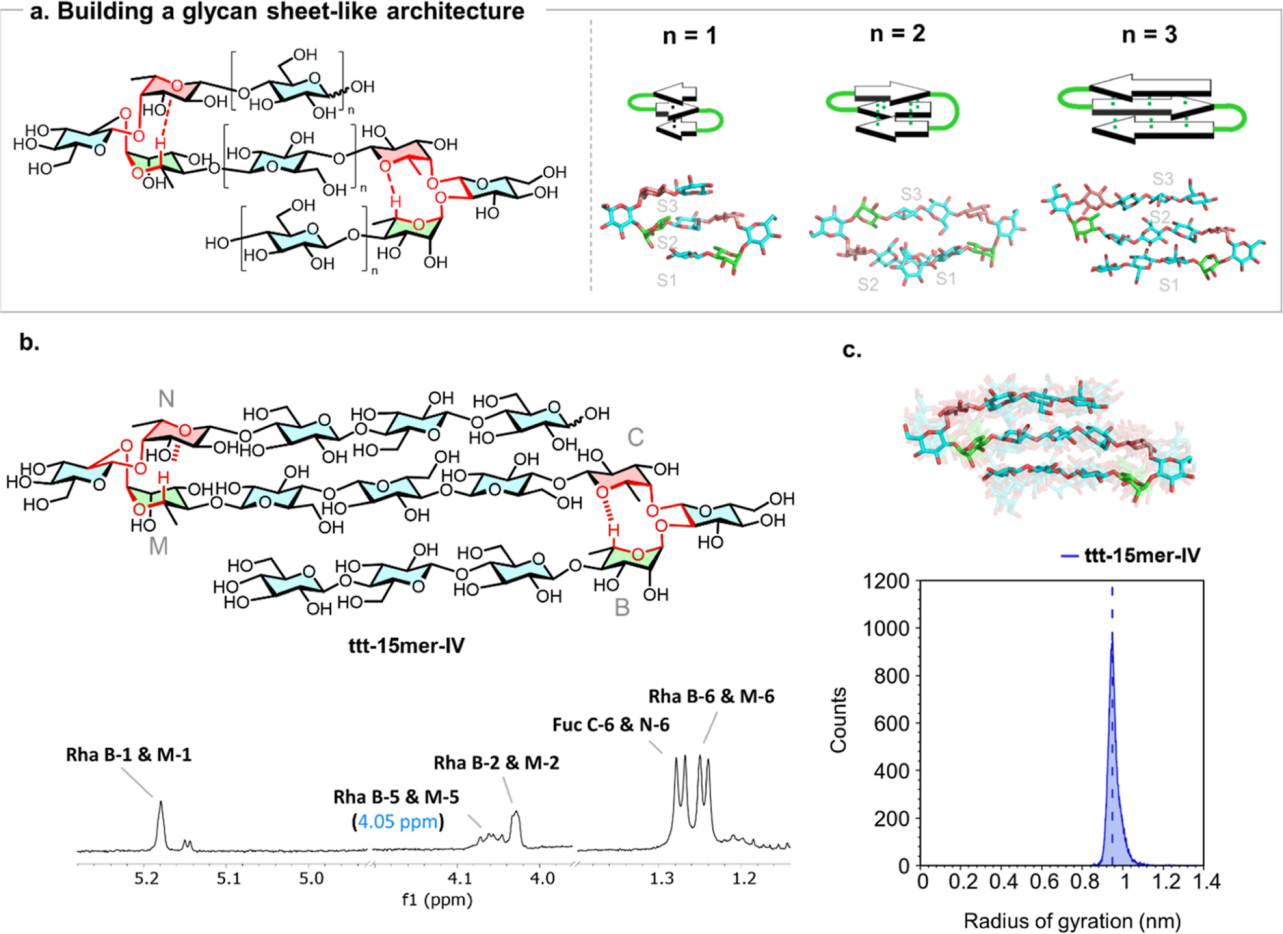
a) Strategy to obtain a glycan sheet-like architecture. The cooperativity along the perpendicular direction is expected to be optimal in systems with odd number of Glc residues in the central strand as shown by representative snapshots from MD simulations of three systems with different strand lengths. (b) The designed tertiary glycan system **ttt-15mer-IV** was analyzed by 1H NMR, showing that both turn units are chemically equivalent. The observed downfield shift of Rha-5—larger than in the related hairpin—indicates a certain degree of cooperativity. (c) Comparison of radius of gyration profile obtained from MD simulations and experimental value measured by SAXS (dashed line).

MD simulations supported that, in the designed **ttt-15mer-IV**, strand S1 aligned on one face of strand S2, while strand S3 aligned on the opposite face (Figure 4a). Monitoring of the inter-residue distances during the simulation time (Supplementary 5.12 and 5.13) confirmed tight packing and strand alignment, resulting in a sheet-like structure as predominant conformation.

The target **ttt-15mer-IV** structure was prepared by AGA (Supplementary 4.5) and analyzed by NMR spectroscopy and SAXS. NMR analysis of **ttt-15mer-IV** confirmed the equivalent chemical environment for both turn units, resulting in a single set of signals for homologous protons (Figure 4b). The downfield shift of Rha-5 was more prominent for **ttt-15mer-IV** compared to the two-stranded hairpin **a-9mer-IV** (δ = 4.05 vs 4.01 ppm, respectively), suggesting stabilization of folding with increased molecular complexity. SAXS analysis resulted in an experimental Rg value of 9.5 Å for **ttt-15mer-IV**, in good agreement with the MD simulation prediction (Figure 4c), confirming the formation of a tertiary motif.

### Quantification of Degree of Folding

The quantification of the “degree of folding” is a long-standing challenge in foldamer design, since classical analytical methods can only deliver information on the most populated states or averaged results.^50,51^ For example, traditional NMR observables mainly capture short-range distances and population-averaged data, making it difficult to resolve the conformational landscape that a molecule adopts.^52,53^ To address this limitation, we developed a semiautomated method based on SAXS combined with MD simulations for the semiquantitative characterization of the conformational space of glycans. Our protocol enabled the characterization of the structural preferences of glycans by (1) identifying accessible conformational states, (2) computing theoretical SAXS profiles for each simulated state, and (3) fitting weighted combinations of these profiles to experimental data to estimate conformational populations.

To group conformations from the MD trajectory for SAXS analysis, an interval-based approach was applied using 1 ns intervals along the trajectory. For each interval, the average Rg was calculated, reducing the effect of short-time scale fluctuations. This averaging strategy reflects the idea that conformational states, especially in flexible glycan, are better represented as dynamic ensembles rather than rigid structures. Based on the average Rg value, each trajectory interval was categorized into a representative conformational state: the folded conformers (fc, Figure 5a, blue) or the unfolded conformers (uf, Figure 5a, red). All trajectory intervals assigned to the same conformational state were concatenated, and SAXS intensity curves were generated for each conformational ensemble using the combined frames (Supplementary 7.2 and 7.3). The predicted SAXS data were then compared to the experimental results by plotting both the SAXS profile and the corresponding Kratky plot.

**Figure 5:**
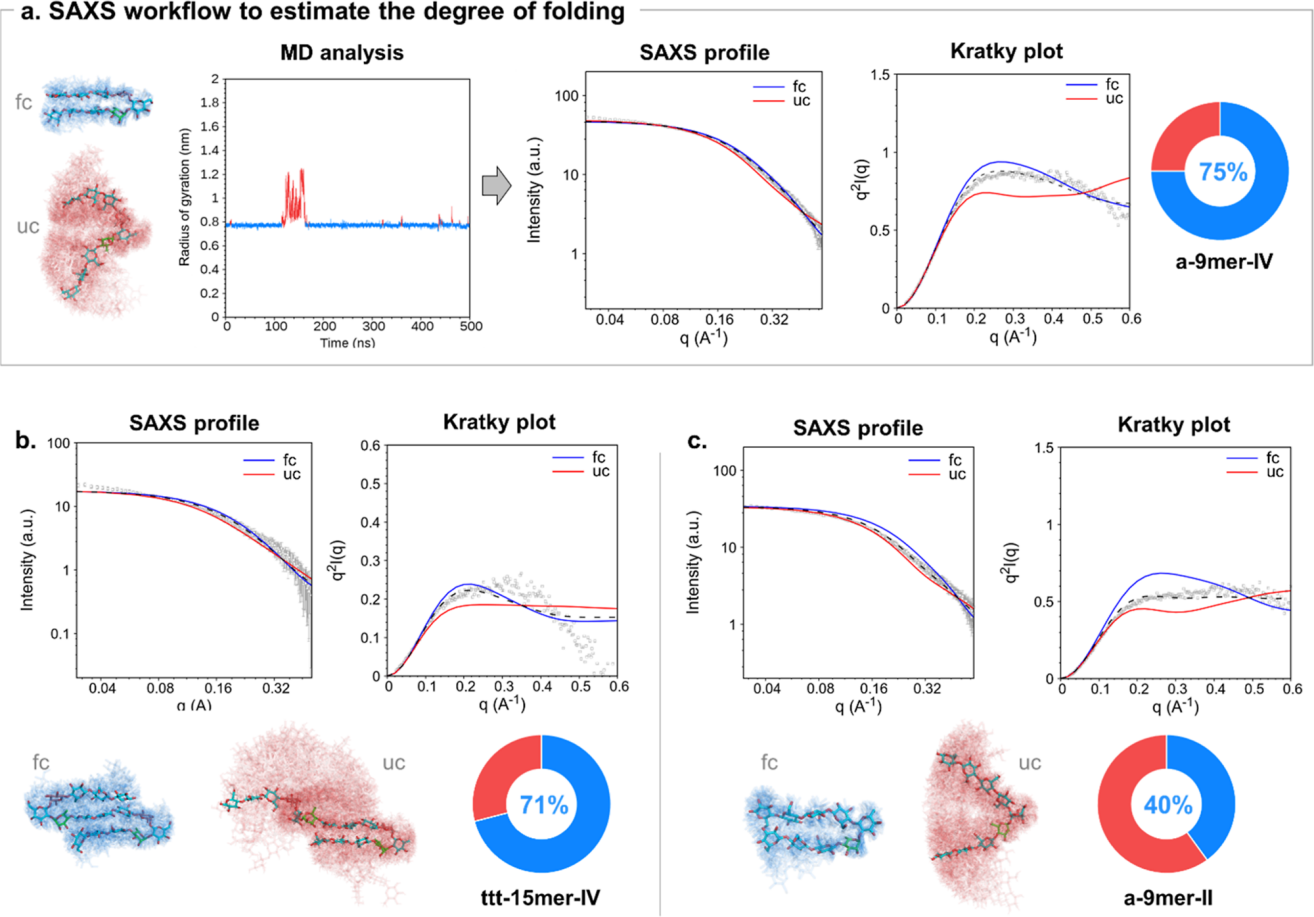
a) SAXS workflow to estimate the degree of folding exemplified for **a-9mer-IV**. Intervals of MD trajectories were categorized into the folded (fc, blue) and unfolded (uf, red) conformational states, as shown in the Rg plot and in the alignment of over 200 snapshots. The predicted scattering intensity for the two conformational states (blue and red solid lines) were compared with the experimental scattering intensity as shown in the SAXS profile and the Kratky plot (gray dots). Fitting the linear combination of the predicted curves (dashed line) to the experimental results allowed us to estimate the degree of folding (circle chart) for each molecule. The same workflow was followed to estimate the degree of folding of (b) **ttt-15mer-IV** and (c) **a-9mer-II**.

The computed SAXS intensity profiles were compared to the experimental data obtained for **a-9mer-IV** (Figure 5a), showing good agreement between the experiment and the predicted profile for the folded state (fc, blue). Fitting the linear combination of the predicted curves for the two conformational states (fc and uc) to the experimental results allowed us to estimate that the folded conformations represented approximately 75% of the overall conformational ensemble for **a-9mer-IV**. This result well matched the prediction of the folded population obtained by MD (87%, Supplementary 7.7).

The same protocol confirmed the predominance of the folded conformation for **ttt-15mer-IV**, resulting in approximately 71% folded conformations (Figure 5b). For this system, the analysis was based on the lower *q* range up to 0.4 Å-1 to focus on its overall molecular conformation, as this molecule is substantially larger than the other two hairpin molecules. The intensity decay observed at the higher *q* range above 0.5 Å-1 suggested the presence of an additional compact conformation state that was not accessible within the MD simulations.^54^

The folded conformations were estimated to contribute to 40% of the overall conformational ensemble of **a-9mer-II** (Figure 5c), supporting the greater conformational flexibility suggested by NMR spectroscopy and MD simulations. Interestingly, the percentage of folded conformations is higher than that predicted by MD (25%, Supplementary 7.6). Thus, SAXS data could be valuable to refine MD results and improve the glycan conformation prediction.

Overall, these results underscored the importance of the designed turn unit in stabilizing rigid secondary and tertiary structures from hairpin to sheet.

## Conclusion

In summary, we reported the *de novo* design of an antiparallel glycan hairpin and a sheet-like architecture, geometries not available in the natural pool of glycans. Central to this achievement was the development of a rigid, compact linear trisaccharide that functions as a turn unit. Taking advantage of multiple week interactions, this motif promoted folding through conformational preorganization and the formation of a nonconventional hydrogen bond, as suggested by detailed NMR studies. While no identical structure was found in the literature, a fragment of chain A from rhamnogalacturonan II in plants showed a similar backbone,^40^ raising the possibility that the folding behavior observed here may also be relevant in natural systems.

The introduction of glycan strands at both ends of the turn unit did not disrupt its rigid conformation but rather allowed the nucleation of the molecule into a stable antiparallel hairpin and a three-stranded glycan sheet, a minimal tertiary unit. The combination of NMR spectroscopy and SAXS analysis provided strong evidence for the compact folded conformations of these structures.

Overall, this work demonstrated that glycans can be rationally programmed to adopt secondary and tertiary structures on demand, suggesting new directions for the design of functional oligosaccharides. A new class of synthetic glycan foldamers could be imagined, opening exciting possibilities in glycomaterial design, glycobiology, and catalysis.^29,55^

The design of programmable glycan structures also pushed the development of analytical protocols that addressed a long-standing challenge in foldamer design^56,57^ —the quantification of folded populations. Our semiautomated workflow, based on SAXS combined with MD simulations, allowed for the quantification of conformational populations in aqueous solution. This protocol allowed us to quickly evaluate the success of glycan secondary structure design while providing important experimental data to refine MD results and improve glycan conformation prediction.

## Experimental Section

### Synthesis

The oligosaccharides were prepared using a home-built synthesizer designed at the Max Planck Institute of Colloids and Interface.^58^ All details concerning BB synthesis, AGA modules, and post-AGA manipulations can be found in Supporting Information, Sections 2–4.

### MD Simulations

For all simulations, a modified version GLYCAM06 force field was used.^38,59^ Initial conformations for single hairpin simulations were constructed with the Glycam Carbohydrate builder (https://glycam.org/). All compounds with a free reducing end were modeled as β anomers. The topology was subsequently converted using the Python script acpype. Simulations were performed in water as solvent using TIP5P^39^ as water model. The simulation time for the single-molecule experiments was 500 ns. Bonds involving hydrogens were constrained using the LINCS^60^ to allow a 2 fs time steps. Nonbonded interactions cutoff at 1.4 nm, long-range electrostatics were calculated using the particle mesh Ewald method.^61^ After energy minimization (steepest descent algorithm) and before the production run, the systems were equilibrated at 300 K for 50 ns in a canonical (NVT) ensemble (constant number of particles, volume, and temperature) and subsequently at 300 K and 1 bar for 50 ns in an isothermal–isobaric (NPT) ensemble. All MD simulations were performed using Gromacs 5.1.2.^62^ A Nosé–Hoover thermostat^63,64^ kept the temperature of 303 K constant, while a Parrinello–Rahman barostat^65,66^ ensured a constant pressure of 1 bar. The analysis was visualized using OriginPro 2023 and Gnuplot software. Further details on MD simulations are reported in Supporting Information Section 5.

### NMR Analysis

^1^H, ^13^C, HSQC, 1D and 2D TOCSY, 2D ROESY, and 2D NOESY NMR spectra were recorded on a Varian 400-MR (400 MHz), Varian 600-NMR (600 MHz), and Bruker Biospin AVANCE700 (700 MHz) spectrometer. Samples were prepared by dissolving lyophilized samples in D_2_O, using concentrations ≈1–6 mM. Proton resonances of the oligosaccharides were assigned using a combination of ^1^H, 2D COSY, HSQC, and 1D and 2D TOCSY. Selective 1D TOCSY (HOHAHA, pulse program: seldigpzs) spectra were recorded using different mixing times to assign all the resonances (d9 = 40, 80, 120, 160, and 200 ms). 2D TOCSY (pulse program: mlevphpp) spectra were recorded using a mixing time (d9 = 80 or 150 ms). 2D ROESY (pulse program: roesyph.2) and 2D NOESY (pulse program: noesygpphpp) spectra were recorded using different mixing times (p15 = 200 or 300 ms for ROESY and d8 = 600, 800, or 1000 ms for NOESY). The full NMR analysis of glycans reported in the manuscript can be found in Supporting Information Section 6.

### SAXS Analysis

X-ray scattering experiments were performed at the BM26 beamline of the European Synchrotron Radiation Facility. Samples of **a-9mer-II**, **a-9mer-IV**, and **ttt-15mer-IV** at concentrations 0.5 wt % at 25 °C were sealed in glass capillaries and mounted on a motorized sample changer. They were exposed to monochromatic X-rays of 12 eV (λ = 1.0332 Å). The scattering intensity was measured using a PILATUS 1M detector (DECTRIS, Switzerland). The data processing was performed using pyFAI software. Intensity and Rg analysis were done using Gnuplot software.

Explicit-solvent SAXS calculations were performed based on a modified version^67,68^ of GROMACS 2022.2 (GROMACS-SWAXS).^69^ Details of explicit-solvent SAXS calculations are presented in a previous publication.^70^ Here, in this modified GROMACS code, the solvent molecules in the solvation layer were considered in the SAXS intensity calculation, in contrast to the common approach, where only the solute molecules are used for the SAXS intensity simulations. This explicit solvent model can provide realistic simulated intensities, as the density fluctuation of water molecules around the solute certainly contributes to the overall SAXS intensities. A spatial envelope was built around the hairpin at a distance of 0.7 nm. The water subtraction was carried out using >1000 simulation frames of the pure-water simulation box. The atomic form factors were approximated by

$$f_{j}\left(q\right)=\sum_{k=1}^{4}c+a_{k}e^{-b_{k}{\left(\frac{q}{4\pi }\right)}^{2}}$$

where the values *a*_*k*_, *b*_*k*_, and *c* are the Cromer–Mann parameters.^71^ The orientational average was carried out using 500 *q*-vectors for each absolute value of *q*, and the solvent electron density was corrected to an experimental value of 334 e/nm^3^.

To fit the experimental SAXS data, we performed a linear combination of the predicted SAXS curves corresponding to different Rg-defined conformational states.

## Supplementary Material



## Data Availability

The authors declare that all data supporting the findings of this study are available within the article and in the Supporting Information files. Raw data for NMR analysis, SAXS analysis, and MD simulations can be downloaded from 10.17617/3.G7UTLR. Data are also available from the corresponding author upon request.

## Abbreviations

MD: molecular dynamics
NMR: nuclear magnetic resonance
SAXS: small-angle X-ray scattering
AGA: automated glycan assembly
Le^x^: Lewis X epitope
d-Glc: d-glucopyranose
l-Rha: l-rhamnopyranose
l-Gal: l-galactopyranose
l-Fuc: l-fucopyranose
NOE: nuclear Overhauser effect
NOESY: nuclear Overhauser effect spectroscopy
COSY: correlation spectroscopy
TOCSY: total correlation spectroscopy
HPLC: high-performance liquid chromatography
MALDI-TOF: matrix-assisted laser desorption ionization-time of flight
HR-MS: high-resolution mass spectrometry
BB: building block
Rg: radius of gyration
