# Supplementary material for: De Novo Design of Glycan Foldamers with Programmable Tertiary Structure
Source: J Am Chem Soc. 2026 Jan 29;148(5):5623–33. doi: 10.1021/jacs.5c20415 (PMC12903858; doi:10.1021/jacs.5c20415)
Supplement: Supplementary file 1 [file ja5c20415_si_001.pdf]

De Novo Design of Glycan Foldamers With Programmable Tertiary Structure

Yadiel Vázquez-Mena,[1,2] Nishu Yadav,[2,3] Martin Rosenthal,[4] Yu Ogawa[1,5*] and Martina Delbianco[2*]

[1] University of Grenoble Alps, CNRS, CERMAV, 38000 Grenoble, France

[2] Department of Biomolecular Systems, Max Planck Institute of Colloids and Interfaces, Am Mühlenberg 1, 14476 Potsdam, Germany

[3] Department of Chemistry and Biochemistry, Freie Universität Berlin, Arnimallee 22, 14195 Berlin, Germany

[4] Faculty of Chemistry, KU Leuven, Celestijnenlaan 200F, Box 2404, B-3001 Leuven, Belgium

[5] Department of Sustainable and Bioinspired Materials, Max Planck Institute of Colloids and Interfaces, Am Mühlenberg 1, 14476, Potsdam, Germany

1 Table of contents

2 General materials and methods 5
3 Building blocks synthesis 5
3.1 Synthesis of BB2 6
3.1.1 S-2 6
3.1.2 S-3 9
3.1.3 S-4 12
3.1.4 S-5 15
3.1.5 BB2 18
3.2 Synthesis of BB3 21
3.2.1 S-7 21
3.2.2 S-8 24
3.2.3 S-9 27
3.2.4 BB3 30
4 Automated glycan assembly 34
4.1 General materials and methods 34
4.2 Preparation of stock solutions 34
4.3 Modules for automated synthesis 34
4.3.1 Module A: Resin preparation 34
4.3.2 Module B: Acidic wash with TMSOTf solution (20 min) 34
4.3.3 Module C1: Thioglycoside glycosylation (35 min-55 min) 35
4.3.4 Module C2: Glycosyl phosphate glycosylation (45 min) 35
4.3.5 Module D: Capping (30 min) 36
4.3.6 Module E1: Fmoc deprotection (9 min) 37
4.3.7 Module E2: Lev deprotection (90 min) 37
4.4 Post-AGA manipulations 37
4.4.1 Module F: On-resin methanolysis 37
4.4.2 Module G1: Cleavage from solid support 37
4.4.3 Module G2: Micro-cleavage from solid support 38
4.4.4 Module H: Hydrogenolysis 38
4.4.5 Module I: Purification 38
4.5 Oligosaccharide synthesis 40
4.5.1 a-3mer-II 41
4.5.2 a-3mer-IV 45
4.5.3 a-5mer-IV 49

4.5.4 a-9mer-II 53
4.5.5 a-9mer-IV 57
4.5.6 ttt-15mer-IV 61
5 Molecular dynamics simulations 65
5.1 General materials and methods 65
5.2 RMSD, EtE distance and Rg of a-3mer-I, a-3mer-IV, and a-3mer-II 66
5.3 Inter-residue distance and Ramachandran plots of 3mers 67
5.4 MD analysis of natural 3mer 68
5.5 RMSD, EtE distance and Rg of a-5mer-I, a-5mer-IV, and a-5mer-II 69
5.6 Inter-residue distance plots of a-5mer-I, a-5mer-IV, and a-5mer-II 70
5.7 Ramachandran plots of a-5mer-I, a-5mer-IV, and a-5mer-II 71
5.8 RMSD, EtE distance and Rg of a-9mer-I, a-9mer-IV, and a-9mer-II 72
5.9 Inter-residue distance plots of a-9mer-I, a-9mer-IV, and a-9mer-II 73
5.10 Ramachandran plots of a-9mer-I, a-9mer-IV, and a-9mer-II 75
5.11 Bar graph of average inter-residue distance of a-3mer-I, a-3mer-IV and a-3mer-II and related hairpins 78
5.12 RMSD, EtE distance and Rg of ttt-15mer-I and ttt-15mer-IV 79
5.13 Inter-residue distance plots of ttt-15mer-I and ttt-15mer-IV 80
5.14 Ramachandran plots of ttt-15mer-I and ttt-15mer-IV 82
5.15 Inter-proton distances for a-3mer-II 87
5.16 Inter-proton distances for a-3mer-IV 88
5.17 Inter-proton distances for a-5mer-IV 90
5.18 Inter-proton distances for a-9mer-IV 93
6 NMR analysis 99
6.1 General materials and methods 99
6.2 NMR characterization of a-3mer-II 100
6.3 NMR characterization of a-3mer-IV 104
6.4 NMR characterization of a-5mer-IV 108
6.5 NMR characterization of a-9mer-II 114
6.6 NMR characterization of a-9mer-IV 116
7 SAXS analysis 119
7.1 General materials and methods 119
7.2 Conformational grouping and SAXS curve calculation 119
7.3 SAXS calculation 120
7.4 SAXS fitting 120

7.5 Experimental Rg calculation ... 122

7.6 Conformational analysis of a-9mer-II ... 123

7.7 Conformational analysis of a-9mer-IV ... 125

7.8 Conformational analysis of ttt-15mer-IV ... 127

8 References ... 129

2 General materials and methods

All chemicals used were reagent grade and used as supplied unless otherwise noted. The automated syntheses were performed on a home-built synthesizer developed at the Max Planck Institute of Colloids and Interfaces.[1] Analytical thin-layer chromatography (TLC) was performed on Merck silica gel 60 F254 plates (0.25 mm). Compounds were visualized by UV irradiation or dipping the plate in a staining solution (sugar stain: 10% H_2SO_4 in EtOH; CAM: 48 g/L ammonium molybdate, 60 g/L ceric ammonium molybdate in 6% H_2SO_4 aqueous solution). Flash column chromatography was carried out by using forced flow of the indicated solvent on Fluka Kieselgel 60 M (0.04 – 0.063 mm). Analysis and purification by normal and reverse phase HPLC were performed by using an Agilent 1200 series. Products were lyophilized using a Christ Alpha 2-4 LD plus freeze dryer. ^{1}H, ^{13}C and HSQC NMR spectra were recorded on a Varian 400-MR (400 MHz), Varian 600-NMR (600 MHz) spectrometer. Spectra were recorded in $CDCl_3$ by using the solvent residual peak chemical shift as the internal standard ($CDCl_3$: 7.26 ppm ^{1}H, 77.0 ppm ^{13}C) or in D_2O using the solvent as the internal standard in ^{1}H NMR (D_2O: 4.79 ppm ^{1}H). ^{1}H NMR spectra for all compounds were recorded without ^{13}C decoupling. Weak intensity ^{13}C resonances were derived from the respective HSQC crosspeaks. ^{1}H NMR integrals of the resonances corresponding to residues at the reducing end are reported as non-integer numbers and the sum of the integrals of α and β anomers is set to 1. High resolution mass spectra were obtained using a 6210 ESI-TOF mass spectrometer (Agilent) and a MALDI-TOF autoflexTM (Bruker). MALDI and ESI mass spectra were run on IonSpec Ultima instruments.

3 Building blocks synthesis

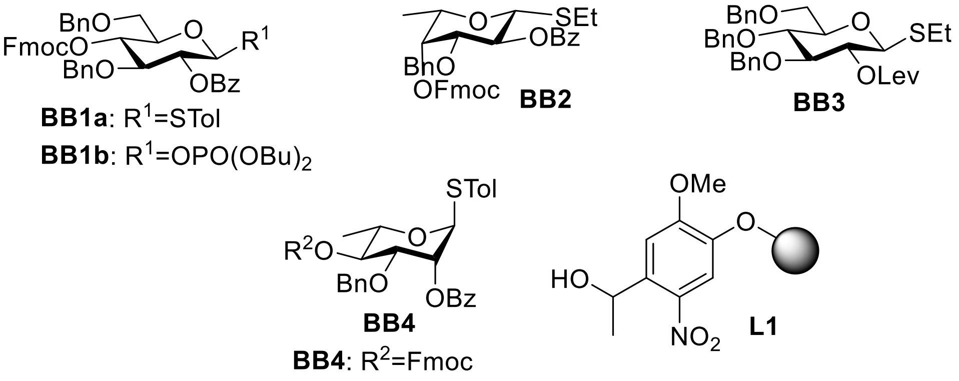

Figure 1 BBs and solid supports used in this work.

BB1a were purchased from GlycoUniverse (Germany). **BB1b**[2] and **BB4**[3] was synthesized according to previously reported procedures. The syntheses of **BB2** and **BB3** are described herein (**Scheme 1** and **2**). Merrifield resin equipped with photocleavable linkers **L1** (loading 0.35 mmol/g) was prepared according to previously reported procedures.[4]

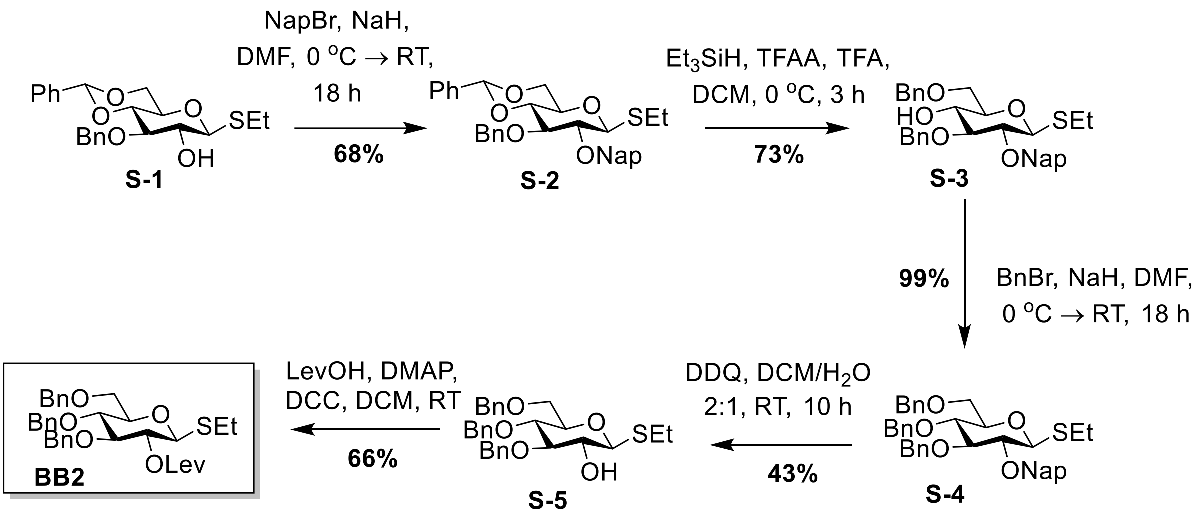

Scheme 1 Synthetic route for **BB2**.

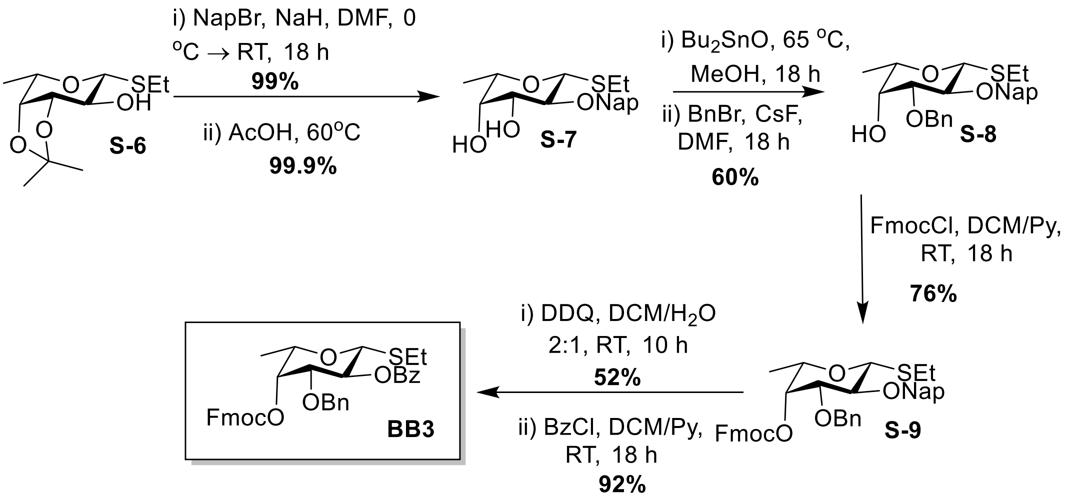

Scheme 2 Synthetic route for **BB3**.

3.1 Synthesis of BB2

3.1.1 S-2

Synthesis of ethyl 3-*O*-benzyl-4,6-*O*-benzylidene-2-*O*-(2-methyl)naphthyl-1-thio-β-D-glucopyranoside:

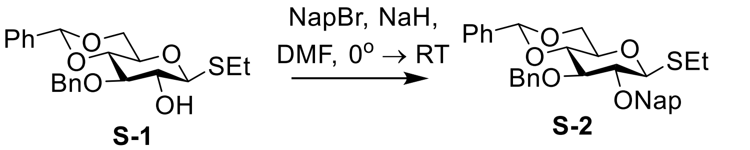

Ethyl 3-*O*-benzyl-4,6-*O*-benzylidene-1-thio-β-D-glucopyranoside S-1 was synthesized according to previously reported procedure.[5]

Ethyl 3-*O*-benzyl-4,6-*O*-benzylidene-1-thio-β-D-glucopyranoside (2.45 g, 6.1 mmol) and 2-(bromomethyl)-naphthalene (2.69 g, 12.2 mmol) were dissolved in DMF (6 mL) and cooled to 0 °C. Sodium hydride (292 mg, 12.2 mmol) was added and the mixture was stirred at RT

overnight. Then saturated NH_4Cl solution (300 mL) was added and the mixture was extracted with diethyl ether (2 x 30 mL). The combined organic layers were dried over Na_2SO_4. The crude product was purified with silica gel flash column chromatography (Hexane/Ethyl acetate, 3:1) to give compound **S-2** as white solid (2.25 g, 68 %).

^{1}H NMR (400 MHz, $CDCl_3$) δ 7.94 – 7.21 (m, 17H), 5.62 (s, 1H), 5.13 – 4.97 (m, 3H), 4.85 (d, *J* = 11.3 Hz, 1H), 4.63 (d, *J* = 9.8 Hz, 1H), 4.40 (dd, *J* = 10.4, 5.0 Hz, 1H), 3.95 – 3.72 (m, 3H), 3.63 – 3.44 (m, 2H), 2.92 – 2.69 (m, 2H), 1.36 (t, *J* = 7.4 Hz, 3H).

^{13}C NMR (101 MHz, $CDCl_3$) δ 138.44, 137.30, 135.48, 133.30, 133.11, 128.98, 128.39, 128.28, 128.08, 127.98, 127.73, 127.70, 127.03, 126.35, 126.01, 125.91, 101.15, 85.91, 82.85, 81.67, 81.33, 77.35, 77.03, 76.71, 76.04, 75.26, 70.27, 68.76, 25.23, 15.14, 1.04.

ESI-HRMS m/z 565.2025 $[M+Na]^+$ ($C_{33}H_{34}O_5SNa$ requires 565.2019).

^{1}H NMR of S-2 (400 MHz, $CDCl_3$)

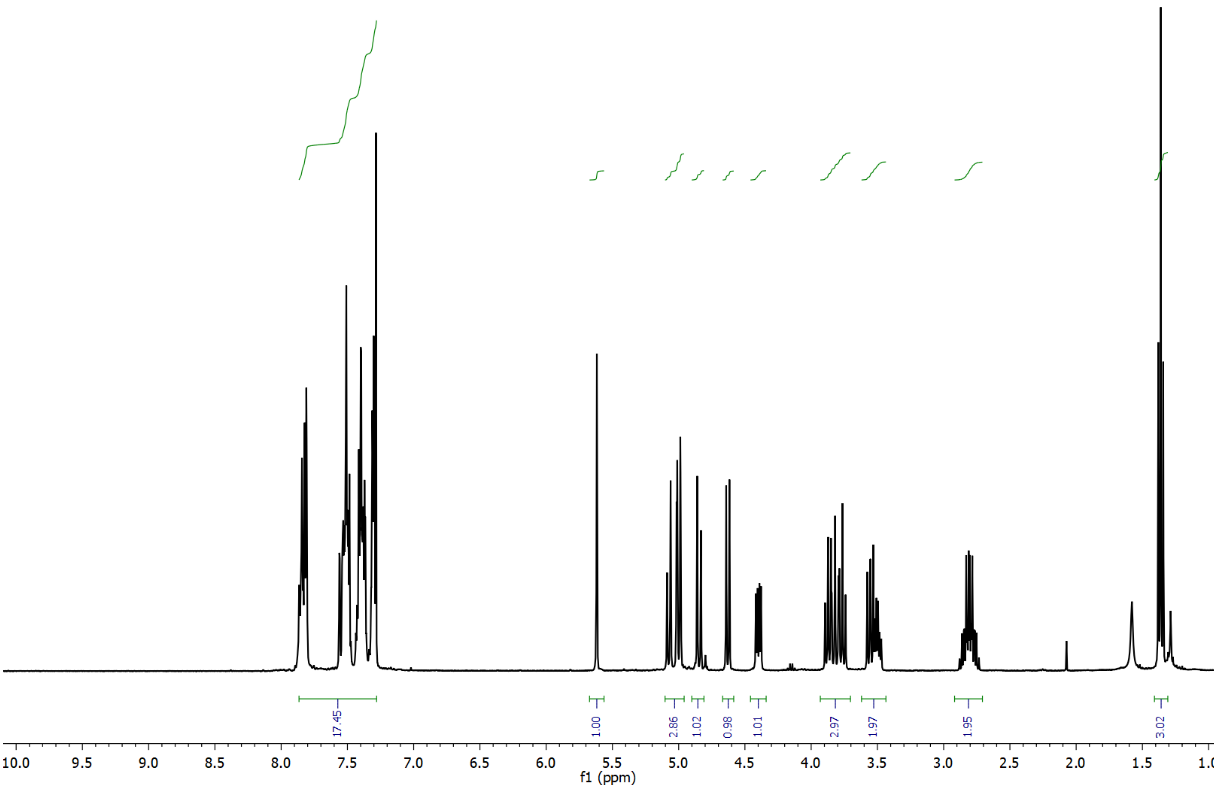

^{13}C NMR of S-2 (101 MHz, $CDCl_3$)

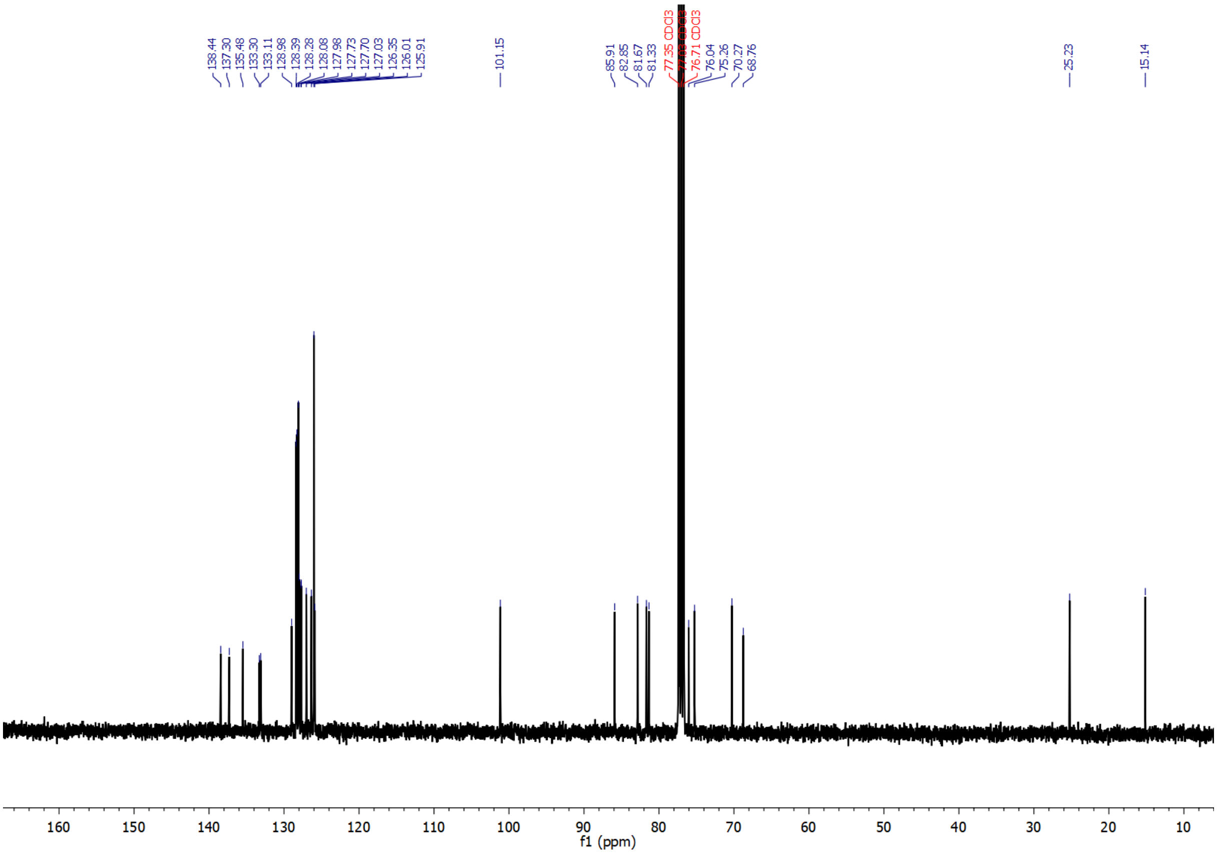

COSY NMR of S-2 ($CDCl_3$)

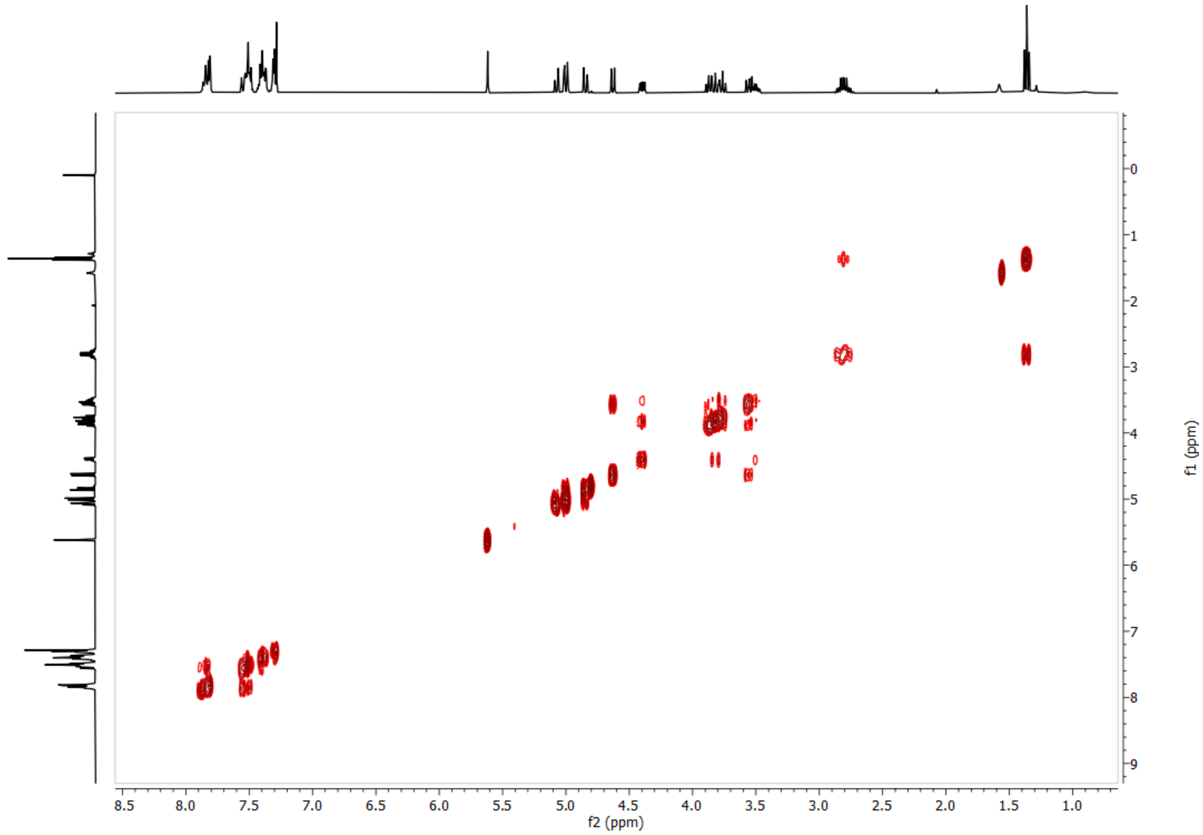

HSQC NMR of S-2 ($CDCl_3$)

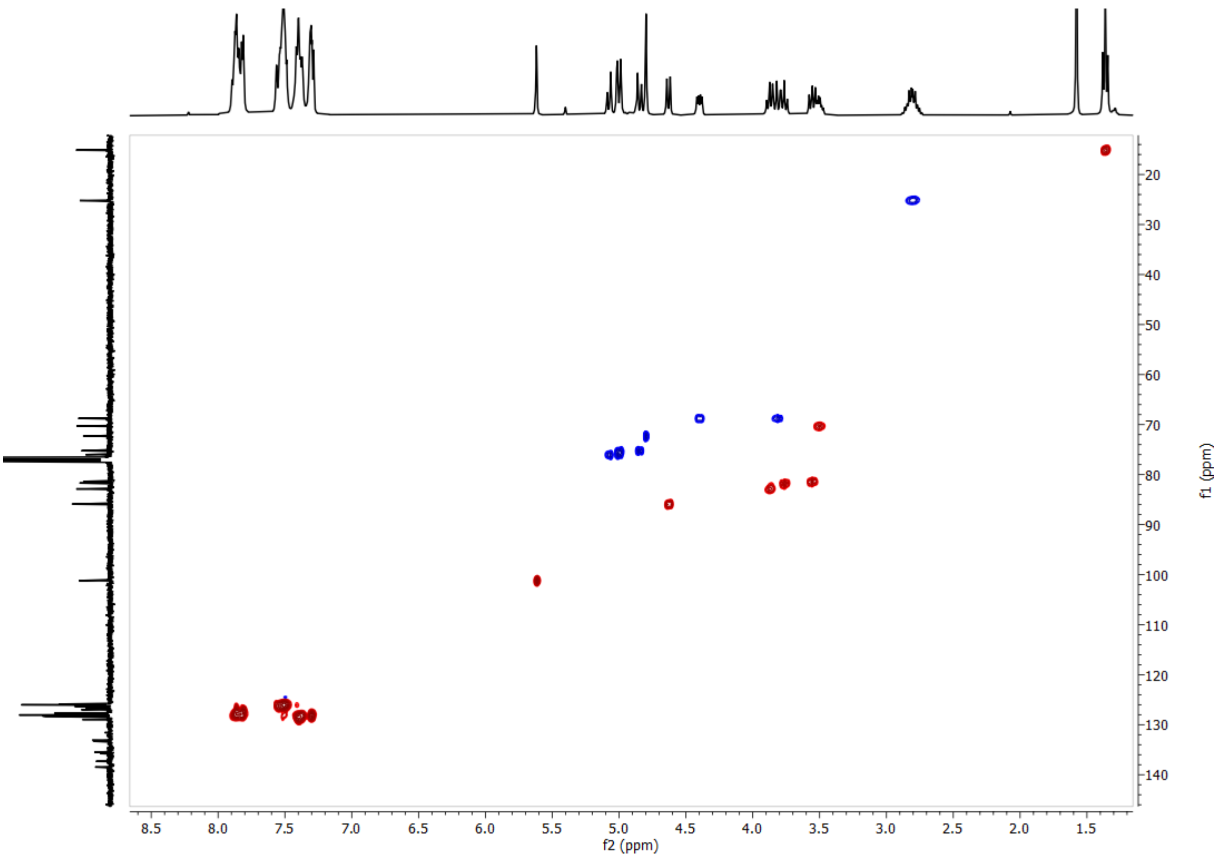

3.1.2 S-3

Synthesis of ethyl 3,6-di-O-benzyl-2-O-(2-methyl)naphthyl-1-thio-β-D-glucopyranoside

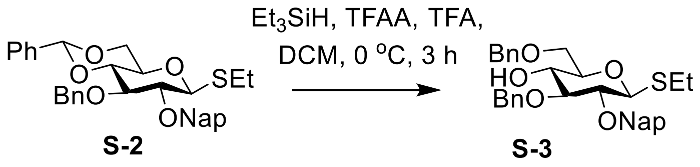

Ethyl 3-*O*-benzyl-4,6-*O*-benzylidene-2-*O*-(2-methyl)naphthyl-1-thio-β-D-glucopyranoside was dissolved in anhydrous DCM (11 mL). Triethylsilane (3.3 mL, 20.7 mmol) was added and the solution was cooled to 0 °C under Ar atmosphere. Trifluoroacetic acid (TFA) (1.6 mL, 20.7 mmol) and trifluoroacetic anhydride (TFAA) (0.3 mL, 2.07 mmol) were added sequentially. The solution was stirred at 0 °C for 3 h, after which time the reaction was diluted with DCM (5 mL) and washed once with a saturated aqueous solution of $NaHCO_3$ and once with brine. The organic layer was dried over $MgSO_4$, filtered, and concentrated under reduced pressure. The crude product was purified by silica gel flash column chromatography (Hexane/Ethyl acetate, 3:1) to give **S-3** as a white solid (1.65 g, 73 %).

^{1}H NMR (400 MHz, $CDCl_3$) δ 7.89 – 7.29 (m, 17H), 5.11 (d, J = 10.5 Hz, 1H), 5.01 – 4.82 (m, 3H), 4.66 – 4.51 (m, 3H), 3.83 – 3.65 (m, 3H), 3.63 – 3.44 (m, 3H), 2.80 (dd, J = 12.0, 7.4 Hz, 2H), 1.37 (d, J = 7.4 Hz, 3H).

^{13}C NMR (101 MHz, $CDCl_3$) δ 138.59, 137.84, 135.47, 133.31, 133.10, 128.59, 128.46, 128.13, 127.99, 127.91, 127.87, 127.80, 127.75, 127.69, 127.06, 126.36, 126.02, 125.92, 86.06, 85.20, 81.32, 77.92, 77.35, 77.03, 76.72, 75.53, 75.46, 73.71, 72.21, 70.67, 25.11, 15.19, 1.04.

ESI-HRMS m/z 567.2186 $[M+Na]^+$ ($C_{33}H_{36}O_5SNa$ requires 567.2175).

^1H NMR of S-3 (400 MHz, $CDCl_3$)

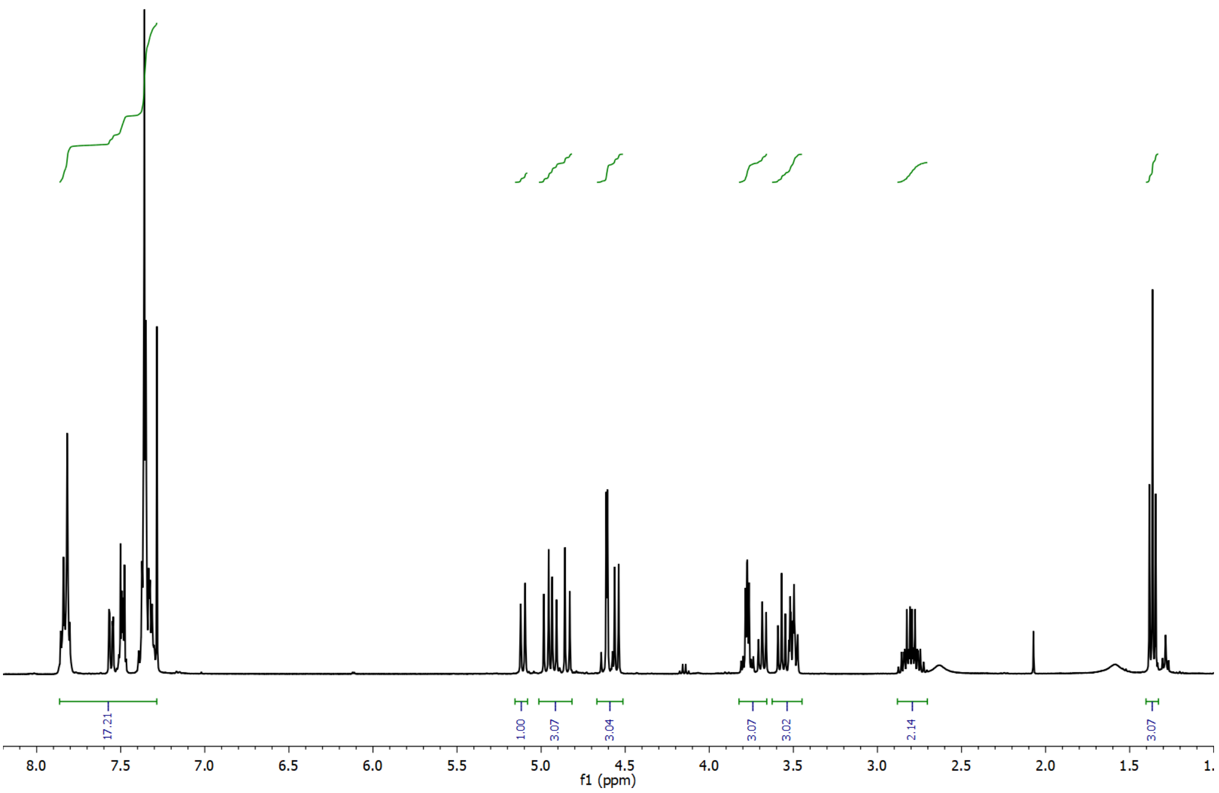

^{13}C NMR of S-3 (101 MHz, $CDCl_3$)

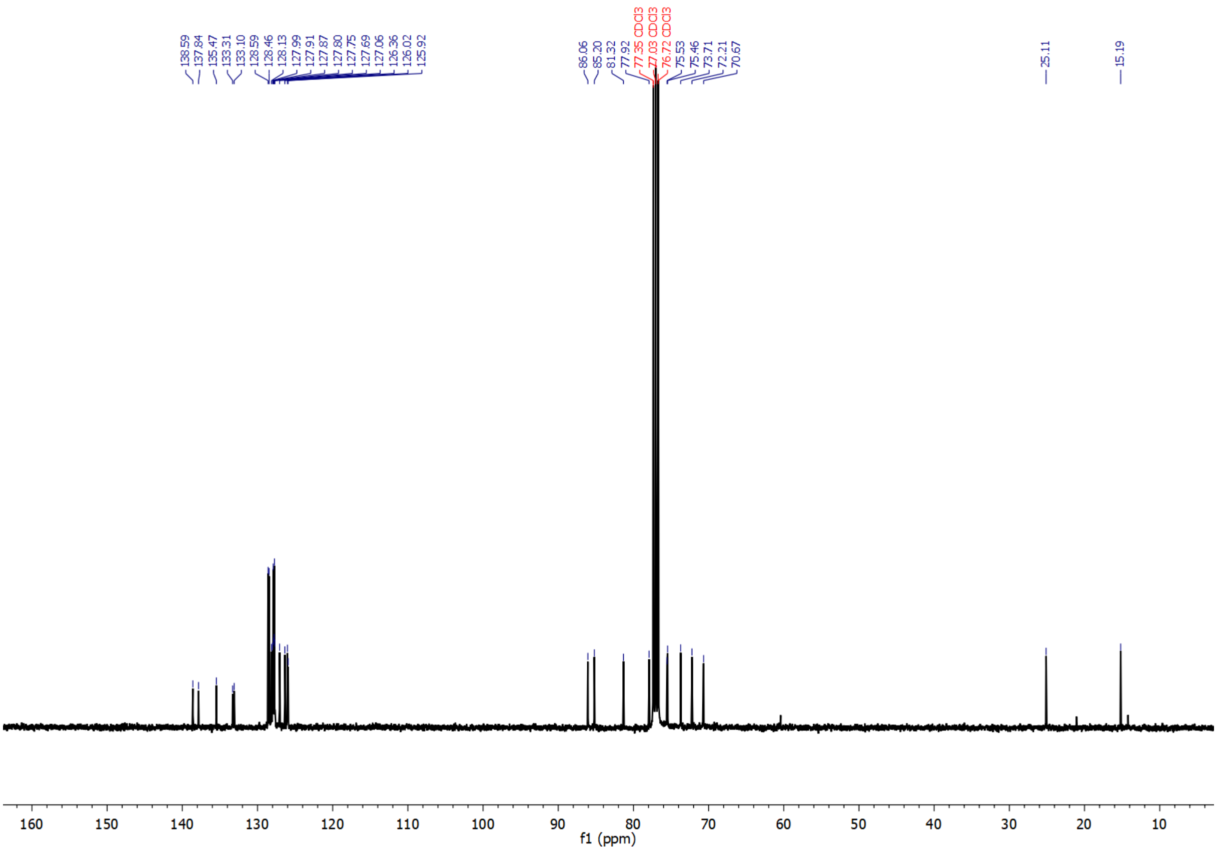

COSY NMR of S-3 ($CDCl_3$)

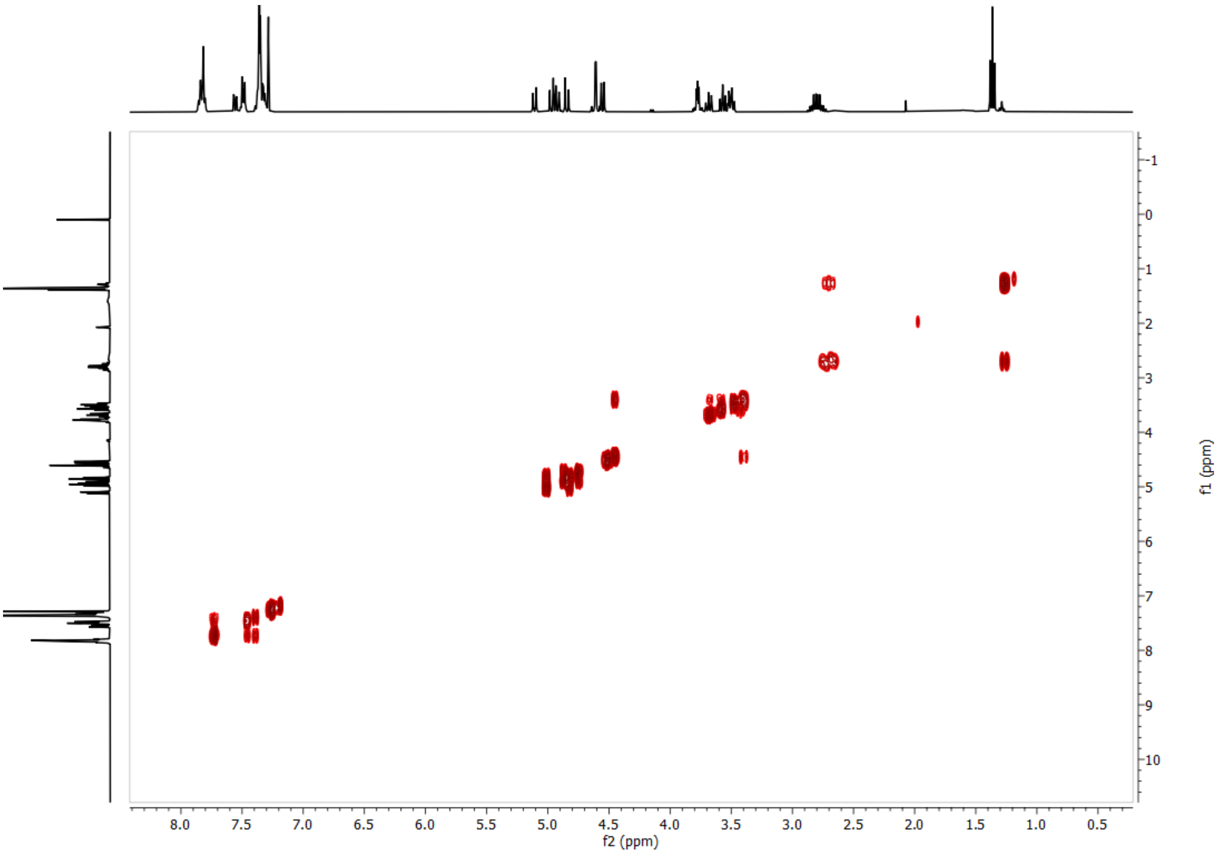

HSQC NMR of S-3 ($CDCl_3$)

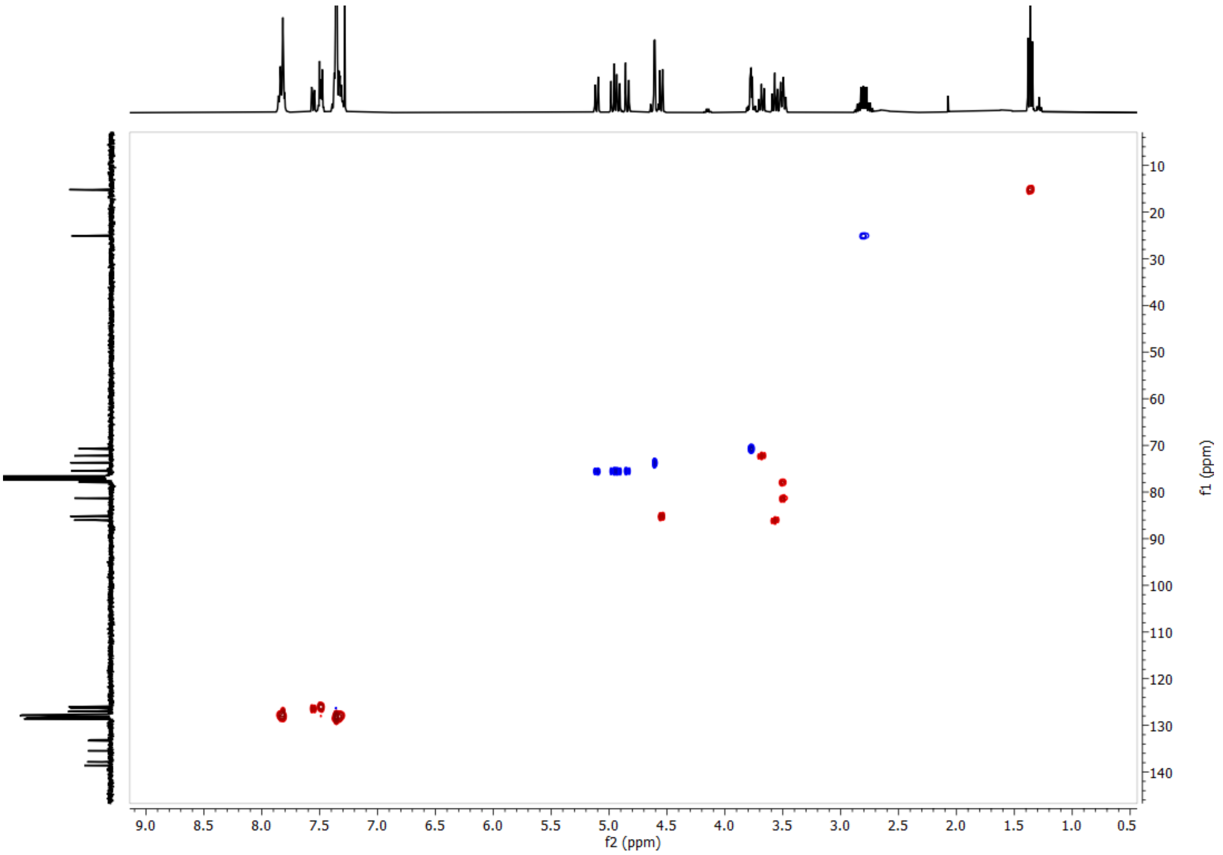

3.1.3 S-4

Synthesis of ethyl 3,4,6-tri-*O*-benzyl-2-*O*-(2-methyl)naphthyl-1-thio-β-D-glucopyranoside

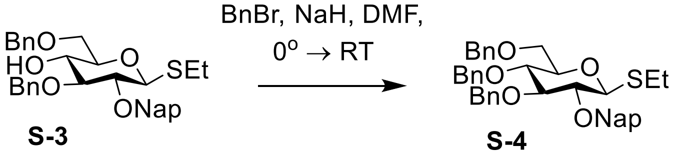

To a solution of ethyl 3,6-di-*O*-benzyl-2-*O*-(2-methyl)naphthyl-1-thio-β-D-glucopyranoside (1.65 g, 3.03 mmol, 1.0 eq) in anhydrous DMF (15 mL, 0.2 M), BnBr (1.1 mL, 9.1 mmol, 3.0 eq.) and sodium hydride (175 mg, 7.3 mmol, 2.4 eq) were added at 0°C. The reaction mixture was stirred at RT for 1 h and quenched with saturated aqueous NH_4Cl, extracted with Et_2O, and dried over $MgSO_4$. The crude was purified by silica gel column chromatography (Hexane/Ethyl acetate, 6:1) to give **S-4** as a white solid (1.92 g, 99 %).

^{1}H NMR (400 MHz, $CDCl_3$) δ 7.81 (dd, *J* = 10.8, 2.4 Hz, 3H), 7.51 – 7.19 (m, 19H), 5.11 (d, *J* = 10.4 Hz, 1H), 5.00 – 4.81 (m, 4H), 4.67 – 4.50 (m, 4H), 3.84 – 3.48 (m, 6H), 2.82 (dd, *J* = 12.5, 7.4 Hz, 2H), 1.37 (t, *J* = 7.4 Hz, 3H).

^{13}C NMR (101 MHz, $CDCl_3$) δ 138.55, 138.24, 138.06, 135.51, 133.30, 133.09, 128.45, 128.43, 128.36, 128.13, 128.00, 127.81, 127.75, 127.72, 127.68, 127.65, 127.60, 127.04, 126.38, 125.99, 125.89, 86.72, 85.10, 81.83, 79.16, 78.03, 77.35, 77.03, 76.71, 75.77, 75.59, 75.07, 73.47, 69.15, 25.06, 15.21, 1.04.

ESI-HRMS m/z 657.2669 [M+Na]$^+$ ($C_{40}H_{42}O_5SNa$ requires 657.2645).

^{1}H NMR of S4 (400 MHz, CDCl$_3$)

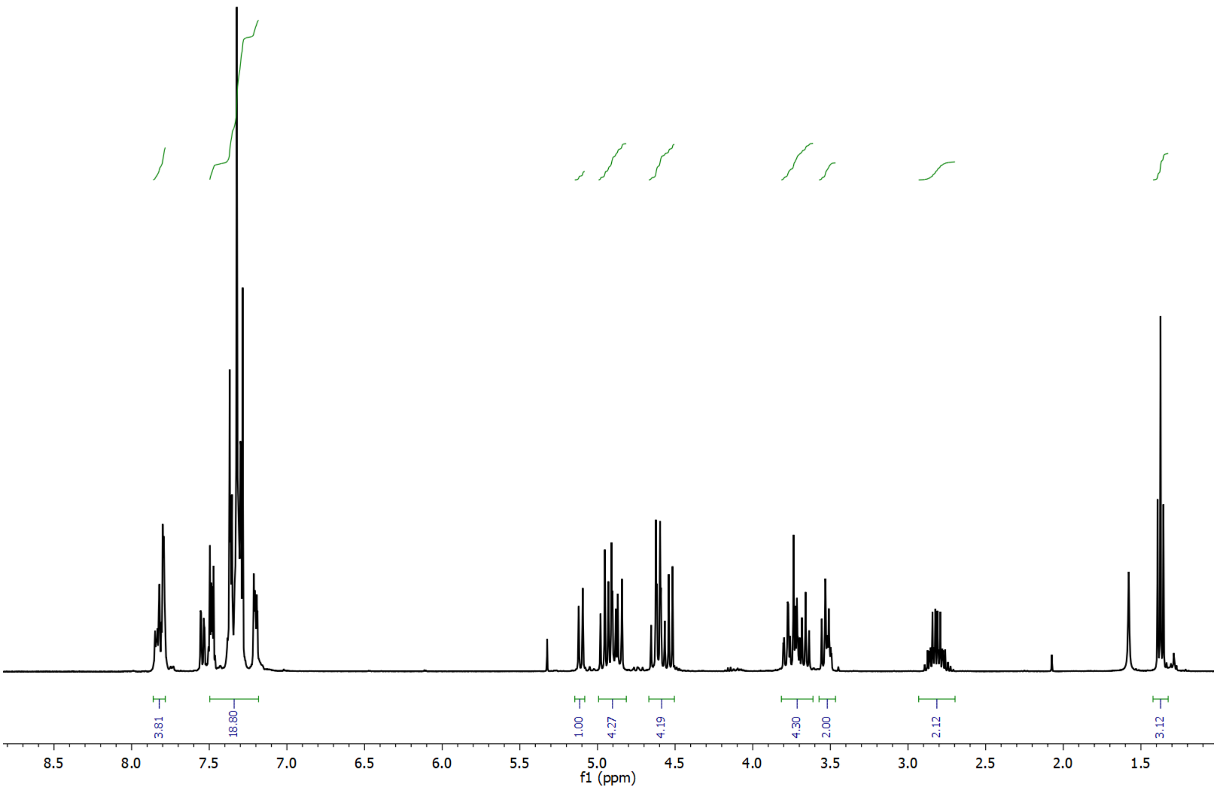

^{13}C NMR of S-4 (101 MHz, CDCl$_3$)

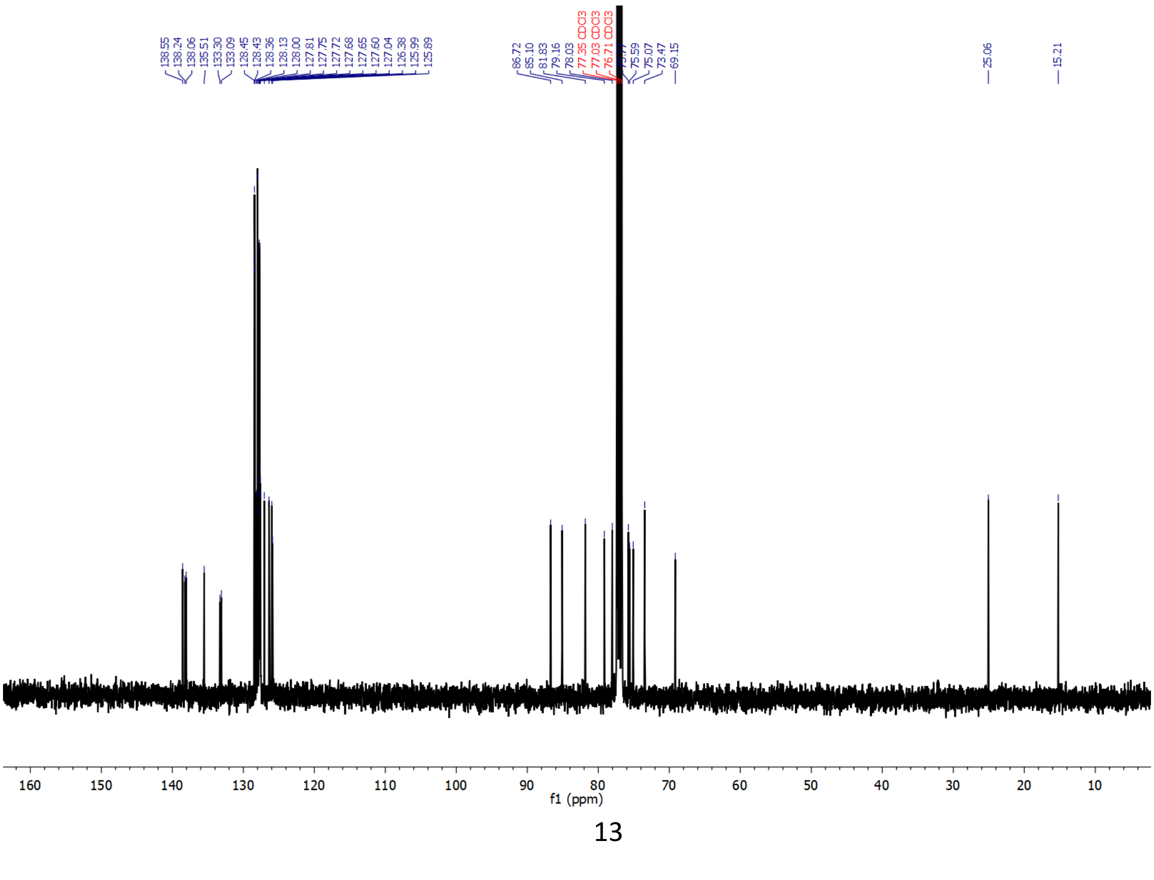

COSY NMR of S-4 (CDCl3)

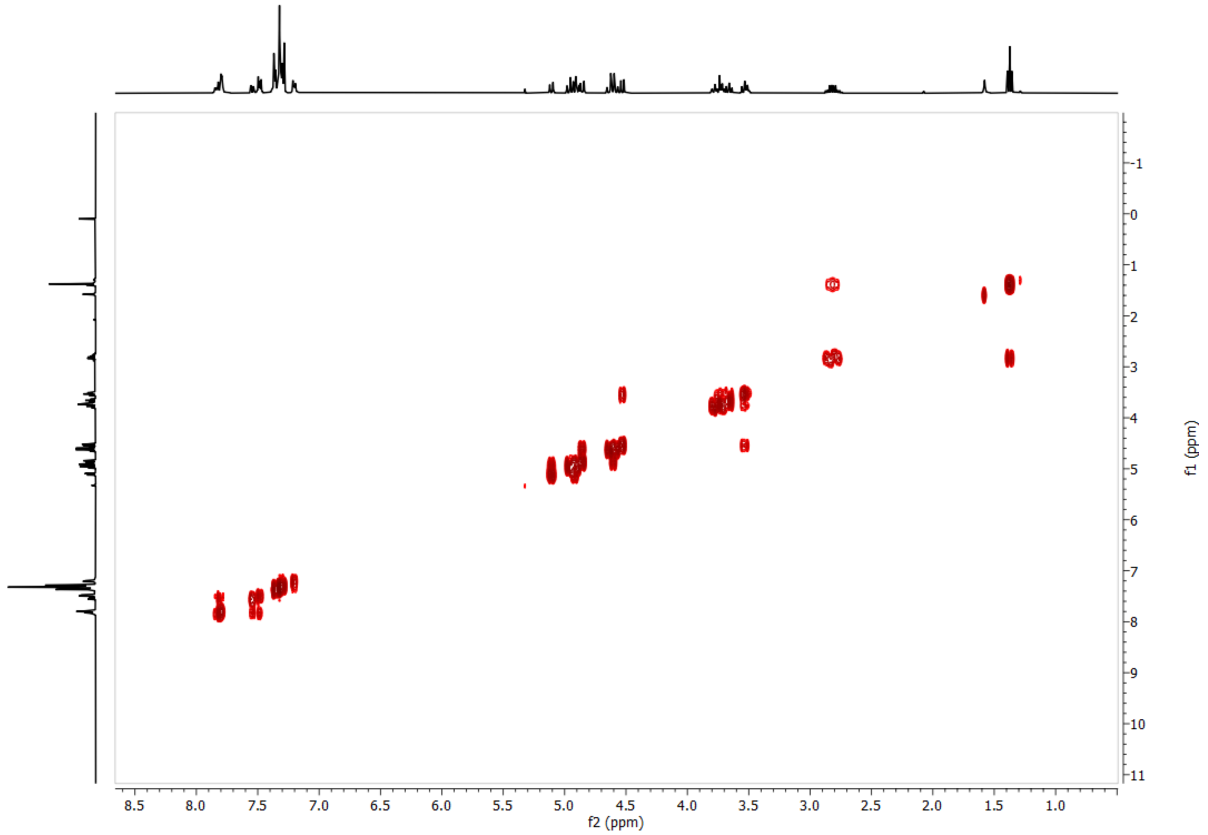

HSQC NMR of S-4 (CDCl3)

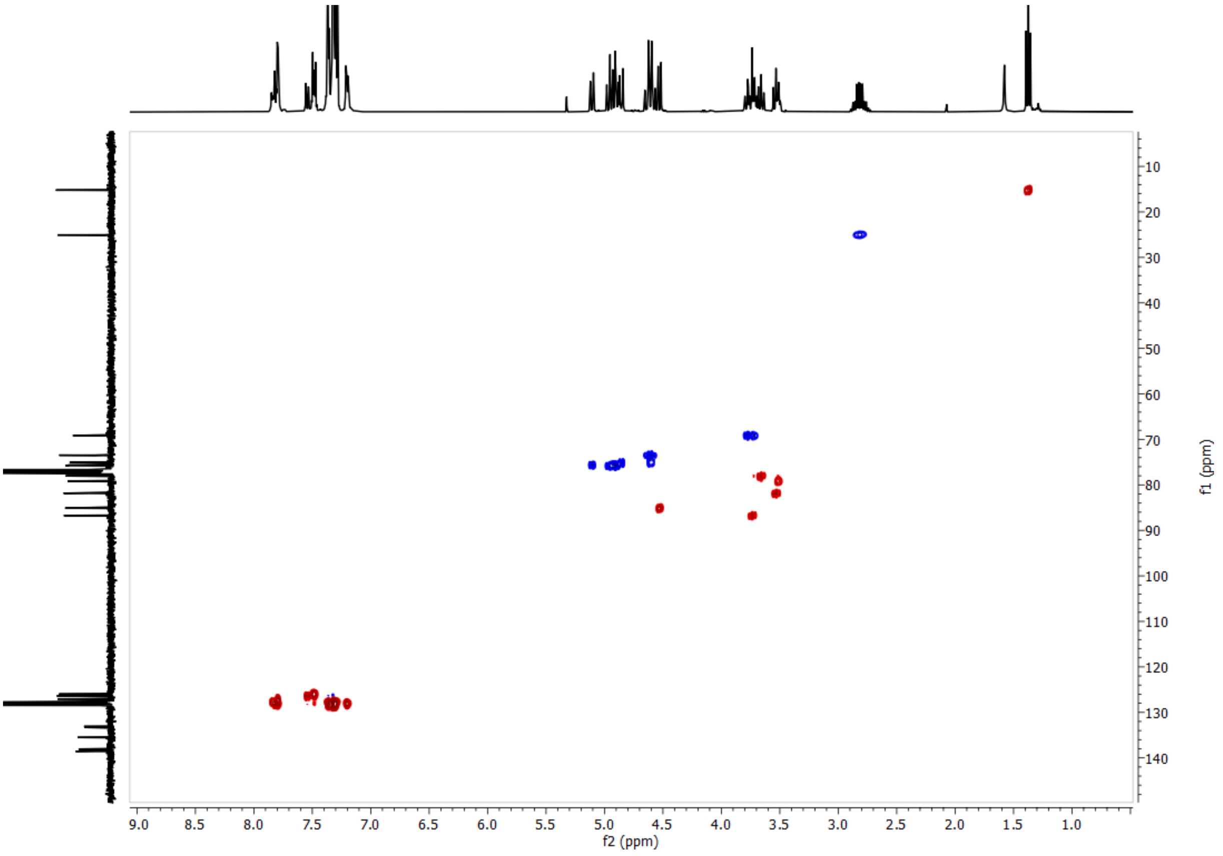

3.1.4 S-5

Synthesis of ethyl 3,4,6-tri-*O*-benzyl-1-thio-β-D-glucopyranoside

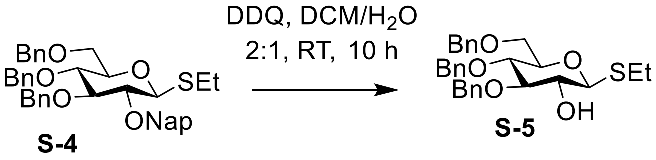

To a solution of ethyl 3,4,6-tri-*O*-benzyl-2-*O*-(2-methyl)naphthyl-1-thio-β-D-glucopyranoside (1.97 g, 3.11 mmol, 1.0 equiv) in a mixture of DCM/H_2O (2:1, v/v, 30 mL, reaction concentration 0.1 M), 2,3-dichloro-5,6-dicyano-1,4-benzoquinone (DDQ) (1,06 g, 4.7 mmol, 1.5 equiv) was added under Ar. The reaction mixture was stirred at RT for 10 h. When TLC showed completion of the reaction, the mixture was diluted with H_2O and DCM. The organic layers were washed with 10% (w/v) $Na_2S_2O_3$ and saturated $NaHCO_3$, dried over Na_2SO_4, filtered and concentrated. The crude was purified by silica gel column chromatography (Hexane/Ethyl acetate, 3:1) to give **S-5** as a white solid (0.64 g, 43 %).

^{1}H NMR (400 MHz, $CDCl_3$) δ 7.48 – 7.15 (m, 15H), 5.03 – 4.83 (m, 3H), 4.70 – 4.50 (m, 3H), 4.33 (d, *J* = 9.0 Hz, 1H), 3.86 – 3.45 (m, 6H), 2.77 (dd, *J* = 7.5, 4.9 Hz, 2H), 1.35 (td, *J* = 7.4, 1.4 Hz, 3H).

^{13}C NMR (101 MHz, $CDCl_3$) δ 138.61, 138.20, 138.08, 128.58, 128.51, 128.48, 128.42, 128.36, 128.00, 127.97, 127.91, 127.80, 127.78, 127.76, 127.61, 86.11, 86.05, 79.47, 77.45, 77.35, 77.03, 76.71, 75.23, 75.10, 73.46, 73.28, 69.06, 24.29, 15.43, 1.04.

ESI-HRMS m/z 517.2027 $[M+Na]^+$ ($C_{29}H_{34}O_5SNa$ requires 517.2019).

^{1}H NMR of S-5 (400 MHz, $CDCl_3$)

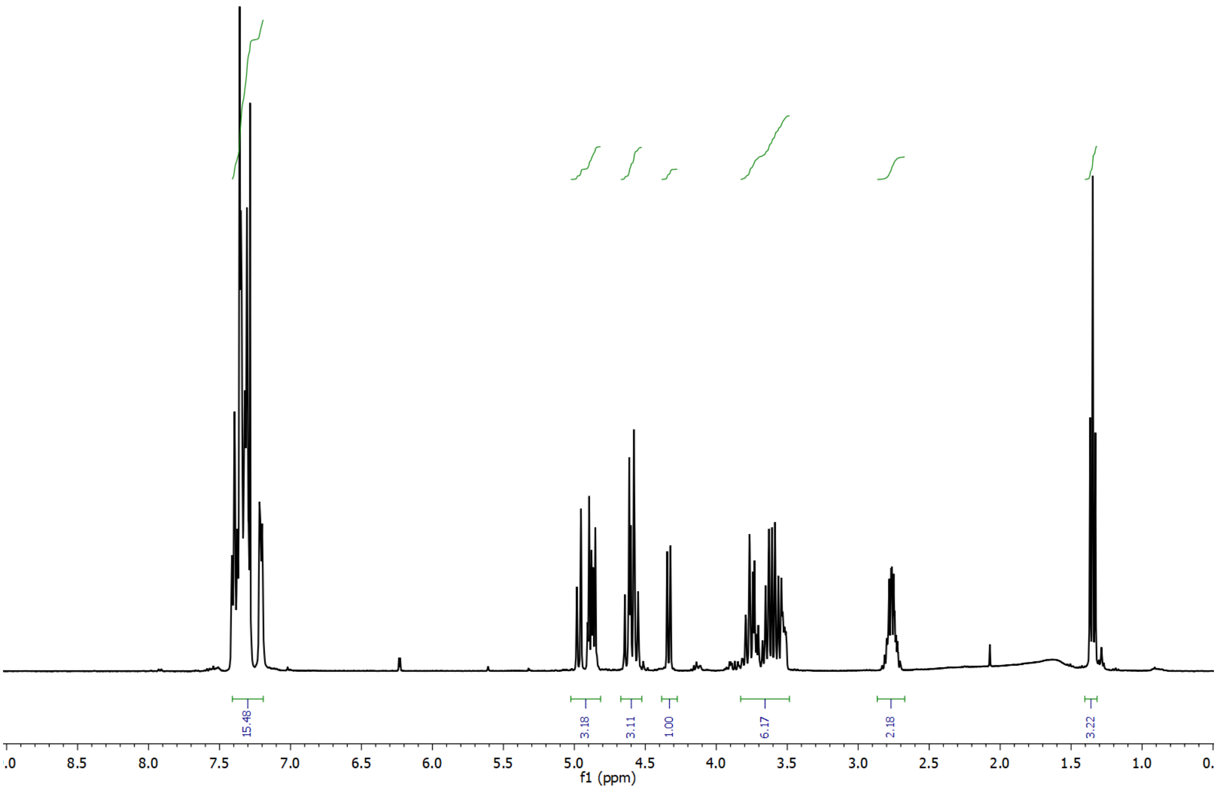

^{13}C NMR of S-5 (101 MHz, $CDCl_3$)

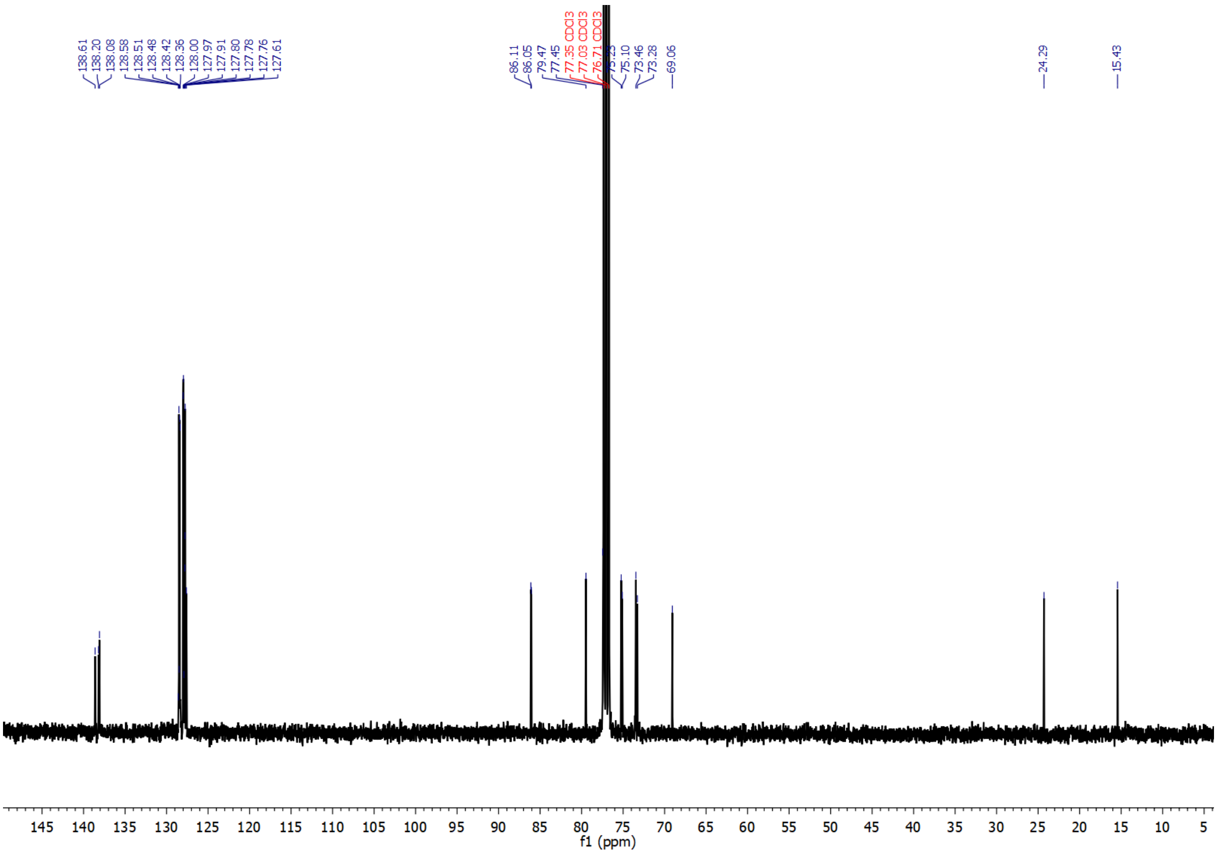

COSY NMR of S-5 ($CDCl_3$)

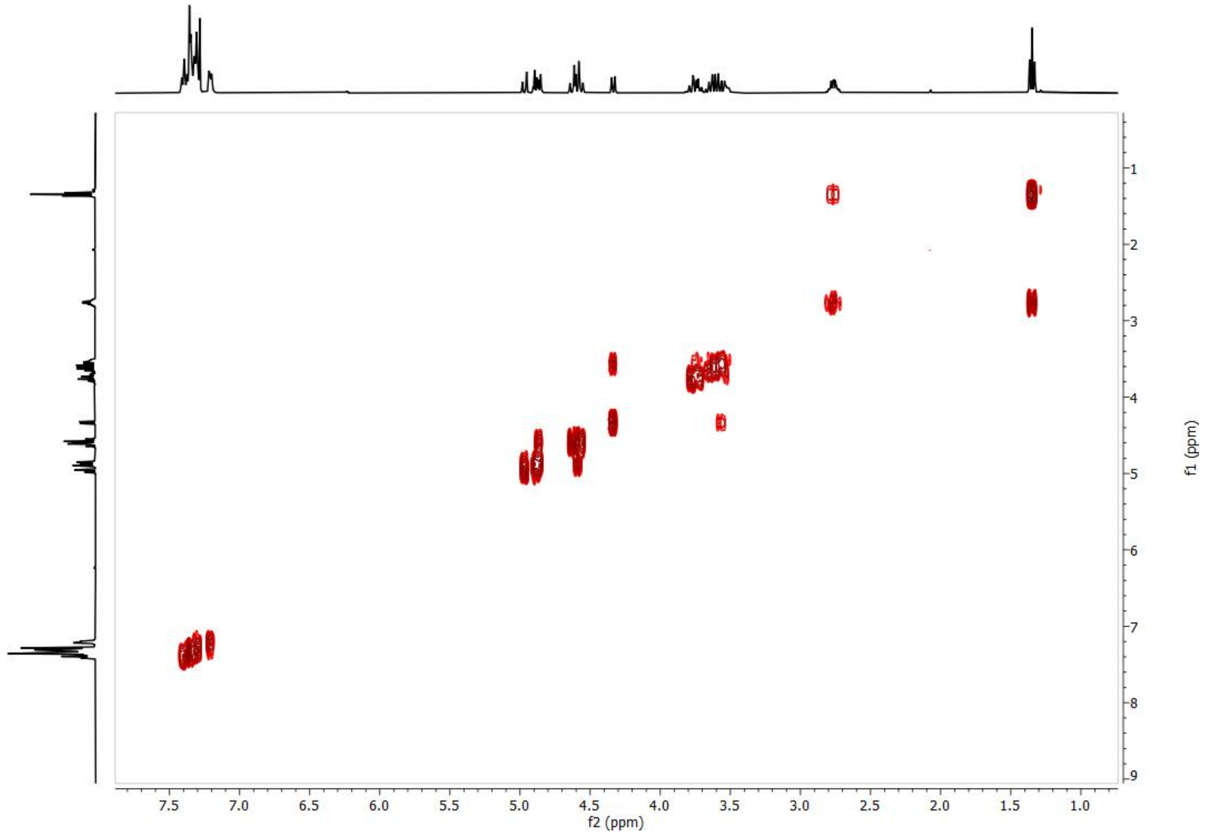

HSQC NMR of S-5 ($CDCl_3$)

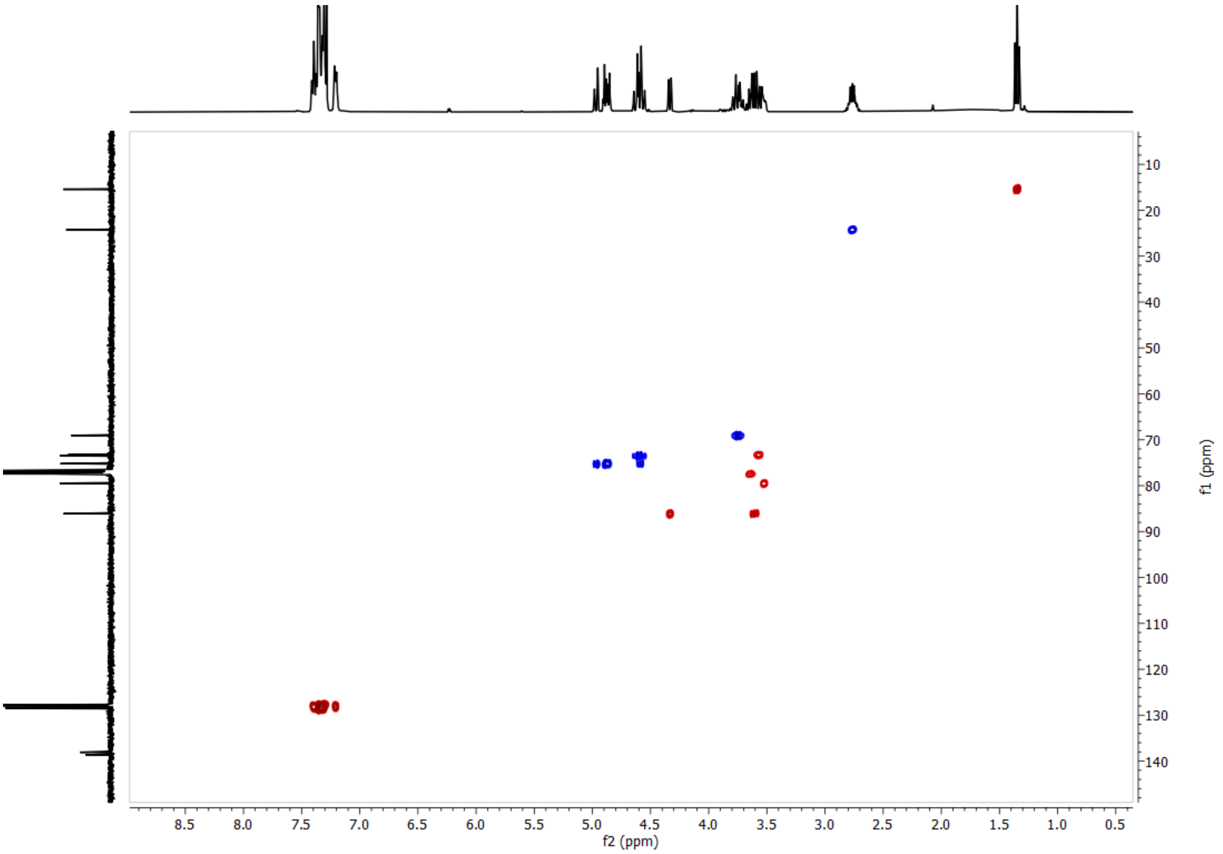

3.1.5 BB2

Synthesis of ethyl 3,4,6-tri-*O*-benzyl-2-*O*-levulinoyl-1-thio-β-D-glucopyranoside

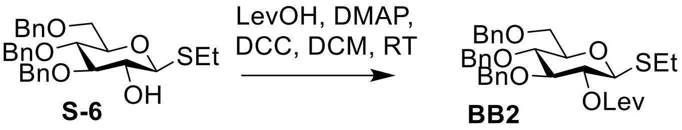

Ethyl 3,4,6-tri-*O*-benzyl-1-thio-β-D-glucopyranoside (641 mg, 1.3 mmol) was dissolved in anhydrous DCM (5 mL) under Ar atmosphere. Levulinic acid (LevOH, 267 μL, 2.6 mmol), *N*,*N′*-dicyclohexylcarbodiimide (DCC, 482 mg, 2.3 mmol), and 4-dimethylaminopyridine (DMAP, 32 mg, 0.26 mmol) were added to the stirred reaction mixture at RT. After 2 h, the reaction was passed through a short plug of silica gel. The crude reaction mixture was concentrated under vacuum and purified by silica gel column chromatography (Hexane/Ethyl acetate, 3:1) to give **BB-2** as a white solid (0.51 g, 66 %).

^{1}H NMR (400 MHz, $CDCl_3$) δ 7.43 – 7.16 (m, 15H), 5.13 – 5.00 (m, 1H), 4.87 – 4.71 (m, 3H), 4.67 – 4.54 (m, 3H), 4.38 (d, J = 10.0 Hz, 1H), 3.84 – 3.66 (m, 4H), 3.52 (ddd, J = 7.9, 4.3, 2.2 Hz, 1H), 2.86 – 2.63 (m, 4H), 2.63 – 2.43 (m, 2H), 2.18 (s, 3H), 1.28 (t, J = 7.4 Hz, 3H).

^{13}C NMR (101 MHz, $CDCl_3$) δ 206.18, 171.56, 138.25, 138.17, 137.93, 128.44, 128.40, 128.37, 128.03, 127.90, 127.85, 127.71, 127.70, 127.61, 84.32, 83.39, 79.49, 77.83, 77.35, 77.03, 76.71, 75.18, 75.10, 73.47, 72.18, 68.89, 37.92, 29.86, 28.11, 23.86, 14.95.

ESI-HRMS m/z 615.2405 $[M+Na]^+$ ($C_{34}H_{40}O_7SNa$ requires 615.2387).

^{1}H NMR of BB2 (400 MHz, $CDCl_3$)

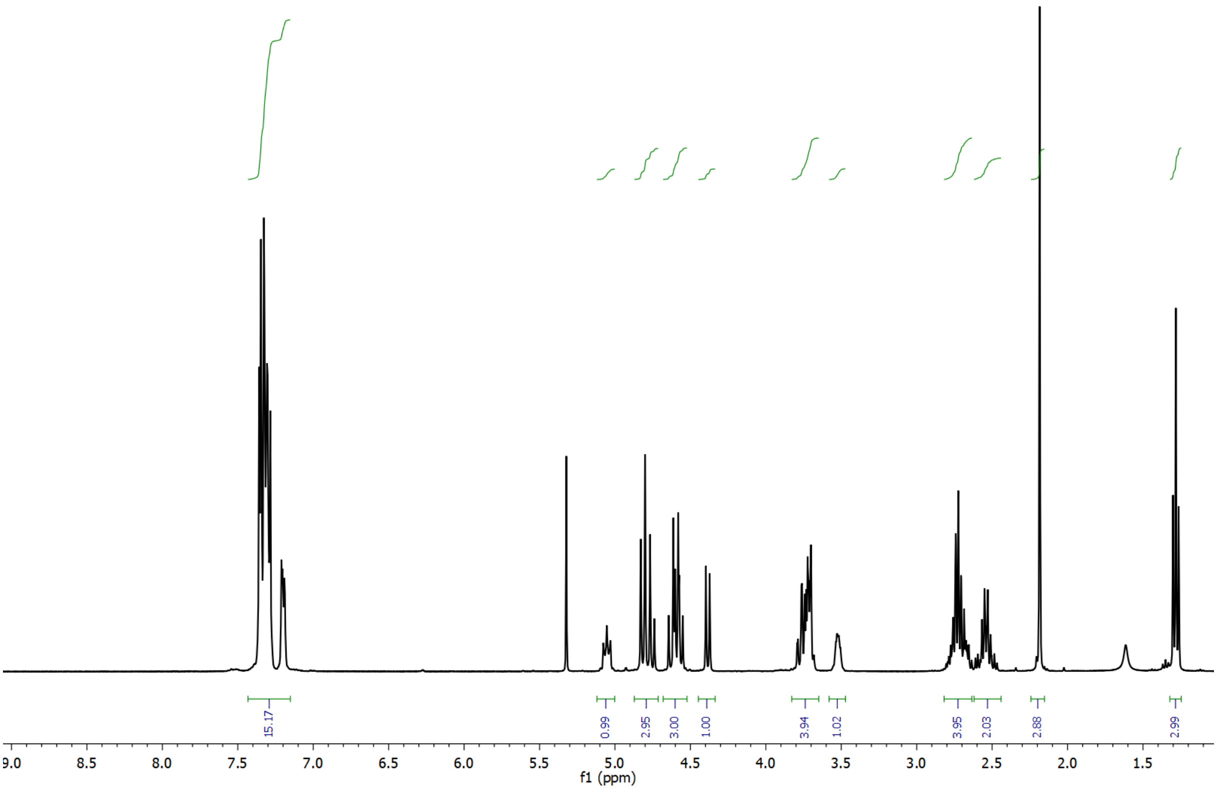

^{13}C NMR of BB2 (101 MHz, $CDCl_3$)

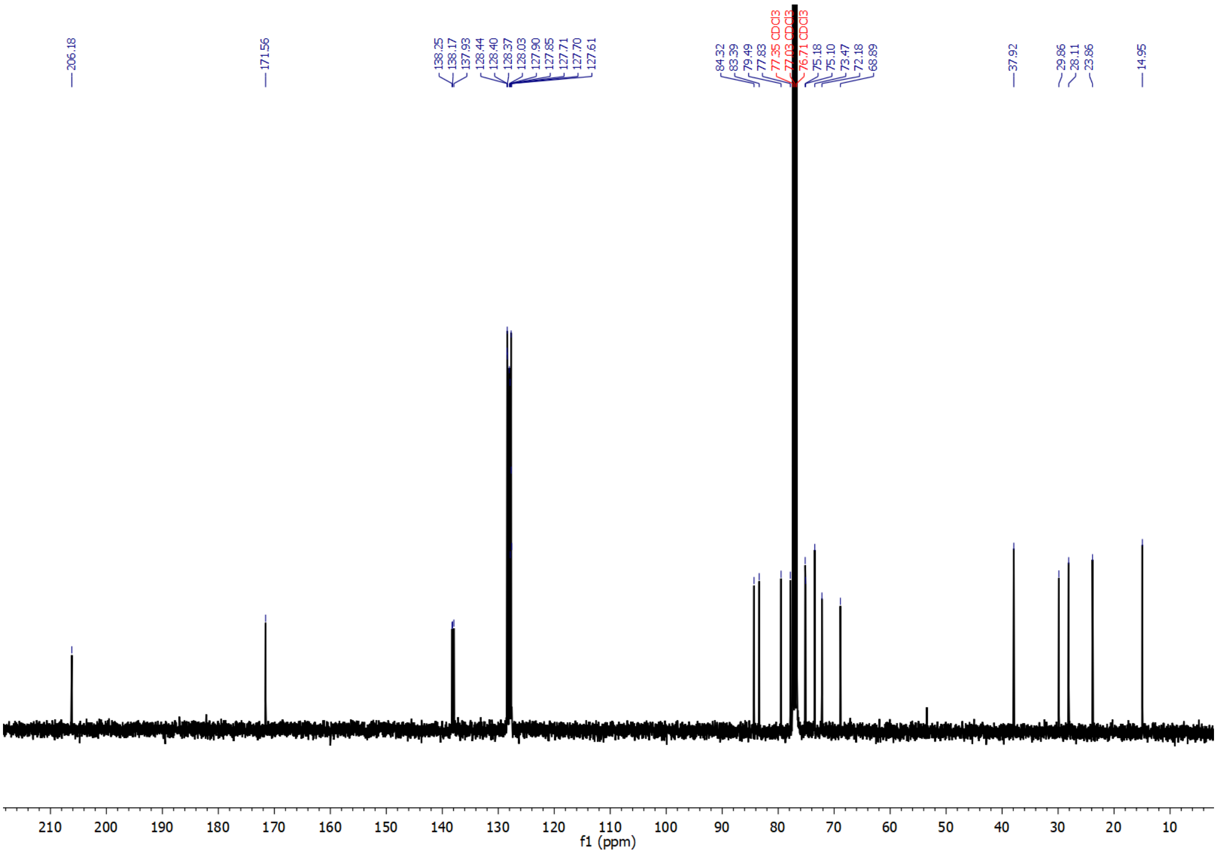

COSY NMR of BB2 ($CDCl_3$)

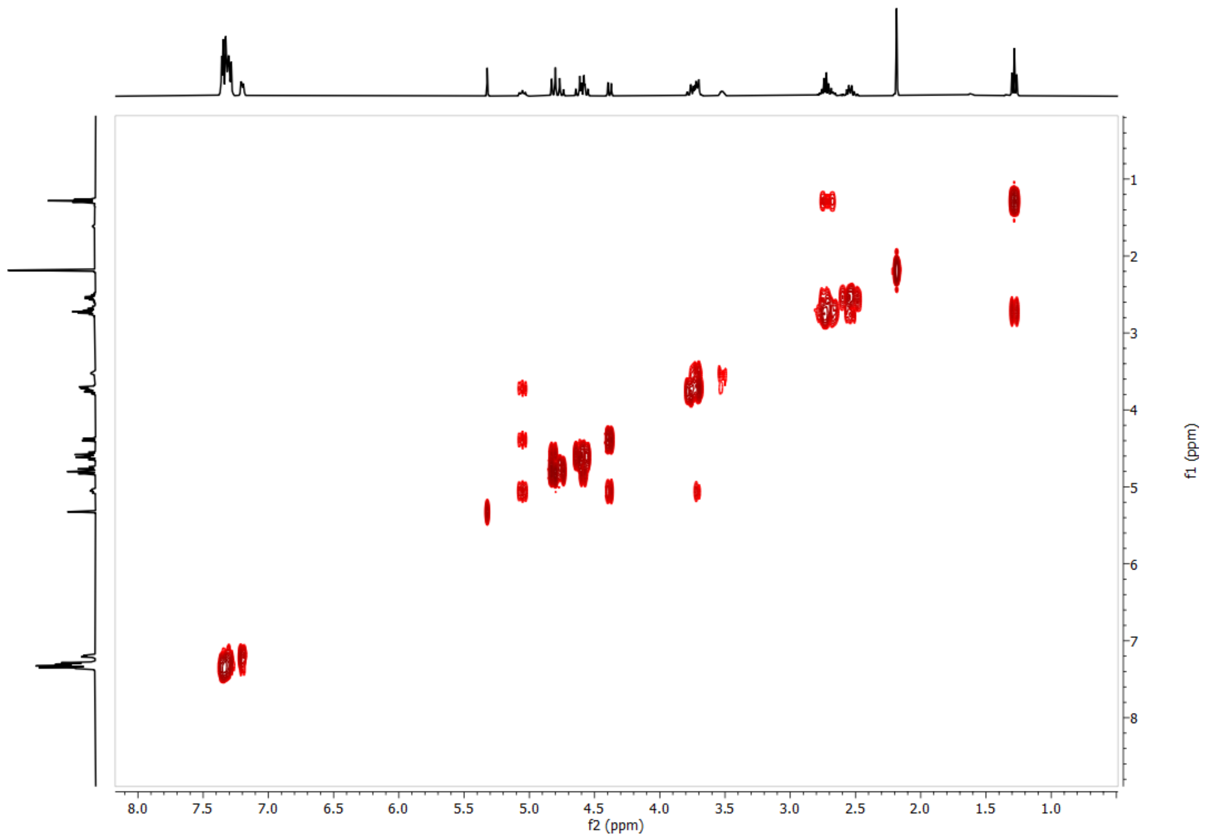

HSQC NMR of BB2 ($CDCl_3$)

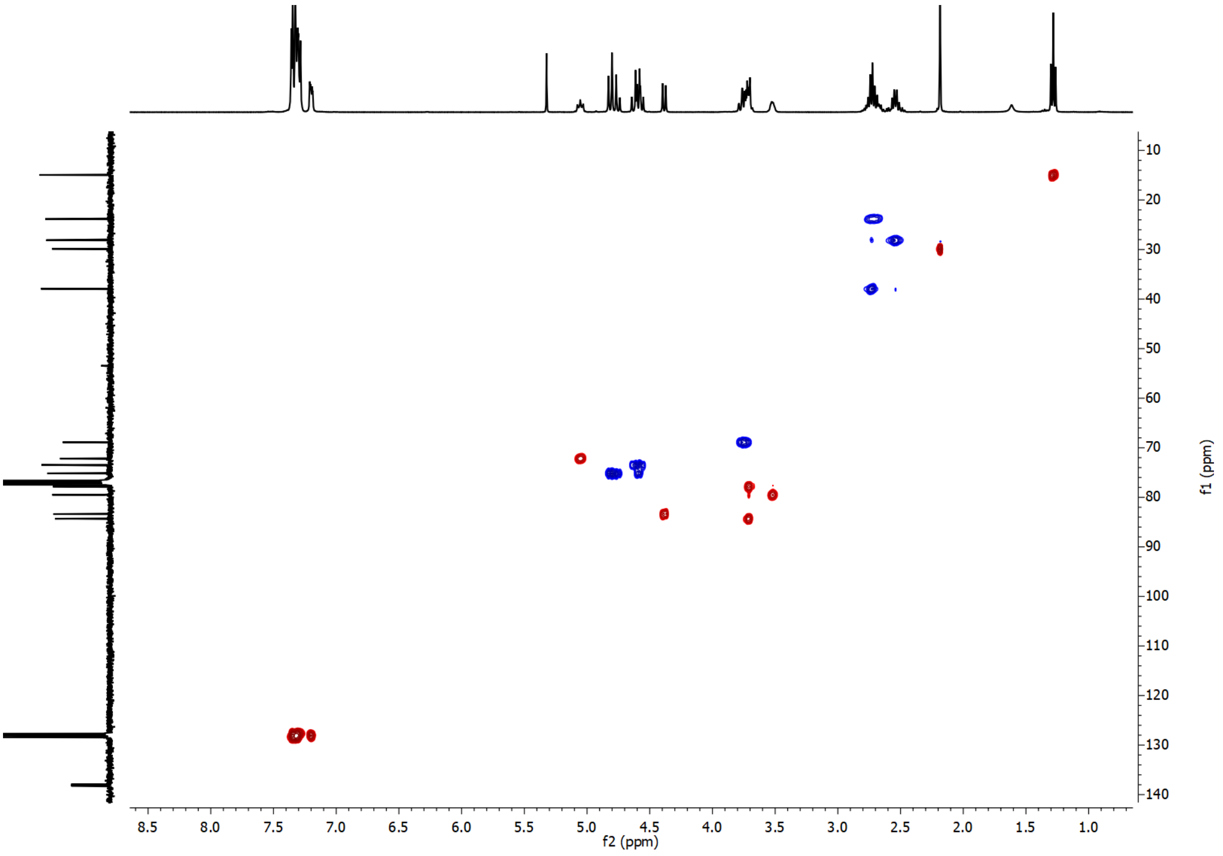

3.2 Synthesis of BB3

3.2.1 S-7

Synthesis of ethyl 2-*O*-(2-methyl)naphthyl-1-thio-β-L-fucopyranoside

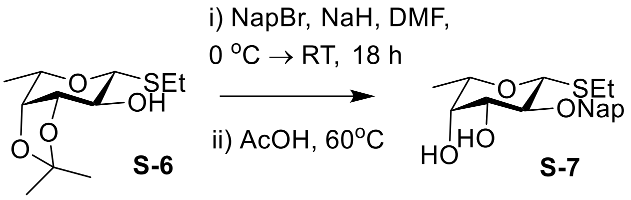

Ethyl 3,4-*O*-isopropylidene-1-thio-β-L-fucopyranoside S-6 was synthesized according to previously reported procedure.[6]

Ethyl 3,4-*O*-isopropylidene-1-thio-β-L-fucopyranoside (3.76 g, 15.2 mmol) and 2-(bromomethyl)-naphthalene (6.7 g, 30.3 mmol) were dissolved in DMF (84 mL) and cooled to 0 °C. Sodium hydride (728 mg, 30.3 mmol) was added and the mixture was stirred at RT overnight. Then, sat. NH_4Cl solution (30 mL) was added and the mixture was extracted with diethyl ether (2 x 30 mL). The combined organic layers were dried over Na_2SO_4. The crude reaction mixture was concentrated under vacuum and purified by silica gel column chromatography (Hexane/Ethyl acetate, 10:1) to give **S-7** as a white solid (5.8 g, 99 %).

A solution of ethyl 3,4-*O*-isopropylidene-2-*O*-(2-methyl)naphthyl-1-thio-β-L-fucopyranoside (5.8 g, 15.1 mmol) in HOAc (92 mL) and water (9.2 mL) was heated at 60 °C for 2 h. The mixture was co-evaporated with toluene. The residue was purified using flash chromatography (Hexane/Ethyl acetate, 2:1) to give **S-8** as a white solid (5.2 g, 100 %).

^{1}H NMR (400 MHz, $CDCl_3$) δ 7.98 – 7.38 (m, 7H), 5.14 (d, *J* = 11.2 Hz, 1H), 4.89 (d, *J* = 11.2 Hz, 1H), 4.45 (d, *J* = 9.6 Hz, 1H), 3.83 – 3.46 (m, 4H), 2.81 (ddt, *J* = 14.9, 7.4, 3.7 Hz, 2H), 1.42 – 1.32 (m, 6H).

^{13}C NMR (101 MHz, $CDCl_3$) δ 135.49, 133.31, 133.14, 128.46, 128.00, 127.73, 127.21, 126.20, 126.17, 126.08, 84.76, 78.93, 77.36, 77.05, 76.73, 75.40, 75.21, 74.39, 71.83, 25.07, 16.57, 15.03, 1.04.

ESI-HRMS m/z 371.1278 $[M+Na]^+$ ($C_{19}H_{24}O_4SNa$ requires 371.1287).

^{1}H NMR of S-7 (400 MHz, $CDCl_3$)

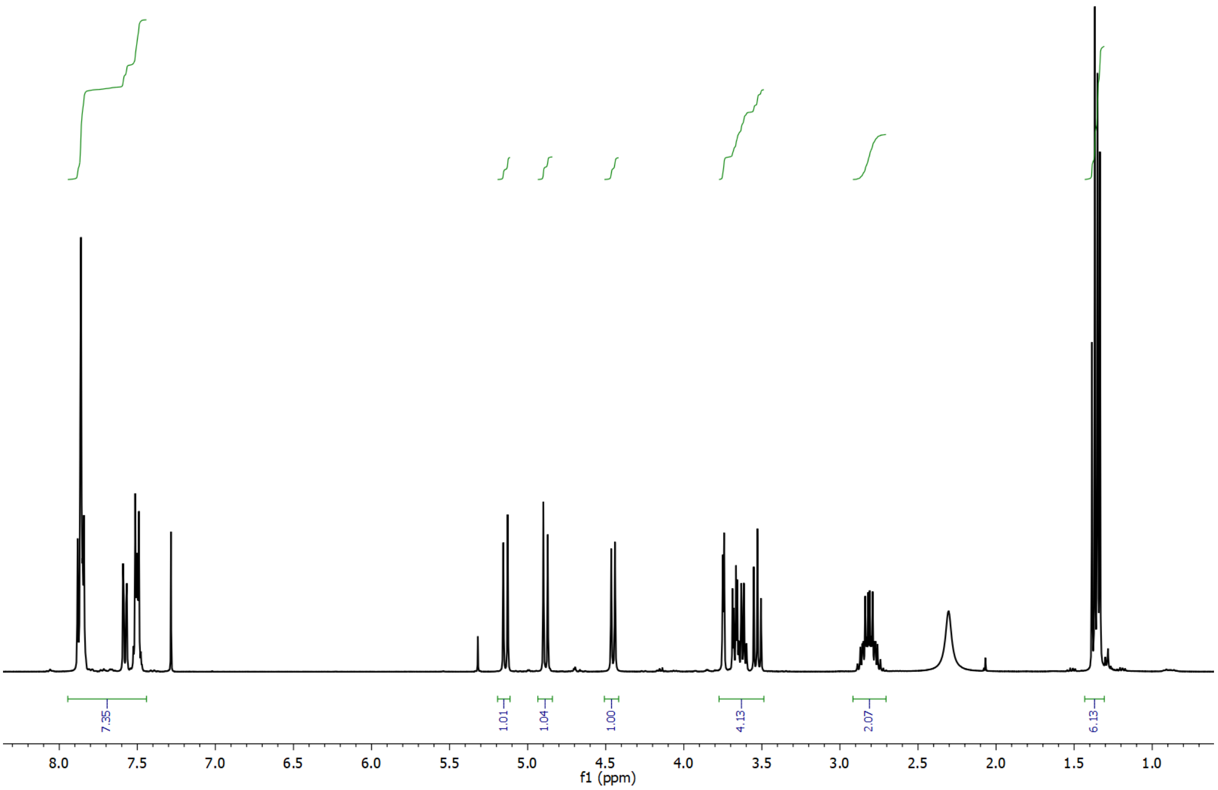

^{13}C NMR of S-7 (101 MHz, $CDCl_3$)

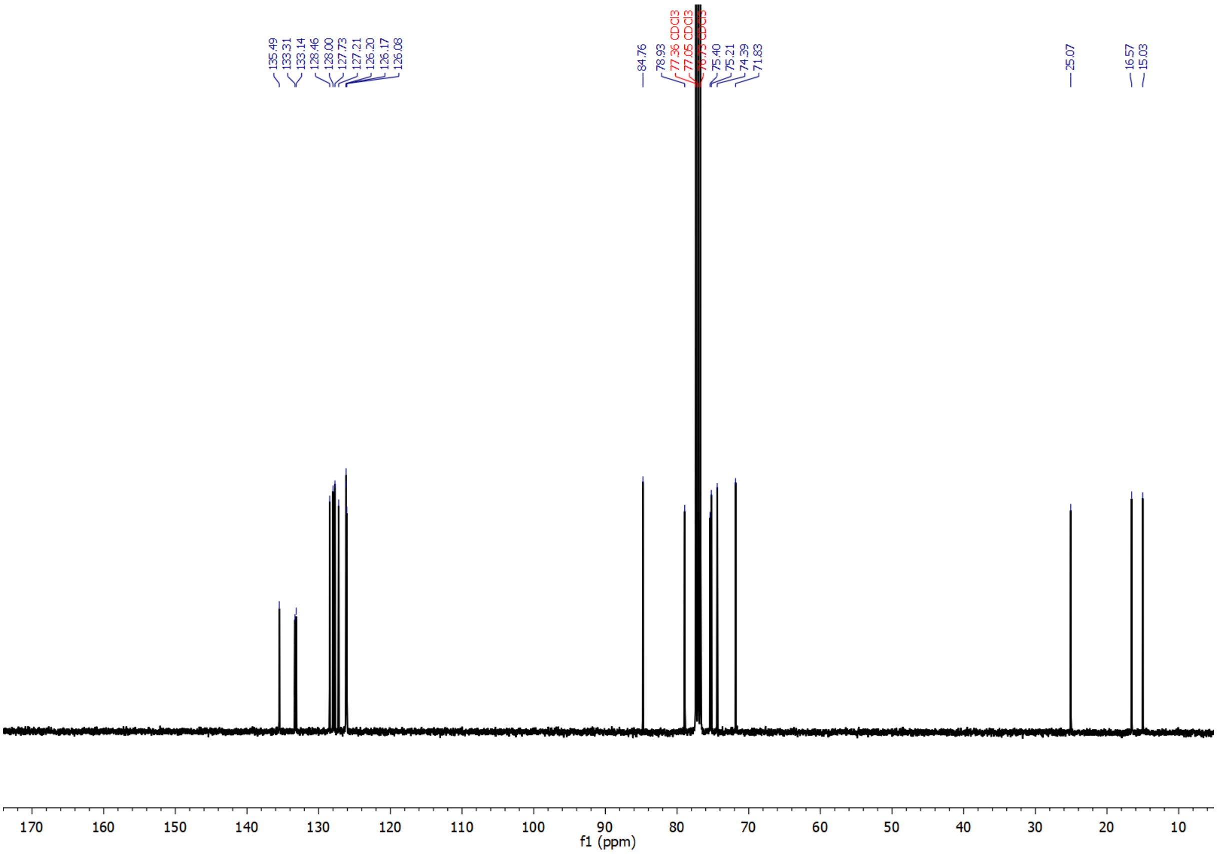

COSY NMR of S-7 ($CDCl_3$)

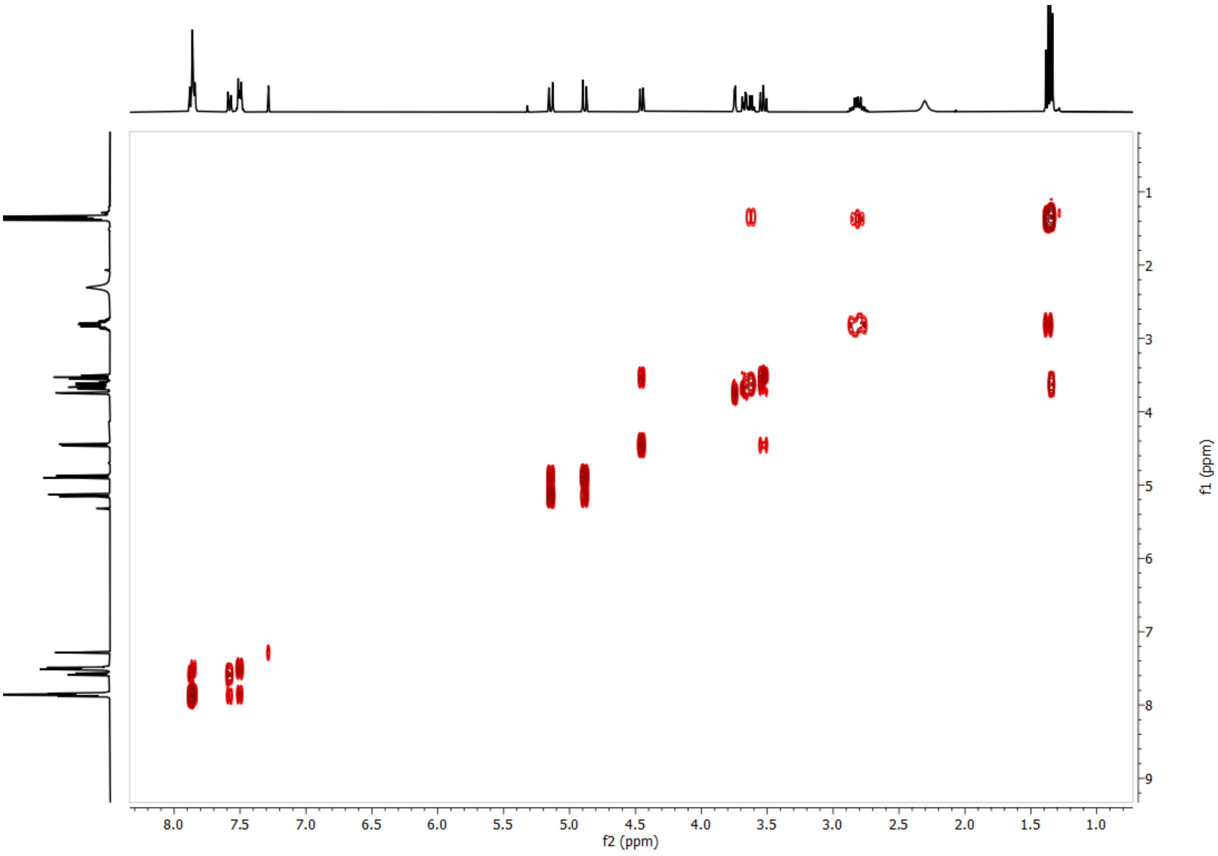

HSQC NMR of S-7 ($CDCl_3$)

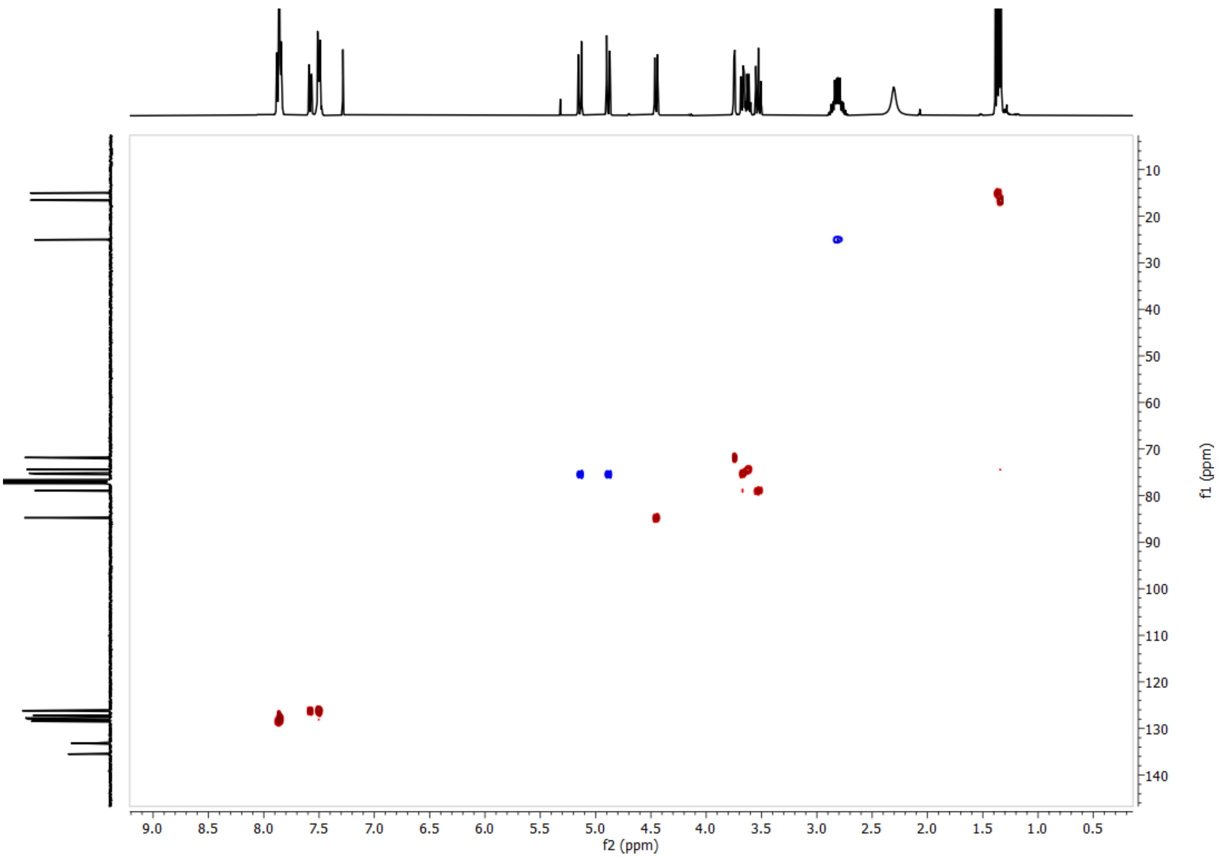

3.2.2 S-8

Synthesis of ethyl 3-*O*-benzyl-2-*O*-(2-methyl)naphthyl-1-thio-β-L-fucopyranoside

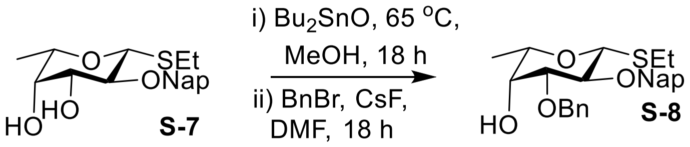

Ethyl 2-*O*-(2-methyl)naphthyl-1-thio-β-L-fucopyranoside (5.2 g, 15.1 mmol) was dissolved in MeOH (84 mL), di-n-butyltin oxide (3.8 g, 21.2 mmol) was added and the reaction mixture heated to 65 °C for 2 h. The reaction mixture was then cooled, concentrated in vacuo and the crude product was used in the next step without further purification. The crude product was dissolved in DMF (84 mL), benzyl bromide (10.4 g, 60,6 mmol) and cesium fluoride (3.45 g, 22.7 mmol) were added and the mixture stirred at RT for 6 h under Ar atmosphere. The reaction mixture was concentrated in vacuo and the residue dissolved in DCM (120 mL). The organic layer was washed with water, dried over $MgSO_4$, filtered and concentrated in vacuo. The crude was purified by silica gel column chromatography (Hexane/Ethyl acetate, 4:1) to give **S-9** as a white solid (3.9 g, 60 %).

^{1}H NMR (400 MHz, $CDCl_3$) δ 7.91 – 7.32 (m, 12H), 5.13 – 4.91 (m, 2H), 4.76 (d, *J* = 2.8 Hz, 1H), 4.45 (d, *J* = 9.7 Hz, 1H), 4.15 (q, *J* = 7.1 Hz, 1H), 3.86 (dd, *J* = 3.3, 1.1 Hz, 1H), 3.76 – 3.51 (m, 3H), 2.80 (dd, *J* = 16.2, 7.4 Hz, 2H), 1.44 – 1.24 (m, 6H).

^{13}C NMR (101 MHz, $CDCl_3$) δ 137.81, 135.76, 133.32, 133.09, 128.57, 128.52, 128.06, 127.99, 127.97, 127.88, 127.69, 126.98, 126.44, 125.98, 125.85, 84.79, 82.73, 77.89, 77.35, 77.03, 76.71, 75.84, 74.14, 72.12, 69.52, 60.41, 24.74, 21.07, 16.72, 15.03, 14.22, 1.03.

ESI-HRMS m/z 461.1758 $[M+Na]^+$ ($C_{26}H_{30}O_4SNa$ requires 461.1757).

^{1}H NMR of S-8 (400 MHz, $CDCl_3$)

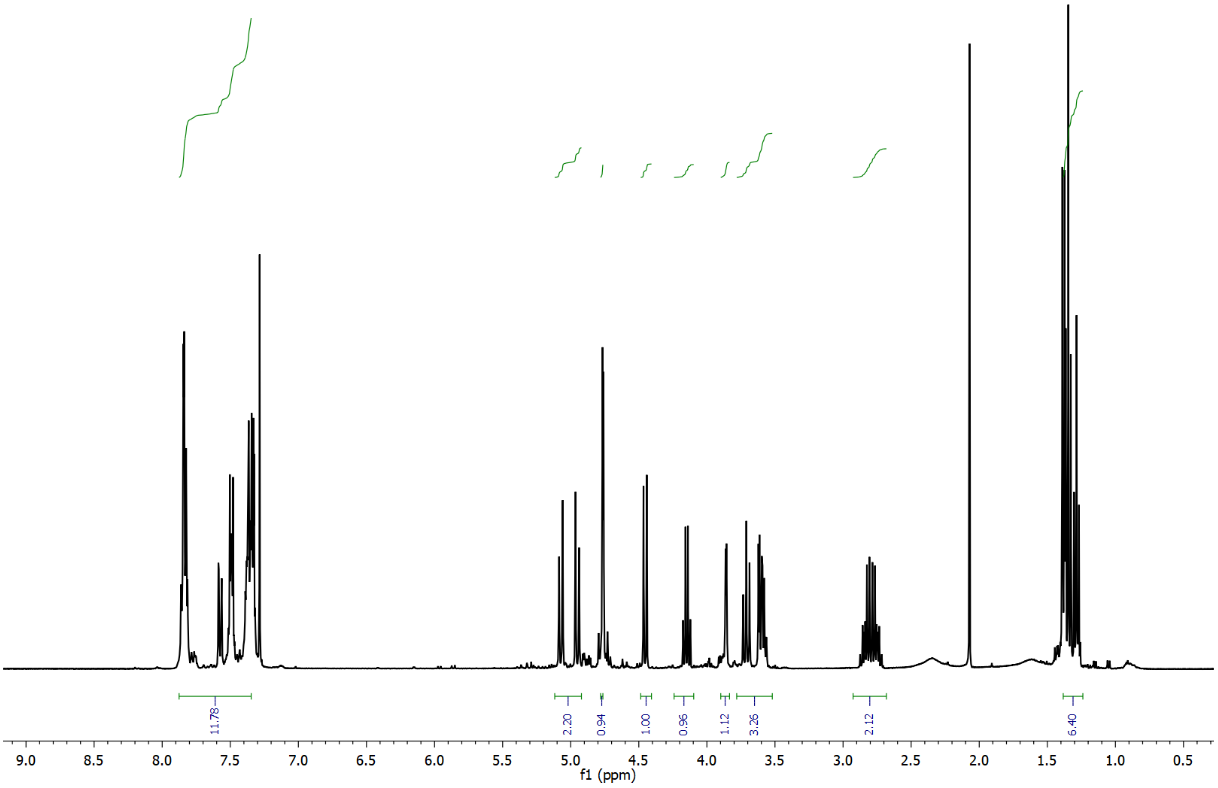

^{13}C NMR of S-8 (101 MHz, $CDCl_3$)

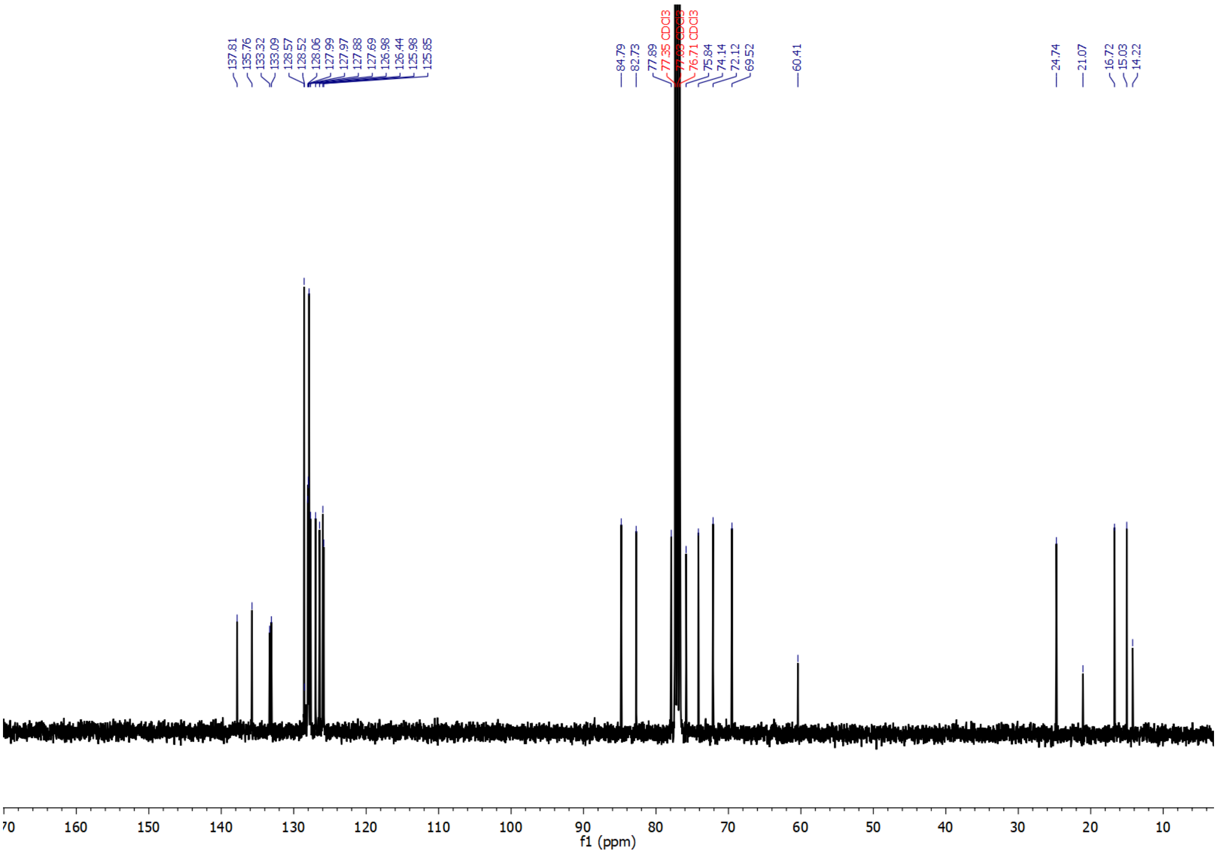

COSY NMR of S-8 ($CDCl_3$)

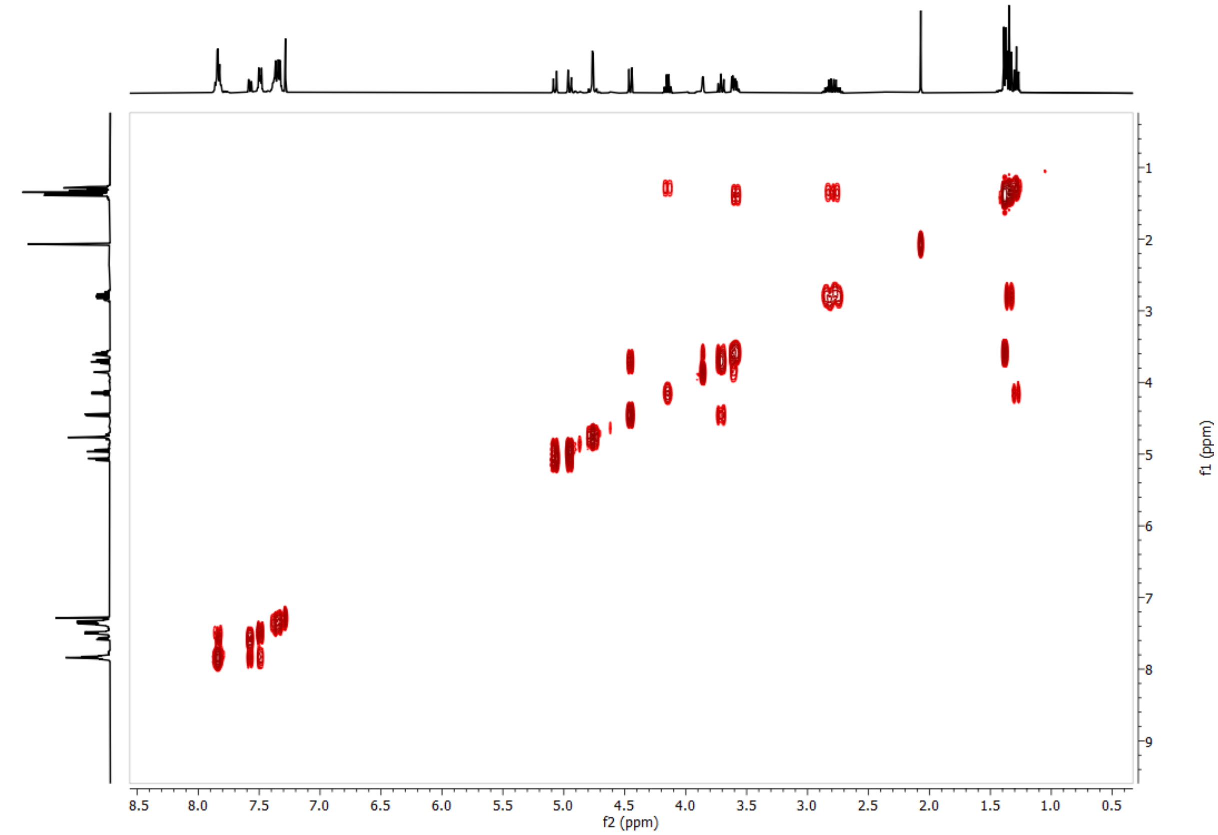

HSQC NMR of S-8 (CDCl3)

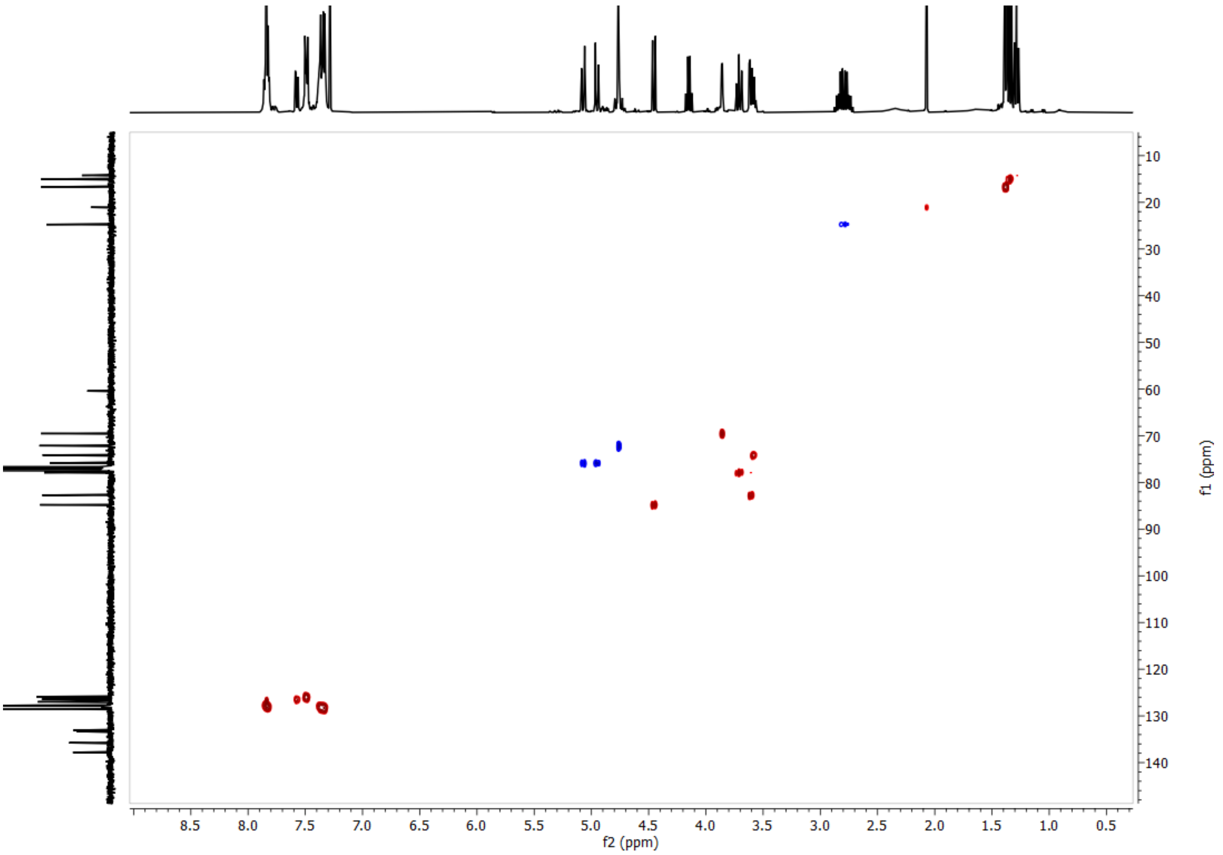

3.2.3 S-9

Synthesis of ethyl 3-*O*-benzyl-4-*O*-fluorenylmethyloxycarbonyl-2-*O*-(2-methyl)naphthyl-1-thio-β-L-fucopyranoside

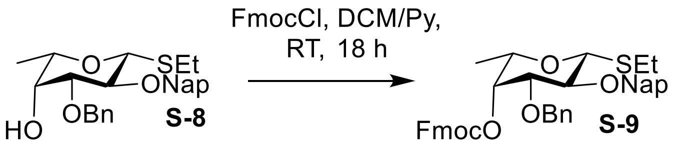

Ethyl 3-*O*-benzyl-2-*O*-(2-methyl)naphthyl-1-thio-β-L-fucopyranoside (3.5 g, 7.9 mmol) was dissolved in DCM (60 mL) under Ar atmosphere. Pyridine (2.7 mL, 32 mmol) and FmocCl (5.67 g, 21.9 mmol) were added to the stirred reaction mixture. The solution was stirred for 3 h and then quenched with an aqueous solution of HCl (1 M). The organic layer was washed once with an aqueous solution of HCl (1 M), once with a saturated aqueous solution of $NaHCO_3$, and once with brine. The crude product was purified with silica gel flash column chromatography (Hexane/Ethyl acetate, 4:1) to give compound **S-10** as a white solid (1.8 g, 77%).

^{1}H NMR (400 MHz, $CDCl_3$) δ 7.89 – 7.17 (m, 20H), 5.23 (dd, *J* = 3.3, 1.0 Hz, 1H), 5.13 – 4.96 (m, 2H), 4.83 (d, *J* = 11.5 Hz, 1H), 4.63 (d, *J* = 11.5 Hz, 1H), 4.56 – 4.35 (m, 3H), 4.30 (d, *J* = 7.5 Hz, 1H), 3.90 – 3.59 (m, 3H), 2.95 – 2.75 (m, 2H), 1.42 – 1.24 (m, 6H).

^{13}C NMR (101 MHz, $CDCl_3$) δ 155.57, 143.66, 143.23, 141.36, 141.25, 137.78, 135.76, 133.33, 133.09, 128.32, 128.04, 127.98, 127.88, 127.84, 127.70, 127.68, 127.18, 126.98, 126.45,

125.96, 125.84, 125.48, 125.24, 120.03, 120.00, 85.02, 81.07, 77.70, 77.35, 77.03, 76.71, 75.98, 74.37, 72.85, 71.96, 70.13, 46.70, 24.87, 16.70, 15.04.

ESI-HRMS m/z 683.2455 $[M+Na]^+$ ($C_{41}H_{40}O_6SNa$ requires 683.2438).

^{1}H NMR of S-9 (400 MHz, $CDCl_3$)

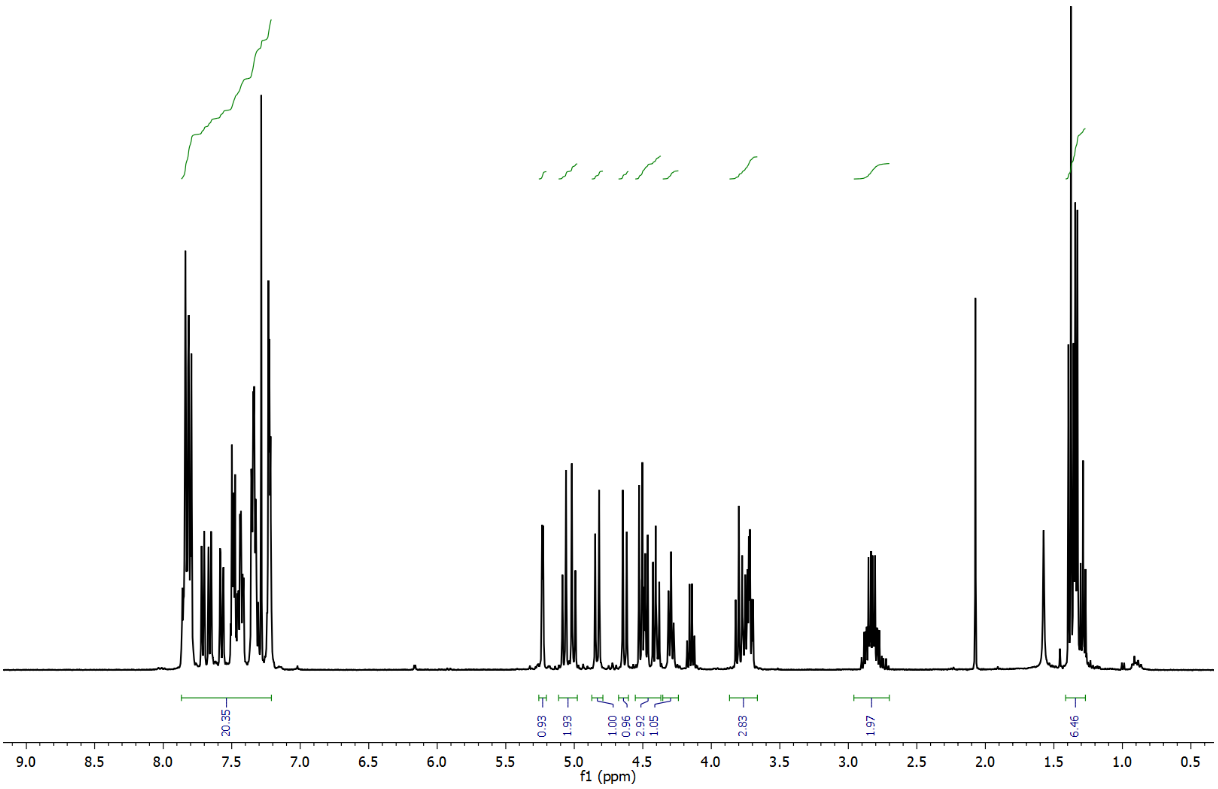

^{13}C NMR of S-9 (101 MHz, $CDCl_3$)

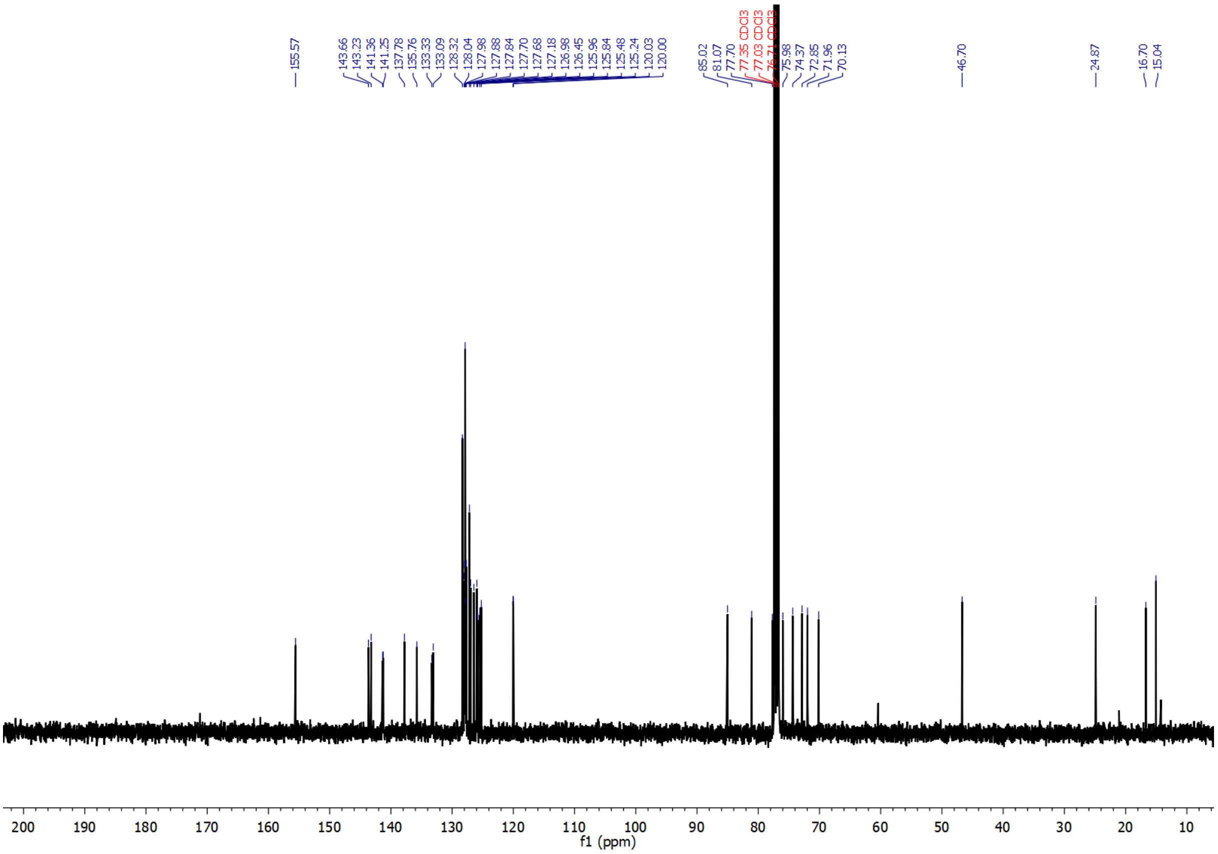

COSY NMR of S-9 ($CDCl_3$)

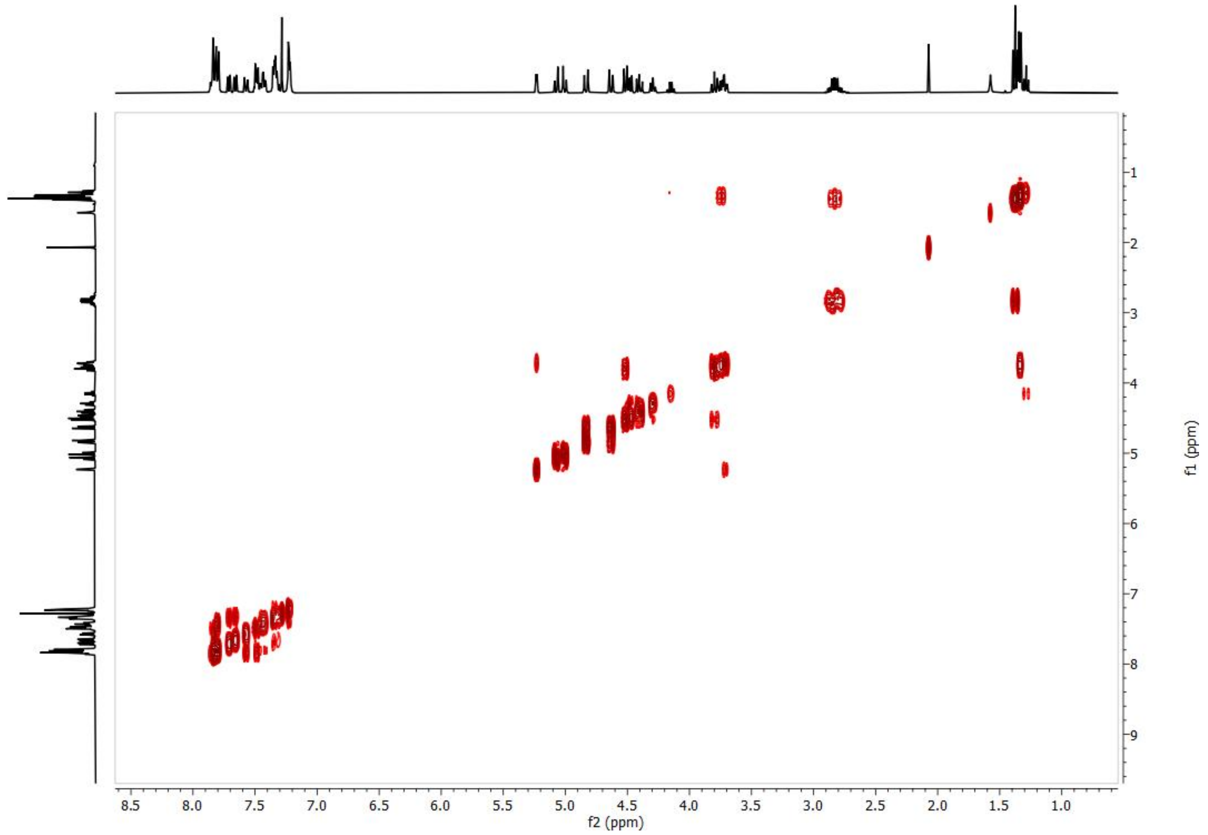

HSQC NMR of S-9 ($CDCl_3$)

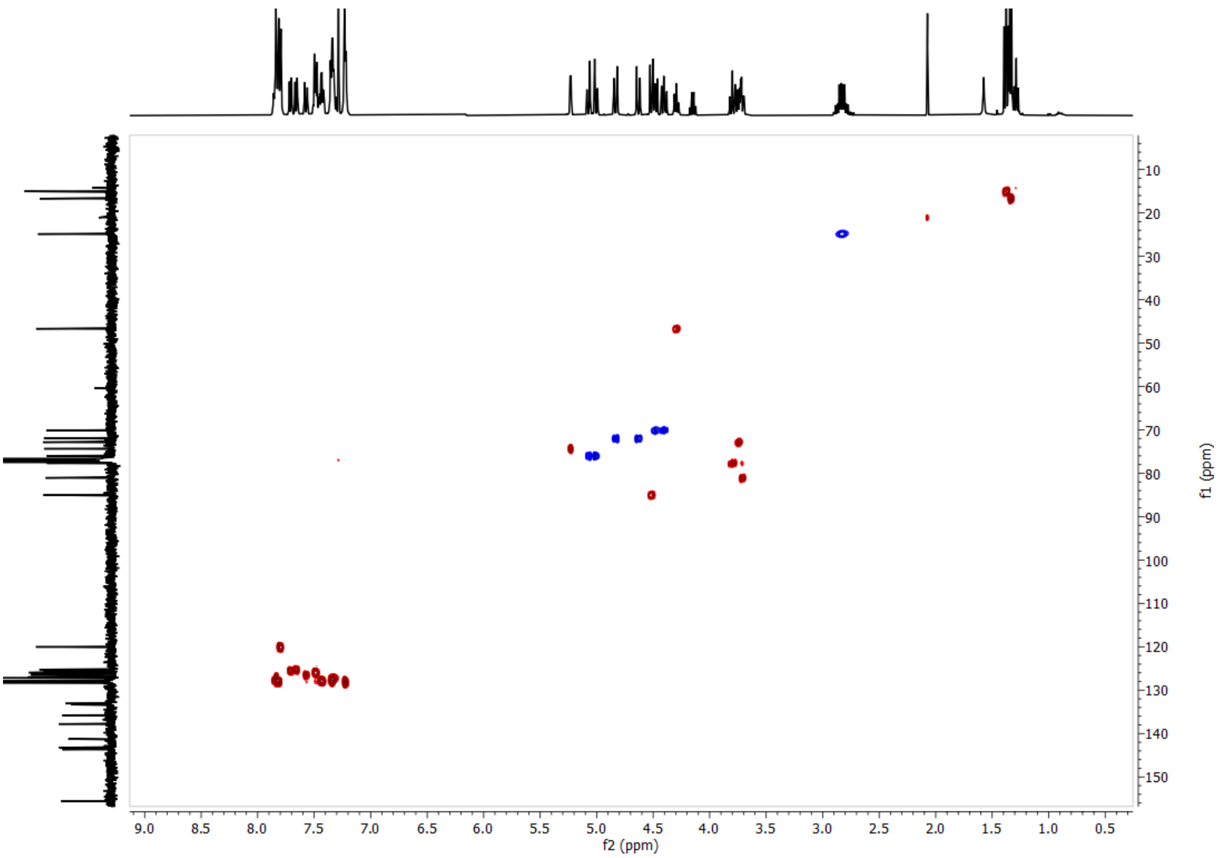

3.2.4 BB3

Synthesis of ethyl 3-*O*-benzyl-2-*O*-benzoyl-4-*O*-fluorenylmethyloxycarbonyl-1-thio-β-L-fucopyranoside

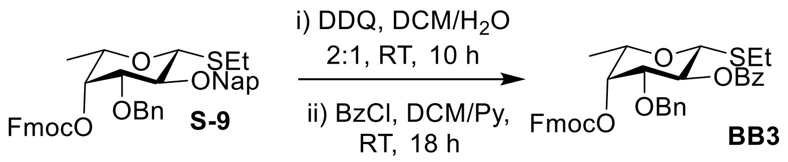

To a solution of ethyl 3-*O*-benzyl-4-*O*-fluorenylmethyloxycarbonyl-2-*O*-(2-methyl)naphthyl-1-thio-β-L-fucopyranoside (1.8 g, 2.75 mmol, 1.0 equiv) in a mixture of DCM/H_2O (2:1, v/v, 12 mL, reaction concentration 0.1 M), 2,3-dichloro-5,6-dicyano-1,4-benzoquinone (DDQ) (933 mg, 1.5 equiv) was added under Ar. The reaction mixture was stirred at RT for 10 h. When TLC showed completion of the reaction, the mixture was diluted with H_2O and DCM. The organic layers were washed with 10% (w/v) $Na_2S_2O_3$ and satd. aq. $NaHCO_3$, dried over Na_2SO_4, filtered and concentrated. The crude was purified by silica gel column chromatography (Hexane/Ethyl acetate, 3:1) to give compound **S-11** as a white solid (746 mg, 52 %).

Ethyl 3-O-benzyl-4-O-fluorenylmethyloxycarbonyl-1-thio-β-L-fucopyranoside (746 g, 1.4 mmol) was dissolved in DCM (8 mL) under Ar atmosphere. Pyridine (0.35 mL, 4 mmol) and BzCl (400 mg, 9.8 mmol) were added to the stirred reaction mixture. The solution was stirred for 24 h and then quenched with an aqueous solution of HCl (1 M). The organic layer was washed once with an aqueous solution of HCl (1 M), once with a saturated aqueous solution of

$NaHCO_3$, and once with brine. The crude product was purified with silica gel flash column chromatography (Toluene) to give **BB3** as white solid (805 mg, 92.8 %).

^{1}H NMR (400 MHz, $CDCl_3$) δ 8.01 – 7.91 (m, 2H), 7.72 – 7.50 (m, 5H), 7.42 – 7.22 (m, 6H), 7.10 – 6.95 (m, 5H), 5.52 (t, *J* = 9.8 Hz, 1H), 5.17 (dd, *J* = 3.3, 1.1 Hz, 1H), 4.61 (d, *J* = 12.6 Hz, 1H), 4.48 – 4.38 (m, 3H), 4.28 – 4.19 (m, 2H), 3.76 – 3.64 (m, 2H), 2.86 – 2.58 (m, 2H), 1.28 (d, *J* = 6.4 Hz, 6H), 1.19 (t, *J* = 7.4 Hz, 3H).

^{13}C NMR (101 MHz, $CDCl_3$) δ 165.27, 155.51, 143.67, 143.12, 141.33, 141.21, 137.27, 133.12, 129.97, 128.36, 128.26, 127.89, 127.87,127.83, 127.71, 127.30, 127.28, 125.70, 125.32, 119.98, 119.95, 83.39, 77.49, 77.37, 77.25, 77.05, 76.73, 73.63, 73.26, 70.95, 70.28, 69.16, 46.58, 23.73, 16.76, 14.80.

ESI-HRMS m/z 647.2095 $[M+Na]^+$ ($C_{37}H_{36}O_7SNa$ requires 647.2074).

^{1}H NMR of BB3 (400 MHz, $CDCl_3$)

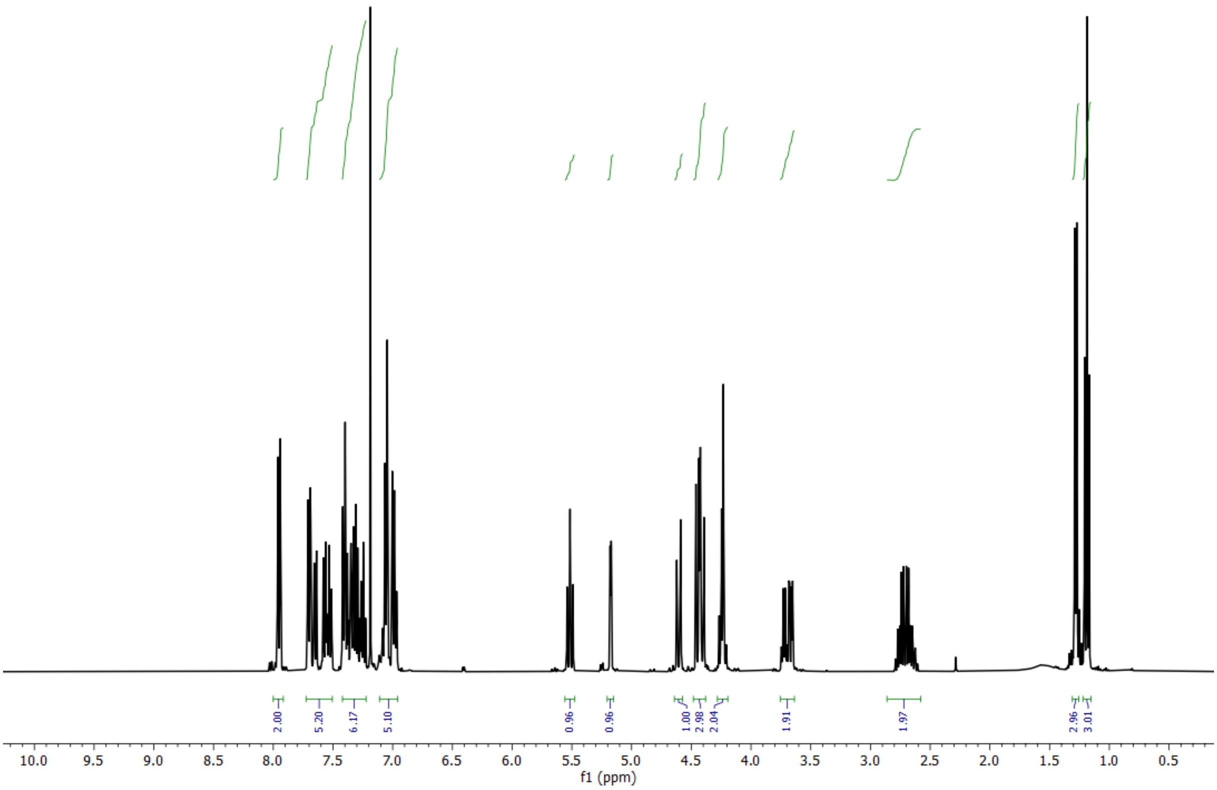

^{13}C NMR of BB3 (101 MHz, $CDCl_3$)

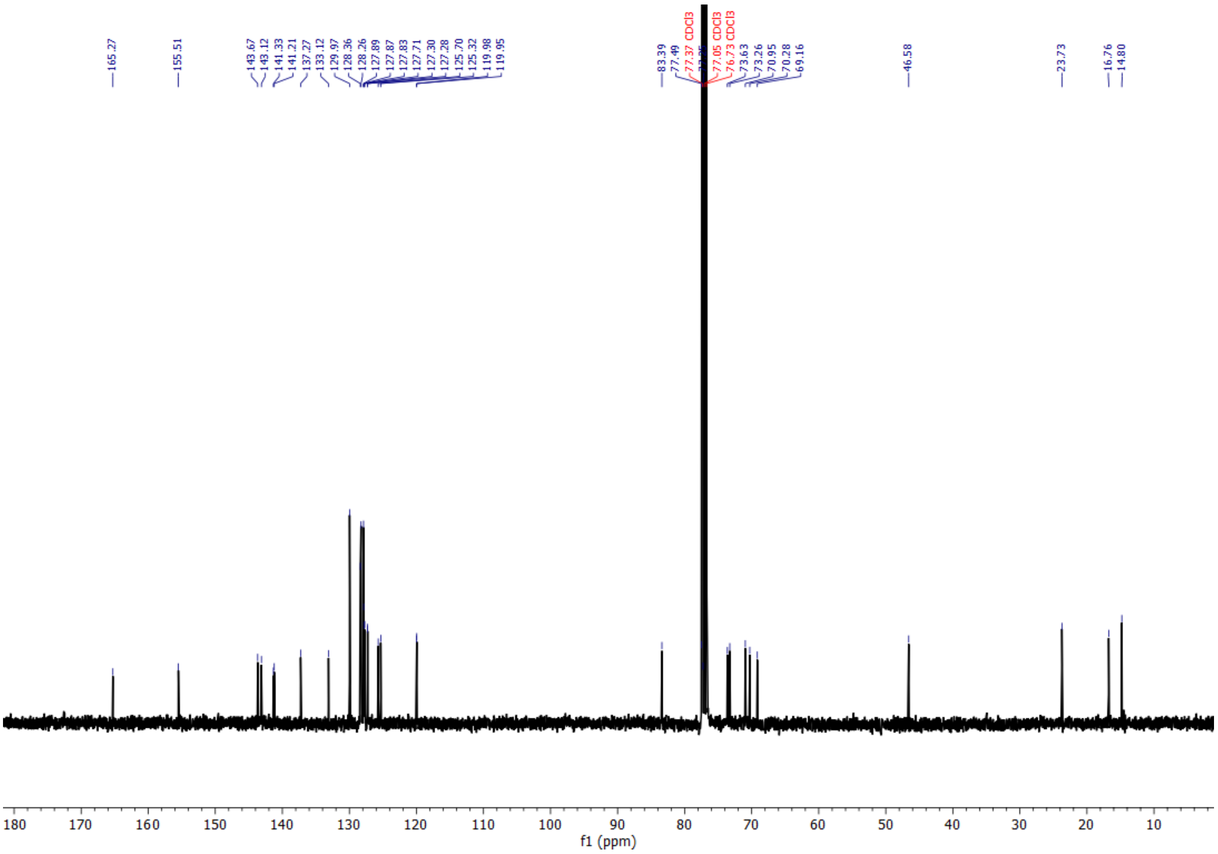

COSY of BB3 ($CDCl_3$)

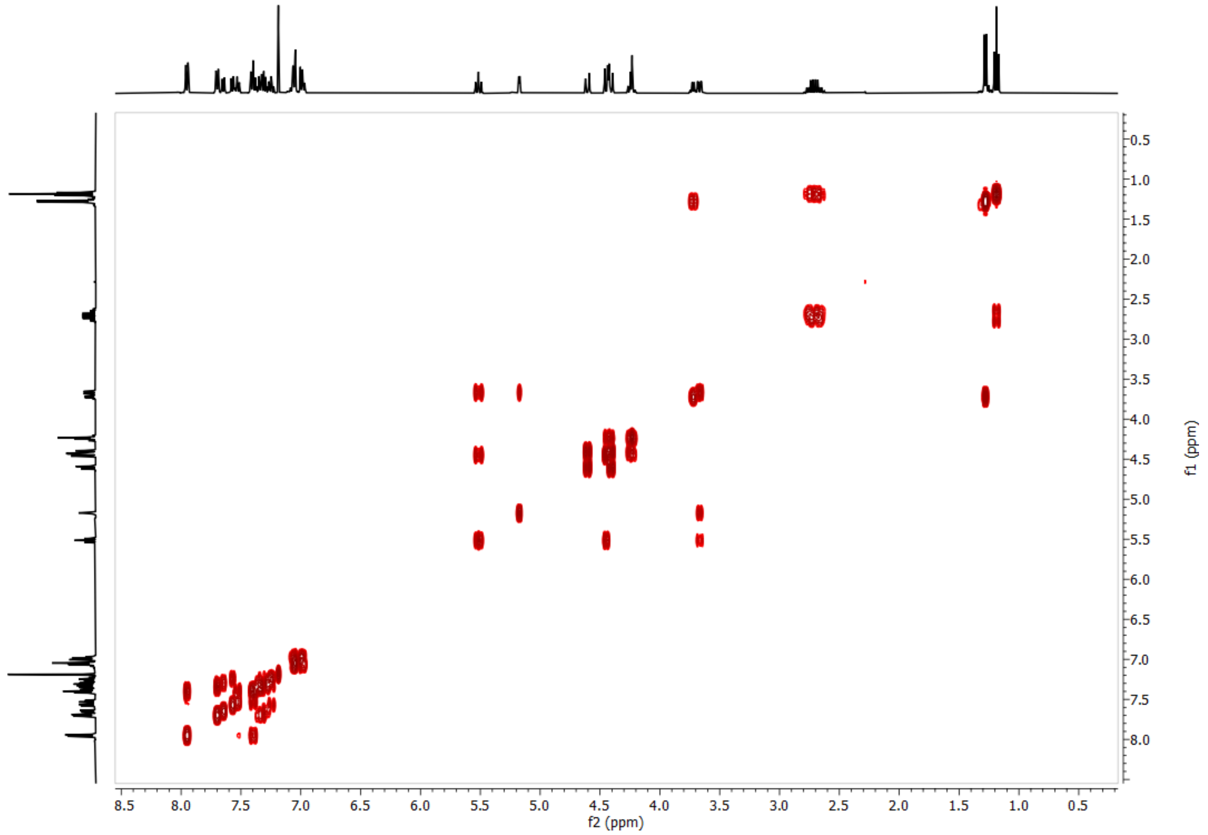

HSQC NMR of BB3 ($CDCl_3$)

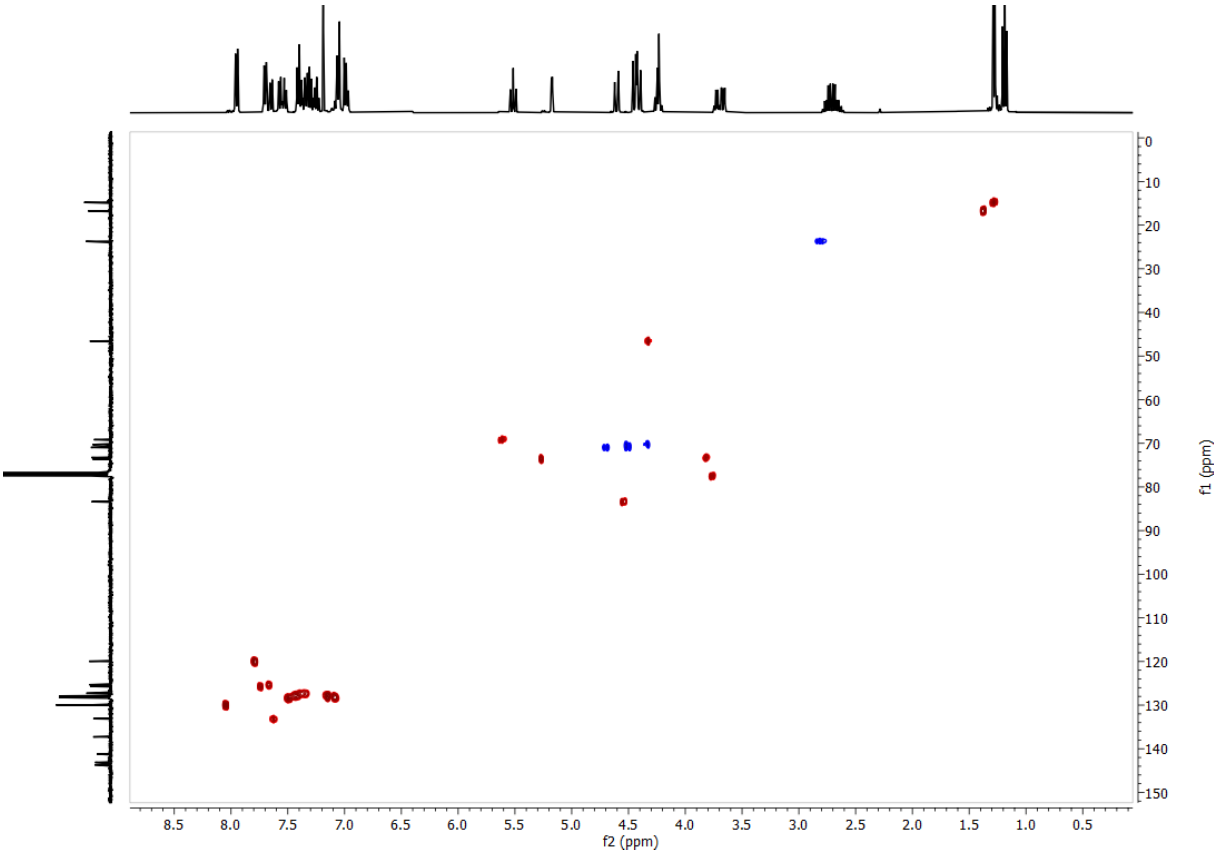

4 Automated glycan assembly

4.1 General materials and methods

The automated syntheses were performed on a home-built synthesizer developed at the Max Planck Institute of Colloids and Interfaces.[62] All solvents used were HPLC-grade. The solvents used for the building blocks, activator, TMSOTf and capping solutions were taken from an anhydrous solvent system (J.C. Meyer). The building blocks were co-evaporated three times with toluene and dried for 1 h on high vacuum before use. Oven-heated, argon-flushed flasks were used to prepare all moisture-sensitive solutions. Activator, capping, deprotection, acidic wash and building block solutions were freshly prepared and kept under argon during the automation run. All yields of products obtained by AGA were calculated on the basis of resin loading. Resin loading was determined following previously established procedures.[11]

4.2 Preparation of stock solutions

- **Building block solution**: Between 0.06 and 0.10 mmol of building block (depending on the BB, see Module C1 and C2) was dissolved in DCM (1 mL).
- **NIS/TfOH activator solution**: 1.35 g (6.0 mmol) of recrystallized NIS was dissolved in 40 mL of a 2:1 v/v mixture of anhydrous DCM and anhydrous dioxane. Then triflic acid (55 µL, 0.6 mmol) was added. The solution is kept at 0 °C (ice bath) for the duration of the automation run.
- **Fmoc deprotection solution**: A solution of 20% v/v piperidine in DMF was prepared.
- **Lev deprotection solution**: Hydrazine acetate (550 mg, 5.97 mmol) was dissolved in pyridine/AcOH/H_2O (40mL, v/v, 32:8:2) and sonicated for 10 min.
- **TMSOTf solution**: TMSOTf (0.45 mL, 2.49 mmol) was added to DCM (40 mL).
- **Capping solution**: A solution of 10% v/v acetic anhydride and 2% v/v methanesulfunic acid in DCM was prepared.

4.3 Modules for automated synthesis

4.3.1 Module A: Resin preparation

All automated syntheses are performed on 0.0125 mmol scale. Resin (**L**) is placed in the reaction vessel and swollen in DCM for 20 min at room temperature prior to the synthesis. During this time, all reagent lines needed for the synthesis are washed and primed. After the swelling, the resin is washed with DMF, THF, and DCM (three times each with 2 mL for 25 s).

4.3.2 Module B: Acidic wash with TMSOTf solution (20 min)

The resin is swollen in 2 mL DCM and the temperature of the reaction vessel adjusted to -20 °C. Upon reaching the low temperature, TMSOTf solution (1 mL) is added dropwise to the reaction vessel. After bubbling for 3 min, the acidic solution is drained and the resin washed with 2 mL DCM for 25 s.

Action	Cycles	Solution	Amount	T (°C)	Incubation time
**Cooling**	-	-	-	-20	(15 min)*
**Deliver**	1	DCM	2 mL	-20	-
**Deliver**	1	TMSOTf solution	1 mL	-20	3 min
**Wash**	1	DCM	2 mL	-20	25 sec

*Time required to reach the desired temperature.

4.3.3 Module C1: Thioglycoside glycosylation (35 min-55 min)

The building block solution (0.10 mmol of BB in 1 mL of DCM per glycosylation) is delivered to the reaction vessel. After the set temperature is reached, the reaction is started by dropwise addition of the NIS/TfOH activator solution (1.0 mL, excess). The glycosylation conditions (T_1, T_2, t_1, and t_2) are building block dependent and are reported in a table below. After completion of the reaction, the solution is drained and the resin was washed with DCM, DCM:dioxane (1:2, 3 mL for 20 s) and DCM (two times, each with 2 mL for 25 s). The temperature of the reaction vessel is increased to 25 °C for the next module. In case of a double cycle (C1*, *Double cycle), module C1 is repeated twice.

Action	Cycles	Solution	Amount	T (°C)	Incubation time
**Cooling**	-	-	-	T_1	-
**Deliver**	1	BB solution	1 mL	T_1	-
**Deliver**	1	NIS/TfOH activator solution	1 mL	T_1	-
**Reaction time (BB dependent)**	1	-	-	T_1 to T_2	t_1 t_2
**Wash**	1	DCM	2 mL	T_2	5 sec
**Wash**	1	DCM : Dioxane (1:2)	2 mL	T_2	20 sec
**Heating**	-	-	-	25	-
**Wash**	2	DCM	2 mL	> 0	25 sec

BB	Equiv.	t_1 (min)	T_1 (°C)	t_2 (min)	T_2 (°C)
**BB1a**	6.5	5	-20	20	0
**BB2**	6.5	5	-20	20	0
**BB2**	6.5	5	-20	20	0
**BB4a**	6.5	5	-20	20	0
**BB4b**	6.5	5	-20	20	0

4.3.4 Module C2: Glycosyl phosphate glycosylation (45 min)

The building block solution (0.06 mmol of BB in 1 mL of DCM per glycosylation) is delivered to the reaction vessel. After the set temperature is reached, the reaction is started by dropwise addition of the TMSOTf solution (1.0 mL, stoichiometric). After completion of the reaction, the solution is drained and the resin washed with DCM (six times, each with 2 mL for 25 s). The temperature of the reaction vessel is increased to 25 °C for the next module. In case of a double cycle (C2*, *Double cycle), module C2 was repeated twice.

Action	Cycles	Solution	Amount	T (°C)	Incubation time
**Cooling**	-	-	-	-30	-
**Deliver**	1	BB solution	1 mL	-30	-
**Deliver**	1	TMSOTf solution	1 mL	-30	-
**Reaction time (BB dependent)**	1	-	-	-30 to -10	5 min 40 min
**Wash**	1	DCM	2 mL	-10	5 sec
**Heating**	-	-	-	25	-
**Wash**	6	DCM	2 mL	> 0	25 sec

BB	Equiv.	t1 (min)	T1 (°C)	t2 (min)	T2 (°C)
**BB1b**	5	5	-30	40	-10

4.3.5 Module D: Capping (30 min)

The resin is washed with DMF (two times with 2 mL for 25 s) and the temperature of the reaction vessel adjusted to 25 °C. A pyridine solution (2 mL, 10%$_{v/v}$ in DMF) is delivered into the reaction vessel. After 1 min, the reaction solution is drained and the resin washed with DCM (three times with 3 mL for 25 s). Capping solution (4 mL) is delivered into the reaction vessel. After 20 min, the reaction solution is drained and the resin washed with DCM (three times with 3 mL for 25 s).

Action	Cycles	Solution	Amount	T (°C)	Incubation time
**Heating**	-	-	-	25	(5 min)*
**Wash**	2	DMF	2 mL	25	25 sec
**Deliver**	1	10% Pyridine in DMF	2 mL	25	1 min
**Wash**	3	DCM	2 mL	25	25 sec
**Deliver**	1	Capping Solution	4 mL	25	20 min
**Wash**	3	DCM	2 mL	25	25 sec

*Time required to reach the desired temperature.

4.3.6 Module E1: Fmoc deprotection (9 min)

The resin is washed with DMF (three times with 2 mL for 25 s) and the temperature of the reaction vessel adjusted to 25 °C. Fmoc deprotection solution (2mL) is delivered to the reaction vessel and kept under Ar bubbling. After 5 min, the reaction solution is drained and the resin washed with DMF (three times with 3 mL for 25 s) and DCM (five times each with 2 mL for 25 s). The temperature of the reaction vessel is decreased to -20 °C for the next module.

Action	Cycles	Solution	Amount	T (°C)	Incubation time
**Wash**	3	DMF	2 mL	25	25 sec
**Deliver**	1	Fmoc depr. solution	2 mL	25	5 min
**Wash**	1	DMF	2 mL		
**Cooling**	-	-	-	-20	-
**Wash**	3	DMF	2 mL	< 25	25 sec
**Wash**	5	DCM	2 mL	< 25	25 sec

4.3.7 Module E2: Lev deprotection (90 min)

The resin is washed with DCM (three times with 2 mL for 25 s). DCM (1.3 mL) is delivered to the reaction vessel and the temperature of the reaction vessel is adjusted to 25 °C. Lev deprotection solution (2mL) is delivered to the reaction vessel, kept under pulsed Ar bubbling for 30 min. This procedure is repeated twice. The reaction solution is drained and the resin washed with DMF (three times with 3 mL for 25 s) and DCM (five times each with 2 mL for 25 s).

Action	Cycles	Solution	Amount	T (°C)	Incubation time
**Wash**	3	DMF	2 mL	25	25 sec
**Deliver**	2	Lev depr. solution	2 mL	25	30 min
**Wash**	1	DMF	2 mL	-	-
**Cooling**	-	-	-	-20	-
**Wash**	3	DMF	2 mL	< 25	25 sec
**Wash**	5	DCM	2 mL	< 25	25 sec

4.4 Post-AGA manipulations

4.4.1 Module F: On-resin methanolysis

The resin is suspended in THF (4 mL). MeONa in MeOH (0.5 M, 0.4 mL) is added and the suspension is gently shaken at room temperature. After micro-cleavage (see *Module G2*) indicates the complete removal of benzoyl groups, the resin is repeatedly washed with MeOH (3 x 2 mL) and DCM (3 x 2 mL).

4.4.2 Module G1: Cleavage from solid support

The oligosaccharides are cleaved from the solid support using a continuous-flow photoreactor as described previously.[12]

4.4.3 Module G2: Micro-cleavage from solid support

Trace amount of resin (around 20 beads) is dispersed in DCM (0.1 mL) and irradiated with a UV lamp (6 W, 356 nm) for 10 minutes. ACN (10 µL) is then added to the resin and the resulting solution analyzed by MALDI.

4.4.4 Module H: Hydrogenolysis

The crude compound obtained from *Module G1* is dissolved in 2 mL of EtOAc:*t*BuOH/H_2O (2:1:1). $Pd(OH)_2/C$ (10-20%$_w$, moistened with water) is added and the reaction stirred in a pressurized reactor under H_2 pressure (4 bar). The reaction progress is monitored to avoid undesired side products formation (*i.e.* degradation of reducing end).[13] Upon completion, the reaction is filtered (PTFE 0.45 µm 25 mm syringe filter, Fisher scientific) and washed with EtOAc, H_2O, and ACN (4 mL each). The filtrates are concentrated *in vacuo.*

4.4.5 Module I: Purification

The final compounds are analyzed using analytical reversed phase HPLC (Agilent 1200 Series, Method A1). The purification of the crudes is conducted using reversed phase HPLC (Agilent 1200 Series, Method A2).

- **Method A1**: (Hypercarb column, ThermoFisher scientific, 150 x 4.6 mm, 3 µm) flow rate of 0.7 mL/min with H_2O (0.1% formic acid) and ACN as eluents [isocratic (5 min), linear gradient to 30% ACN (30 min), linear gradient to 100% ACN (5 min), isocratic 100% ACN (5 min)].
- **Method A1 (Prep)**: (Hypercarb column, ThermoFisher scientific, 150 x 10 mm, 5 µm) flow rate of 0.7 mL/min with H_2O (0.1% formic acid) and ACN as eluents [isocratic (5 min), linear gradient to 30% ACN (30 min), linear gradient to 100% ACN (5 min), isocratic 100% ACN (5 min)].
- **Method A2**: (Hypercarb column, ThermoFisher scientific, 150 x 10 mm, 5 µm), flow rate of 3 mL /min with H_2O (0.1% formic acid) and ACN as eluents [isocratic (5 min), linear gradient to 60% ACN (60 min), linear gradient to 100% ACN (5 min), isocratic 100% ACN (5 min)].
- **Method A2 (Prep)**: (Hypercarb column, ThermoFisher scientific, 150 x 10 mm, 5 µm), flow rate of 3 mL /min with H_2O (0.1% formic acid) and ACN as eluents [isocratic (5 min), linear gradient to 60% ACN (60 min), linear gradient to 100% ACN (5 min), isocratic 100% ACN (5 min)].
- **Method A3**: (Synergi Hydro RP18 column, Phenomenex, 250 x 4.6 mm), flow rate of 1 mL /min with H_2O (0.1% formic acid) and ACN as eluents [isocratic (5 min), linear gradient to 10% ACN (50 min), linear gradient to 100% ACN (5 min), isocratic 100% ACN (5 min)].
- **Method A3 (Prep)**: (Synergi Hydro RP18 column, Phenomenex, 250 x 4.6 mm), flow rate of 4 mL /min with H_2O (0.1% formic acid) and ACN as eluents [isocratic (5 min), linear gradient to 10% ACN (50 min), linear gradient to 100% ACN (5 min), isocratic 100% ACN (5 min)].
- **Method A4**: (Synergi Hydro RP18 column, Phenomenex, 250 x 4.6 mm), flow rate of 1 mL /min with H_2O (0.1% formic acid) and ACN as eluents [isocratic (5 min), linear gradient to 15% ACN (40 min), linear gradient to 100% ACN (5 min), isocratic 100% ACN (5 min)].
- **Method A4 (Prep)**: (Synergi Hydro RP18 column, Phenomenex, 250 x 10 mm), flow rate of 4 mL /min with H_2O (0.1% formic acid) and ACN as eluents [isocratic (5 min), linear gradient to 15% ACN (40 min), linear gradient to 100% ACN (5 min), isocratic 100% ACN (5 min)].

Following final purification, all deprotected products are lyophilized on a Christ Alpha 2-4 LD plus freeze dryer prior to characterization.

4.5 Oligosaccharide synthesis

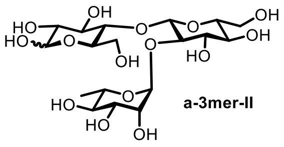

a-3mer-II

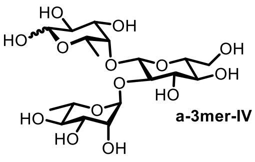

a-3mer-IV

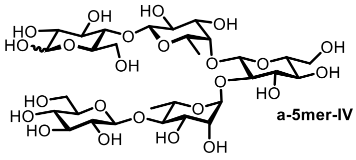

a-5mer-IV

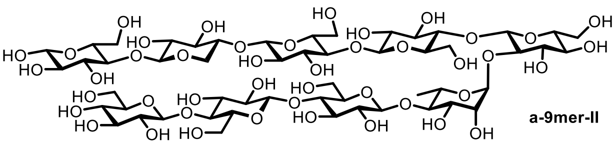

a-9mer-II

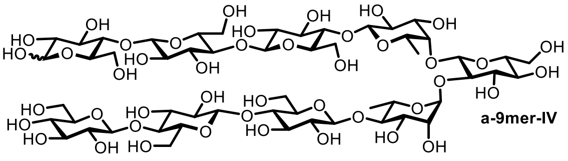

a-9mer-IV

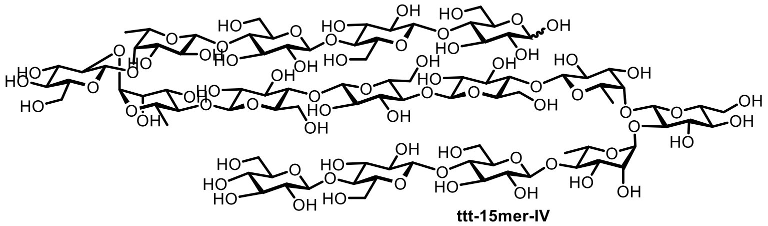

ttt-15mer-IV

4.5.1 a-3mer-II

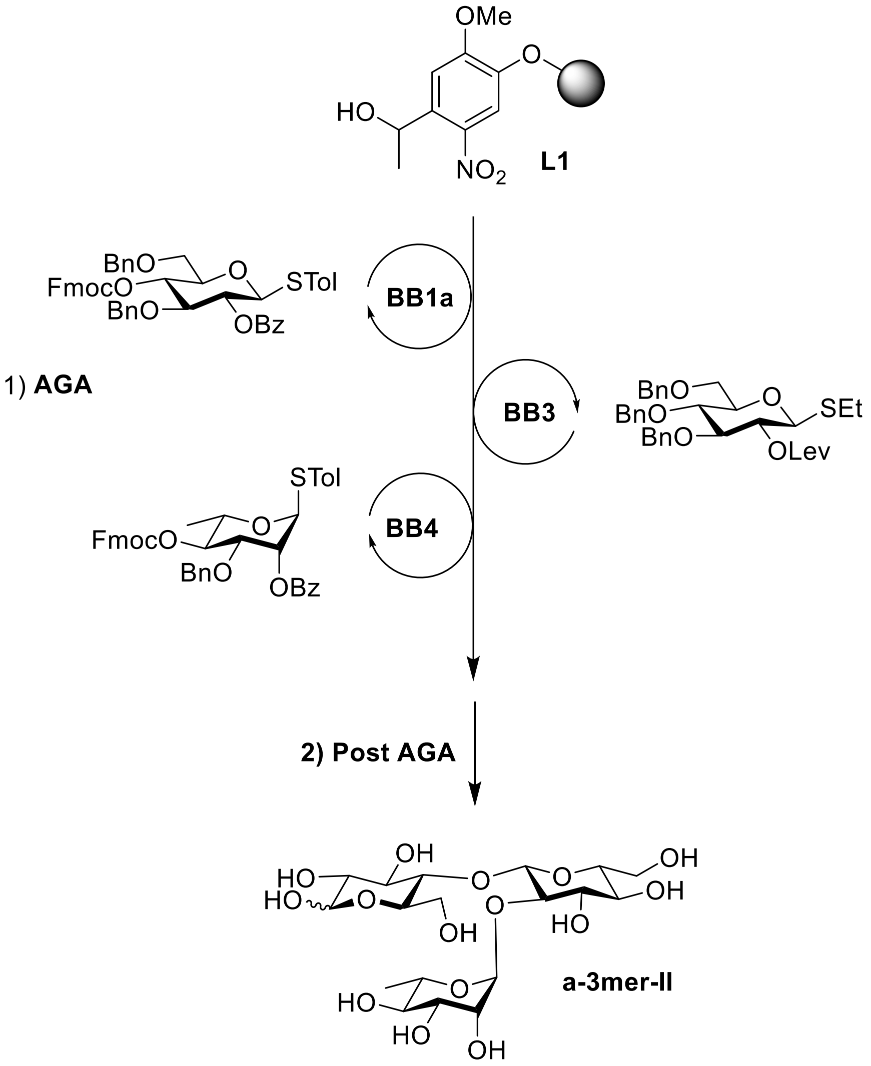

Step	BB	Modules	Notes
AGA	-	**A**	**L1** swelling
	**BB1a**	**B**, **C1**, **D**, **E1**	**C1**: (**BB1a**, -20 °C for 5 min, 0 °C for 20 min)
	**BB3**	**B**, **C1**, **D**, **E2**	**C1**: (**BB3**, -20 °C for 5 min, 0 °C for 20 min)
	**BB4**	**B**, **C1**	**C1**: (**BB4b**, -20 °C for 5 min, 0 °C for 20 min)
**Post-AGA**	-	**F**, **G1**, **H2**, **I**	**F**: (3 h); **H2**: (7 h); **I**: (Method A1: 25.1 min)

Automated synthesis, global deprotection, and purification afforded **a-3mer-II** as a white solid (1.4 mg, 23% overall yield).

^{1}H NMR (400 MHz, D_2O) δ 5.09 (d, *J* = 3.8 Hz, 0.4 H, H-1α Glc), 5.05 (d, *J* = 1.8 Hz, 0.6H, H-1 Rha), 5.03 (d, *J* = 1.8 Hz, 0.4H, H-1 Rha), 4.52 (d, *J* = 7.9 Hz, 0.6H, H-1β Glc), 4.43 (d, *J* = 7.8 Hz, 1H, H-1 Glc), 3.93 (t, *J* = 1.8 Hz, 1H, H-2 Rha), 3.87 – 3.72 (m, 3H), 3.72 – 3.38 (m, 7.4H), 3.38 – 3.23 (m, 4H), 3.14 (dd, *J* = 9.4, 7.9 Hz, 0.6H, H-2β Glc), 1.14 (dd, *J* = 6.4, 1.2 Hz, 3H, H-6 Rha).

^{13}**C NMR** (101 MHz, D_2O) δ 100.98, 100.82, 100.09, 95.75, 91.75, 78.14, 77.76, 76.43, 76.24, 75.94, 75.06, 74.04, 73.97, 71.68, 71.29, 71.06, 70.17, 70.09, 69.90, 69.42, 68.97, 60.54, 59.87, 16.36, 16.31.

ESI-HRMS m/z 511.1614 $[M+Na]^+$ ($C_{18}H_{32}O_{15}Na$ requires 511.1633).

RP-HPLC of a-3mer-II (ELSD trace, Method A1, tR = 25.1 min)

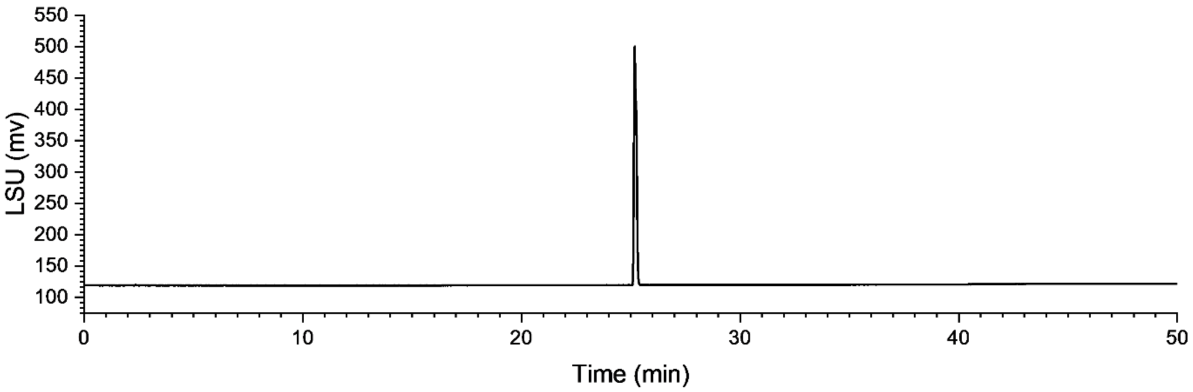

^{1}H NMR of a-3mer-II (400 MHz, D_2O)

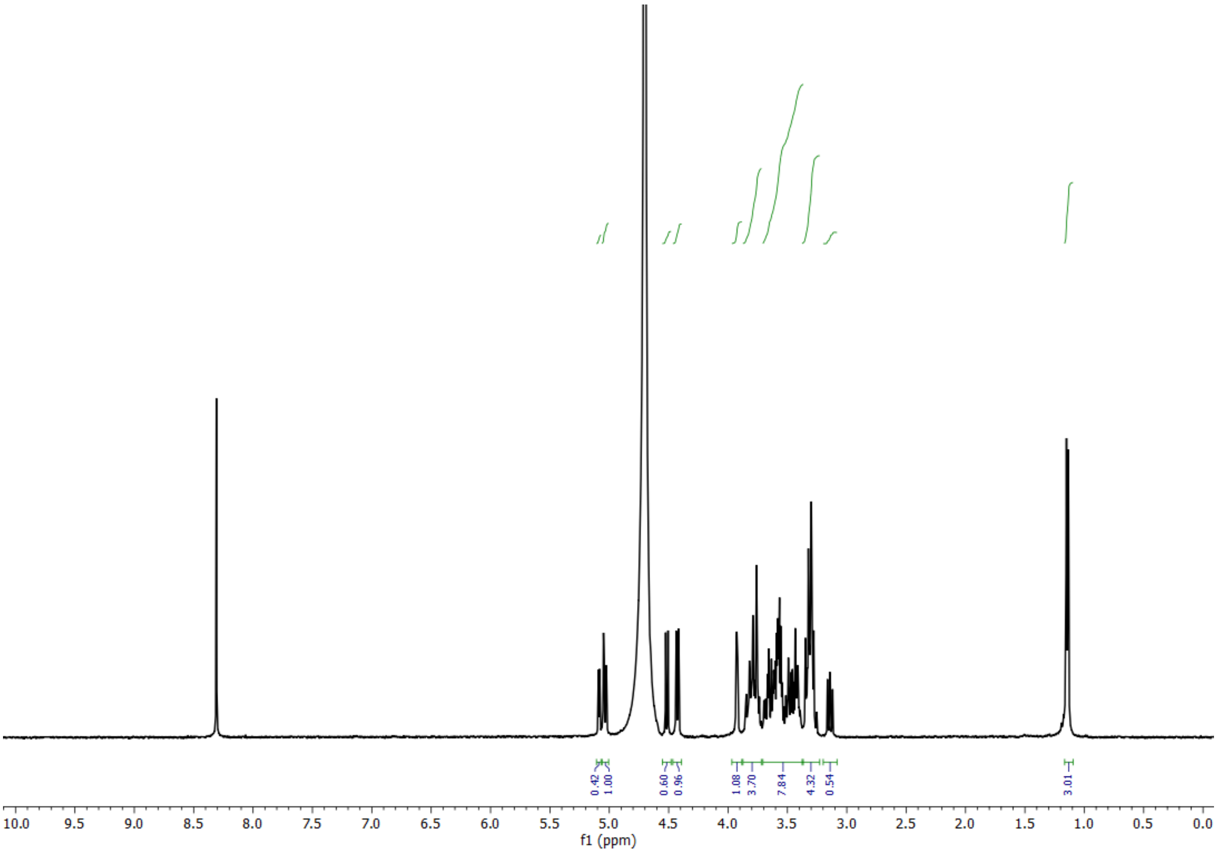

^{13}C NMR of a-3mer-II (101 MHz, D_2O)

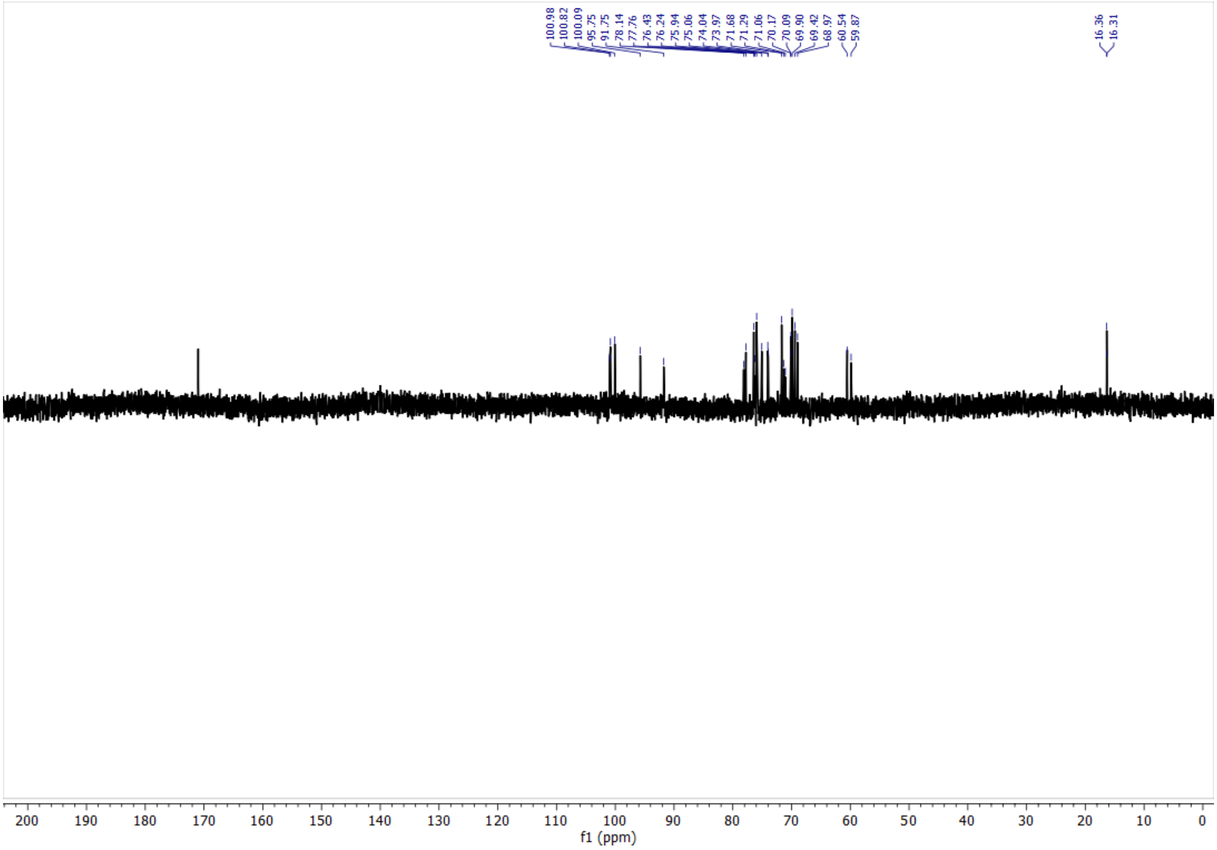

COSY NMR of a-3mer-II (D_2O)

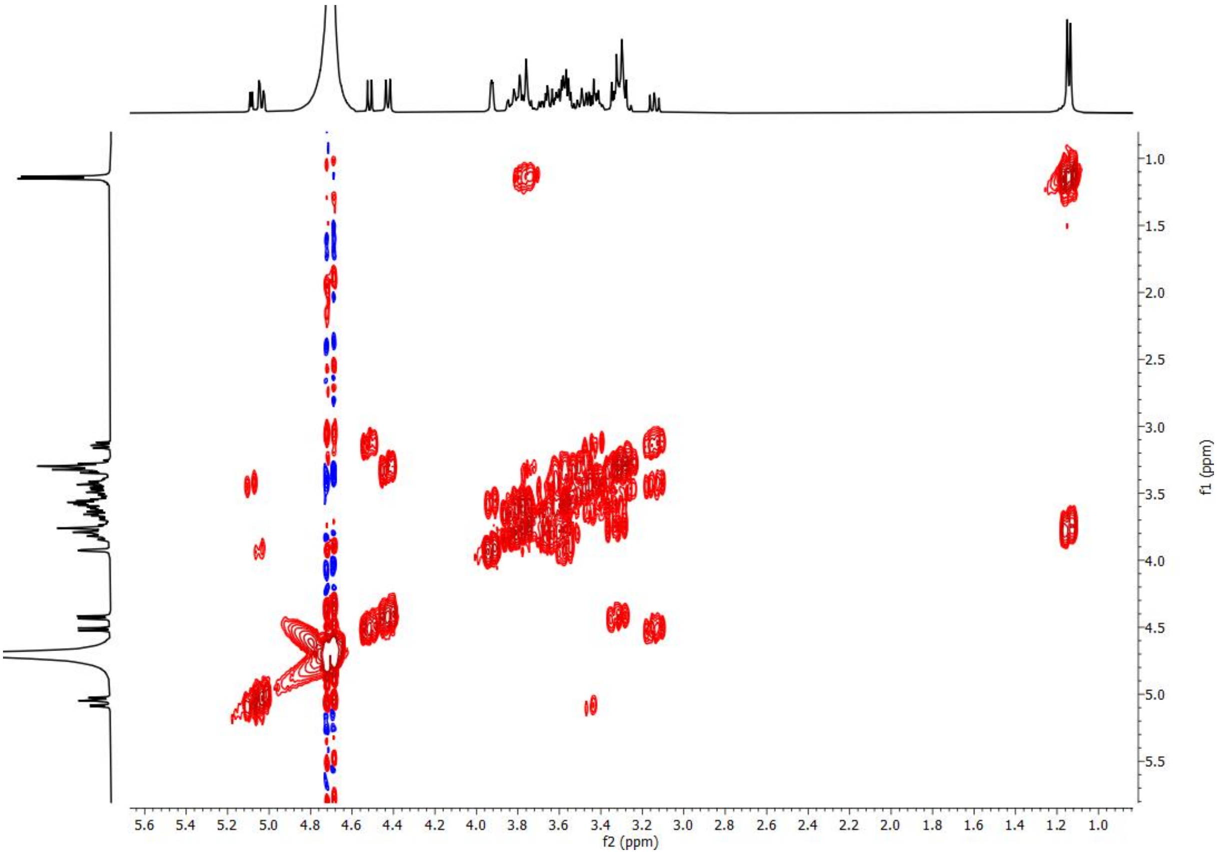

HSQC NMR of a-3mer-II (D_2O)

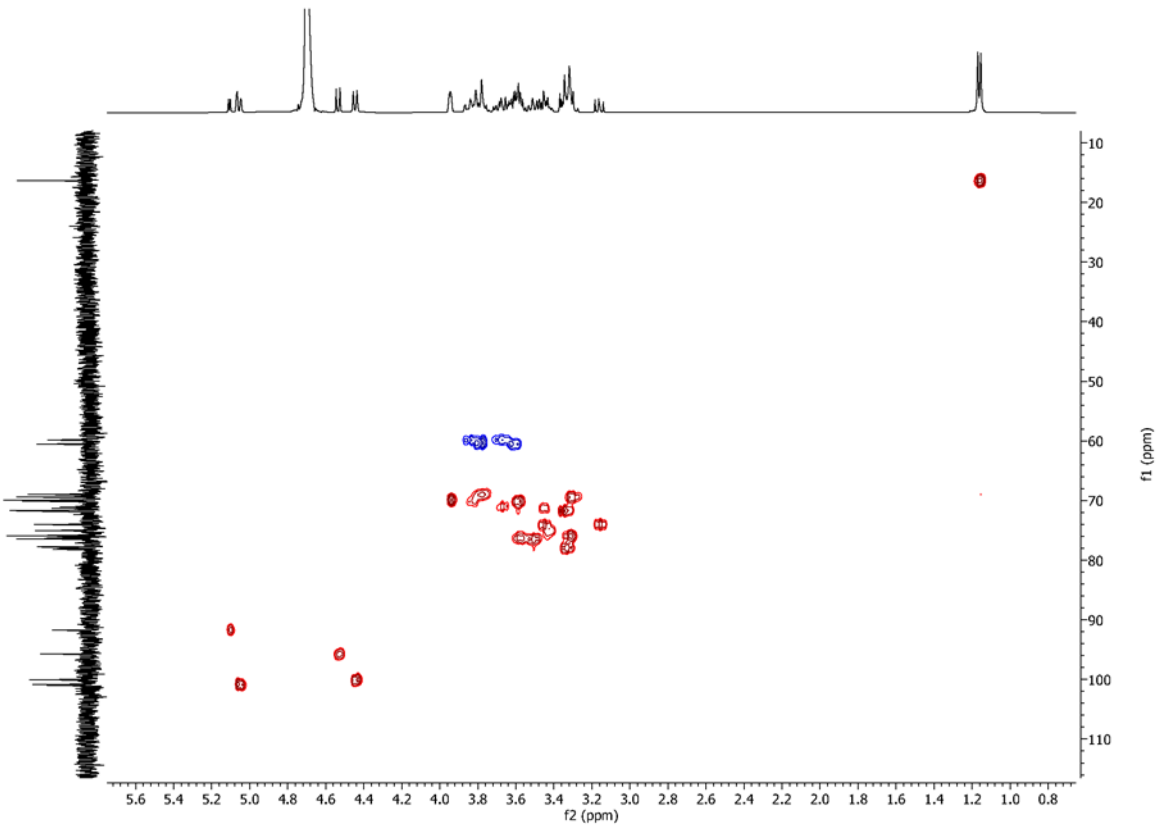

4.5.2 a-3mer-IV

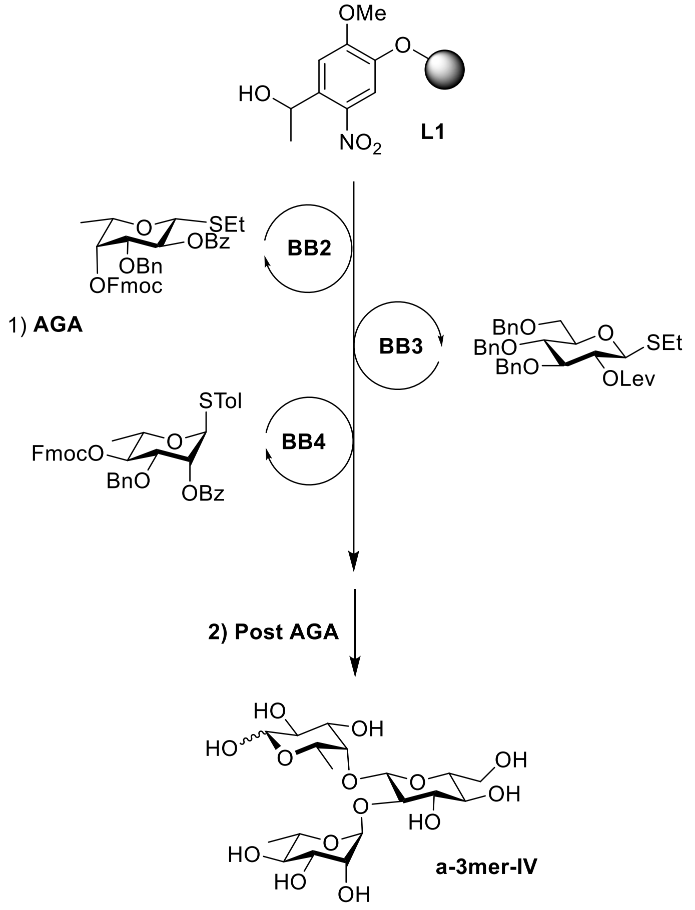

Step	BB	Modules	Notes
AGA	-	**A**	**L1** swelling
	**BB2**	**B**, **C1***, **D**, **E1**	**C1**: (**BB2**, -20 °C for 5 min, 0 °C for 20 min) *Double cycle
	**BB3**	**B**, **C1**, **D**, **E2**	**C1**: (**BB3**, -20 °C for 5 min, 0 °C for 20 min)
	**BB4**	**B**, **C1**	**C1**: (**BB4b**, -20 °C for 5 min, 0 °C for 20 min)
Post-AGA	-	**F**, **G1**, **H2**, **I**	**F**: (3 h); **H2**: (7 h); **I**: (Method A1: 19.2, 19.4 min)

Automated synthesis, global deprotection, and purification afforded **a-3mer-IV** as a white solid (1.7 mg, 29% overall yield).

^{1}H NMR (400 MHz, D_2O) δ 5.22 (d, J = 1.6 Hz, 0.6H, H-1 Rha), 5.16 (d, J = 1.7 Hz, 0.4H, H-1 Rha), 5.06 (d, J = 3.8 Hz, 0.4H, H-1α Fuc), 4.46 (t, J = 7.3 Hz, 1H, H-1 Glc), 4.42 (d, J = 7.9 Hz, 0.6H, H-1β Fuc), 4.10 (hept, J = 6.1 Hz, 1.4H, H-5 Rha and H-5α Fuc), 3.97 – 3.85 (m, 2H), 3.83 – 3.63 (m, 3H), 3.62 – 3.39 (m, 4H), 3.37 – 3.19 (m, 3.6H), 1.28 – 1.07 (m, 6H).

^{13}C NMR (101 MHz, D_2O) δ 170.97, 100.94, 100.67, 100.45, 99.78, 96.20, 92.17, 78.16, 77.64, 77.00, 76.85, 75.92, 75.86, 75.36, 72.42, 72.11, 72.02, 71.78, 70.74, 70.11, 70.05, 70.00, 69.65, 69.55, 68.74, 68.52, 68.45, 68.11, 66.38, 60.69, 60.61, 16.91, 16.78, 15.77, 15.73.

ESI-HRMS m/z 495.1691 $[M+Na]^+$ ($C_{18}H_{32}O_{14}Na$ requires 495.1684).

RP-HPLC of a-3mer-IV (ELSD trace, Method A1, tR = 19.2, 19.4 min)

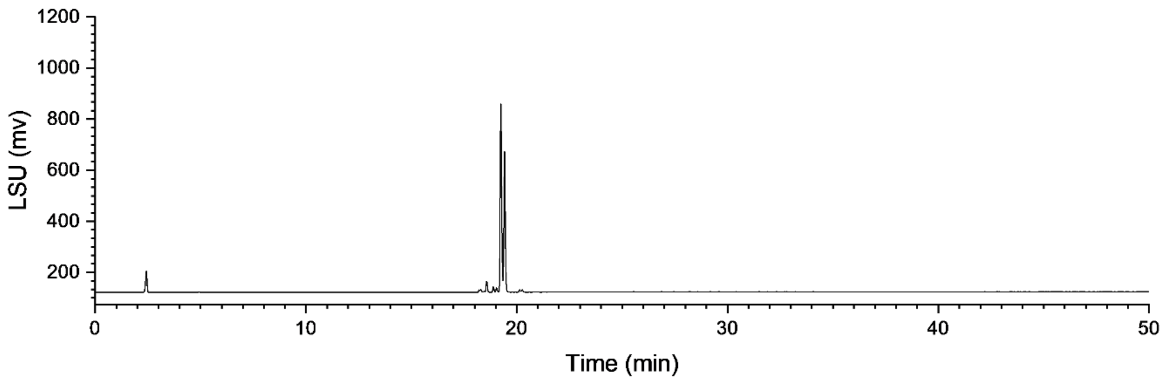

^{1}H NMR of a-3mer-IV (400 MHz, D_2O)

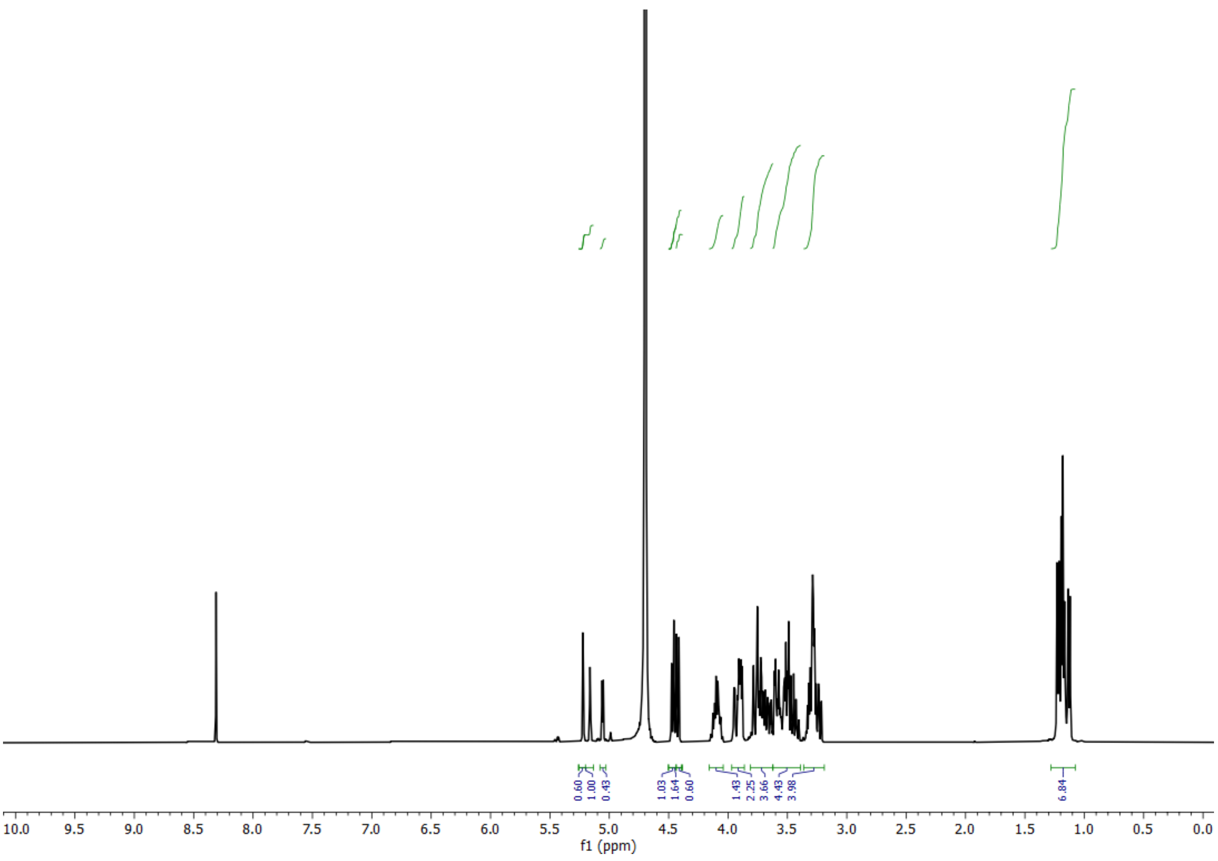

^{13}C NMR of a-3mer-IV (101 MHz, D_2O)

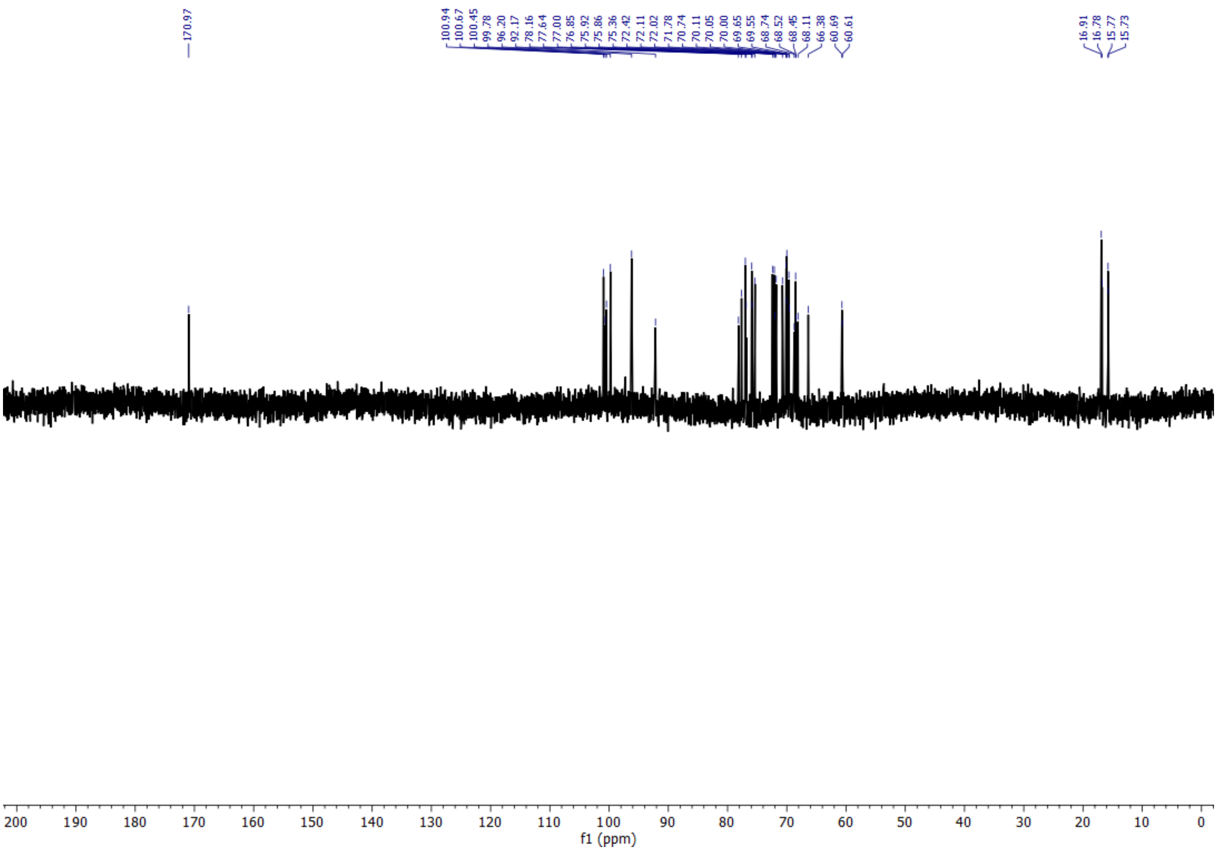

COSY NMR of a-3mer-IV(D_2O)

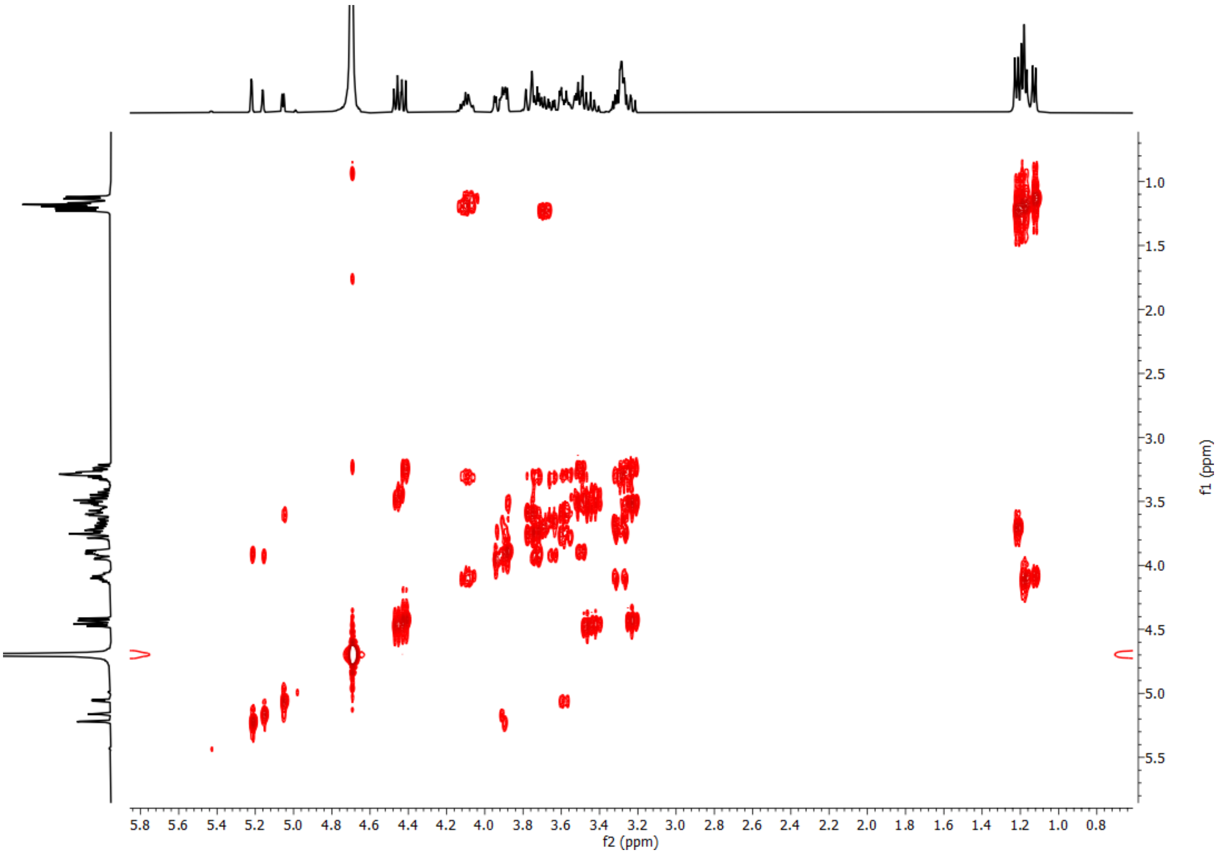

HSQC NMR of a-3mer-IV (D_2O)

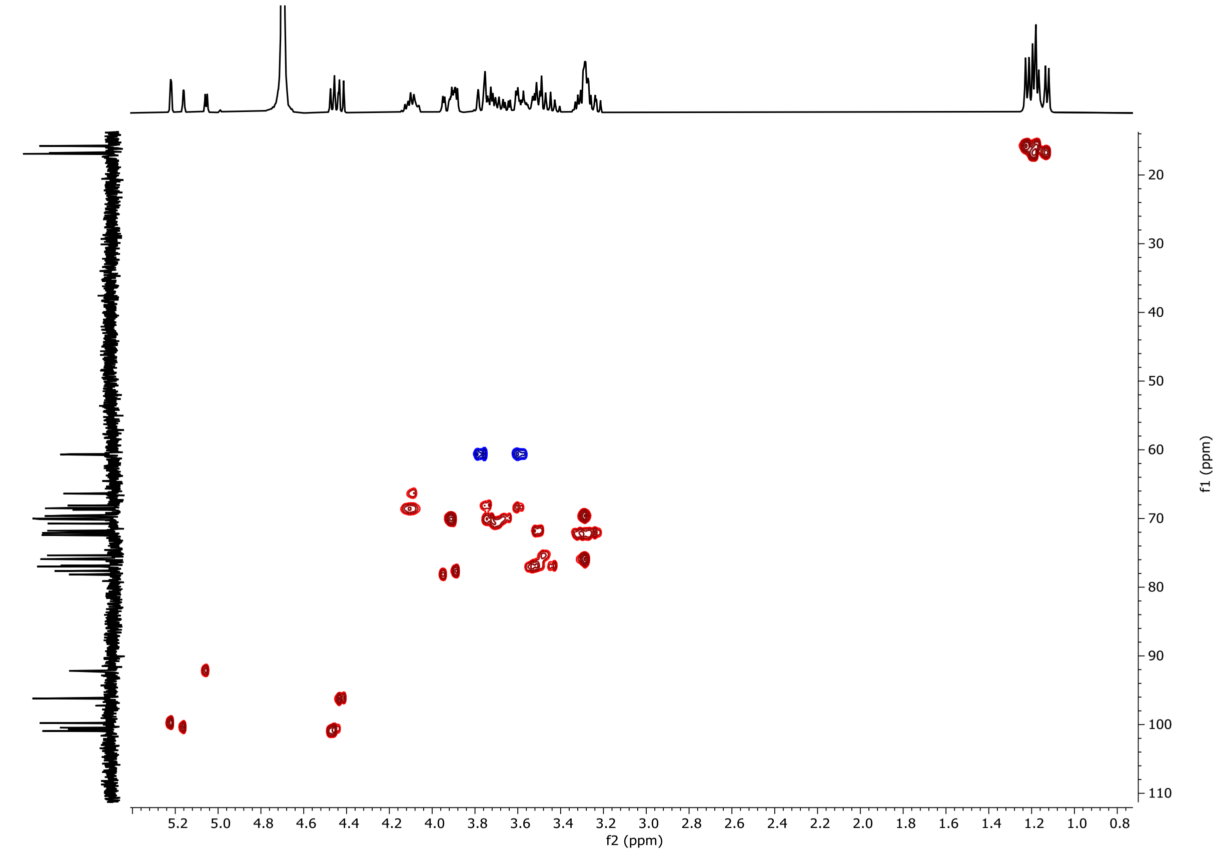

4.5.3 a-5mer-IV

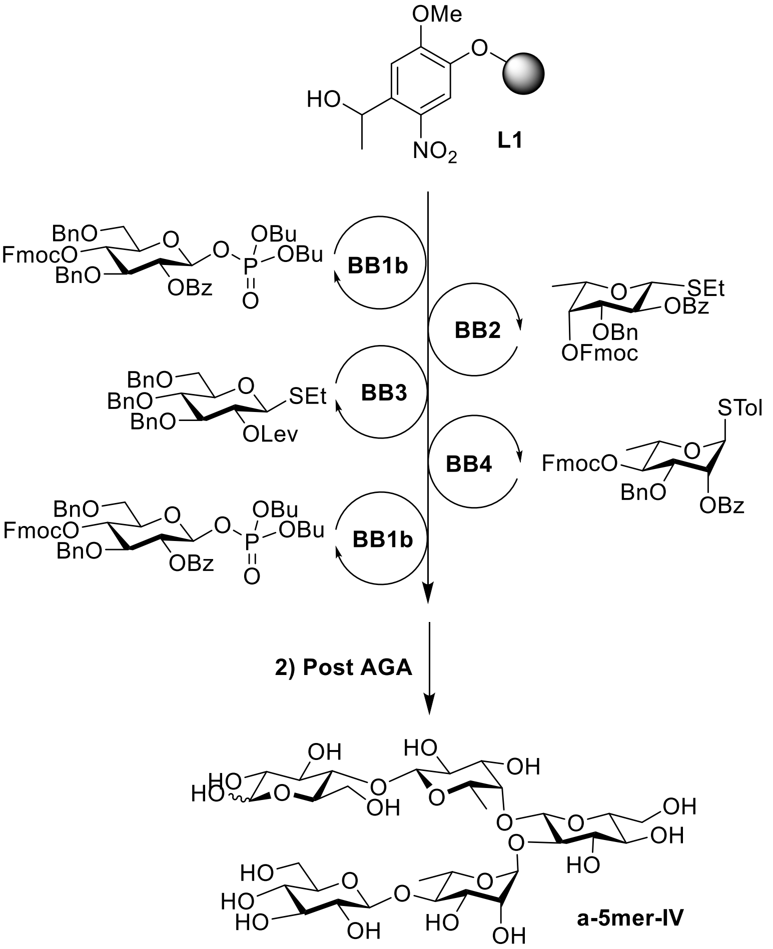

Step	BB	Modules	Notes
AGA	-	**A**	**L1** swelling
	**BB1b**	**B, C2, D, E1**	**C1:** (**BB1b**, -30 °C for 5 min, -10 °C for 40 min)
	**BB2**	**B**, **C1***, **D**, **E1**	**C1**: (**BB2**, -20 °C for 5 min, 0 °C for 20 min) *Double cycle
	**BB3**	**B**, **C1**, **D**, **E2**	**C1**: (**BB3**, -20 °C for 5 min, 0 °C for 20 min)
	**BB4**	**B**, **C1**, **D**, **E1**	**C1**: (**BB4a**, -20 °C for 5 min, 0 °C for 20 min)
	**BB1b**	**B, C2, D, E1**	**C1:** (**BB1b**, -30 °C for 5 min, -10 °C for 40 min)
**Post-AGA**	-	**F**, **G1**, **H2**, **I**	**F**: (3 h); **H2**: (7 h); **I**: (Method A2: **27.6, 27.8 min**)

Automated synthesis, global deprotection, and purification afforded **a-5mer-IV** as a white solid (2.5 mg, 25% overall yield).

^{1}H NMR (400 MHz, D_2O) δ 5.14 (s, 1H, H-1 Rha), 5.09 (d, J = 3.7 Hz, 0.4H, H-1α Glc), 4.60 – 4.53 (m, 1H, H-1 Glc), 4.51 (d, J = 8.0 Hz, 0.6H, H-1β Glc), 4.45 (d, J = 7.7 Hz, 1H, H-1 Fuc), 4.43 (d, J = 7.9 Hz, 1H, H-1 Glc), 4.08 – 3.95 (m, 2H), 3.94 – 3.64 (m, 9H), 3.64 – 3.18 (m, 14.4H), 3.14 (dd, J = 9.3, 8.0 Hz, 0.6H, H-2β Glc), 1.26 – 1.16 (m, 6H).

¹³C NMR (101 MHz, D_2O) δ 103.90, 103.73, 103.62, 100.88, 100.75, 95.67, 91.85, 81.03, 80.87, 78.70, 78.51, 78.09, 78.00, 77.71, 77.62, 76.55, 75.87, 75.80, 75.03, 74.23, 73.77, 73.70, 72.05, 71.76, 71.52, 71.09, 70.73, 70.29, 69.96, 69.75, 69.65, 69.53, 68.10, 60.83, 60.70, 60.52, 17.51, 17.48, 15.56.

ESI-HRMS m/z 819,2792 $[M+Na]^+$ ($C_{30}H_{52}O_{24}Na$ requires 819,2741).

RP-HPLC of a-5mer-IV (ELSD trace, Method A2, tR = 27.6, 27.8 min)

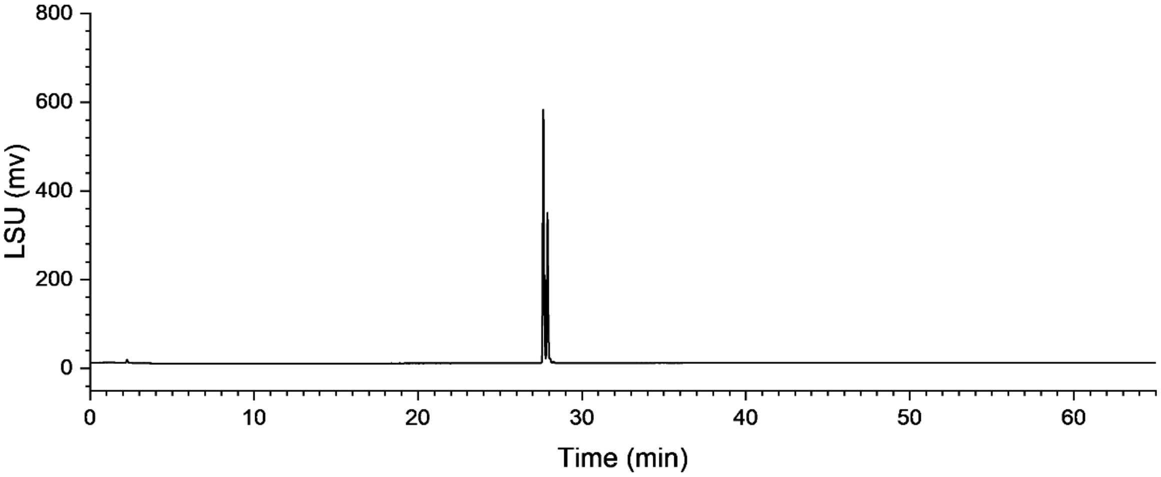

¹H NMR of a-5mer-IV (400 MHz, D_2O)

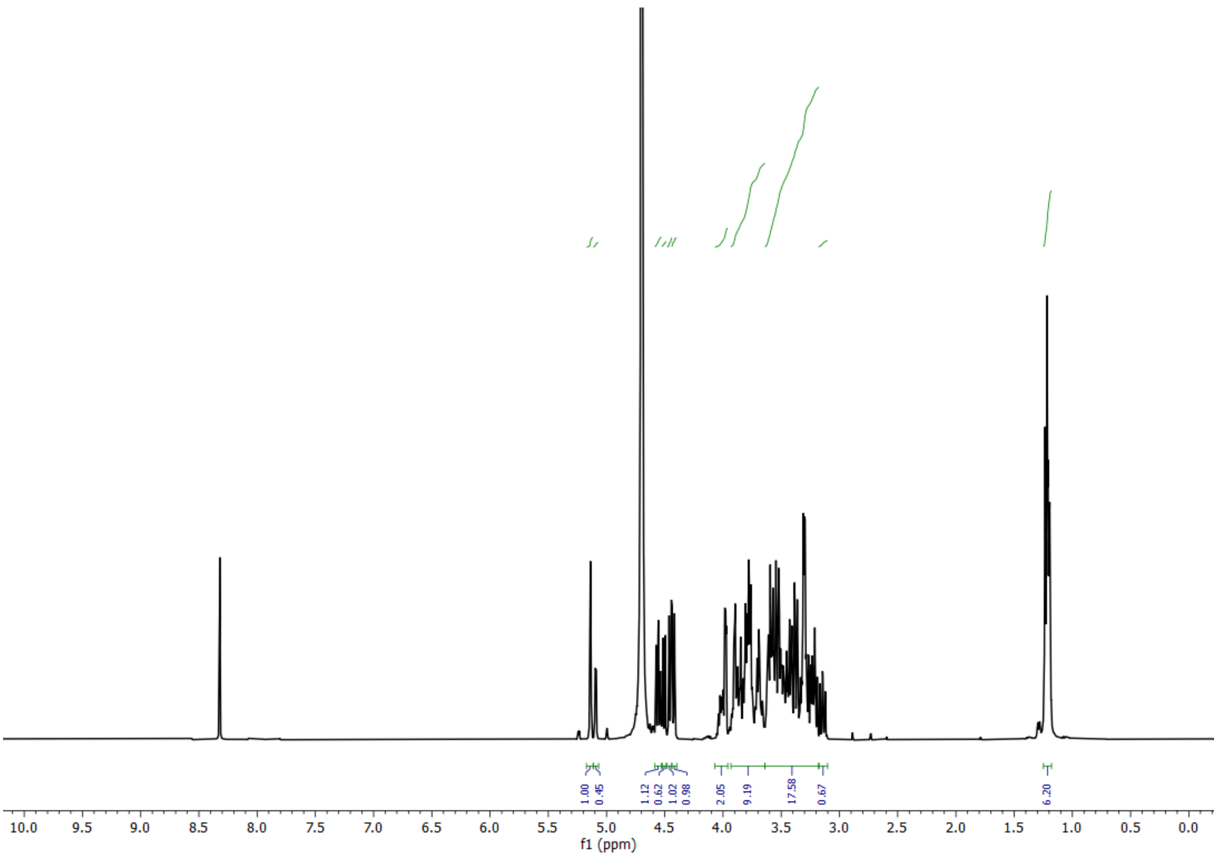

^{13}C NMR of a-5mer-IV (101 MHz, D_2O)

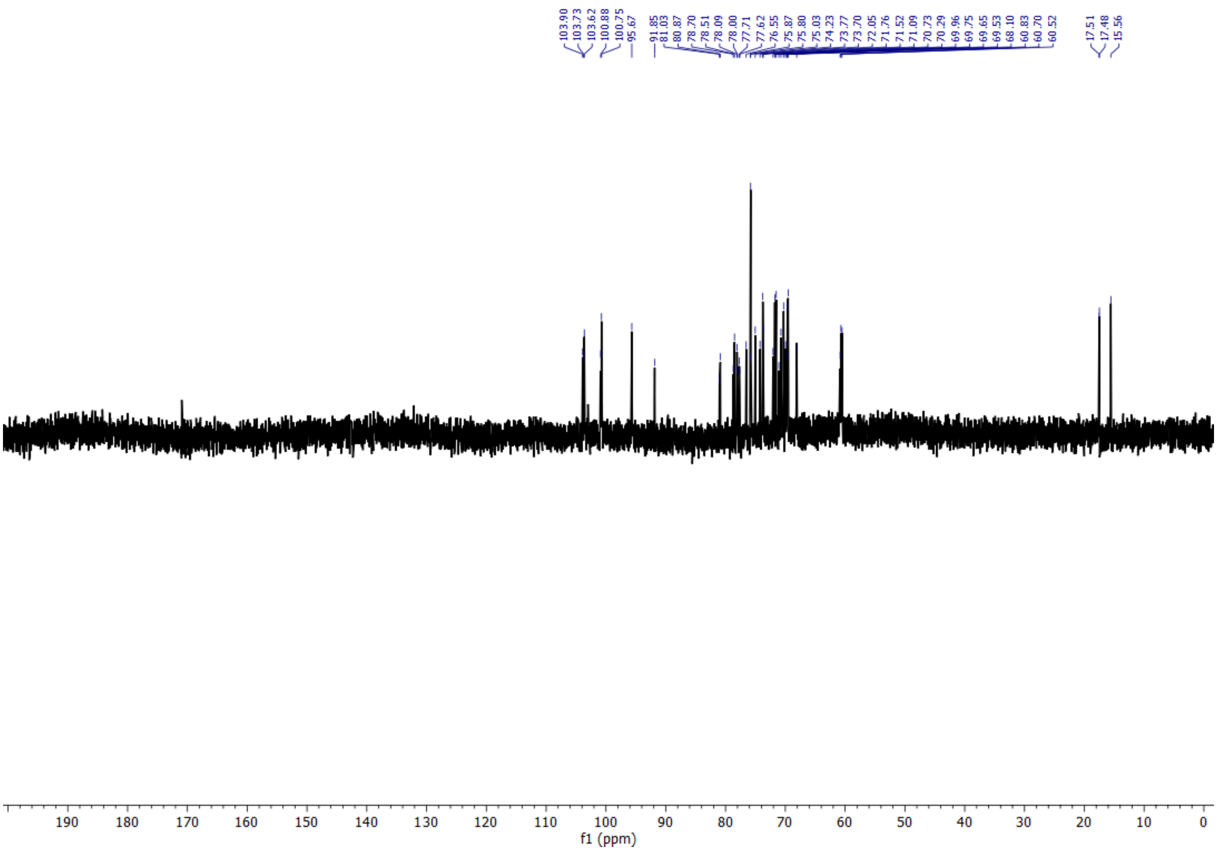

COSY NMR of a-5mer-IV (D_2O)

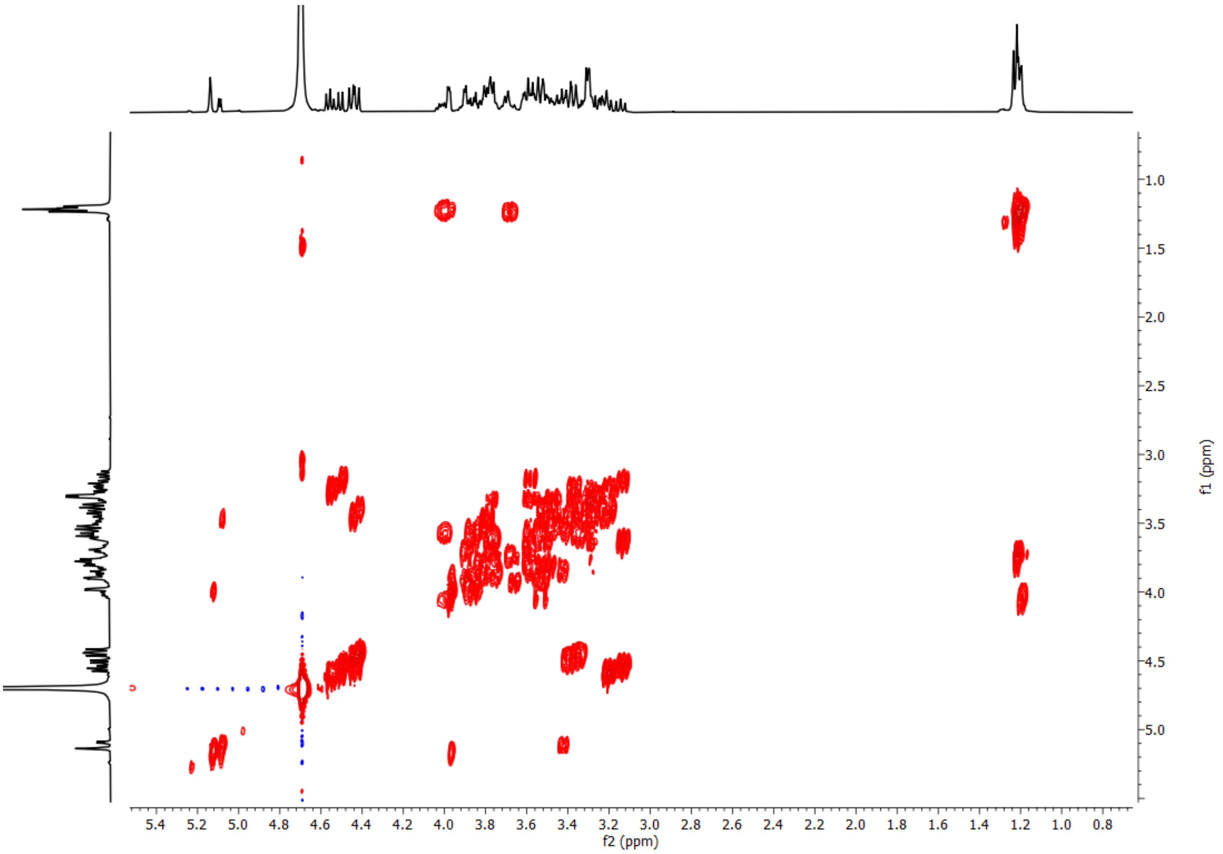

HSQC NMR of a-5mer-IV (D_2O)

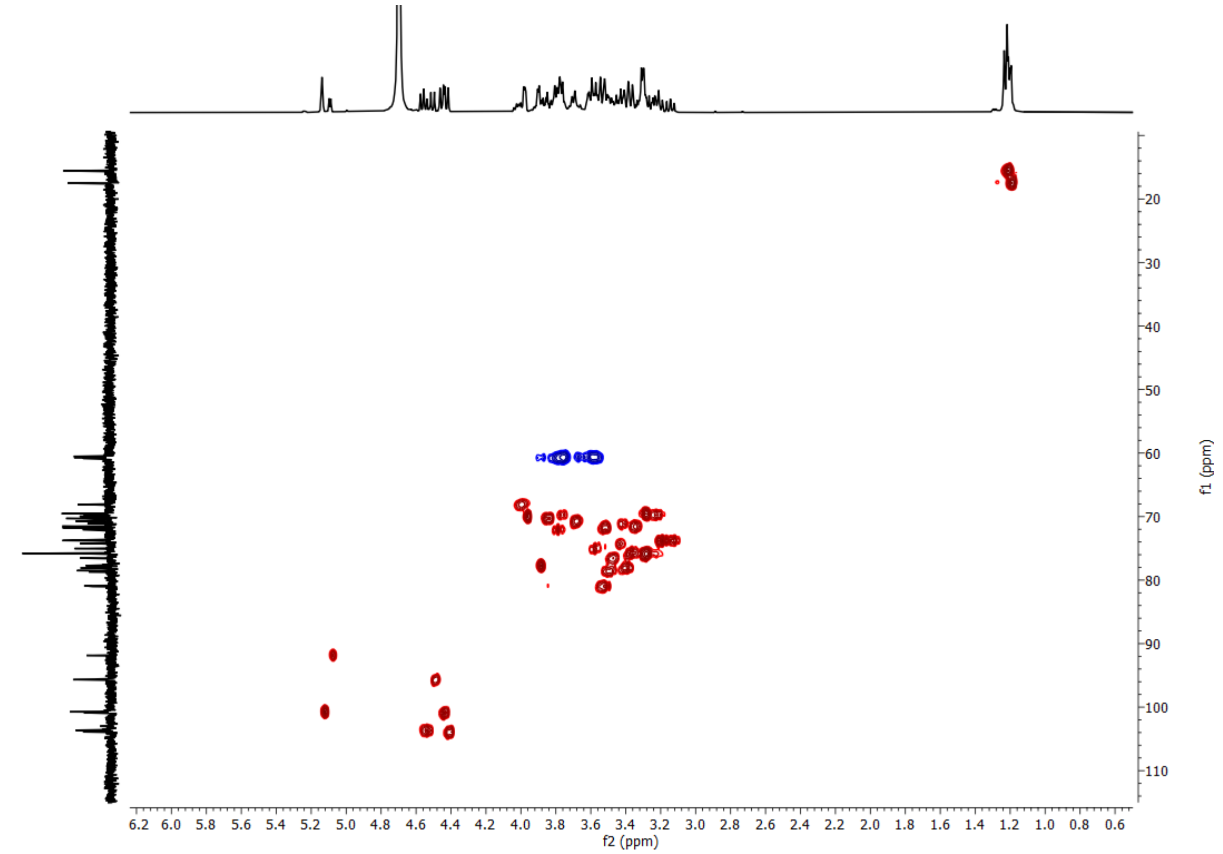

4.5.4 a-9mer-II

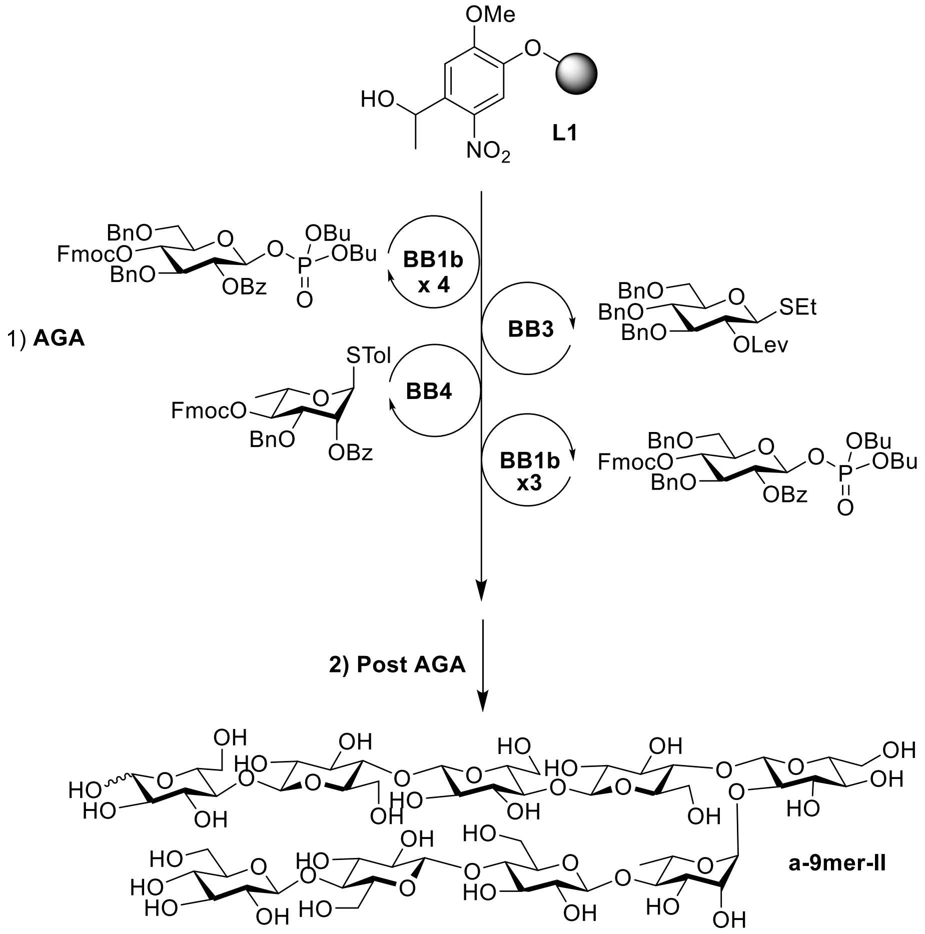

Step	BB	Modules	Notes
AGA	-	**A**	**L1** swelling
	(**BB1b**)x4	(**B, C2, D, E1**)x4	**C1:** (**BB1b**, -30 °C for 5 min, -10 °C for 40 min)
	**BB3**	**B**, **C1***, **D**, **E2**	**C1**: (**BB3**, -20 °C for 5 min, 0 °C for 20 min) *Double cycle
	**BB4**	**B**, **C1, D**, **E1**	**C1**: (**BB4a**, -20 °C for 5 min, 0 °C for 20 min)
	**BB1b**	**B, C2*, D, E1**	**C1:** (**BB1b**, -30 °C for 5 min, -10 °C for 40 min) *Double cycle
	(**BB1b**)x2	(**B, C2, D, E1**)x2	**C1:** (**BB1b**, -30 °C for 5 min, -10 °C for 40 min)
**Post-AGA**	-	**F**, **G1**, **H2**, **I**	**F**: (3 h); **H2**: (7 h); **I**: (Method A3: 28.02 min)

Automated synthesis, global deprotection, and purification afforded **a-9mer-II** as a white solid (3.4 mg, 19% overall yield).

^{1}H NMR (400 MHz, D_2O) δ 5.10 (d, J = 3.8 Hz, 0.4H, H-1α Glc), 5.07 (d, J = 1.8 Hz, 1H, H-1 Rha), 4.63 (d, J = 7.9 Hz, 1H, H-1 Glc), 4.54 (d, J = 8.0 Hz, 0.6H, H-1β Glc), 4.41 (m, 6H), 3.94 (dd, J = 3.4,

1.8 Hz, 1H, H-2 Rha), 3.92 – 3.65 (m, 16H), 3.65 – 3.43 (m, 22H), 3.42 – 3.12 (m, 13H), 1.23 (d, J = 6.3 Hz, 3H).

^{13}C NMR (101 MHz, D_2O) δ 103.17, 102.52, 102.27, 102.28, 100.46, 100.05, 95.6, 80.99, 78.36, 77.94, 77.49, 75.91, 75.60, 74.73, 73.94, 73.82, 73.06, 72.87, 71.16, 70.32, 69.36, 69.90, 67.45, 60.49, 59.80, 55.92, 16.64.

ESI-HRMS m/z 1461.502 $[M+H]^+$ ($C_{54}H_{93}O_{45}$ requires 1461.4984), 1483.487 $[M+Na]^+$ ($C_{54}H_{92}O_{45}Na$ requires 1483.4803).

RP-HPLC of a-9mer-II (ELSD trace, Method A3, tR = 28.02 min)

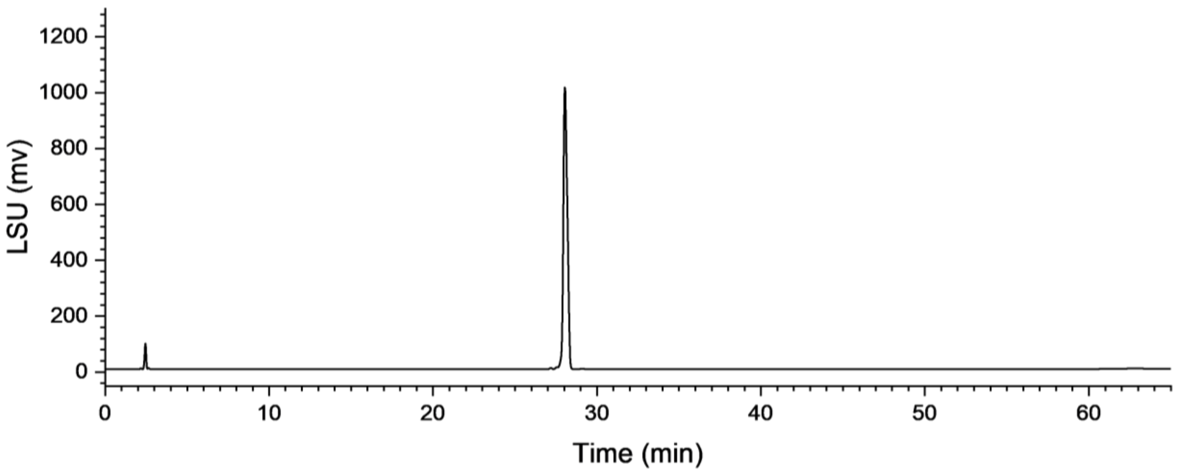

^{1}H NMR of a-9mer-II (400 MHz, D_2O)

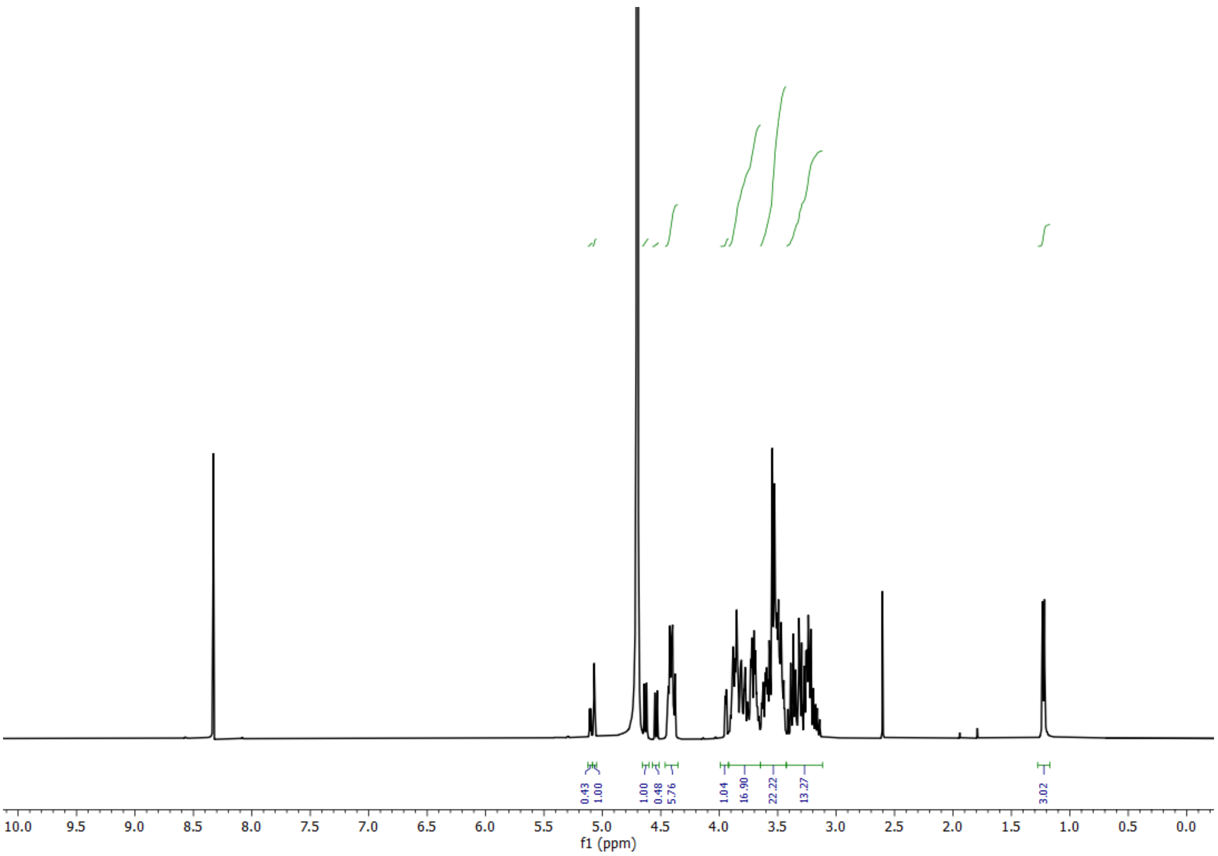

^{13}C NMR of a-9mer-II (101 MHz, D_2O)

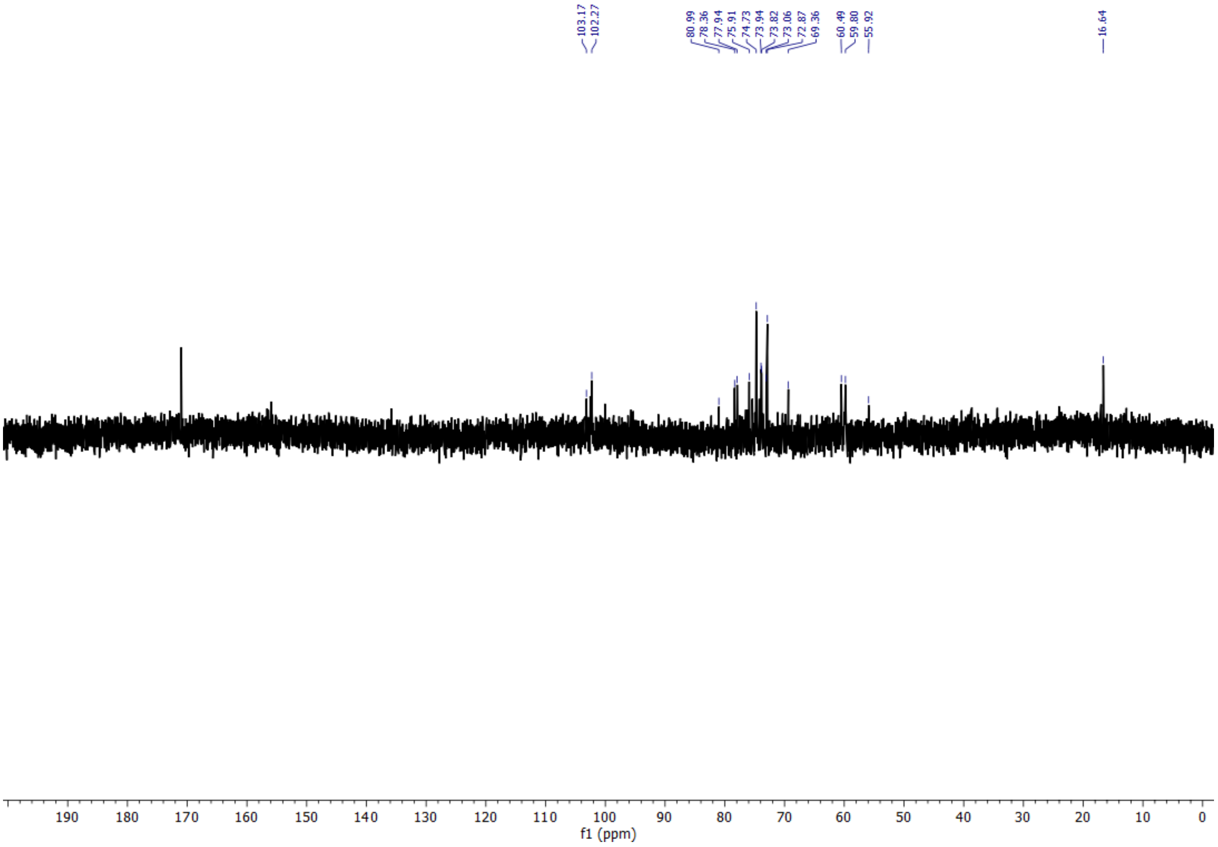

COSY NMR of a-9mer-II (D_2O)

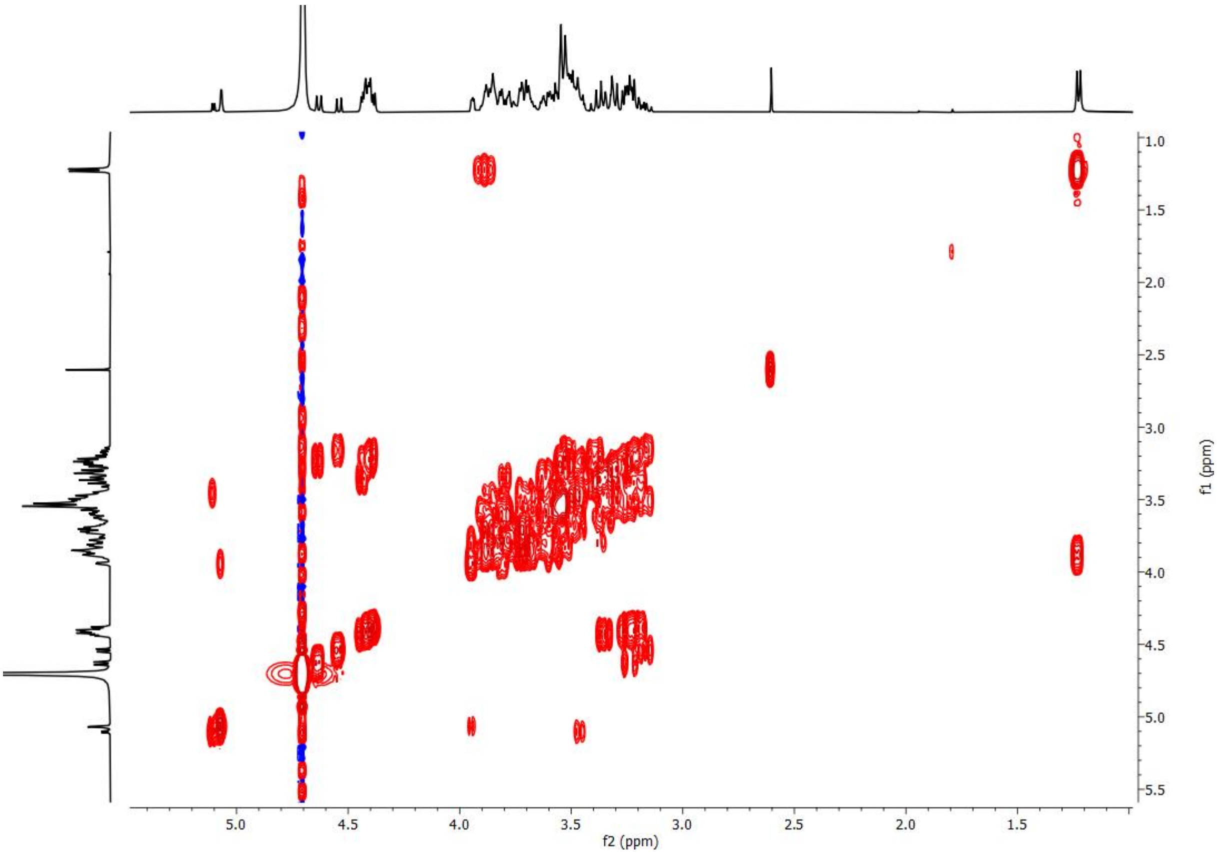

HSQC NMR of a-9mer-II (D_2O)

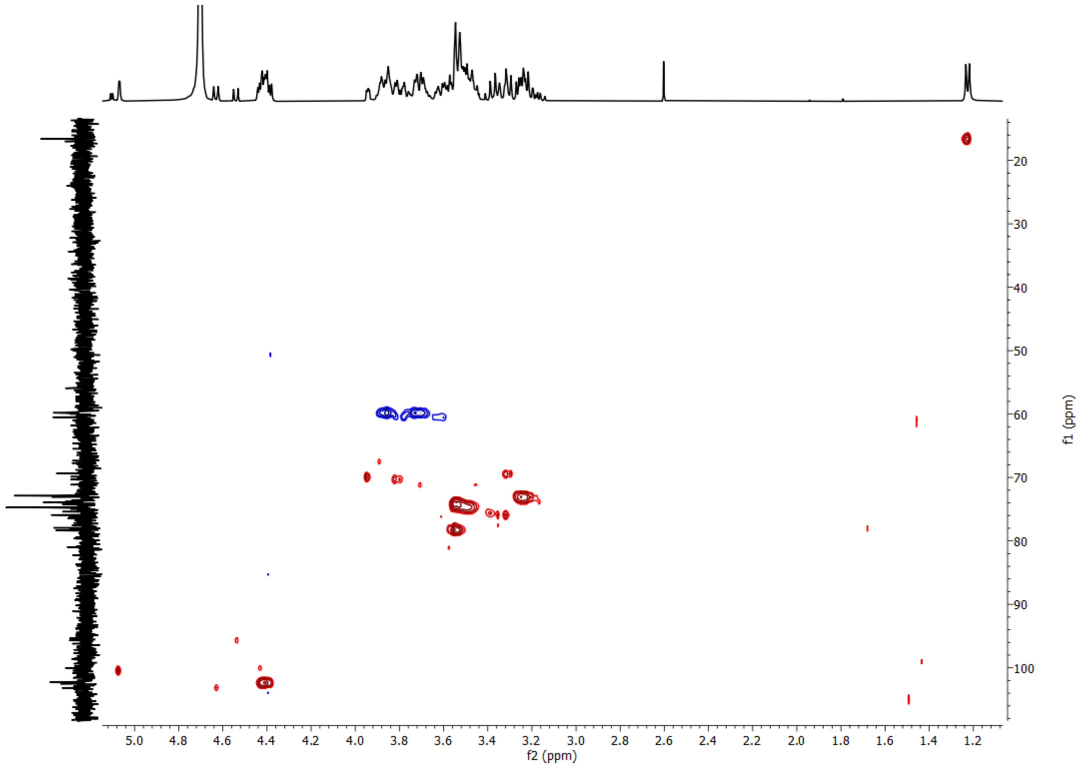

4.5.5 a-9mer-IV

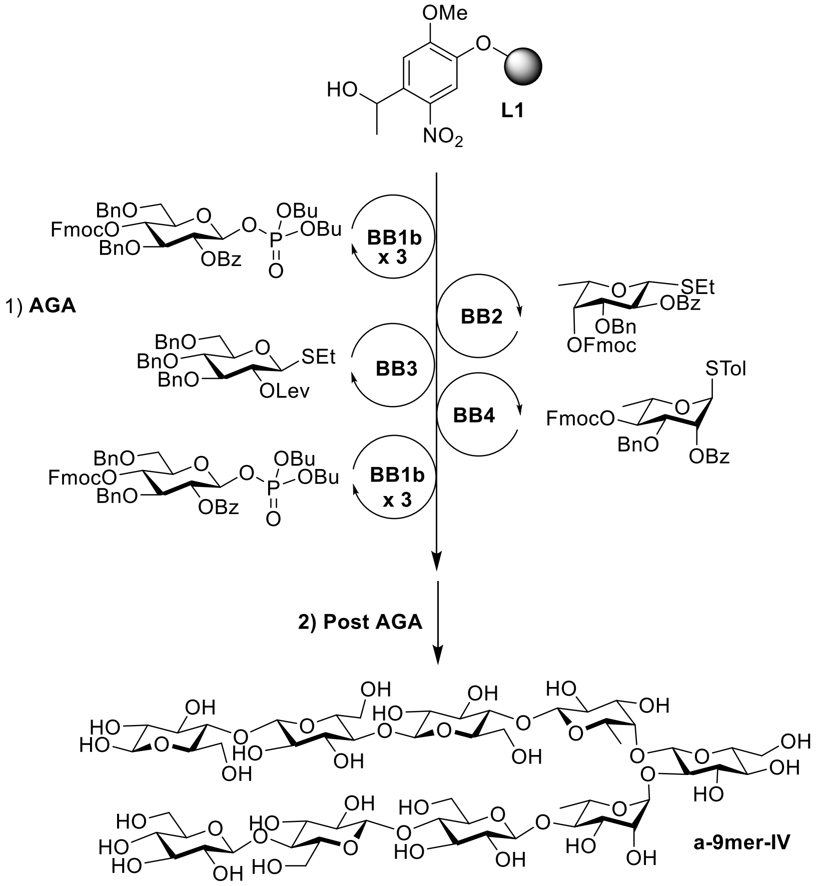

Step	BB	Modules	Notes
AGA	-	**A**	**L1** swelling
	(**BB1b**)x3	(**B, C2, D, E1**)x3	**C1:** (**BB1b**, -30 °C for 5 min, -10 °C for 40 min)
	**BB2**	**B**, **C1***, **D**, **E1**	**C1**: (**BB2**, -20 °C for 5 min, 0 °C for 20 min) *Double cycle
	**BB3**	**B**, **C1***, **D**, **E2**	**C1**: (**BB3**, -20 °C for 5 min, 0 °C for 20 min) *Double cycle
	**BB4**	**B**, **C1**, **D**, **E1**	**C1**: (**BB4a**, -20 °C for 5 min, 0 °C for 20 min)
	**BB1b**	**B, C2*, D, E1**	**C1:** (**BB1b**, -30 °C for 5 min, -10 °C for 40 min) *Double cycle
	(**BB1b**)x2	(**B, C2, D, E1**)x2	**C1:** (**BB1b**, -30 °C for 5 min, -10 °C for 40 min)
**Post-AGA**	-	**F**, **G1**, **H2**, **I**	**F**: (3 h); **H2**: (7 h); **I**: (Method A2: 39.7, 40.2 min)

Automated synthesis, global deprotection, and purification afforded **a-9mer-IV** as a white solid (1.9 mg, 11% overall yield).

^{1}H NMR (600 MHz, D_2O) δ 5.13 (d, J = 1.7 Hz, 1H, H-1 Rha), 5.10 (d, J = 3.8 Hz, 0.4H, H-1α Glc), 4.59 (d, J = 8.0 Hz, 1H, H-1α Glc), 4.54 (d, J = 8.0 Hz, 0.6H, H-1β Glc), 4.48 – 4.35 (m, 6H), 4.07 – 3.95 (m, 2H), 3.92 – 3.12 (m, 48H), 1.23 (d, J = 6.5 Hz, 3H, H-6 Fuc), 1.20 (d, J = 6.2 Hz, 3H, H-6 Rha).

^{13}C NMR (101 MHz, D_2O) δ 170.96, 103.50, 102.37, 100.80, 95.64, 78.38, 76.55, 75.88, 75.79, 75.38, 74.68, 74.40, 74.21, 74.13, 73.76, 73.08, 72.85, 70.71, 70.24, 69.51, 69.35, 60.67, 17.46, 15.51.

ESI-HRMS m/z 1445.503 $[M+H]^+$ ($C_{54}H_{93}O_{44}$ requires 1445.503), 1467.482 $[M+Na]^+$ ($C_{54}H_{92}O_{44}Na$ requires 1467.485).

RP-HPLC of a-9mer-IV (ELSD trace, Method A2, tR = 39.7, 40.2 min)

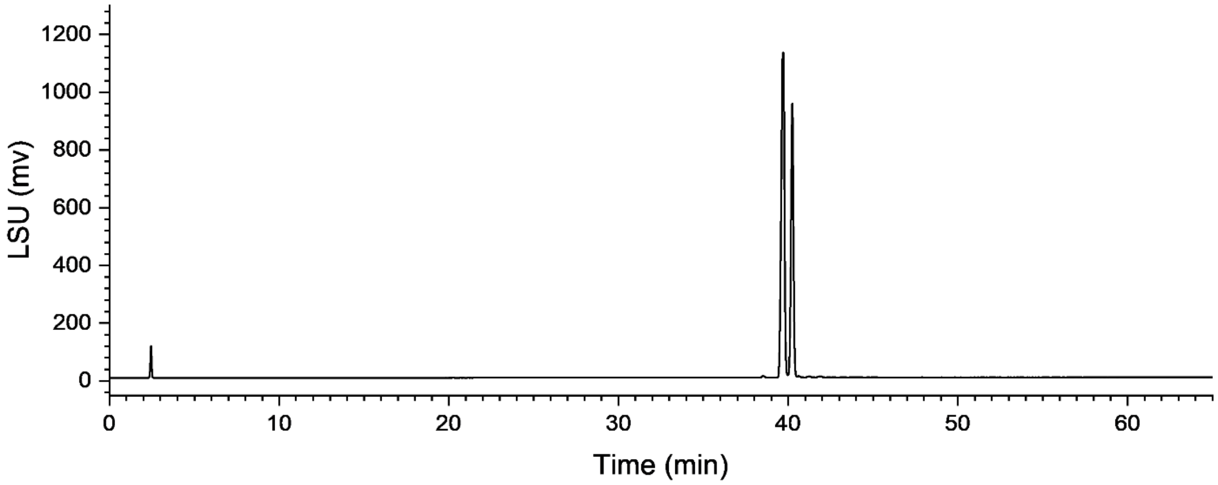

^{1}H NMR of a-9mer-IV (400 MHz, D_2O)

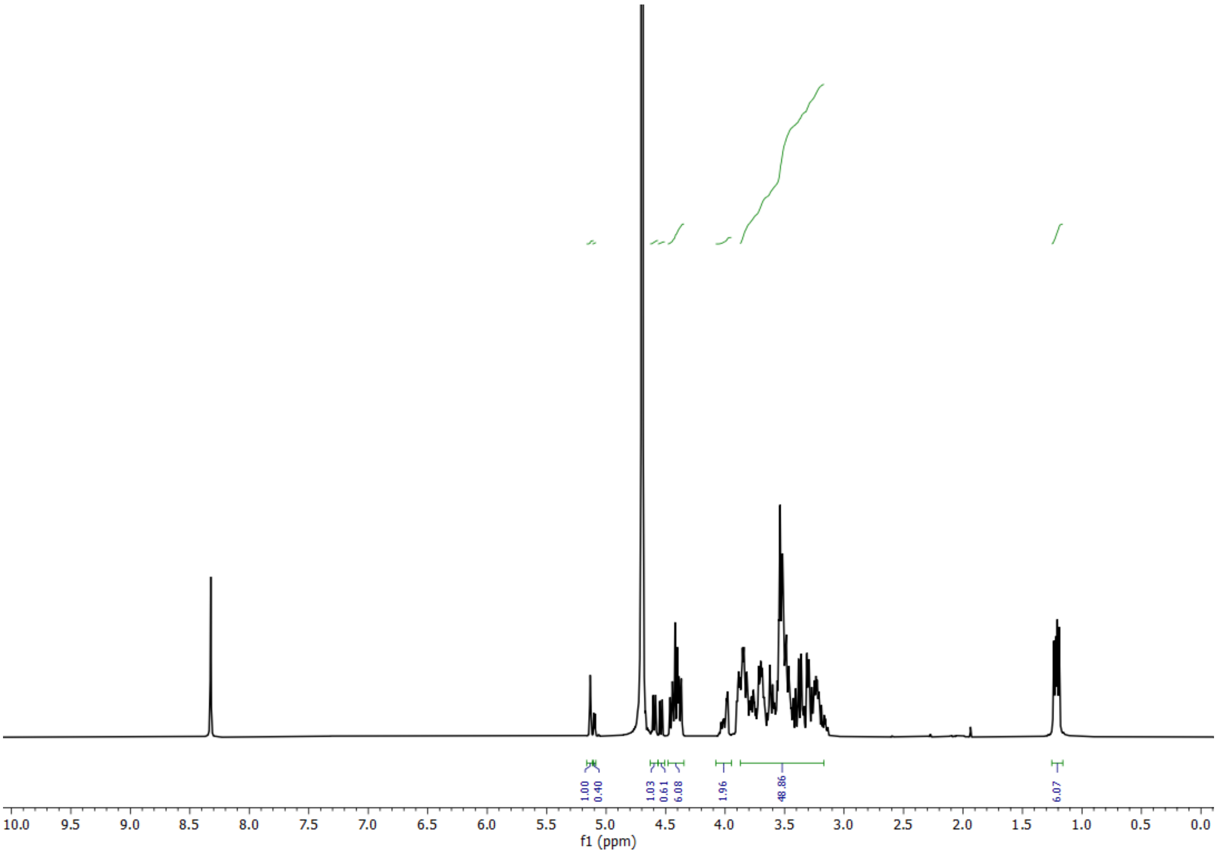

^{13}C NMR of a-9mer-IV (101 MHz, D_2O)

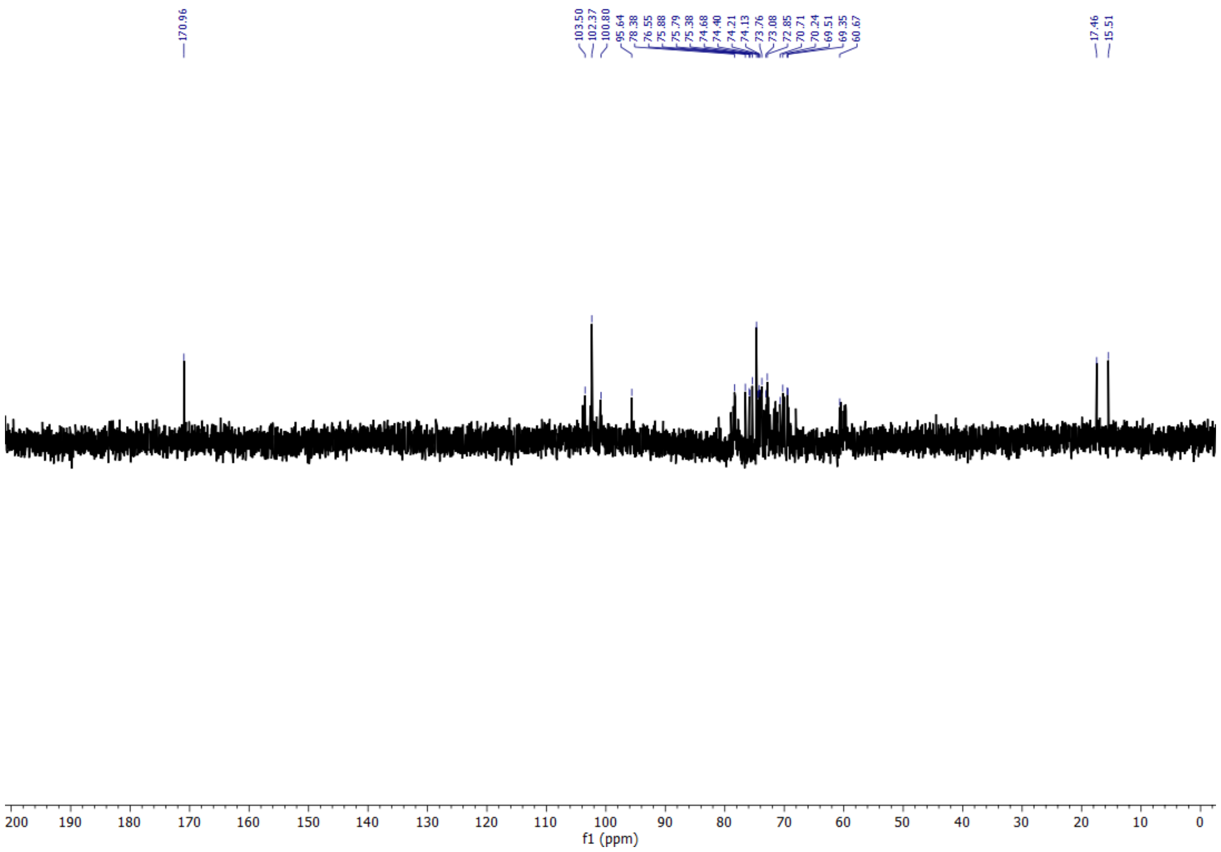

COSY NMR of a-9mer-IV (D_2O)

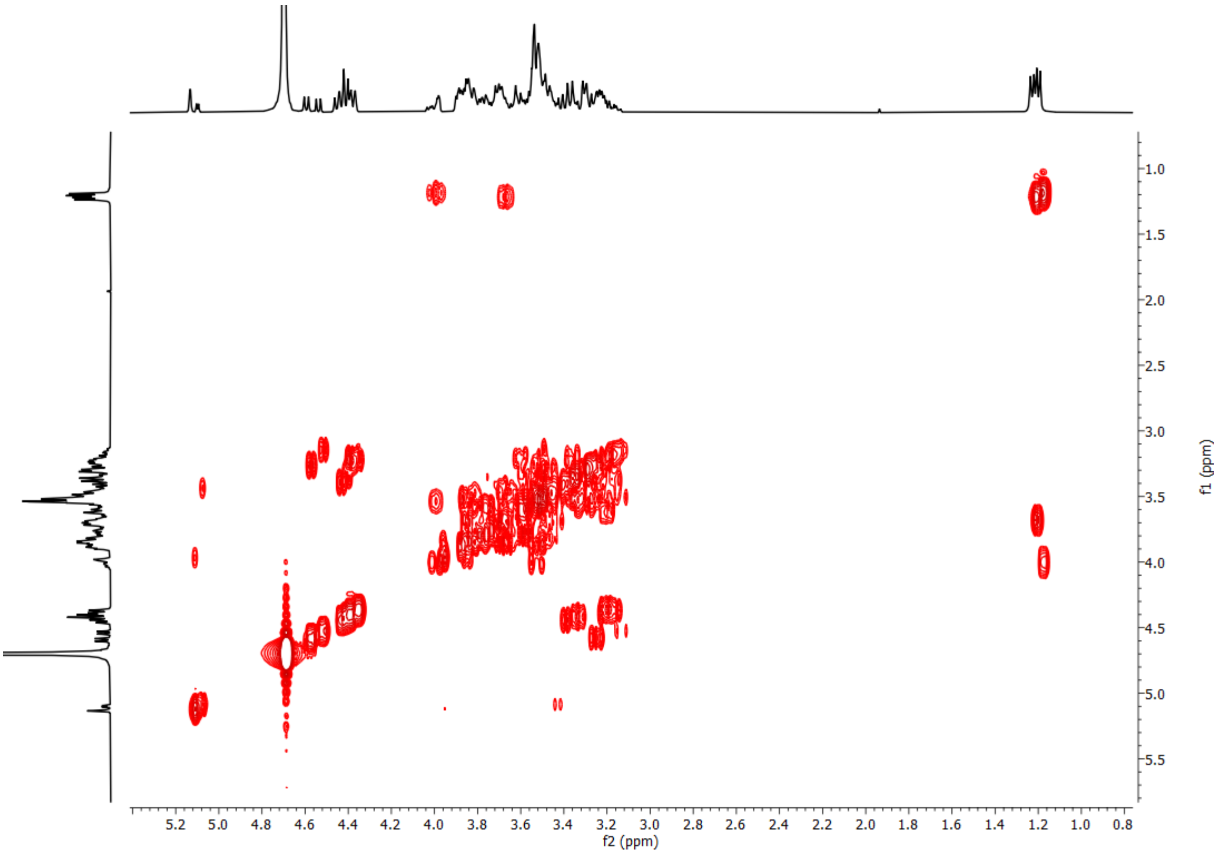

HSQC NMR of a-9mer-IV (D_2O)

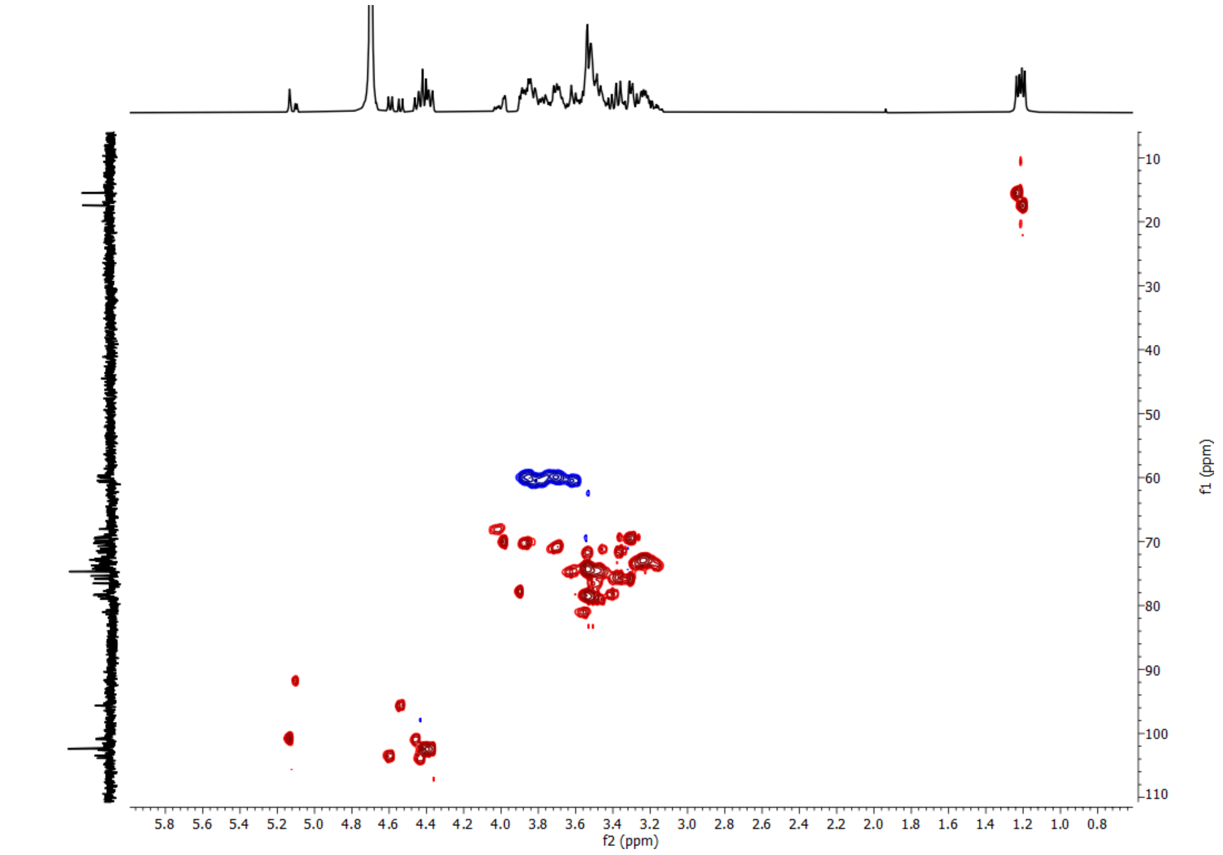

4.5.6 ttt-15mer-IV

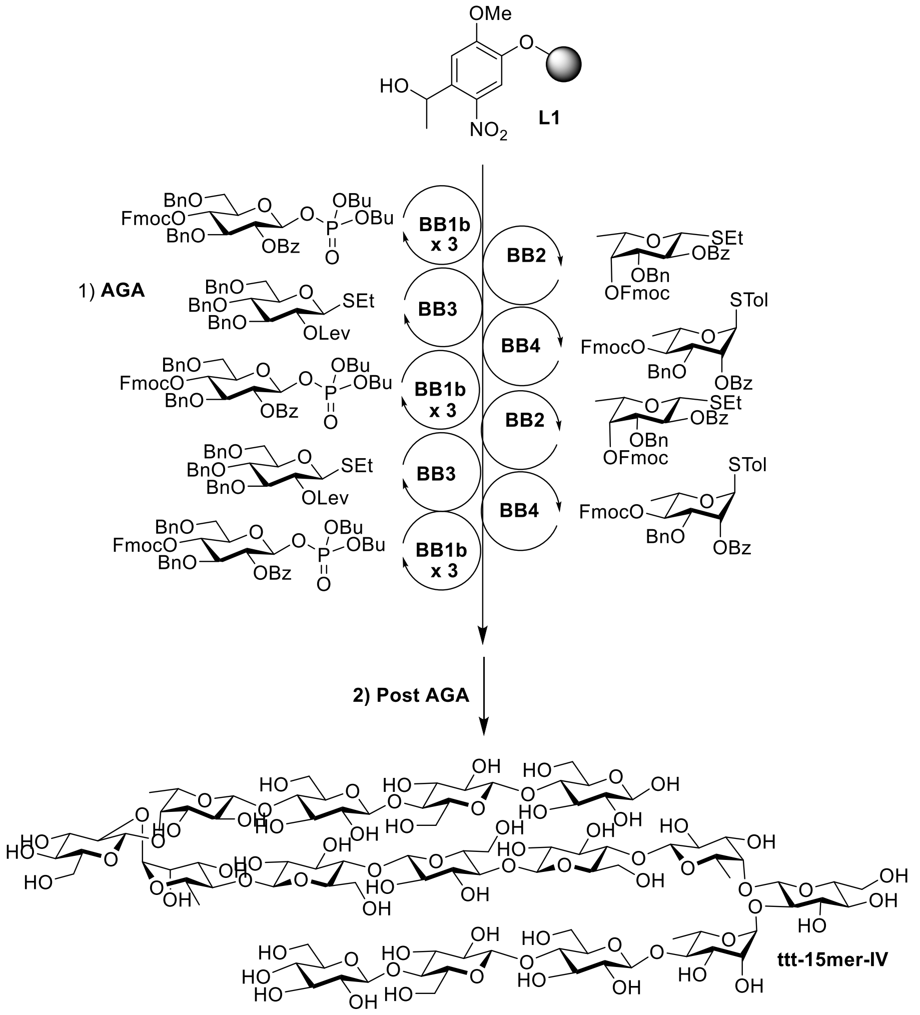

Step	BB	Modules	Notes
AGA	-	**A**	**L1** swelling
	(**BB1b**)x3	(**B, C2, D, E1**)x3	**C1:** (**BB1b**, -30 °C for 5 min, -10 °C for 40 min)
	**BB2**	**B**, **C1***, **D**, **E1**	**C1**: (**BB2**, -20 °C for 5 min, 0 °C for 20 min)<br>*Double cycle
	**BB3**	**B**, **C1***, **D**, **E2**	**C1**: (**BB3**, -20 °C for 5 min, 0 °C for 20 min)<br>*Double cycle
	**BB4**	**B**, **C1**, **D**, **E1**	**C1**: (**BB4a**, -20 °C for 5 min, 0 °C for 20 min)
	**BB1b**	**B, C2*, D, E1**	**C1:** (**BB1b**, -30 °C for 5 min, -10 °C for 40 min)<br>*Double cycle
	(**BB1b**)x2	(**B, C2, D, E1**)x2	**C1:** (**BB1b**, -30 °C for 5 min, -10 °C for 40 min)
	**BB2**	**B**, **C1***, **D**, **E1**	**C1**: (**BB2**, -20 °C for 5 min, 0 °C for 20 min)<br>*Double cycle
	**BB3**	**B**, **C1***, **D**, **E2**	**C1**: (**BB3**, -20 °C for 5 min, 0 °C for 20 min)

			*Double cycle
	**BB4a**	**B**, **C1, D**, **E1**	**C1**: (**BB4a**, -20 °C for 5 min, 0 °C for 20 min)
	**BB1b**	**B, C2*, D, E1**	**C1:** (**BB1b**, -30 °C for 5 min, -10 °C for 40 min) *Double cycle
	(**BB1b**)x2	(**B, C2, D, E1**)x2	**C1:** (**BB1b**, -30 °C for 5 min, -10 °C for 40 min)
**Post-AGA**	-	**F**, **G1**, **H2**, **I**	**F**: (3 h); **H2**: (7 h); **I**: (Method A3: 35.1, 35.5 min)

Automated synthesis, global deprotection, and purification afforded **ttt-15mer-IV** as a white solid (1.5 mg, 5% overall yield).

^{1}H NMR (600 MHz, D_2O) δ 5.18 (s, 2H, H-1 Rha), 5.15 (d, J = 3.8 Hz, 0.4H, H-1α Glc), 4.64 (d, J = 8.0 Hz, 2H, H-1 Glc), 4.59 (d, J = 8.0 Hz, 0.6H, H-1β Glc), 4.52 – 4.41 (m, 12H), 4.08 – 4.01 (m, 4H), 3.94 – 3.64 (m, 38H), 3.62 – 3.25 (m, 56H), 1.27 (d, J = 6.4 Hz, 6H, H-6 Fuc), 1.25 (d, J = 6.1 Hz, 6H, H-6 Rha).

ESI-HRMS m/z 1193.413 $[M+2H]^{2+}$ ($C_{90}H_{154}O_{74}$ requires 1193.419).

RP-HPLC of ttt-15mer-IV (ELSD trace, Method A4, tR = 35.1, 35.5 min)

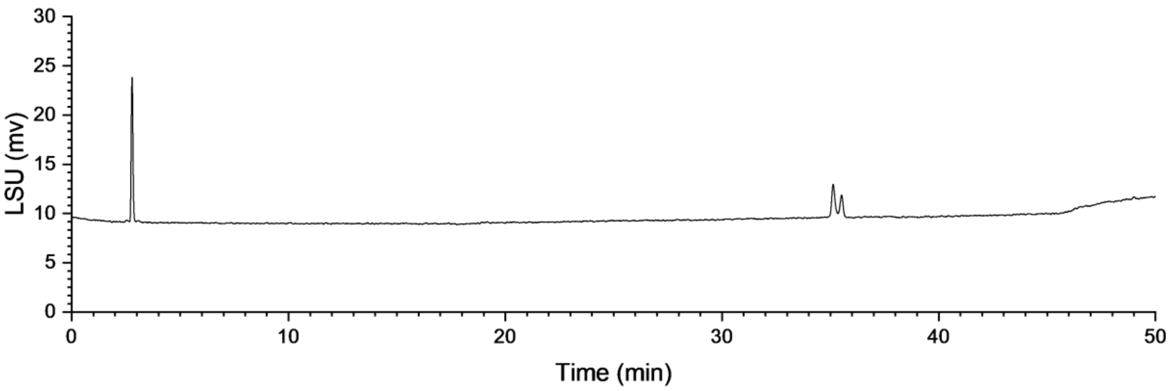

^{1}H NMR of ttt-15mer-IV (600 MHz, D_2O)

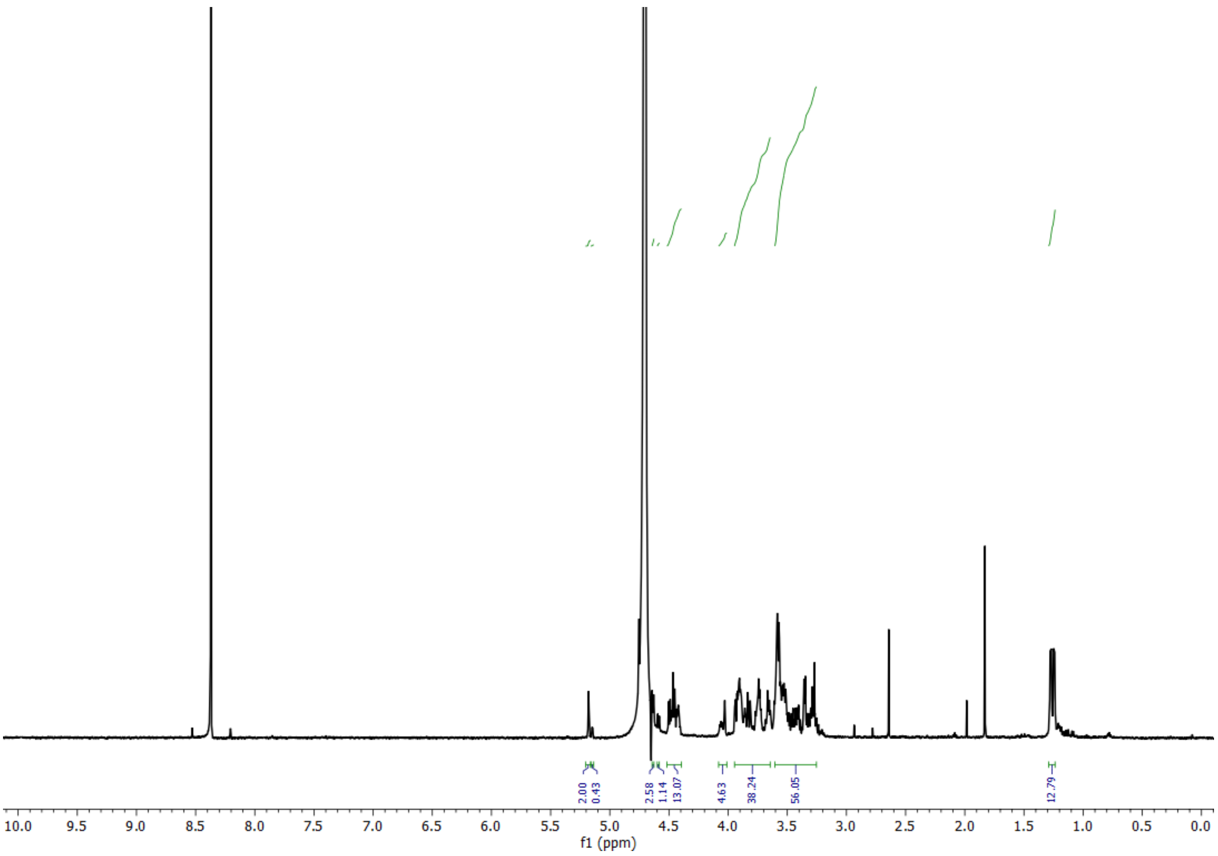

COSY NMR of ttt-15mer-IV (D_2O)

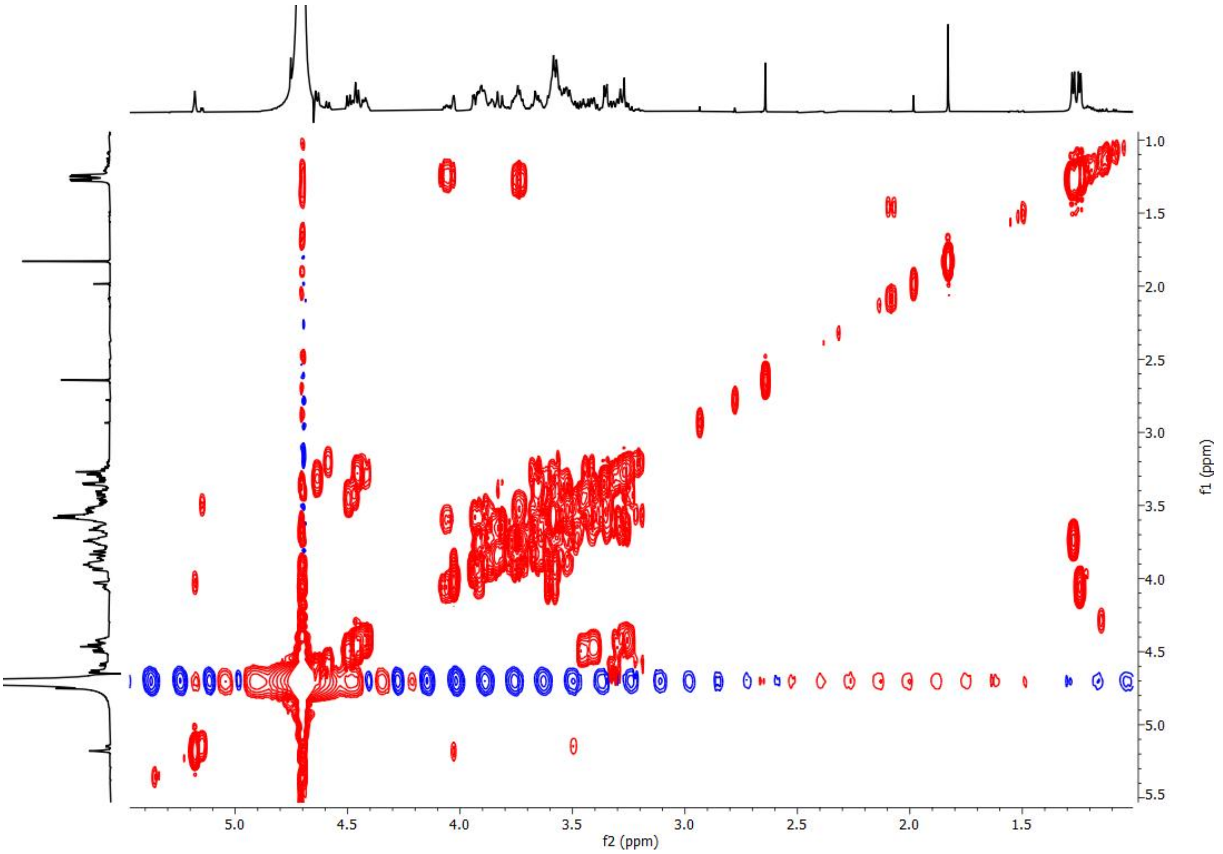

HSQC NMR of ttt-15mer-IV (D_2O)

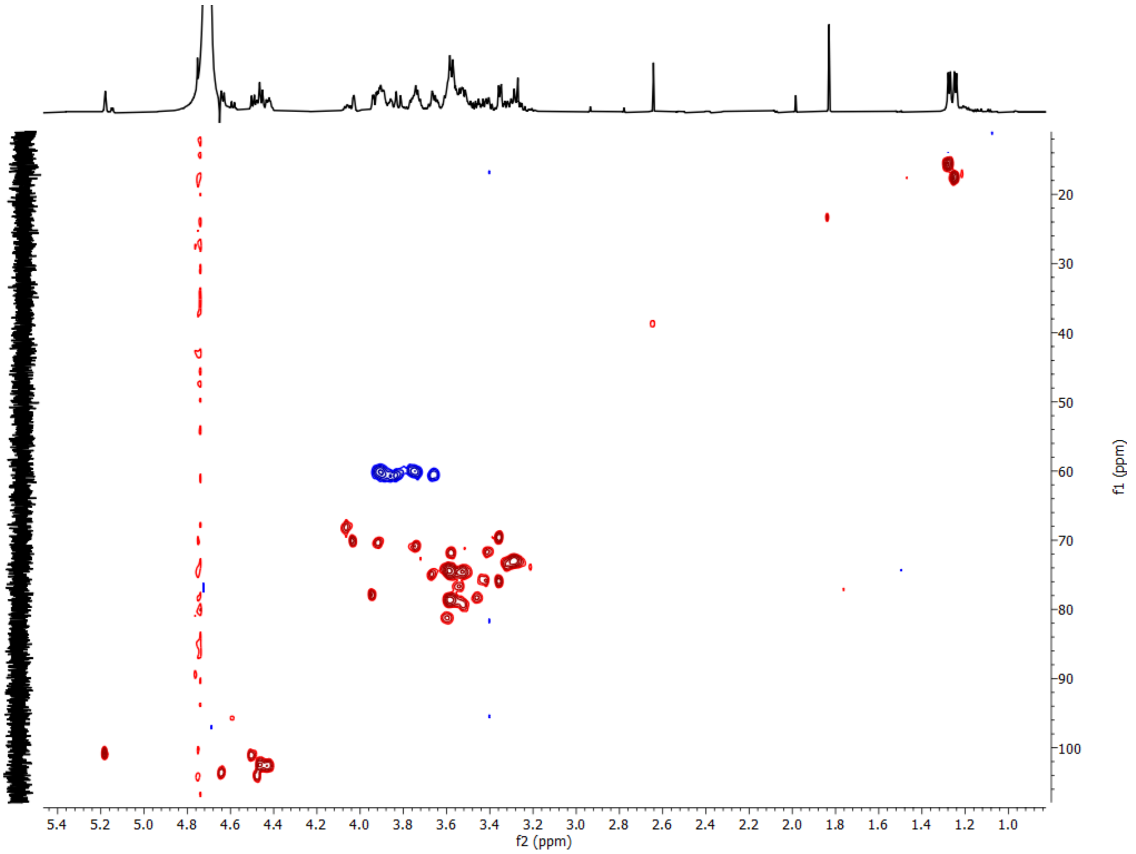

5 Molecular dynamics simulations

5.1 General materials and methods

All-atom molecular dynamics (MD) simulations were performed using Gromacs 5.1.2[66]. The oligosaccharides were modelled using a modified version of $GLYCAM06_{OSMO,r14}$ force field,[7,8] and the system was solvated with TIP5P[9] water molecules to avoid excessive interactions between the monomers. The topology was converted to gromacs format using the glycam2gmx.pl script and solvated with 2100 water molecules using gromacs tools. The systems were kept at a constant temperature of 303 K using a Nosé-Hoover thermostat[10,11] and at constant pressure of 1 bar with the Parrinello-Rahman Barostat[12,13]. Non-bonded interactions were cut-off at 1.4 nm, long range electrostatics were calculated using the particle mesh method[14]. Bonds involving hydrogens were constrained using the LINCS[15] algorithm to allow a 2 fs time step algorithm; water molecules were kept rigid with SETTLE[16] algorithm.

After energy minimization (steepest descent algorithm) and before the production run, the systems were equilibrated at 300 K for 50 ns in a canonical (NVT) ensemble (constant number of particles, volume and temperature) and subsequently at 300 K and 1 atm for 50 ns in an isothermal-isobaric (NPT) ensemble. All the modelled structures were simulated for 500 ns. The End-to-end distance was calculated from the O-1 of the reducing end to the O-4 of non-reducing end and the inter-residue distances from center of mass of each residue.

5.2 RMSD, EtE distance and Rg of a-3mer-I, a-3mer-IV, and a-3mer-II

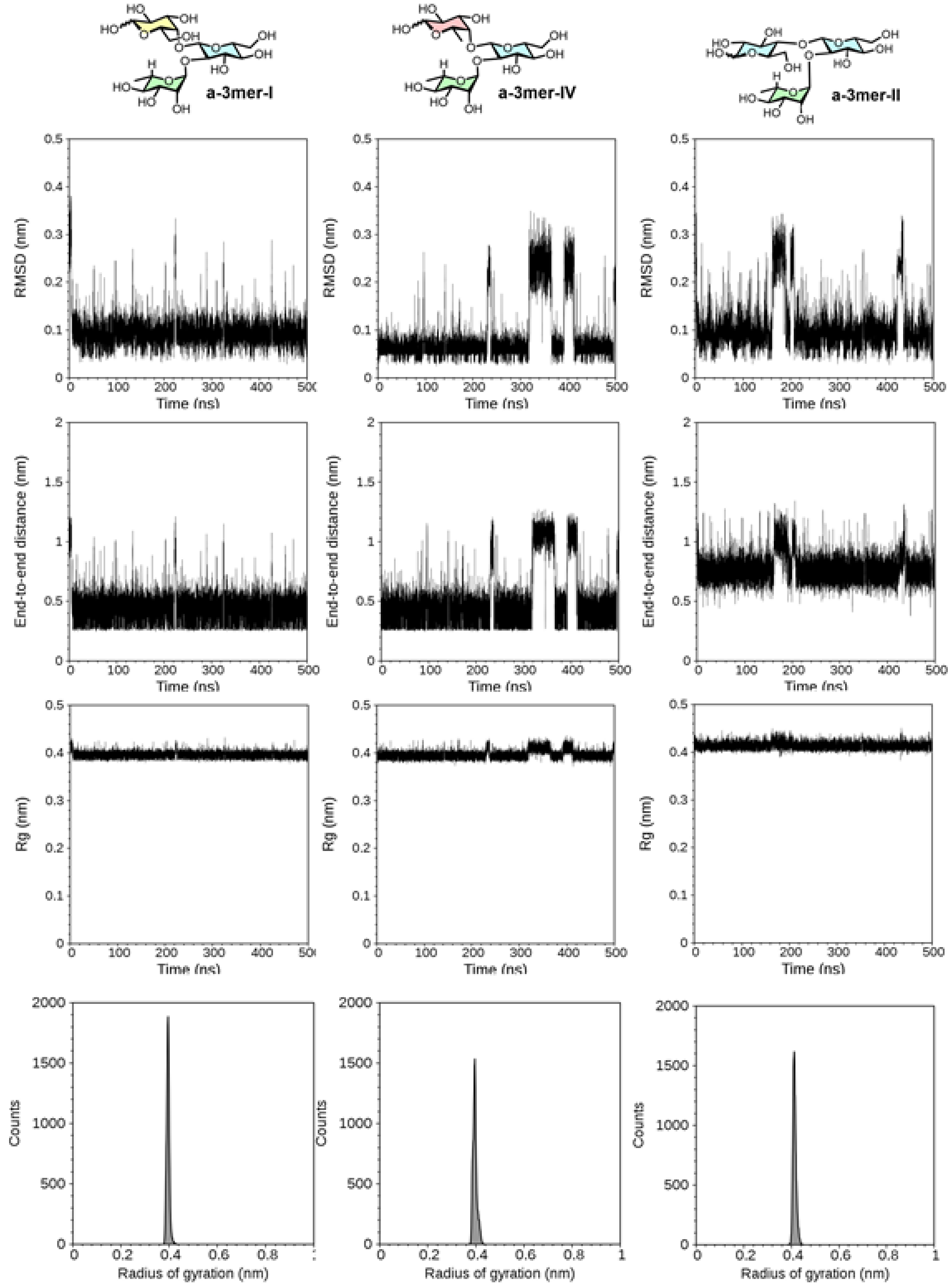

Figure S1 RMSD, End-to-End distance and Rg of **a-3mer-I, a-3mer-II** and **a-3mer-IV**. The End-to-End distance was calculated using the C-4 hydroxyl group of the non-reducing end and C-1 hydroxyl group of the reducing end.

5.3 Inter-residue distance and Ramachandran plots of 3mers

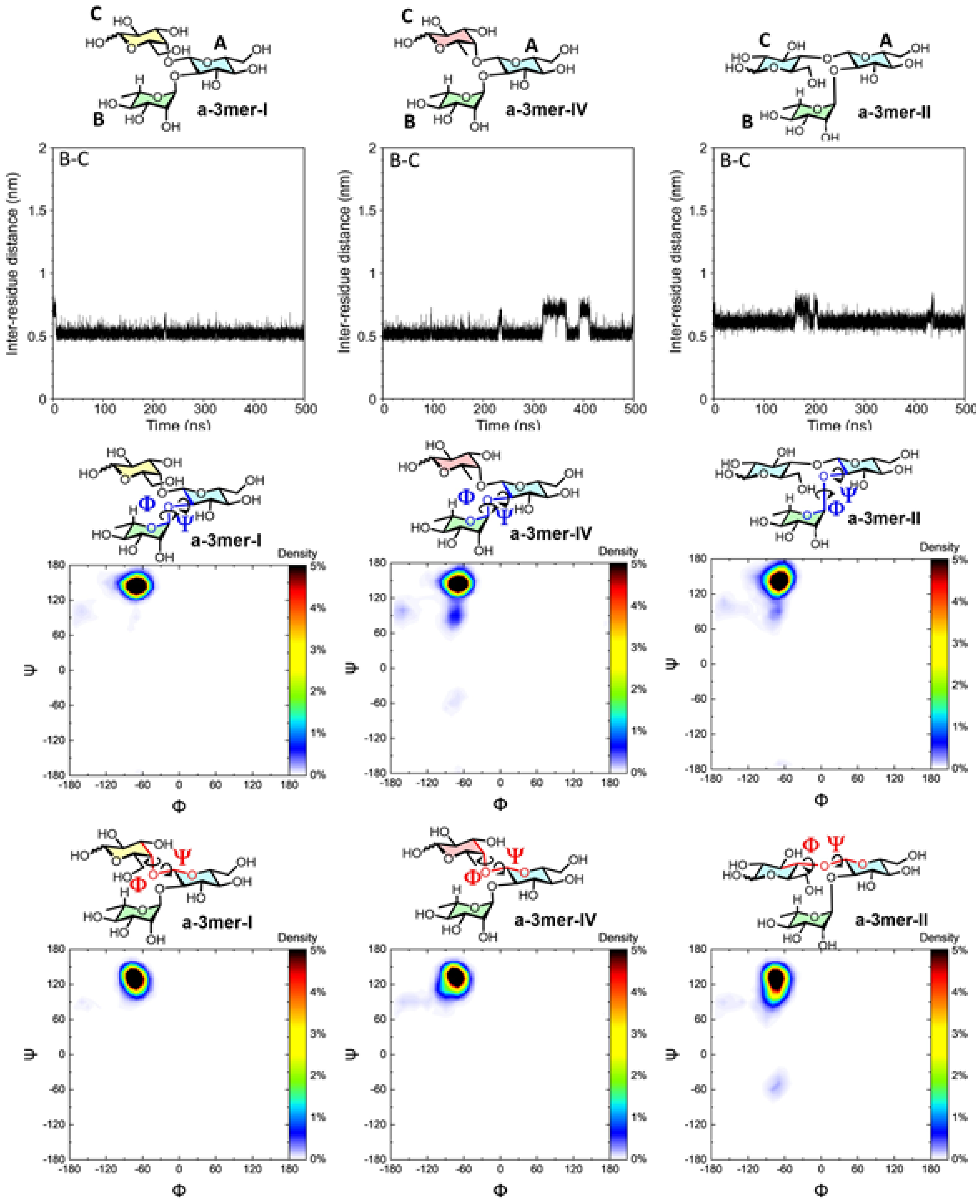

Figure S2 Inter-residue distance of **a-3mer-I, a-3mer-II** and **a-3mer-IV** between center of mass of residues in both ends and Ramachandran plots (Φ: $O^{5'}$-$C^{1'}$-O^{n}-C^{n}, Ψ: $C^{1'}$-O^{n}-C^{n}-C^{n-1}).

5.4 MD analysis of natural 3mer

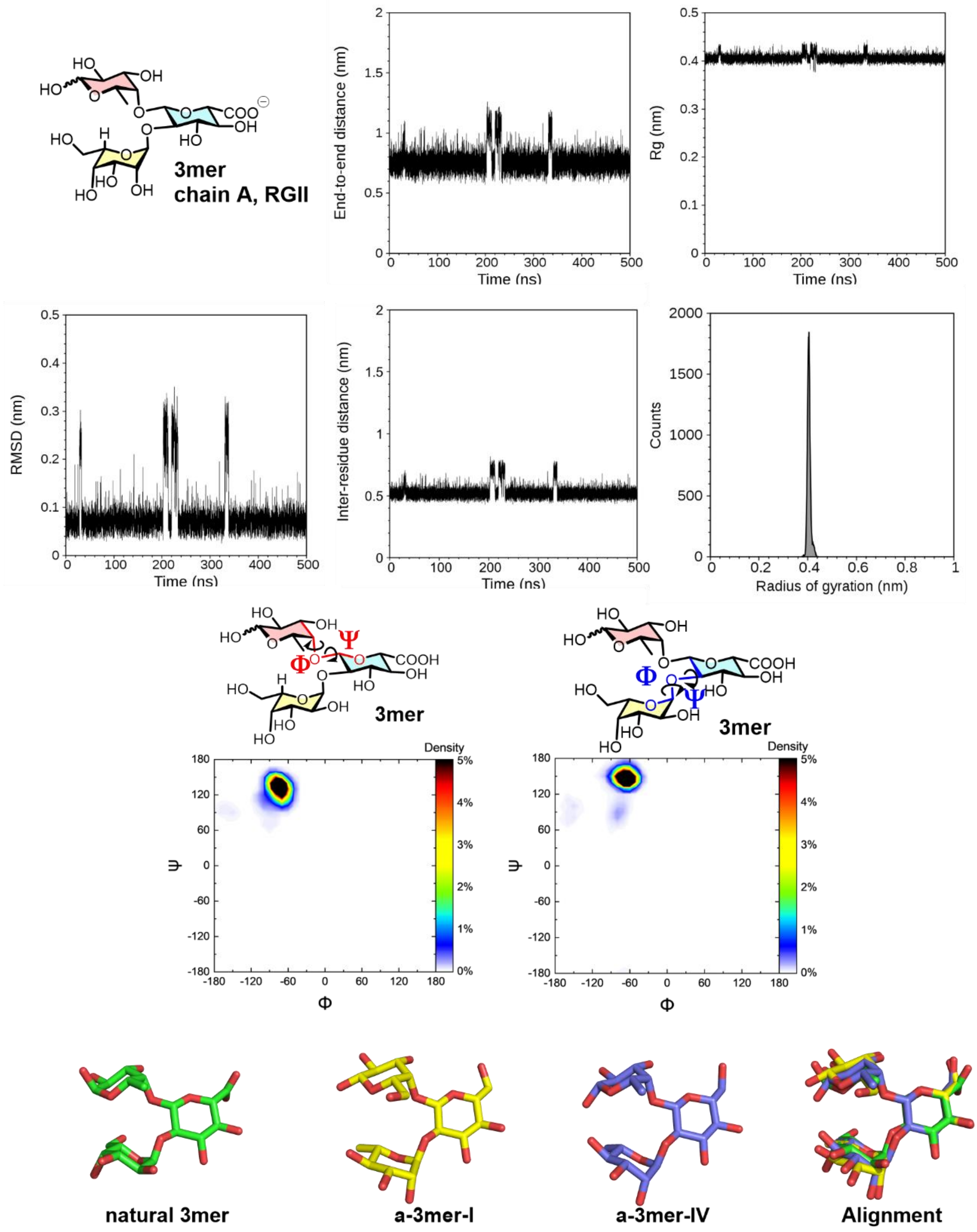

Figure S3 RMSD, End-to-End distance, inter-residue distance, Rg and Ramachandran plots of the natural **3mer** from rhamnogalacturonan II chain A. The End-to-End distance was calculated using the C-4 hydroxyl group of the non-reducing end and C-1 hydroxy group of the reducing end. Inter-residue distance was calculated between center of mass of residues in both ends. Representative snapshot and alignment of the natural **3mer**, **a-3mer-I** and **a-3mer-IV** showing backbone similarity.

5.5 RMSD, EtE distance and Rg of a-5mer-I, a-5mer-IV, and a-5mer-II

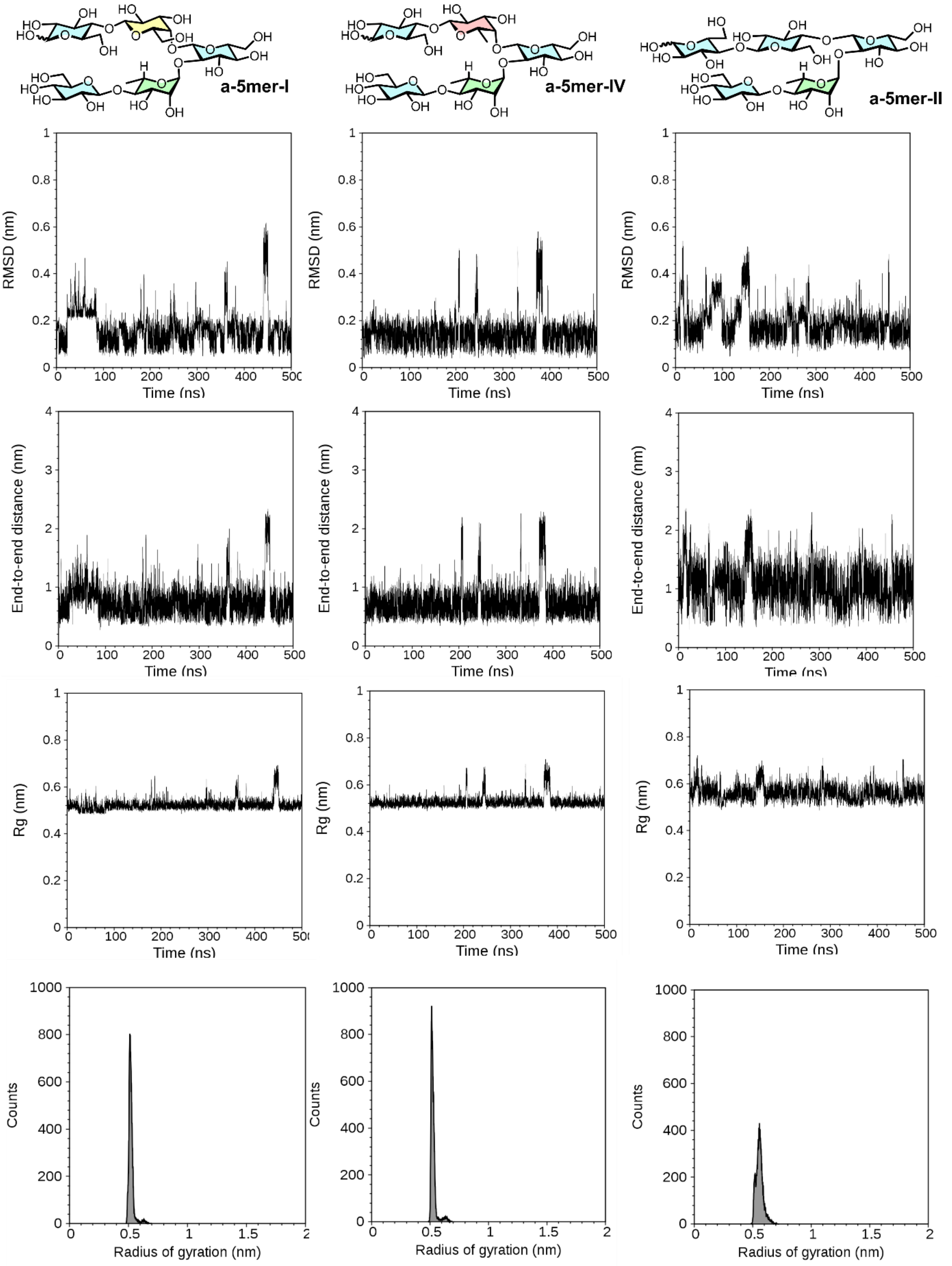

Figure S4 RMSD, End-to-End distance and Rg of **a-5mer-I, a-5mer-II** and **a-5mer-IV**. The End-to-End distance was calculated using the C-4 hydroxyl group of the non-reducing end and C-1 hydroxyl group of the reducing end.

5.6 Inter-residue distance plots of a-5mer-I, a-5mer-IV, and a-5mer-II

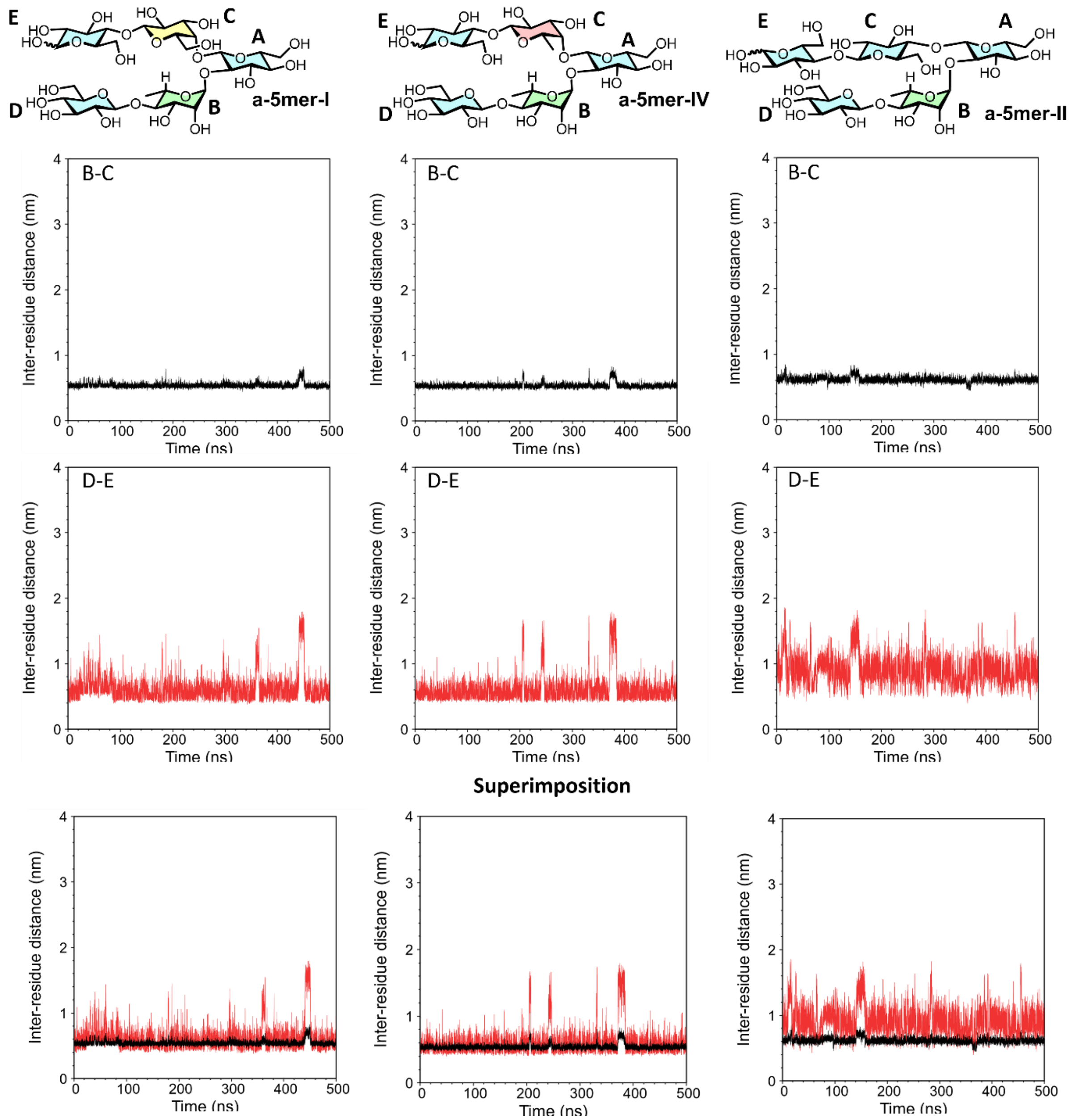

Figure S5 Inter-residue distance of **a-5mer-I, a-5mer-II** and **a-5mer-IV** between center of mass of homologous residues in both strands.

5.7 Ramachandran plots of a-5mer-I, a-5mer-IV, and a-5mer-II

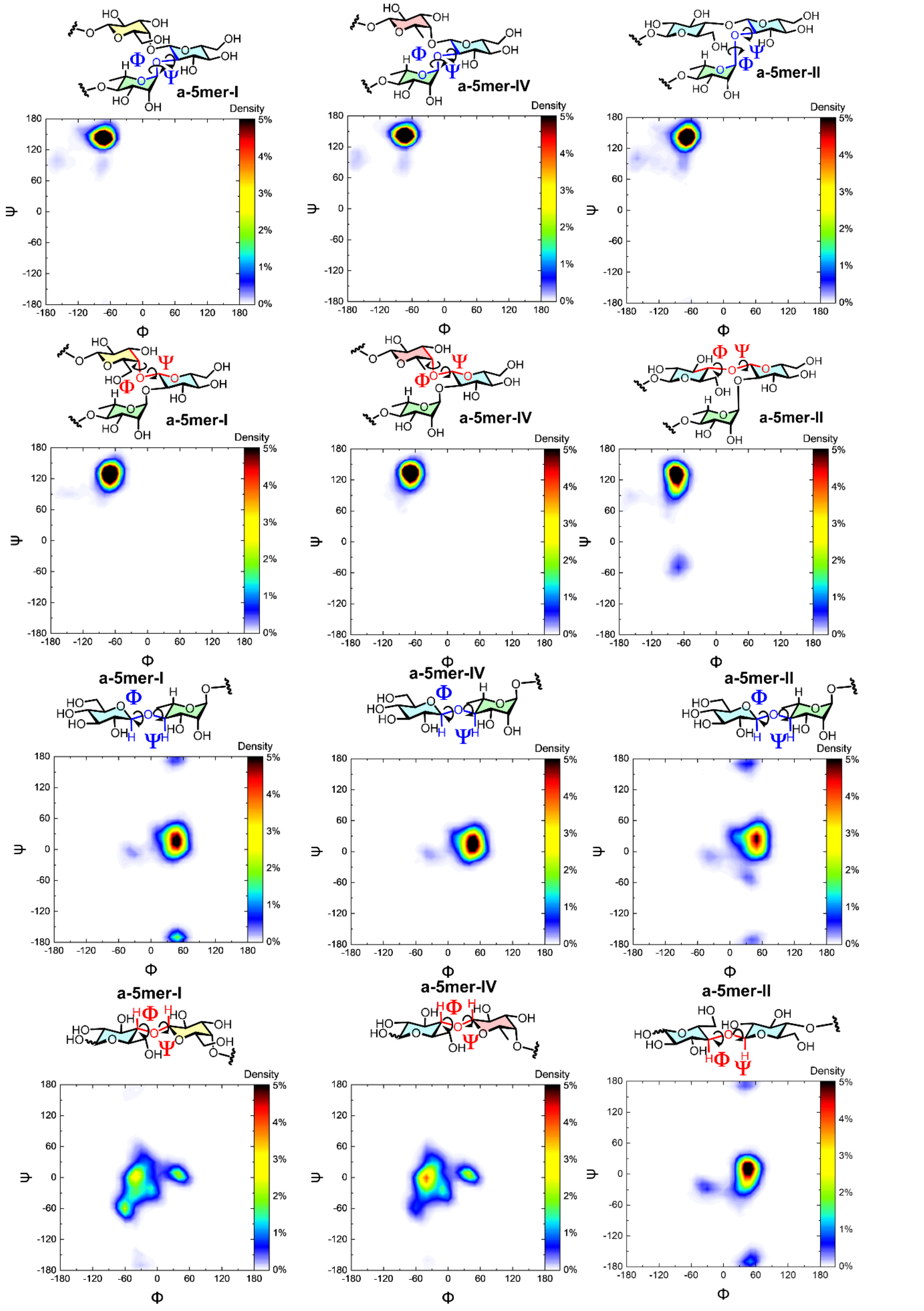

Figure S6 Ramachandran plot of **a-5mer-I, a-5mer-II** and **a-5mer-IV** (Turn unit linkages Φ: $O^{5'}$-$C^{1'}$-O^{n}-C^{n}, Ψ: $C^{1'}$-O^{n}-C^{n}-C^{n-1} and strand linkages Φ: $H^{1'}$-$C^{1'}$-O^{4}-C^{4}, Ψ: $C^{1'}$-O^{4}-C^{4}-H^{4}).

5.8 RMSD, EtE distance and Rg of a-9mer-I, a-9mer-IV, and a-9mer-II

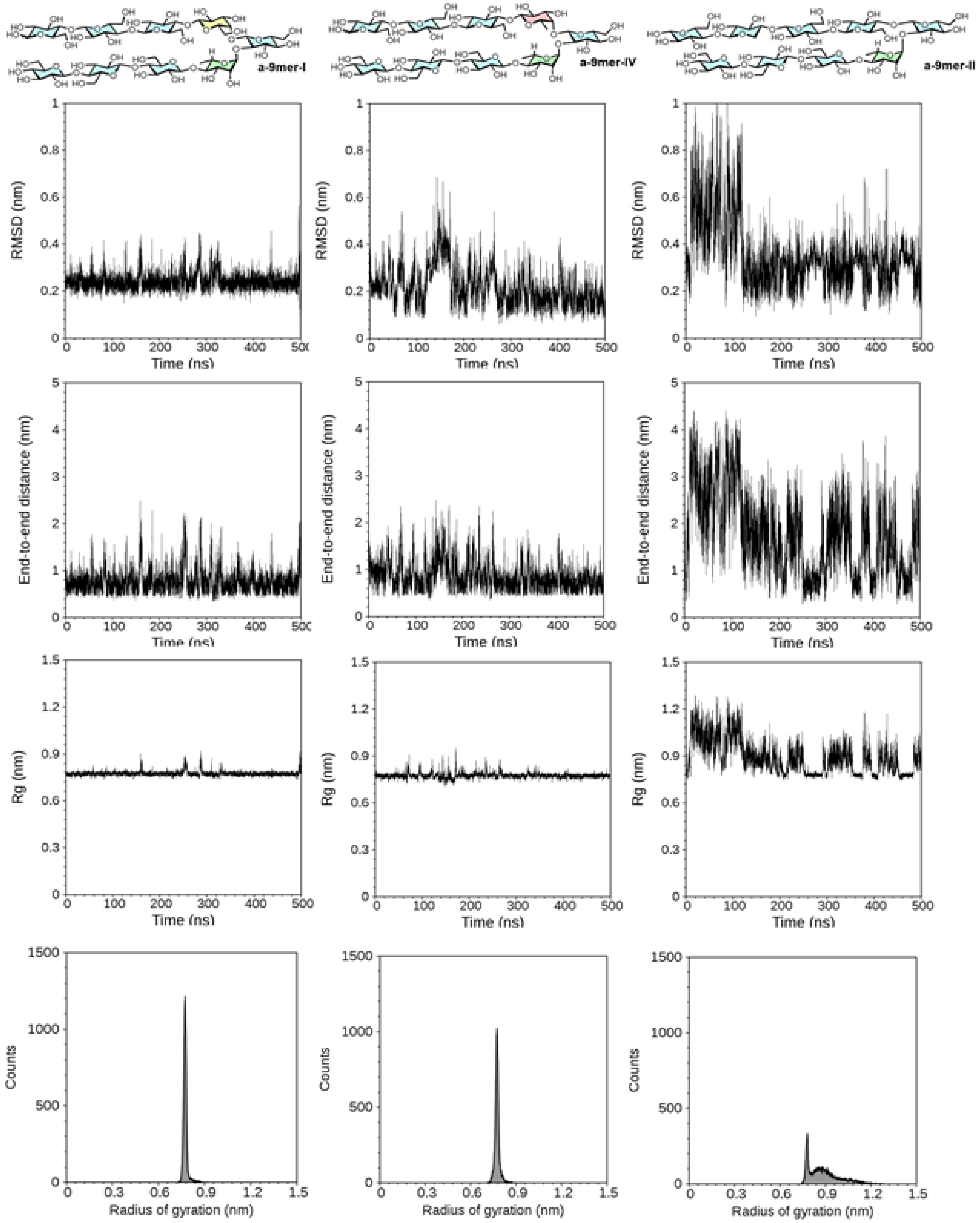

Figure S7 RMSD, End-to-End distance and Rg of **a-9mer-I**, **a-9mer-II** and **a-9mer-IV**. The End-to-End distance was calculated using the C-4 hydroxyl group of the non-reducing end and C-1 hydroxyl group of the reducing end.

5.9 Inter-residue distance plots of a-9mer-I, a-9mer-IV, and a-9mer-II

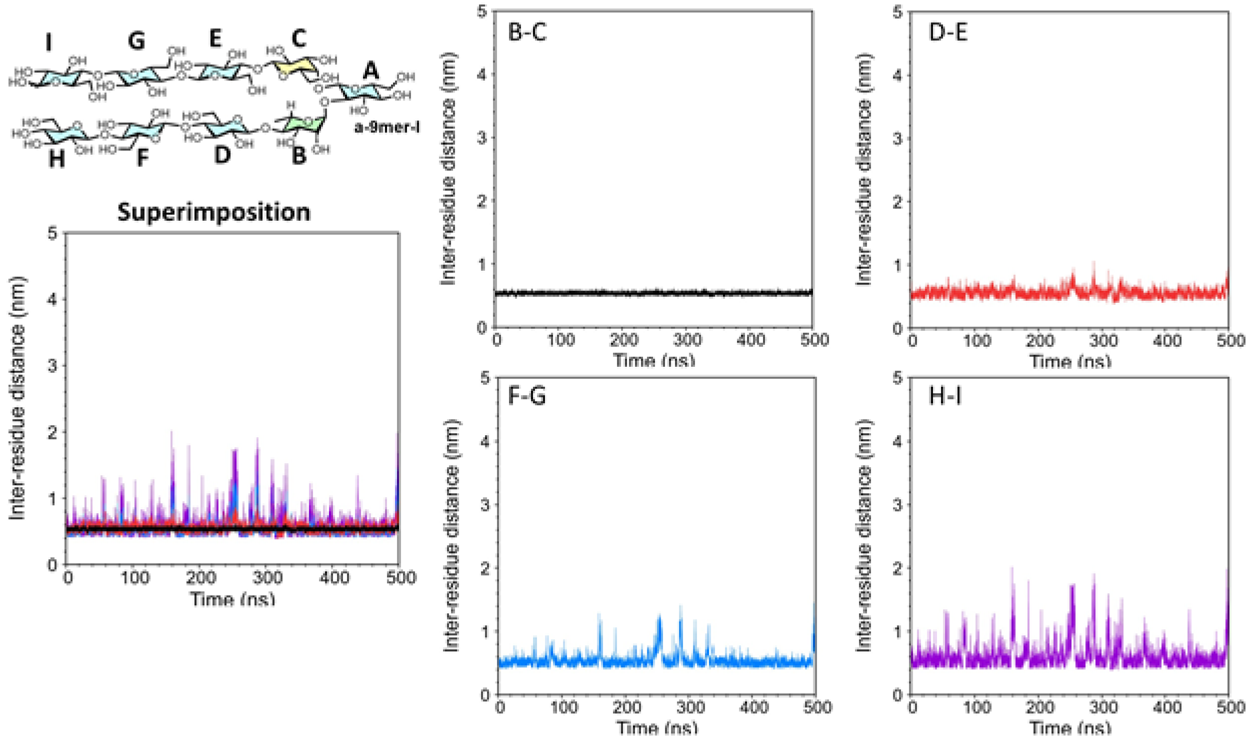

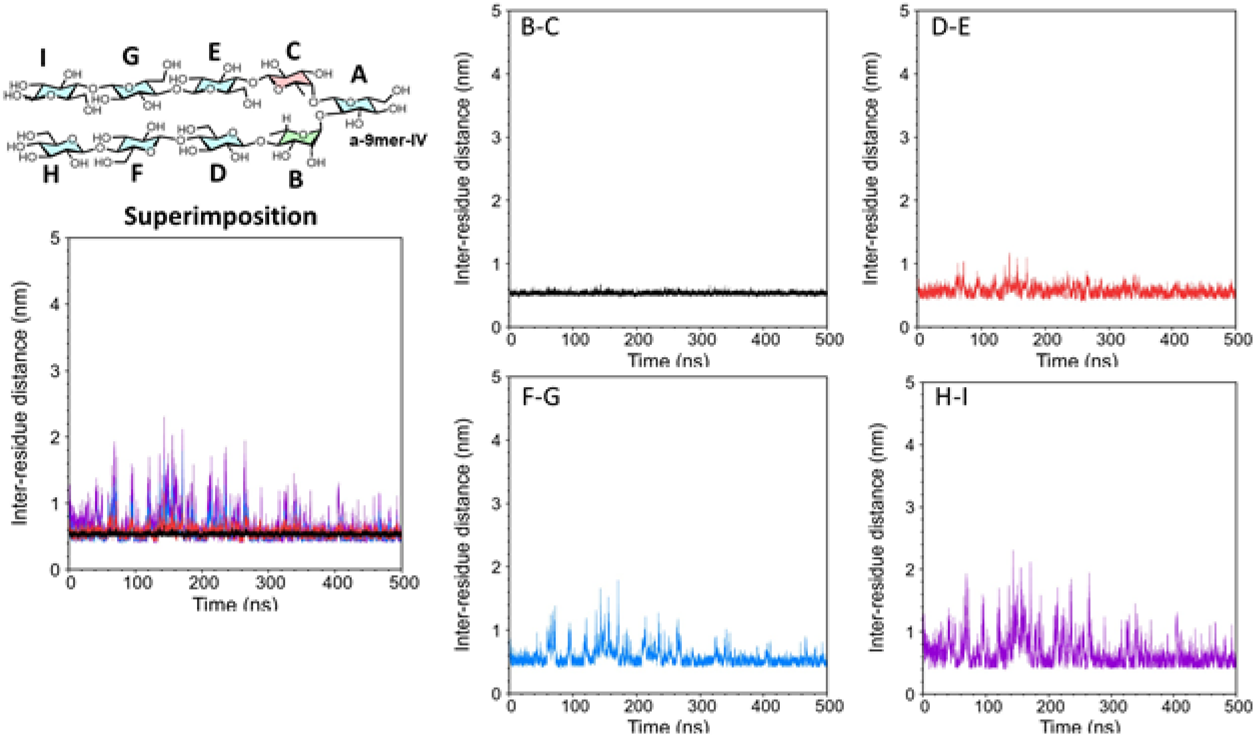

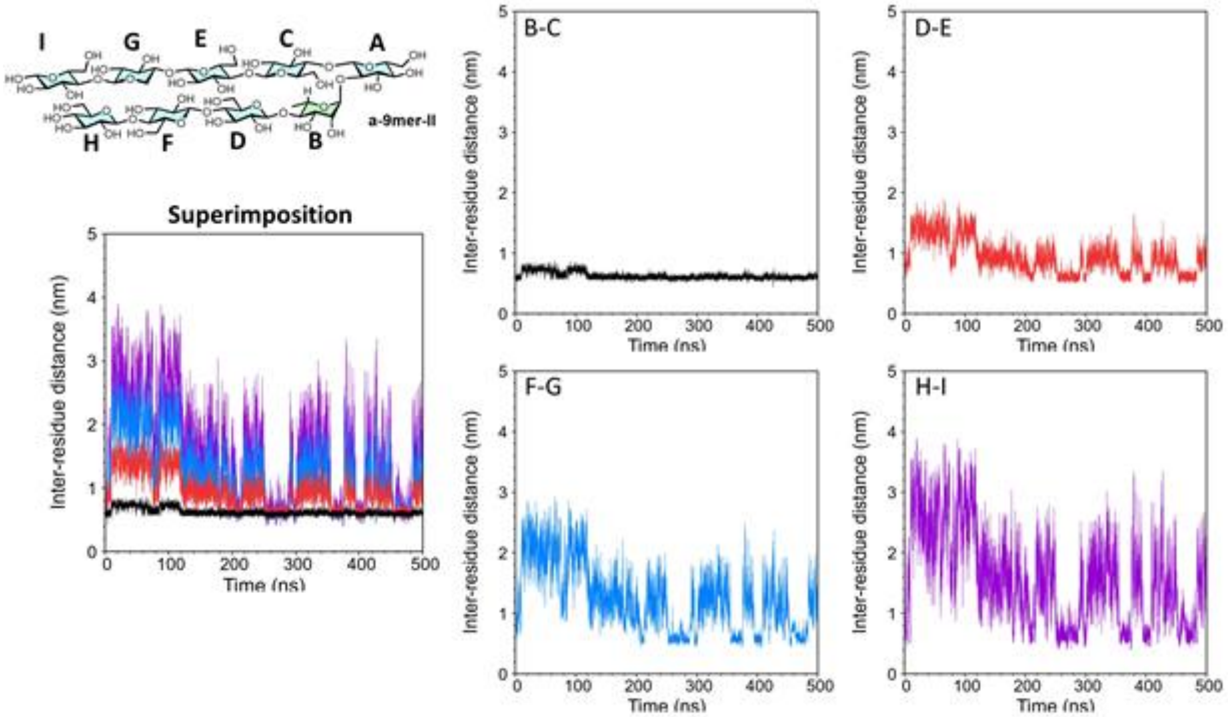

Figure S8 Inter-residue distance of **a-9mer-I, a-9mer-II** and **a-9mer-IV** between center of mass of homologous residues in both strands.

5.10 Ramachandran plots of a-9mer-I, a-9mer-IV, and a-9mer-II

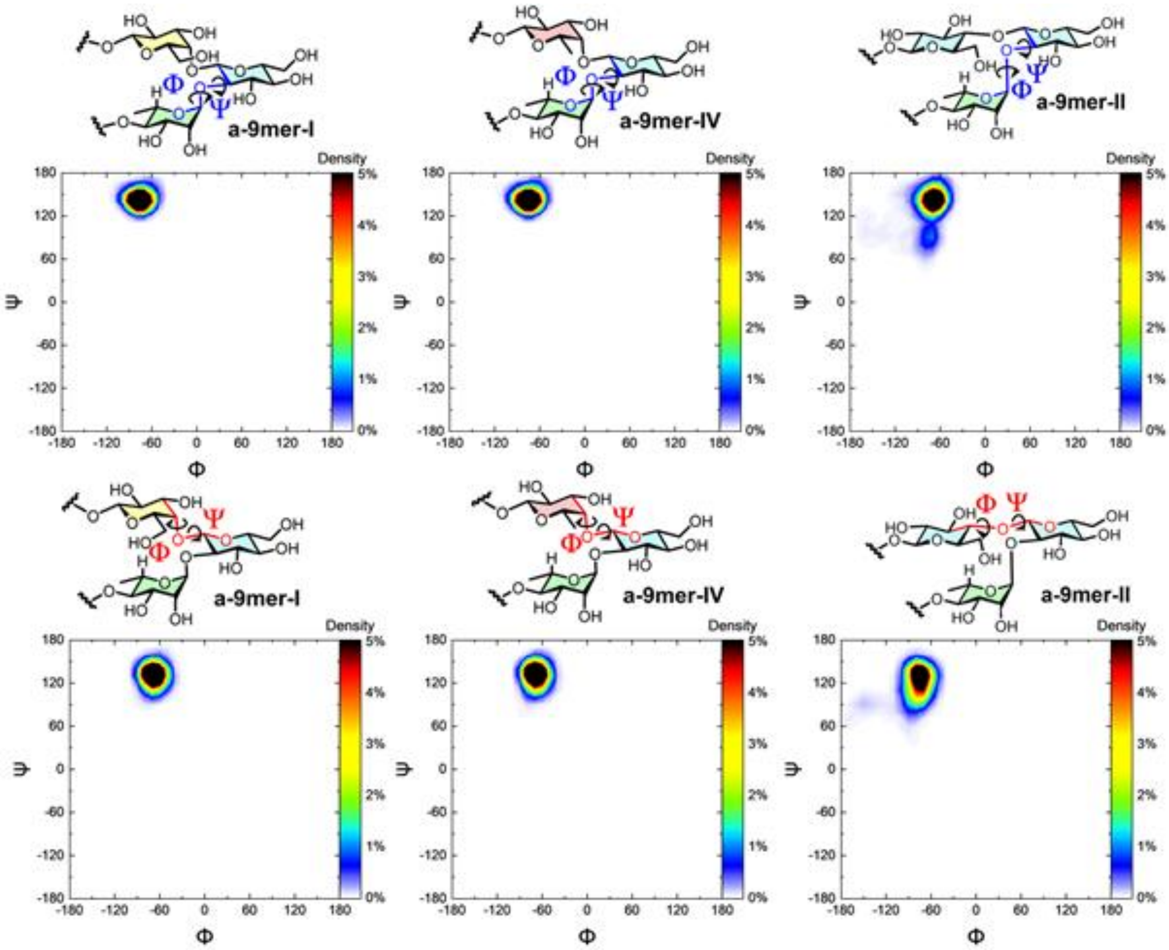

Figure S9 Ramachandran plot of **a-9mer-I, a-9mer-II** and **a-9mer-IV** on the turn unit (Φ: $O^{5'}$-$C^{1'}$-O^{n}-C^{n}, Ψ: $C^{1'}$-O^{n}-C^{n}-C^{n-1}).

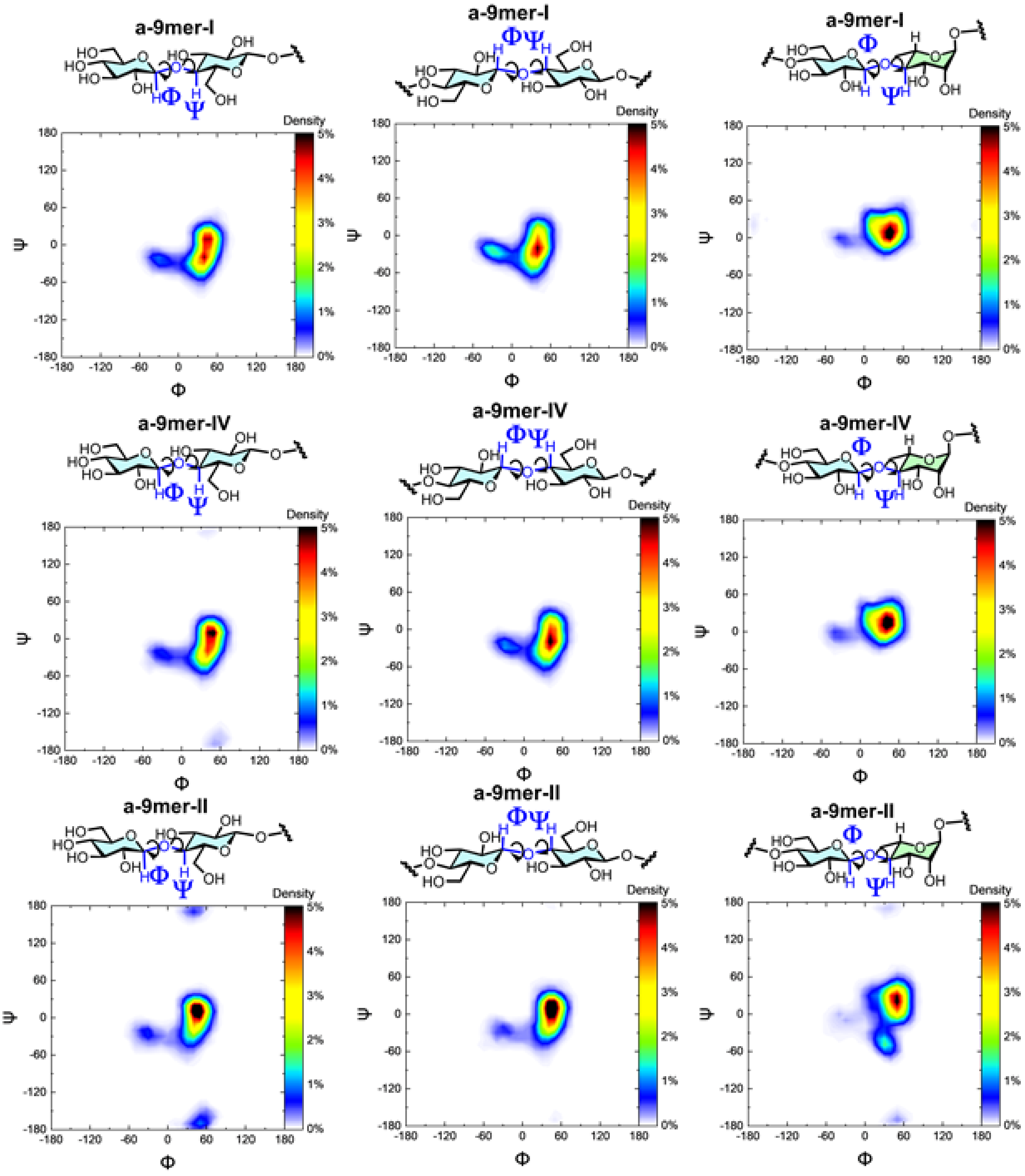

Figure S10 Ramachandran plot of **a-9mer-I, a-9mer-II** and **a-9mer-IV** on the strand with the non-reducing end (Φ: H1'-C1'-O4-C4, Ψ: C1'-O4-C4-H4).

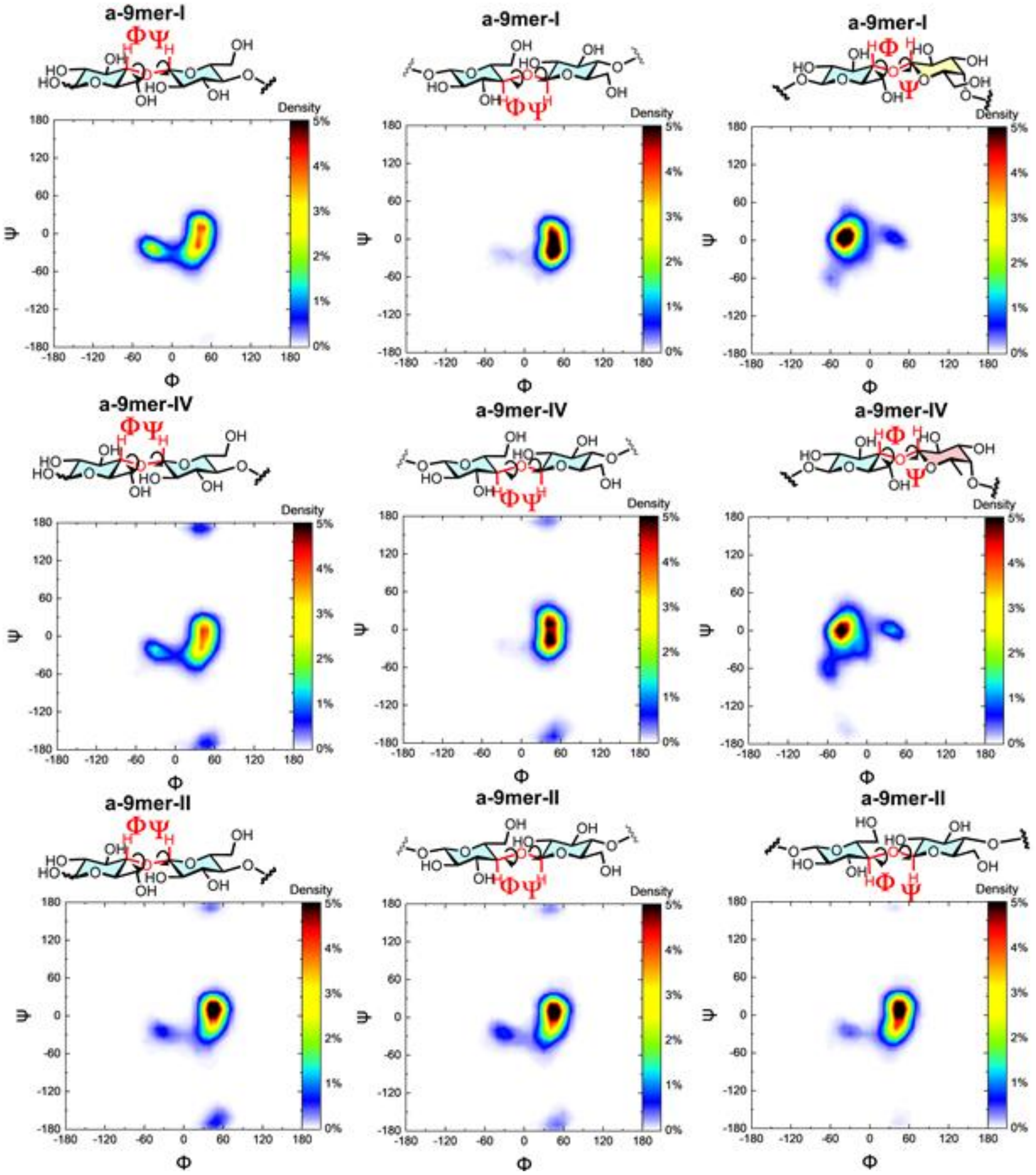

Figure S11 Ramachandran plot of **a-9mer-I, a-9mer-II** and **a-9mer-IV** on the strand with the reducing end (Φ: H$^{1'}$-C$^{1'}$-O^4-C^4, Ψ: C$^{1'}$-O^4-C^4-H^4).

5.11 Bar graph of average inter-residue distance of a-3mer-I, a-3mer-IV and a-3mer-II and related hairpins

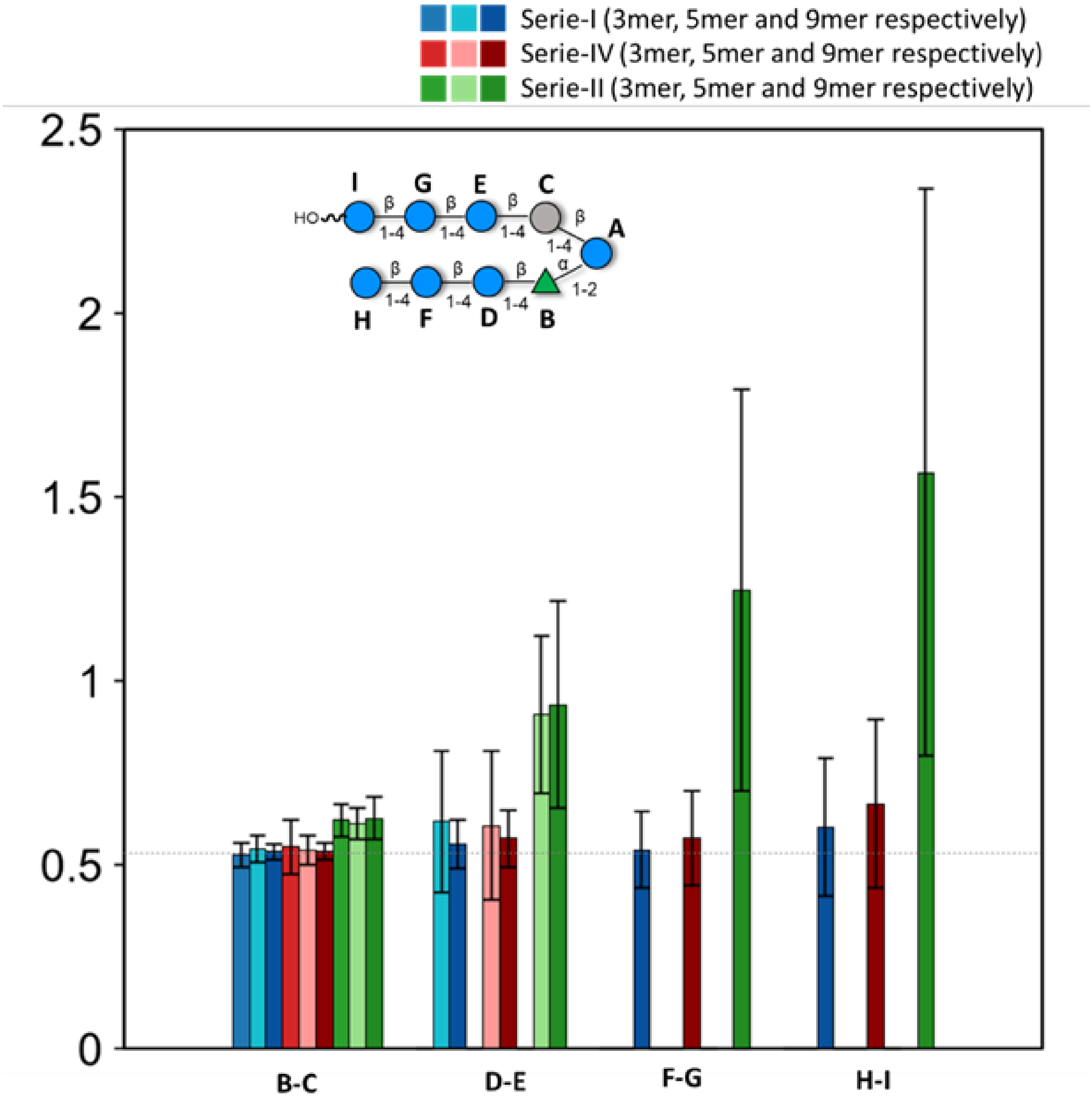

Figure S12 Bar graph showing the average inter-residue distance and their standard deviation of 3mers, 5mers and 9mers, based on turn units **a-3mer-I** (blue), **a-3mer-IV** (red) and **a-3mer-II** (green). Distances were calculated between center of mass of homologous residues in the two strands. MD simulation predict similar rigidity and compactness for comparable hairpins in series I and series IV, whereas series II adopt a more open conformation with greater flexibility. Stabilization of the hairpin fold is evident in series I and series IV: comparation of D-E inter-reside distance for 5mers and 9mers shows a reduction to values comparable with the B-C, accompanied by decreased fluctuations.

5.12 RMSD, EtE distance and Rg of ttt-15mer-I and ttt-15mer-IV

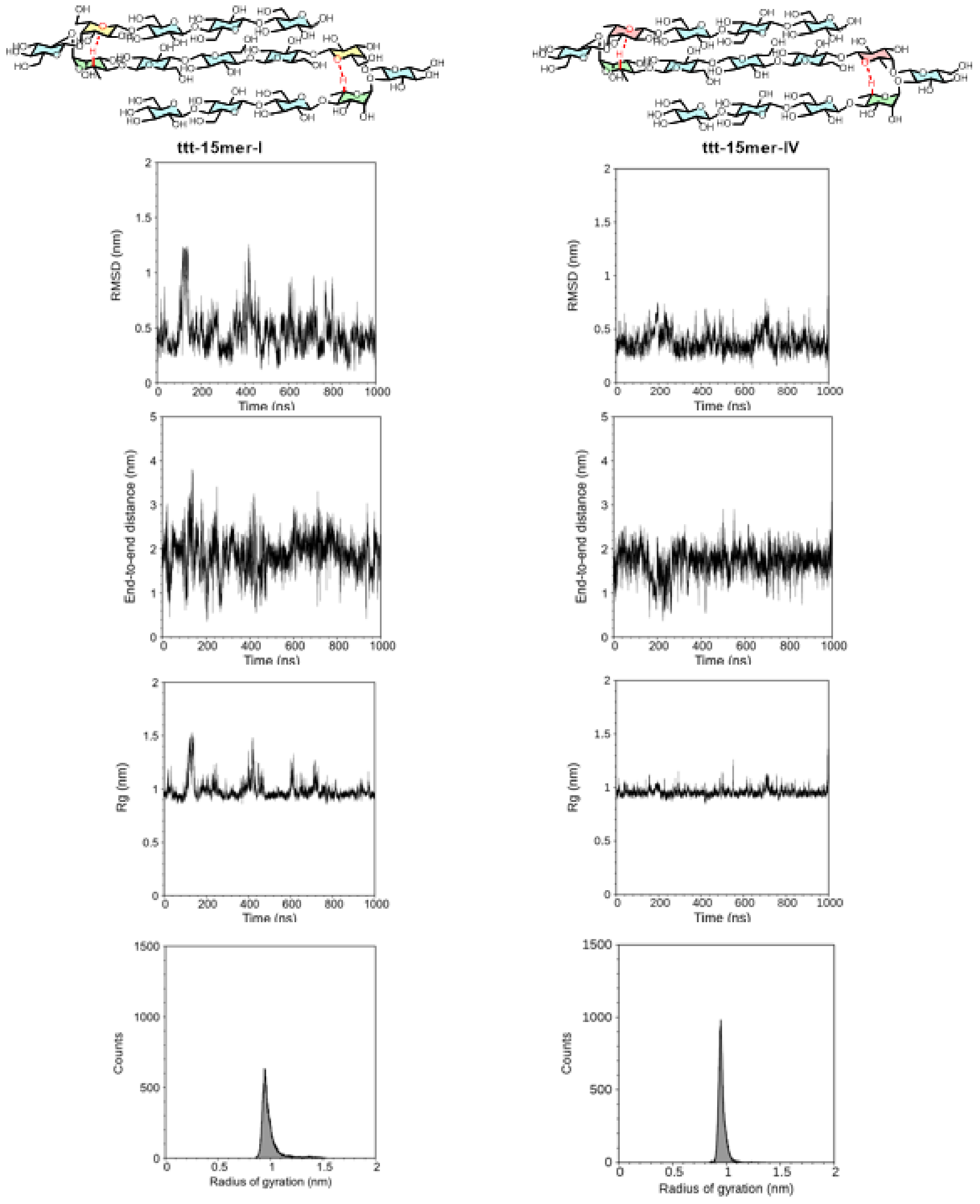

Figure S13 RMSD, End-to-End distance and Rg of **ttt-15mer-I** and **ttt-15mer-IV**. The End-to-End distance was calculated using the C-4 hydroxy group of the non-reducing end and C-1 hydroxy group of the reducing end.

5.13 Inter-residue distance plots of ttt-15mer-I and ttt-15mer-IV

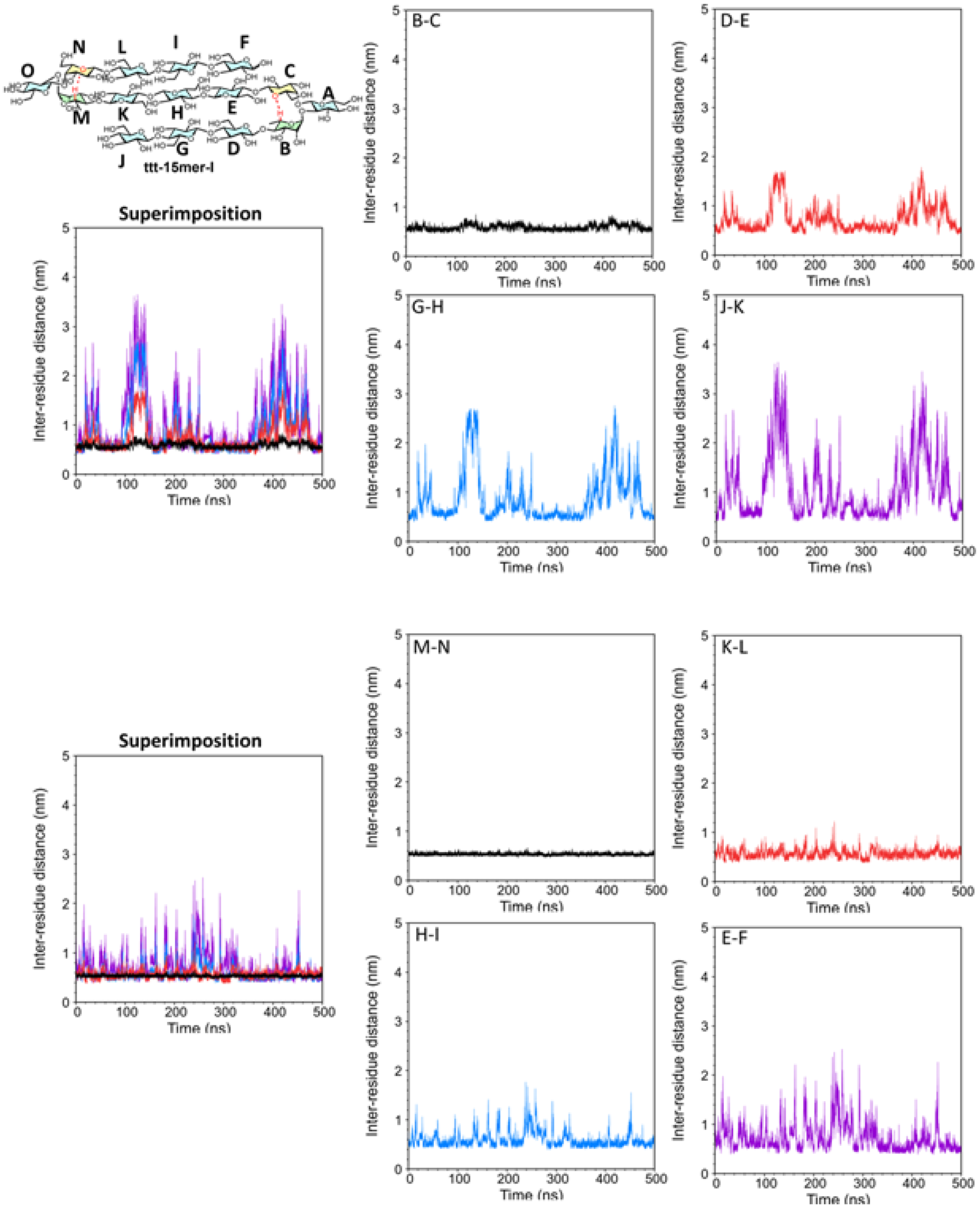

Figure S14 Inter-residue distance of **ttt-15mer-I** between center of mass of homologous residues in both strands.

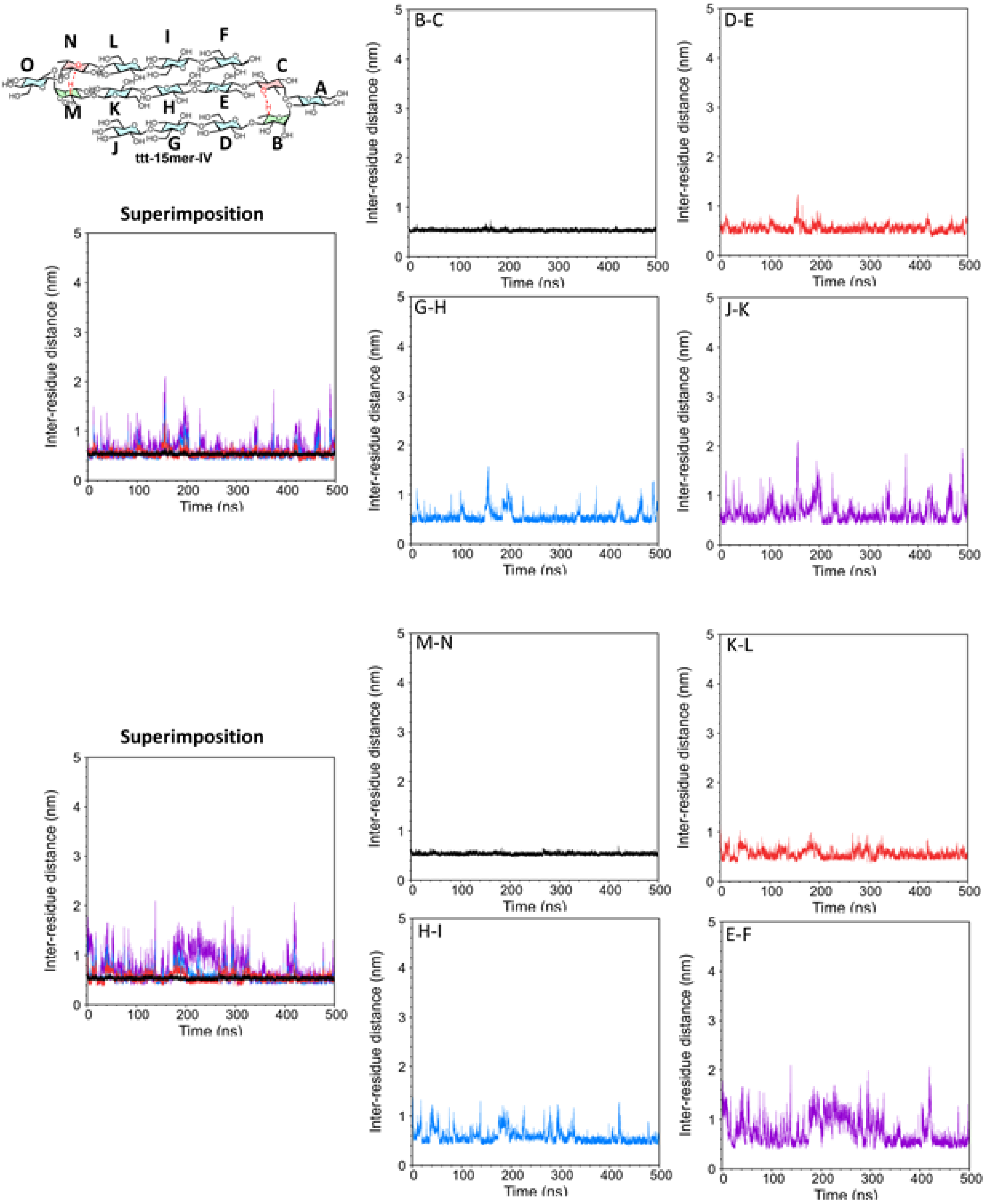

Figure S15 Inter-residue distance of **ttt-15mer-IV** between center of mass of homologous residues in both strands.

5.14 Ramachandran plots of ttt-15mer-I and ttt-15mer-IV

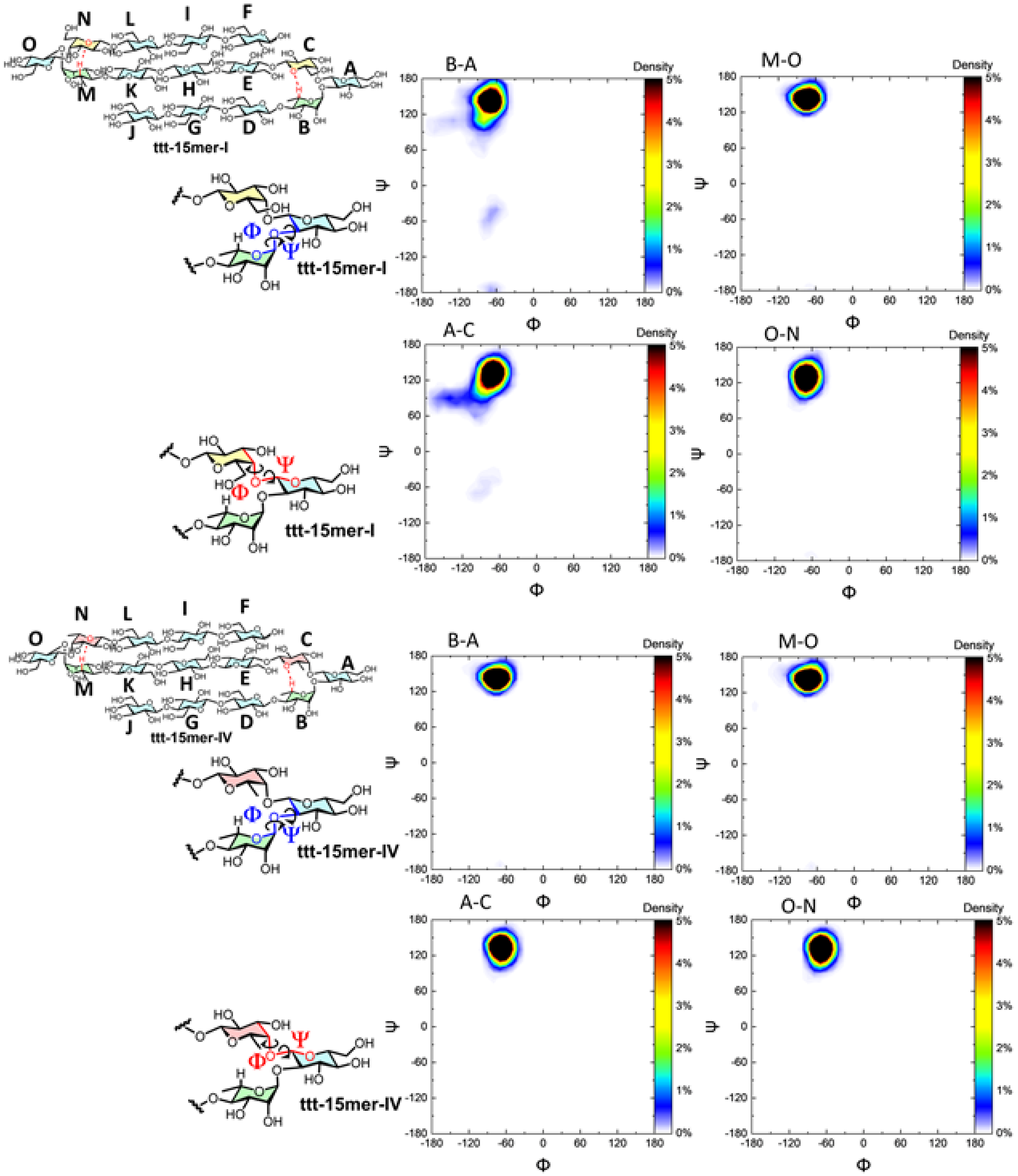

Figure S16 Ramachandran plot of **ttt-15mer-I** and **ttt-15mer-IV** on the turn unit (Φ: $O^{5'}$-$C^{1'}$-O^{n}-C^{n}, Ψ: $C^{1'}$-O^{n}-C^{n}-C^{n-1}).

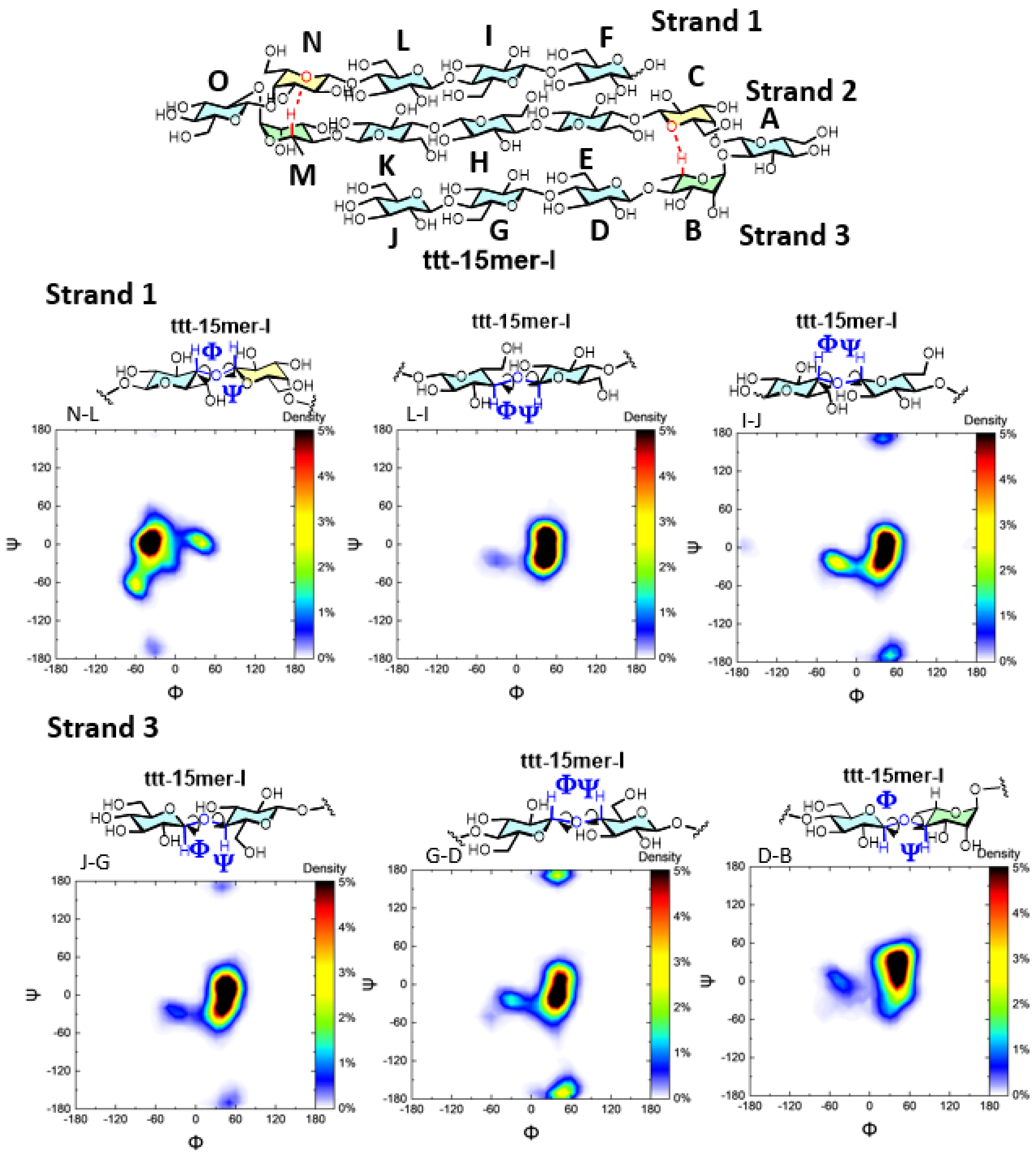

Figure S17 Ramachandran plot of **ttt-15mer-I** on the strands 1 and 3 (Φ: H$^{1'}$-C$^{1'}$-O^{4}-C^{4}, Ψ: C$^{1'}$-O^{4}-C^{4}-H^{4}).

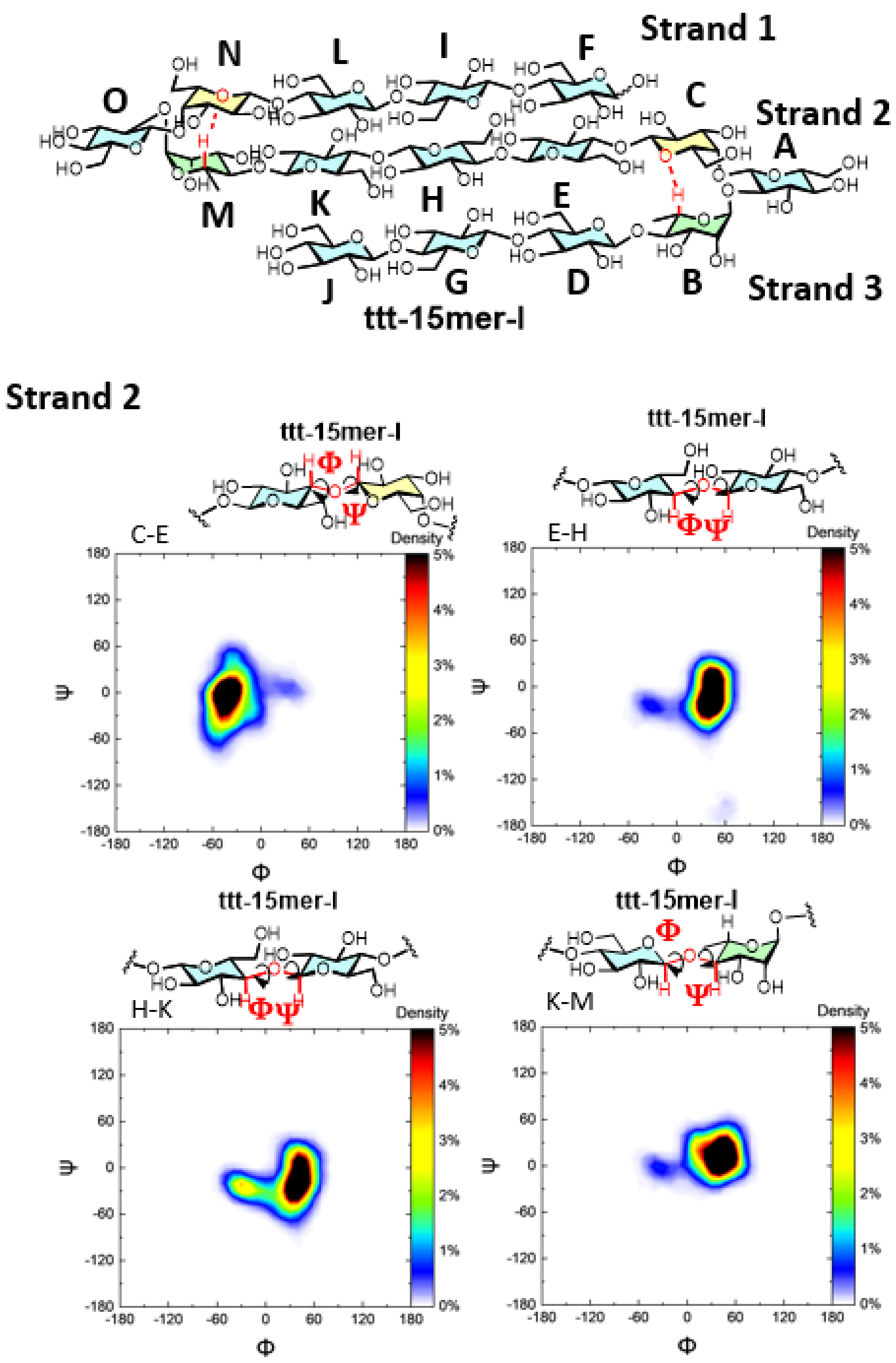

Figure S18 Ramachandran plot of **ttt-15mer-I** on the strand 2 (Φ: $H^{1'}$-$C^{1'}$-O^4-C^4, Ψ: $C^{1'}$-O^4-C^4-H^4).

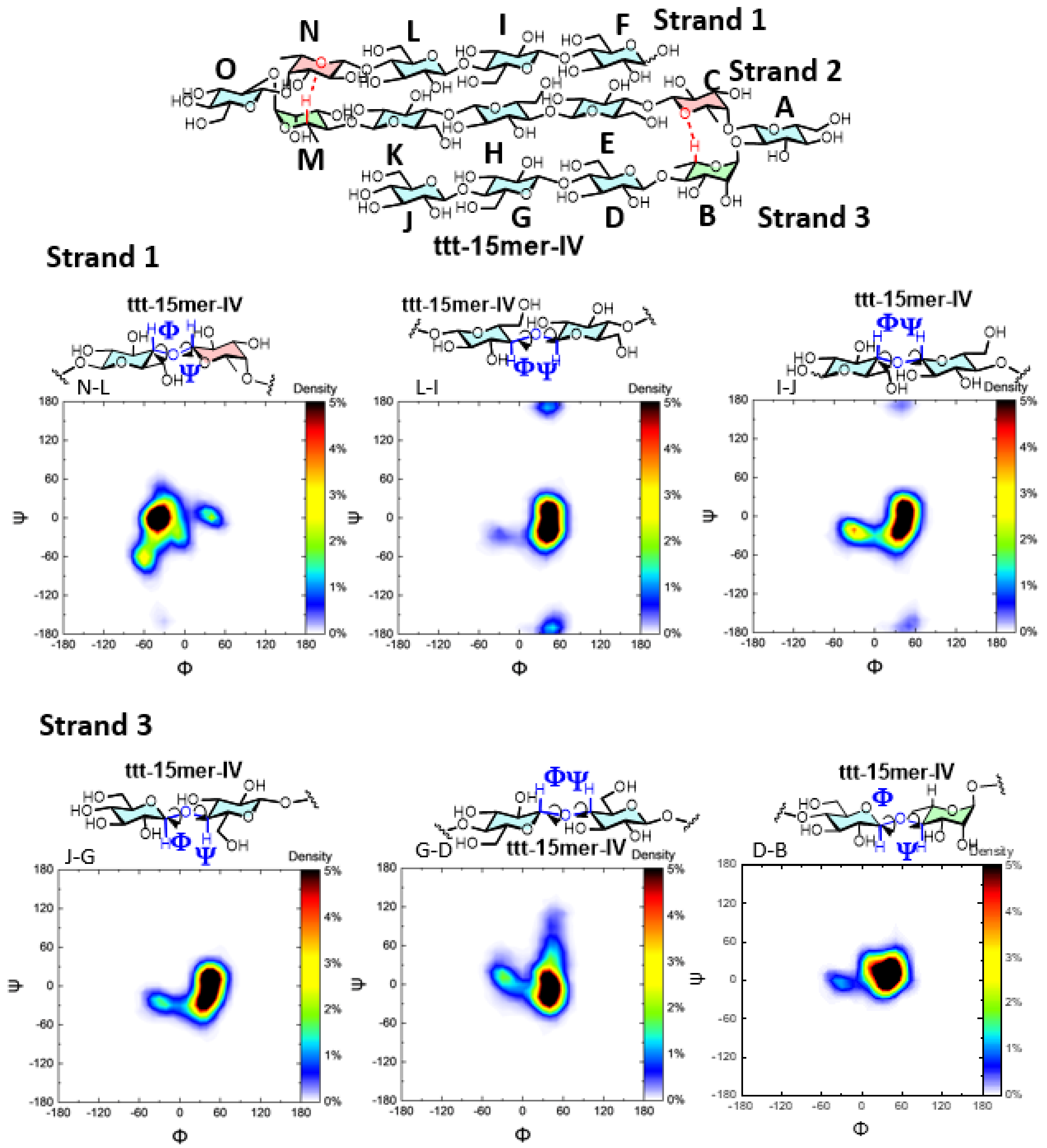

Figure S19 Ramachandran plot of **ttt-15mer-IV** on the strands 1 and 3 (Φ: $H^{1'}$-$C^{1'}$-O^4-C^4, Ψ: $C^{1'}$-O^4-C^4-H^4).

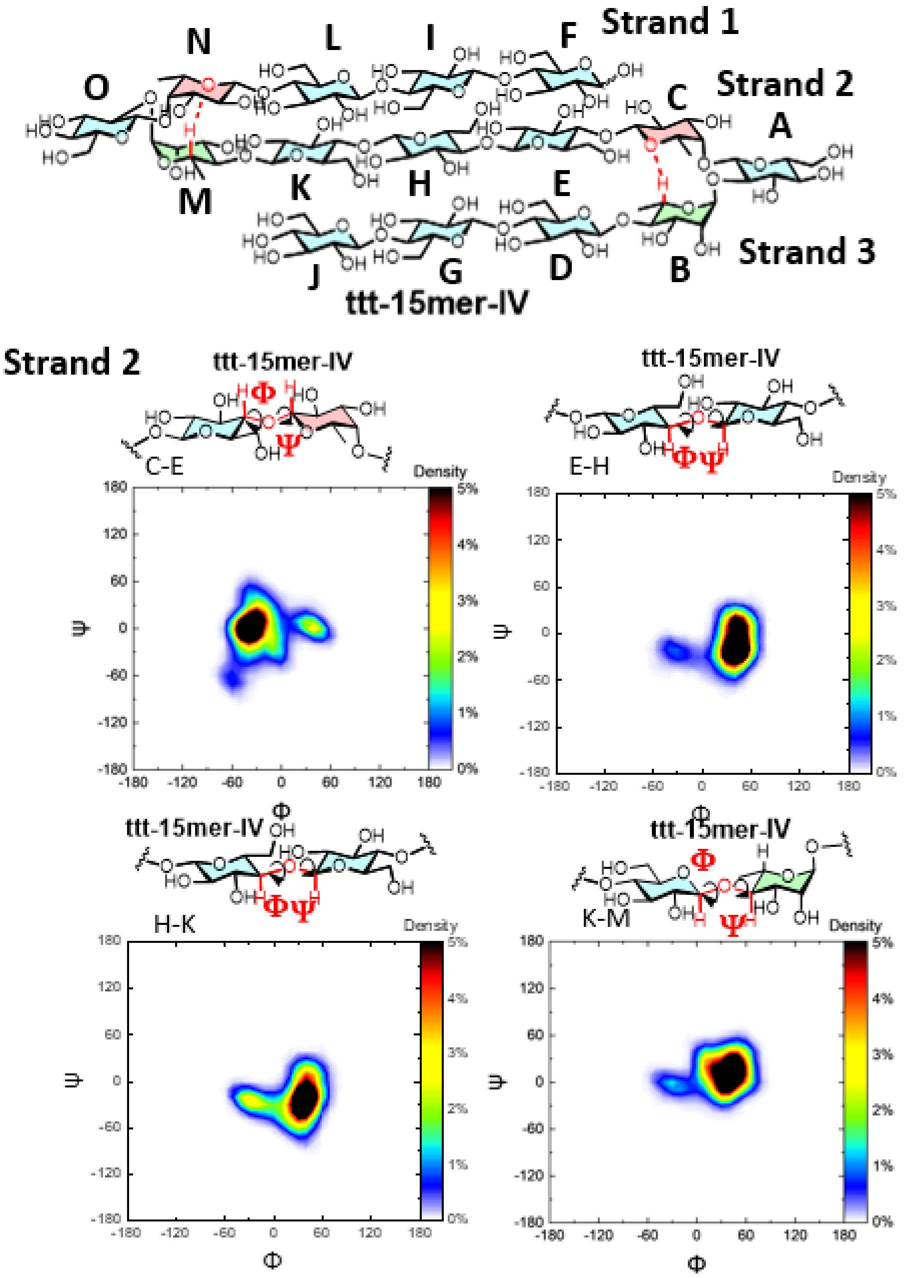

Figure S20 Ramachandran plot of **ttt-15mer-IV** on the strand 2 (Φ: H1'-C1'-O4-C4, Ψ: C1'-O4-C4-H4).

5.15 Inter-proton distances for a-3mer-II

Table S1. Average inter-proton distances and standard deviation (in nm) between **Rha B/Glc C** extracted from MD simulations. Predicted pair according to the MD simulation shown in bold. Cells highlighted in green represent experimentally observed NOEs**.**

		**Glc C**						
		**H-1**	**H-2**	**H-3**	**H-4**	**H-5**	**H-6**	**H-6'**
**Rha B**	**H-1**	0.83 ± 0.05	0.86 ± 0.04	0.66 ± 0.05	0.62 ± 0.04	0.60 ± 0.05	0.54 ± 0.09	0.59 ± 0.09
	**H-2**	0.88 ± 0.07	0.93 ± 0.04	0.76 ± 0.05	0.68 ± 0.03	0.63 ± 0.07	0.52 ± 0.11	0.55 ± 0.11
	**H-3**	0.69 ± 0.11	0.78 ± 0.06	0.63 ± 0.08	0.56 ± 0.05	0.46 ± 0.12	**0.36 ± 0.14**	**0.38 ± 0.14**
	**H-4**	0.76 ± 0.11	0.90 ± 0.06	0.69 ± 0.09	0.73 ± 0.04	0.56 ± 0.10	0.57 ± 0.10	0.57 ± 0.09
	**H-5**	0.53 ± 0.10	0.62 ± 0.06	0.42 ± 0.09	0.44 ± 0.04	**0.31 ± 0.11**	**0.34 ± 0.10**	**0.36 ± 0.10**
	**H-6**	0.60 ± 0.13	0.74 ± 0.11	0.50 ± 0.14	0.62 ± 0.09	0.52 ± 0.09	0.56 ± 0.11	0.56 ± 0.10

Table S2. Average inter-proton distances and standard deviation (in nm) between **Glc C/Glc A** extracted from MD simulations. Predicted pair according to the MD simulation shown in bold. Cells highlighted in green represent experimentally observed NOEs**.**

		**Glc A**						
		**H-1**	**H-2**	**H-3**	**H-4**	**H-5**	**H-6**	**H-6'**
**Glc C**	**H-1**	0.59 ± 0.02	0.68 ± 0.03	0.84 ± 0.03	0.87 ± 0.04	0.78 ± 0.04	0.86 ± 0.10	0.85 ± 0.10
	**H-2**	0.51 ± 0.02	0.67 ± 0.02	0.78 ± 0.03	0.77 ± 0.03	0.64 ± 0.04	0.69 ± 0.07	0.66 ± 0.09
	**H-3**	0.44 ± 0.03	0.47 ± 0.02	0.66 ± 0.03	0.63 ± 0.04	0.59 ± 0.05	0.64 ± 0.11	0.64 ± 0.10
	**H-4**	**0.24 ± 0.02**	0.44 ± 0.02	0.51 ± 0.03	0.56 ± 0.03	**0.41 ± 0.04**	0.53 ± 0.05	0.51 ± 0.07
	**H-5**	**0.39 ± 0.02**	0.48 ± 0.02	0.63 ± 0.03	0.70 ± 0.04	0.62 ± 0.04	0.75 ± 0.10	0.74 ± 0.09
	**H-6**	**0.29 ± 0.06**	0.46 ± 0.07	0.50 ± 0.07	0.65 ± 0.06	0.53 ± 0.07	0.71 ± 0.07	0.69 ± 0.07
	**H-6'**	**0.36 ± 0.08**	0.52 ± 0.09	0.57 ± 0.09	0.72 ± 0.09	0.60 ± 0.09	0.78 ± 0.10	0.76 ± 0.10

Table S3. Average inter-proton distances and standard deviation (in nm) between **Glc A/Rha B** extracted from MD simulations. Predicted pair according to the MD simulation shown in bold. Cells highlighted in green represent experimentally observed NOEs.

		Rha B					
		H-1	H-2	H-3	H-4	H-5	H-6b
Glc A	H-1	0.44 ± 0.02	0.49 ± 0.02	0.43 ± 0.03	0.64 ± 0.03	**0.38 ± 0.04**	0.60 ± 0.07
	H-2	**0.25 ± 0.02**	0.44 ± 0.02	0.45 ± 0.02	0.54 ± 0.02	**0.34 ± 0.04**	0.48 ± 0.07
	H-3	**0.34 ± 0.04**	**0.41 ± 0.03**	0.49 ± 0.03	0.68 ± 0.02	0.52 ± 0.02	0.71 ± 0.04
	H-4	0.46 ± 0.03	0.62 ± 0.03	0.69 ± 0.03	0.81 ± 0.02	0.62 ± 0.04	0.75 ± 0.08
	H-5	0.57 ± 0.02	0.64 ± 0.04	0.65 ± 0.04	0.86 ± 0.03	0.62 ± 0.04	0.82 ± 0.06
	H-6	0.70 ± 0.03	0.83 ± 0.04	0.85 ± 0.05	1.01 ± 0.05	0.76 ± 0.06	0.91 ± 0.10
	H-6’	0.72 ± 0.03	0.84 ± 0.04	0.85 ± 0.04	1.02 ± 0.03	0.77 ± 0.05	0.93 ± 0.08

5.16 Inter-proton distances for a-3mer-IV

Table S4. Average inter-proton distances and standard deviation (in nm) between **Rha B/Fuc C** extracted from MD simulations. Predicted pair according to the MD simulation shown in bold. Cells highlighted in green represent experimentally observed NOEs.

		Fuc C					
		H-1	H-2	H-3	H-4	H-5	H-6
Rha B	H-1	0.80 ± 0.05	0.62 ± 0.06	0.78 ± 0.05	0.62 ± 0.05	0.72 ± 0.05	0.57 ± 0.09
	H-2	0.85 ± 0.08	0.73 ± 0.07	0.85 ± 0.04	0.68 ± 0.03	0.74 ± 0.06	0.54 ± 0.10
	H-3	0.68 ± 0.14	0.61 ± 0.11	0.71 ± 0.07	0.57 ± 0.04	0.57 ± 0.10	**0.40 ± 0.13**
	H-4	0.74 ± 0.12	0.64 ± 0.14	0.83 ± 0.06	0.74 ± 0.04	0.73 ± 0.05	0.602 ± 0.091
	H-5	0.51 ± 0.12	**0.39 ± 0.14**	0.54 ± 0.07	0.45 ± 0.05	0.47 ± 0.07	**0.38 ± 0.09**
	H-6	0.59 ± 0.13	0.44 ± 0.18	0.66 ± 0.10	0.63 ± 0.09	0.63 ± 0.09	0.59 ± 0.11

Table S5. Average inter-proton distances and standard deviation (in nm) between **Fuc C/Glc A** extracted from MD simulations. Predicted pair according to the MD simulation shown in bold. Cells highlighted in green represent experimentally observed NOEs.

		Glc A						
		H-1	H-2	H-3	H-4	H-5	H-6	H-6’
Fuc C	H-1	0.58 ± 0.01	0.66 ± 0.02	0.83 ± 0.02	0.87 ± 0.03	0.79 ± 0.02	0.85 ± 0.12	0.87 ± 0.10
	H-2	0.48 ± 0.02	0.45 ± 0.03	0.67 ± 0.03	0.64 ± 0.05	0.64 ± 0.05	0.66 ± 0.15	0.69 ± 0.13
	H-3	0.46 ± 0.02	0.58 ± 0.02	0.73 ± 0.02	0.71 ± 0.03	0.61 ± 0.02	0.65 ± 0.08	0.65 ± 0.08
	H-4	**0.24 ± 0.02**	0.43 ± 0.02	0.52 ± 0.03	0.55 ± 0.03	**0.41 ± 0.04**	0.51 ± 0.05	0.51 ± 0.07
	H-5	**0.40 ± 0.02**	0.58 ± 0.03	0.68 ± 0.03	0.76 ± 0.02	0.62 ± 0.04	0.74 ± 0.07	0.73 ± 0.07
	H-6	**0.31 ± 0.07**	0.49 ± 0.09	0.53 ± 0.08	0.69 ± 0.07	0.56 ± 0.09	0.73 ± 0.09	0.72 ± 0.09

Table S6. Average inter-proton distances and standard deviation (in nm) between **Glc A/Rha B** extracted from MD simulations. Predicted pair according to the MD simulation shown in bold. Cells highlighted in green represent experimentally observed NOEs.

		Rha B					
		H-1	H-2	H-3	H-4	H-5	H-6
Glc A	H-1	0,44 ± 0,03	0,49 ± 0,02	0,43 ± 0,03	0,63 ± 0,04	**0,38 ± 0,05**	0,60 ± 0,08
	H-2	**0,26 ± 0,02**	0,43 ± 0,03	0,45 ± 0,02	0,54 ± 0,02	**0,35 ± 0,04**	0,48 ± 0,07
	H-3	**0,34 ± 0,04**	**0,40 ± 0,03**	0,49 ± 0,03	0,68 ± 0,03	0,52 ± 0,03	0,71 ± 0,05
	H-4	0,46 ± 0,03	0,62 ± 0,04	0,68 ± 0,04	0,81 ± 0,02	0,62 ± 0,04	0,75 ± 0,08
	H-5	0,57 ± 0,03	0,64 ± 0,04	0,65 ± 0,05	0,86 ± 0,04	0,62 ± 0,05	0,82 ± 0,07
	H-6	0,69 ± 0,04	0,82 ± 0,05	0,84 ± 0,05	1,00 ± 0,05	0,75 ± 0,07	0,90 ± 0,10
	H-6’	0,72 ± 0,03	0,83 ± 0,04	0,85 ± 0,05	1,02 ± 0,04	0,77 ± 0,06	0,93 ± 0,09

5.17 Inter-proton distances for a-5mer-IV

Table S7. Average inter-proton distances and standard deviation (in nm) between **Glc E/Glc D** extracted from MD simulations. Predicted pair according to the MD simulation shown in bold. Cells highlighted in green represent experimentally observed NOEs**.**

		Glc D						
		**H-1**	**H-2**	**H-3**	**H-4**	**H-5**	**H-6**	**H-6'**
**Glc E**	**H-1**	0.74 ± 0.22	0.55 ± 0.27	0.72 ± 0.28	0.50 ± 0.30	0.71 ± 0.25	0.60 ± 0.29	0.65 ± 0.27
	**H-2**	0.88 ± 0.16	0.67 ± 0.21	0.89 ± 0.21	0.68 ± 0.23	0.89 ± 0.18	0.79 ± 0.24	0.84 ± 0.21
	**H-3**	0.76 ± 0.20	0.60 ± 0.24	0.83 ± 0.24	0.63 ± 0.27	0.78 ± 0.23	0.68 ± 0.28	0.72 ± 0.26
	**H-4**	0.74 ± 0.12	0.54 ± 0.18	0.81 ± 0.16	0.66 ± 0.18	0.81 ± 0.14	0.77 ± 0.18	0.81 ± 0.16
	**H-5**	0.58 ± 0.20	**0.41 ± 0.24**	0.64 ± 0.23	0,48 ± 0.25	0.62 ± 0.21	0.58 ± 0.24	0.62 ± 0.22
	**H-6**	0.55 ± 0.16	**0.39 ± 0.19**	0.64 ± 0.18	0.55 ± 0.21	0.66 ± 0.17	0.68 ± 0.21	0.70 ± 0.19
	**H-6'**	0.54 ± 0.17	**0.36 ± 0.22**	0.60 ± 0.20	0.49 ± 0.23	0.63 ± 0.19	0.64 ± 0.22	0.67 ± 0.21

Table S8. Average inter-proton distances and standard deviation (in nm) between **Fuc C/Rha B** extracted from MD simulations. Predicted pair according to the MD simulation shown in bold. Cells highlighted in green represent experimentally observed NOEs**.**

		Rha B					
		**H-1**	**H-2**	**H-3**	**H-4**	**H-5**	**H-6**
**Fuc C**	**H-1**	0.81 ± 0.04	0.85 ± 0.05	0.67 ± 0.08	0.74 ± 0.06	0.49 ± 0.07	0.55 ± 0.10
	**H-2**	0.60 ± 0.05	0.71 ± 0.05	0.59 ± 0.07	0.61 ± 0.08	**0.36 ± 0.09**	**0.38 ± 0.13**
	**H-3**	0.77 ± 0.03	0.86 ± 0.03	0.72 ± 0.05	0.83 ± 0.04	0.54 ± 0.05	0.64 ± 0.09
	**H-4**	0.63 ± 0.03	0.70 ± 0.03	0.59 ± 0.04	0.76 ± 0.03	0.47 ± 0.04	0.64 ± 0.08
	**H-5**	0.73 ± 0.04	0.75 ± 0.04	0.58 ± 0.06	0.74 ± 0.04	0,47 ± 0.05	0.63 ± 0.08
	**H-6**	0.59 ± 0.09	0.56 ± 0.09	**0.40 ± 0.10**	0.62 ± 0.09	**0.38 ± 0.09**	0.60 ± 0.10

Table S9. Average inter-proton distances and standard deviation (in nm) between **Glc E/Fuc C** extracted from MD simulations. Predicted pair according to the MD simulation shown in bold. Cells highlighted in green represent experimentally observed NOEs**.**

		Fuc C					
		**H-1**	**H-2**	**H-3**	**H-4**	**H-5**	**H-6**
**Glc E**	**H-1**	0.58 ± 0.02	0.68 ± 0.03	0.83 ± 0.03	0.95 ± 0.03	0.79 ± 0.03	0.87 ± 0.08
	**H-2**	0.48 ± 0.03	0.65 ± 0.05	0.71 ± 0.04	0.87 ± 0.04	0.69 ± 0.06	0.82 ± 0.09
	**H-3**	**0.39 ± 0.05**	0.45 ± 0.03	0.60 ± 0.04	0.75 ± 0.03	0.62 ± 0.05	0.74 ± 0.05
	**H-4**	**0.23 ± 0.03**	0.43 ± 0.03	0.49 ± 0.03	0.61 ± 0.02	0.43 ± 0.04	0.56 ± 0.08
	**H-5**	0.430 ± 0.03	0.492 ± 0.02	0.66 ± 0.02	0.75 ± 0.04	0.60 ± 0.04	0.65 ± 0.09
	**H-6**	**0.38 ± 0.07**	0.48 ± 0.05	0.59 ± 0.06	0.62 ± 0.07	0.46 ± 0.08	0.48 ± 0.12
	**H-6’**	0.46 ± 0.07	0.56 ± 0.09	0.68 ± 0.08	0.72 ± 0.08	0.56 ± 0.08	0.57 ± 0.11

Table S10. Average inter-proton distances and standard deviation (in nm) between **Fuc C/Glc A** extracted from MD simulations. Predicted pair according to the MD simulation shown in bold. Cells highlighted in green represent experimentally observed NOEs**.**

		Glc A						
		**H-1**	**H-2**	**H-3**	**H-4**	**H-5**	**H-6**	**H-6’**
**Fuc C**	**H-1**	0.58 ± 0.01	0.67 ± 0.02	0.83 ± 0.02	0.87 ± 0.02	0.78 ± 0.02	0.87 ± 0.08	0.88 ± 0.07
	**H-2**	0.48 ± 0.02	0.44 ± 0.03	0.68 ± 0.02	0.65 ± 0.04	0.66 ± 0.03	0.73 ± 0.08	0.71 ± 0.10
	**H-3**	0.47 ± 0.01	0.58 ± 0.01	0.72 ± 0.02	0.70 ± 0.03	0.60 ± 0.02	0.65 ± 0.08	0.66 ± 0.07
	**H-4**	**0.25 ± 0.02**	0.44 ± 0.01	0.51 ± 0.02	0.54 ± 0.02	**0.39 ± 0.03**	0.49 ± 0.07	0.51 ± 0.04
	**H-5**	**0.40 ± 0.02**	0.59 ± 0.02	0.67 ± 0.02	0.75 ± 0.02	0.60 ± 0.03	0.72 ± 0.07	0.74 ± 0.05
	**H-6**	**0.30 ± 0.07**	0.51 ± 0.07	0.52 ± 0.07	0.69 ± 0.07	0.53 ± 0.08	0.71 ± 0.09	0.74 ± 0.08

Table S11. Average inter-proton distances and standard deviation (in nm) between **Glc A/Rha B** extracted from MD simulations. Predicted pair according to the MD simulation shown in bold. Cells highlighted in green represent experimentally observed NOEs**.**

		Rha B					
		**H-1**	**H-2**	**H-3**	**H-4**	**H-5**	**H-6**
**Glc A**	**H-1**	0.45 ± 0.02	0.49 ± 0.02	0.44 ± 0.03	0.65 ± 0.03	**0.40 ± 0.03**	0.62 ± 0.06
	**H-2**	**0.25 ± 0.02**	0.43 ± 0.03	0.46 ± 0.02	0.54 ± 0.02	**0.36 ± 0.03**	0.49 ± 0.06
	**H-3**	**0.35 ± 0.03**	**0.40 ± 0.02**	0.49 ± 0.03	0.68 ± 0.022	0.53 ± 0.02	0.72 ± 0.04
	**H-4**	0.45 ± 0.02	0.61 ± 0.03	0.69 ± 0.03	0.81 ± 0.02	0.63 ± 0.03	0.76 ± 0.07
	**H-5**	0.57 ± 0.02	0.64 ± 0.03	0.66 ± 0.04	0.86 ± 0.02	0.63 ± 0.03	0.83 ± 0.06
	**H-6**	0.72 ± 0.02	0.84 ± 0.03	0.87 ± 0.04	1.03 ± 0.03	0.80 ± 0.04	0.95 ± 0.08
	**H-6’**	0.69 ± 0.03	0.83 ± 0.03	0.86 ± 0.04	1.01 ± 0.04	0.78 ± 0.05	0.92 ± 0.09

Table S12. Average inter-proton distances and standard deviation (in nm) between **Rha B**/**Glc D** extracted from MD simulations. Predicted pair according to the MD simulation shown in bold. Cells highlighted in green represent experimentally observed NOEs**.**

		Glc D						
		**H-1**	**H-2**	**H-3**	**H-4**	**H-5**	**H-6**	**H-6’**
**Rha B**	**H-1**	0.62 ± 0.01	0.78 ± 0.01	0.89 ± 0.02	0.94 ± 0.02	0.79 ± 0.03	0.90 ± 0.06	0.87 ± 0.09
	**H-2**	0.52 ± 0.02	0.70 ± 0.02	0.77 ± 0.03	0.88 ± 0.02	0.73 ± 0.04	0.90 ± 0.05	0.87 ± 0.08
	**H-3**	**0.38 ± 0.03**	0.48 ± 0.02	0.62 ± 0.03	0.71 ± 0.02	0.62 ± 0.03	0.77 ± 0.04	0.76 ± 0.06
	**H-4**	**0.24 ± 0.02**	0.44 ± 0.01	0.51 ± 0.02	0.55 ± 0.02	**0.40 ± 0.02**	0.53 ± 0.05	0.51 ± 0.07
	**H-5**	0.45 ± 0.01	0.48 ± 0.02	0.68 ± 0.01	0.66 ± 0.03	0.62 ± 0.02	0.69 ± 0.07	0.69 ± 0.07
	**H-6**	0.47 ± 0.07	0.51 ± 0.07	0.68 ± 0.07	0.58 ± 0.08	0.54 ± 0.08	0.54 ± 0.11	0.53 ± 0.12

5.18 Inter-proton distances for a-9mer-IV

Table S13. Average inter-proton distances and standard deviation (in nm) between **Glc I/Glc G** extracted from MD simulations. Predicted pair according to the MD simulation shown in bold. Cells highlighted in green represent experimentally observed NOEs**.**

		**Glc G**						
		**H-1**	**H-2**	**H-3**	**H-4**	**H-5**	**H-6**	**H-6'**
**Glc I**	**H-1**	0.58 ± 0.05	0.69 ± 0.03	0.84 ± 0.06	0.86 ± 0.05	0.76 ± 0.08	0.86 ± 0.11	0.82 ± 0.12
	**H-2**	0.50 ± 0.04	0.67 ± 0.03	0.77 ± 0.05	0.79 ± 0.04	0.64 ± 0.05	0.74 ± 0.09	0.69 ± 0.11
	**H-3**	**0.40 ± 0.07**	0.48 ± 0.03	0.64 ± 0.06	0.62 ± 0.04	0.56 ± 0.07	0.63 ± 0.10	0.61 ± 0.09
	**H-4**	**0.25 ± 0.04**	0.44 ± 0.03	0.52 ± 0.05	0.58 ± 0.04	0.44 ± 0.06	0.59 ± 0.07	0.56 ± 0.09
	**H-5**	0.41 ± 0.05	0.47 ± 0.03	0.64 ± 0.05	0.68 ± 0.06	0.62 ± 0.09	0.74 ± 0.12	0.71 ± 0.12
	**H-6**	**0.35 ± 0.09**	0.45 ± 0.08	0.55 ± 0.08	0.66 ± 0.07	0.58 ± 0.08	0.74 ± 0.10	0.72 ± 0.10
	**H-6'**	**0.40 ± 0.09**	0.48 ± 0.11	0.60 ± 0.09	0.70 ± 0.11	0.62 ± 0.10	0.78 ± 0.13	0.76 ± 0.13

Table S14. Average inter-proton distances and standard deviation (in nm) between **Glc G/Glc E** extracted from MD simulations. Predicted pair according to the MD simulation shown in bold. Cells highlighted in green represent experimentally observed NOEs**.**

		**Glc E**						
		**H-1**	**H-2**	**H-3**	**H-4**	**H-5**	**H-6**	**H-6'**
**GlcG**	**H-1**	0.58 ± 0.05	0.68 ± 0.03	0.84 ± 0.05	0.86 ± 0.05	0.77 ± 0.07	0.89 ± 0.09	0.80 ± 0.09
	**H-2**	0.51 ± 0.03	0.67 ± 0.02	0.78 ± 0.03	0.78 ± 0.04	0.63 ± 0.05	0.73 ± 0.07	0.63 ± 0.10
	**H-3**	0.42 ± 0.06	0.47 ± 0.03	0.65 ± 0.06	0.62 ± 0.05	0.57 ± 0.07	0.66 ± 0.08	0.58 ± 0.09
	**H-4**	**0.25 ± 0.04**	0.45 ± 0.02	0.52 ± 0.04	0.58 ± 0.04	0.42 ± 0.05	0.58 ± 0.05	0.50 ± 0.08
	**H-5**	**0.40 ± 0.05**	0.48 ± 0.03	0.63 ± 0.05	0.69 ± 0.05	0.62 ± 0.08	0.77 ± 0.10	0.70 ± 0.09
	**H-6**	**0.33 ± 0.07**	0.48 ± 0.08	0.54 ± 0.09	0.68 ± 0.08	0.56 ± 0.08	0.75 ± 0.11	0.70 ± 0.09
	**H-6'**	**0.38 ± 0.09**	0.52 ± 0.09	0.59 ± 0.10	0.72 ± 0.09	0.61 ± 0.09	0.80 ± 0.12	0.75 ± 0.11

Table S15. Average inter-proton distances and standard deviation (in nm) between **Glc E/Fuc C** extracted from MD simulations. Predicted pair according to the MD simulation shown in bold. Cells highlighted in green represent experimentally observed NOEs**.**

		Fuc C					
		H-1	H-2	H-3	H-4	H-5	H-6
Glc E	H-1	0.58 ± 0.03	0.68 ± 0.04	0.83 ± 0.04	0.95 ± 0.03	0.79 ± 0.04	0.87 ± 0.07
	H-2	0.48 ± 0.03	0.66 ± 0.05	0.72 ± 0.04	0.87 ± 0.04	0.69 ± 0.06	0.82 ± 0.09
	H-3	0.39 ± 0.05	0.46 ± 0.04	0.60 ± 0.05	0.75 ± 0.04	0.62 ± 0.06	0.75 ± 0.06
	H-4	0.24 ± 0.03	0.44 ± 0.03	0.49 ± 0.03	0.61 ± 0.02	0.43 ± 0.04	0.55 ± 0.07
	H-5	0.43 ± 0.03	0.49 ± 0.03	0.66 ± 0.03	0.75 ± 0.04	0.60 ± 0.04	0.65 ± 0.08
	H-6	0.39 ± 0.07	0.48 ± 0.06	0.60 ± 0.07	0.62 ± 0.08	0.47 ± 0.09	0.47 ± 0.12
	H-6’	0.48 ± 0.07	0.57 ± 0.08	0.70 ± 0.07	0.73 ± 0.07	0.57 ± 0.07	0.56 ± 0.11

Table S16. Average inter-proton distances and standard deviation (in nm) between **Fuc C/Glc A** extracted from MD simulations. Predicted pair according to the MD simulation shown in bold. Cells highlighted in green represent experimentally observed NOEs**.**

		Glc A						
		H-1	H-2	H-3	H-4	H-5	H-6	H-6’
Fuc C	H-1	0.59 ± 0.02	0.67 ± 0.02	0.84 ± 0.02	0.87 ± 0.03	0.79 ± 0.02	0.89 ± 0.06	0.88 ± 0.08
	H-2	0.49 ± 0.02	0.44 ± 0.03	0.68 ± 0.03	0.65 ± 0.04	0.67 ± 0.03	0.73 ± 0.08	0.74 ± 0.07
	H-3	0.47 ± 0.02	0.58 ± 0.02	0.73 ± 0.02	0.71 ± 0.03	0.61 ± 0.03	0.67 ± 0.07	0.66 ± 0.08
	H-4	**0.25 ± 0.02**	0.44 ± 0.01	0.52 ± 0.02	0.54 ± 0.02	**0.39 ± 0.02**	0.52 ± 0.05	0.49 ± 0.07
	H-5	0.41 ± 0.02	0.60 ± 0.01	0.68 ± 0.03	0.76 ± 0.02	0.60 ± 0.03	0.75 ± 0.04	0.73 ± 0.08
	H-6	**0.31 ± 0.07**	0.52 ± 0.07	0.53 ± 0.08	0.70 ± 0.07	0.53 ± 0.08	0.74 ± 0.08	0.72 ± 0.10

Table S17. Average inter-proton distances and standard deviation (in nm) between **Glc A/Rha B** extracted from MD simulations. Predicted pair according to the MD simulation shown in bold. Cells highlighted in green represent experimentally observed NOEs.

		Rha B					
		**H-1**	**H-2**	**H-3**	**H-4**	**H-5**	**H-6**
**Glc A**	**H-1**	0.45 ± 0.01	0.49 ± 0.02	0.45 ± 0.03	0.66 ± 0.02	0.41 ± 0.03	0.63 ± 0.05
	**H-2**	**0.25 ± 0.02**	0.44 ± 0.01	0.47 ± 0.02	0.55 ± 0.02	**0.37 ± 0.03**	0.49 ± 0.06
	**H-3**	**0.35 ± 0.03**	**0.40 ± 0.03**	0.49 ± 0.03	0.68 ± 0.02	0.53 ± 0.02	0.73 ± 0.04
	**H-4**	0.45 ± 0.02	0.62 ± 0.03	0.70 ± 0.02	0.81 ± 0.02	0.64 ± 0.03	0.76 ± 0.07
	**H-5**	0.57 ± 0.02	0.65 ± 0.03	0.67 ± 0.03	0.87 ± 0.02	0.64 ± 0.03	0.84 ± 0.06
	**H-6**	0.70 ± 0.03	0.84 ± 0.03	0.87 ± 0.03	1.02 ± 0.04	0.80 ± 0.05	0.93 ± 0.09
	**H-6’**	0.73 ± 0.02	0.85 ± 0.03	0.87 ± 0.03	1.04 ± 0.02	0.81 ± 0.04	0.96 ± 0.08

Table S18. Average inter-proton distances and standard deviation (in nm) between **Rha B/Glc D** extracted from MD simulations. Predicted pair according to the MD simulation shown in bold. Cells highlighted in green represent experimentally observed NOEs.

		Glc D						
		**H-1**	**H-2**	**H-3**	**H-4**	**H-5**	**H-6**	**H-6’**
**Rha B**	**H-1**	0.62 ± 0.02	0.79 ± 0.02	0.89 ± 0.03	0.95 ± 0.02	0.80 ± 0.04	0.93 ± 0.07	0.88 ± 0.10
	**H-2**	0.52 ± 0.03	0.69 ± 0.04	0.76 ± 0.04	0.89 ± 0.02	0.74 ± 0.04	0.92 ± 0.05	0.88 ± 0.08
	**H-3**	**0.39 ± 0.03**	0.48 ± 0.03	0.62 ± 0.04	0.71 ± 0.03	0.63 ± 0.03	0.79 ± 0.05	0.76 ± 0.06
	**H-4**	**0.24 ± 0.03**	0.44 ± 0.02	0.51 ± 0.03	0.56 ± 0.03	**0.40 ± 0.03**	0.56 ± 0.06	0.52 ± 0.08
	**H-5**	0.45 ± 0.02	0.49 ± 0.03	0.69 ± 0.02	0.67 ± 0.03	0.62 ± 0.03	0.71 ± 0.07	0.69 ± 0.08
	**H-6**	0.46 ± 0.07	0.53 ± 0.08	0.68 ± 0.08	0.60 ± 0.09	0.54 ± 0.09	0.56 ± 0.11	0.54 ± 0.12

Table S19. Average inter-proton distances and standard deviation (in nm) between **Glc D/Glc F** extracted from MD simulations. Predicted pair according to the MD simulation shown in bold. Cells highlighted in green represent experimentally observed NOEs**.**

		Glc F						
		**H-1**	**H-2**	**H-3**	**H-4**	**H-5**	**H-6**	**H-6'**
**GlcD**	**H-1**	0.59 ± 0.02	0.68 ± 0.03	0.85 ± 0.02	0.85 ± 0.04	0.78 ± 0.03	0.88 ± 0.07	0.82 ± 0.08
	**H-2**	0.50 ± 0.04	0.68 ± 0.02	0.76 ± 0.04	0.78 ± 0.03	0.63 ± 0.04	0.74 ± 0.07	0.66 ± 0.10
	**H-3**	0.42 ± 0.04	0.47 ± 0.03	0.65 ± 0.03	0.60 ± 0.05	0.56 ± 0.06	0.63 ± 0.09	0.58 ± 0.09
	**H-4**	**0.24 ± 0.02**	0.44 ± 0.03	0.50 ± 0.03	0.58 ± 0.03	0.43 ± 0.04	0.60 ± 0.06	0.54 ± 0.09
	**H-5**	0.42 ± 0.03	0.47 ± 0.03	0.66 ± 0.03	0.69 ± 0.03	0.65 ± 0.02	0.78 ± 0.06	0.74 ± 0.06
	**H-6**	**0.35 ± 0.07**	0.46 ± 0.08	0.55 ± 0.07	0.68 ± 0.06	0.60 ± 0.07	0.79 ± 0.06	0.75 ± 0.08
	**H-6'**	0.41 ± 0.10	0.49 ± 0.10	0.60 ± 0.10	0.72 ± 0.10	0.65 ± 0.10	0.83 ± 0.09	0.80 ± 0.09

Table S20. Average inter-proton distances and standard deviation (in nm) between **Glc F/Glc H** extracted from MD simulations. Predicted pair according to the MD simulation shown in bold. Cells highlighted in green represent experimentally observed NOEs**.**

		Glc H						
		**H-1**	**H-2**	**H-3**	**H-4**	**H-5**	**H-6**	**H-6'**
**GlcF**	**H-1**	0.59 ± 0.03	0.69 ± 0.03	0.85 ± 0.03	0.87 ± 0.04	0.78 ± 0.05	0.87 ± 0.08	0.86 ± 0.09
	**H-2**	0.50 ± 0.03	0.67 ± 0.02	0.77 ± 0.04	0.78 ± 0.04	0.62 ± 0.04	0.71 ± 0.08	0.69 ± 0.10
	**H-3**	0.42 ± 0.04	0.48 ± 0.03	0.66 ± 0.04	0.62 ± 0.05	0.57 ± 0.06	0.63 ± 0.09	0.64 ± 0.09
	**H-4**	**0.24 ± 0.03**	0.44 ± 0.03	0.51 ± 0.03	0.57 ± 0.03	0.42 ± 0.04	0.57 ± 0.06	0.55 ± 0.09
	**H-5**	0.41 ± 0.03	0.48 ± 0.03	0.65 ± 0.03	0.70 ± 0.04	0.64 ± 0.04	0.76 ± 0.07	0.76 ± 0.07
	**H-6**	**0.33 ± 0.07**	0.45 ± 0.07	0.52 ± 0.07	0.66 ± 0.06	0.56 ± 0.08	0.75 ± 0.07	0.74 ± 0.09
	**H-6'**	0.41 ± 0.09	0.52 ± 0.10	0.61 ± 0.09	0.74 ± 0.09	0.64 ± 0.09	0.82 ± 0.09	0.81 ± 0.10

Table S21. Average inter-proton distances and standard deviation (in nm) between **Glc I/Glc H** extracted from MD simulations. Predicted pair according to the MD simulation shown in bold. Cells highlighted in green represent experimentally observed NOEs**.**

		Glc H						
		**H-1**	**H-2**	**H-3**	**H-4**	**H-5**	**H-6**	**H-6'**
**GlcI**	**H-1**	0.71 ± 0.27	0.53 ± 0.31	0.73 ± 0.30	0.63 ± 0.33	0.77 ± 0.29	0.76 ± 0.34	0.80 ± 0.32
	**H-2**	0.81 ± 0.20	0.65 ± 0.23	0.86 ± 0.22	0.75 ± 0.26	0.89 ± 0.22	0.85 ± 0.30	0.90 ± 0.27
	**H-3**	0.64 ± 0.23	0.54 ± 0.24	0.76 ± 0.23	0.68 ± 0.27	0.76 ± 0.25	0.77 ± 0.30	0.80 ± 0.28
	**H-4**	0.74 ± 0.17	0.62 ± 0.21	0.85 ± 0.19	0.79 ± 0.23	0.88 ± 0.19	0.90 ± 0.26	0.94 ± 0.22
	**H-5**	0.63 ± 0.25	0.50 ± 0.28	0.71 ± 0.27	0.68 ± 0.28	0.77 ± 0.26	0.81 ± 0.29	0.84 ± 0.26
	**H-6**	0.73 ± 0.23	0.61 ± 0.27	0.82 ± 0.25	0.80 ± 0.27	0.88 ± 0.23	0.93 ± 0.28	0.96 ± 0.26
	**H-6'**	0.73 ± 0.24	0.60 ± 0.28	0.80 ± 0.27	0.79 ± 0.29	0.87 ± 0.25	0.93 ± 0.28	0.96 ± 0.26

Table S22. Average inter-proton distances and standard deviation (in nm) between **Glc G/Glc F** extracted from MD simulations. Predicted pair according to the MD simulation shown in bold. Cells highlighted in green represent experimentally observed NOEs**.**

		Glc F						
		**H-1**	**H-2**	**H-3**	**H-4**	**H-5**	**H-6**	**H-6'**
**GlcG**	**H-1**	0.58 ± 0.16	0.79 ± 0.14	0.58 ± 0.19	0.75 ± 0.13	0.52 ± 0.16	0.67 ± 0.17	0.64 ± 0.16
	**H-2**	0.42 ± 0.20	0.65 ± 0.18	0.50 ± 0.20	0.62 ± 0.17	**0.39 ± 0.19**	0.55 ± 0.20	0.50 ± 0.21
	**H-3**	0.57 ± 0.15	0.82 ± 0.13	0.68 ± 0.17	0.82 ± 0.13	0.56 ± 0.15	0.72 ± 0.17	0.66 ± 0.17
	**H-4**	0.43 ± 0.16	0.69 ± 0.15	0.58 ± 0.16	0.74 ± 0.13	0.51 ± 0.14	0.70 ± 0.15	0.64 ± 0.17
	**H-5**	0.58 ± 0.14	0.82 ± 0.14	0.64 ± 0.16	0.83 ± 0.12	0.59 ± 0.13	0.78 ± 0.16	0.73 ± 0.16
	**H-6**	0.56 ± 0.20	0.77 ± 0.21	0.64 ± 0.21	0.84 ± 0.18	0.63 ± 0.18	0.82 ± 0.20	0.78 ± 0.21
	**H-6'**	0.58 ± 0.19	0.79 ± 0.20	0.64 ± 0.20	0.85 ± 0.17	0.65 ± 0.17	0.84 ± 0.19	0.80 ± 0.20

Table S23. Average inter-proton distances and standard deviation (in nm) between **Glc E/Glc D** extracted from MD simulations. Predicted pair according to the MD simulation shown in bold. Cells highlighted in green represent experimentally observed NOEs.

		Glc D						
		**H-1**	**H-2**	**H-3**	**H-4**	**H-5**	**H-6**	**H-6'**
**GlcE**	**H-1**	0.70 ± 0.12	0.53 ± 0.14	0.71 ± 0.14	0.46 ± 0.14	0.66 ± 0.14	0.52 ± 0.18	0.56 ± 0.17
	**H-2**	0.86 ± 0.09	0.65 ± 0.11	0.89 ± 0.11	0.66 ± 0.10	0.86 ± 0.10	0.75 ± 0.16	0.79 ± 0.14
	**H-3**	0.75 ± 0.12	0.62 ± 0.12	0.84 ± 0.12	0.63 ± 0.13	0.76 ± 0.15	0.65 ± 0.19	0.67 ± 0.18
	**H-4**	0.72 ± 0.07	0.53 ± 0.09	0.80 ± 0.09	0.64 ± 0.09	0.79 ± 0.08	0.74 ± 0.15	0.76 ± 0.11
	**H-5**	0.54 ± 0.11	**0.39 ± 0.11**	0.62 ± 0.11	0.44 ± 0.10	0.56 ± 0.11	0.51 ± 0.15	0.53 ± 0.13
	**H-6**	0.50 ± 0.08	**0.35 ± 0.08**	0.61 ± 0.08	0.50 ± 0.11	0.61 ± 0.10	0.62 ± 0.15	0.64 ± 0.13
	**H-6'**	0.48 ± 0.09	**0.29 ± 0.10**	0.54 ± 0.09	0.43 ± 0.12	0.56 ± 0.09	0.57 ± 0.15	0.60 ± 0.12

Table S24. Average inter-proton distances and standard deviation (in nm) between **Fuc C/Rha B** extracted from MD simulations. Predicted pair according to the MD simulation shown in bold. Cells highlighted in green represent experimentally observed NOEs.

		Rha B					
		**H-1**	**H-2**	**H-3**	**H-4**	**H-5**	**H-6**
**Fuc C**	**H-1**	0.81 ± 0.03	0.86 ± 0.04	0.67 ± 0.05	0.73 ± 0.04	0.48 ± 0.03	0.54 ± 0.09
	**H-2**	0.60 ± 0.04	0.72 ± 0.04	0.59 ± 0.05	0.60 ± 0.05	**0.35 ± 0.05**	**0.37 ± 0.09**
	**H-3**	0.78 ± 0.02	0.87 ± 0.02	0.72 ± 0.04	0.83 ± 0.03	0.54 ± 0.03	0.64 ± 0.08
	**H-4**	0.64 ± 0.02	0.70 ± 0.03	0.59 ± 0.04	0.77 ± 0.03	0.47 ± 0.03	0.65 ± 0.07
	**H-5**	0.74 ± 0.02	0.76 ± 0.04	0.58 ± 0.05	0.75 ± 0.04	0.46 ± 0.04	0.63 ± 0.08
	**H-6**	0.60 ± 0.08	0.56 ± 0.09	**0.39 ± 0.09**	0.62 ± 0.10	**0.38 ± 0.09**	0.61 ± 0.10

6 NMR analysis

6.1 General materials and methods

^{1}H, ^{13}C, HSQC, 1D and 2D TOCSY, 1D and 2D ROESY, 1D and 2D NOESY NMR spectra were recorded on a Varian 400-MR (400 MHz), Varian 600-NMR (600 MHz), Bruker Biospin AVANCE700 (700 MHz) spectrometer. Samples were prepared by dissolving lyophilized oligomers in D_2O (concentration ≈ 1 - 6 mM). Proton resonances of the oligosaccharides were assigned using a combination of ^{1}H, 2D COSY, HSQC, 1D and 2D TOCSY. Selective 1D TOCSY (HOHAHA, pulse program: seldigpzs) spectra were recorded using different mixing times to assign all the resonances (d9 = 40, 80, 120, 160, and 200 ms). 2D TOCSY (pulse program: mlevphpp) spectra were recorded using different mixing times (d9 = 80, or 150 ms). Selective 1D t-ROESY (pulse program: selrogp.2) spectra were recorded using different mixing times (p15 = 200, or 300 ms). 2D t-ROESY (pulse program: reosyph.2) and 2D NOESY (pulse program: noesygpphpp) spectra were recorded using different mixing times (p15 = 200, or 300 ms for ROESY and d8 = 600, 800, or 1000 ms for NOESY). Monosaccharide were named as follows: D-glucose (Glc), L-fucose (Fuc), L-rhamnose (Rha), L-Galactose (Gal). Labelling of protons in a monosaccharide is done as follows: e.g. proton attached to B-1 of Rha is named "Rha B-1". Resonances of residues at the reducing end are additionally labelled with α or β.

6.2 NMR characterization of a-3mer-II

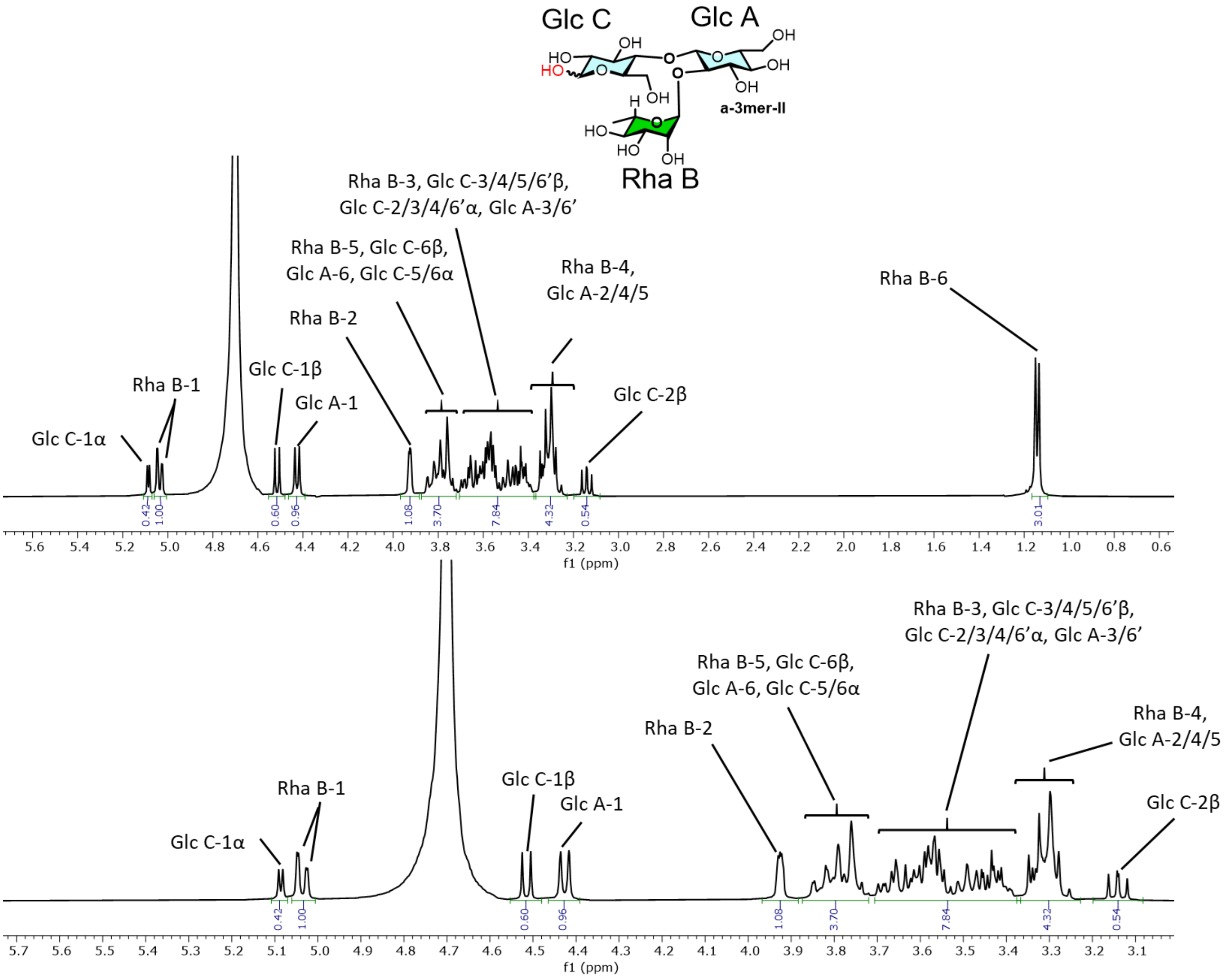

Figure S21 ^{1}H NMR (400 MHz, D_2O) of **a-3mer-II** with assignments.

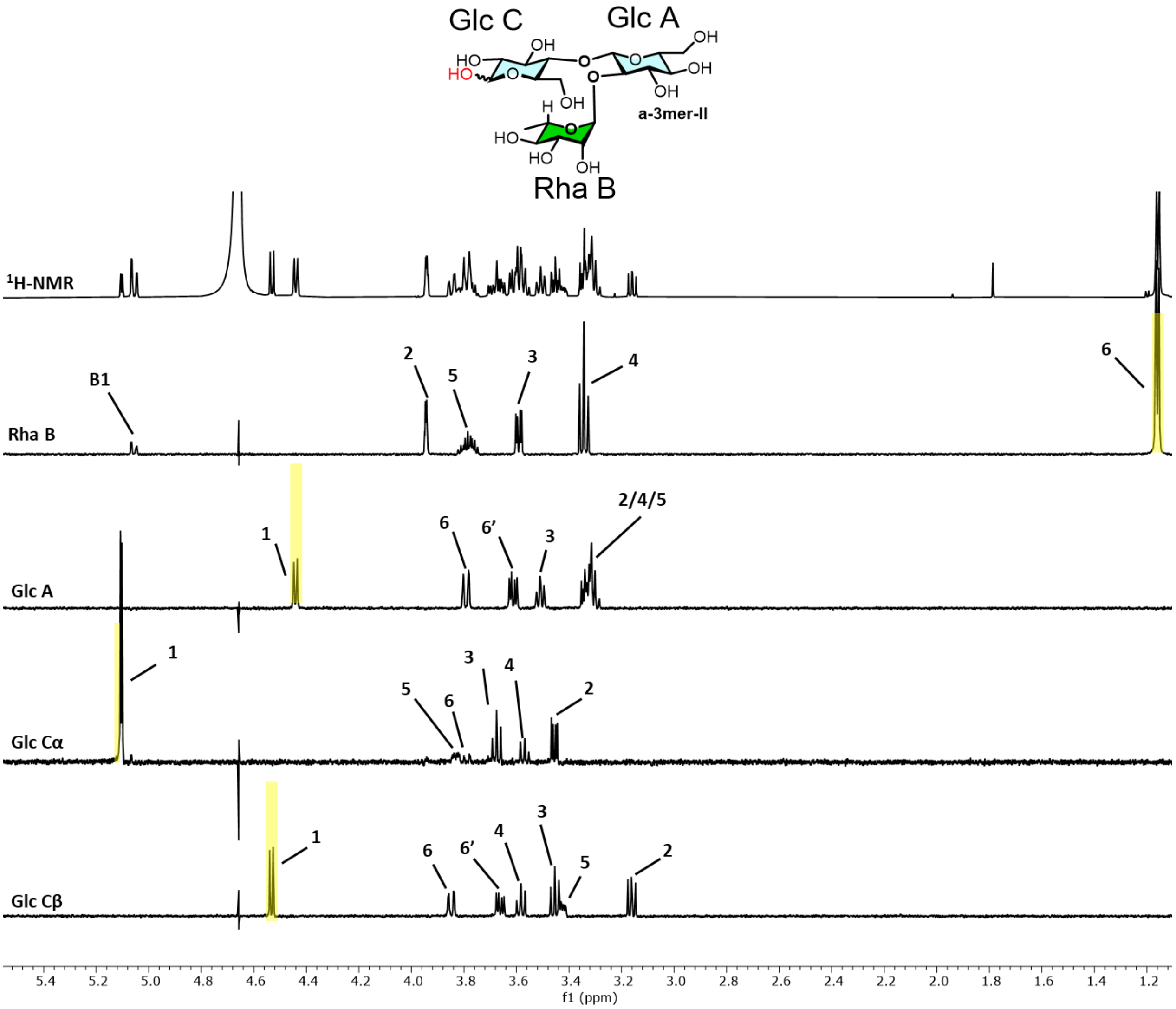

Figure S22 1D TOCSY (600 MHz, d9 200 ms, D_2O) of **a-3mer-II** with assignments. Resonances chosen
for selective excitation are highlighted in yellow.

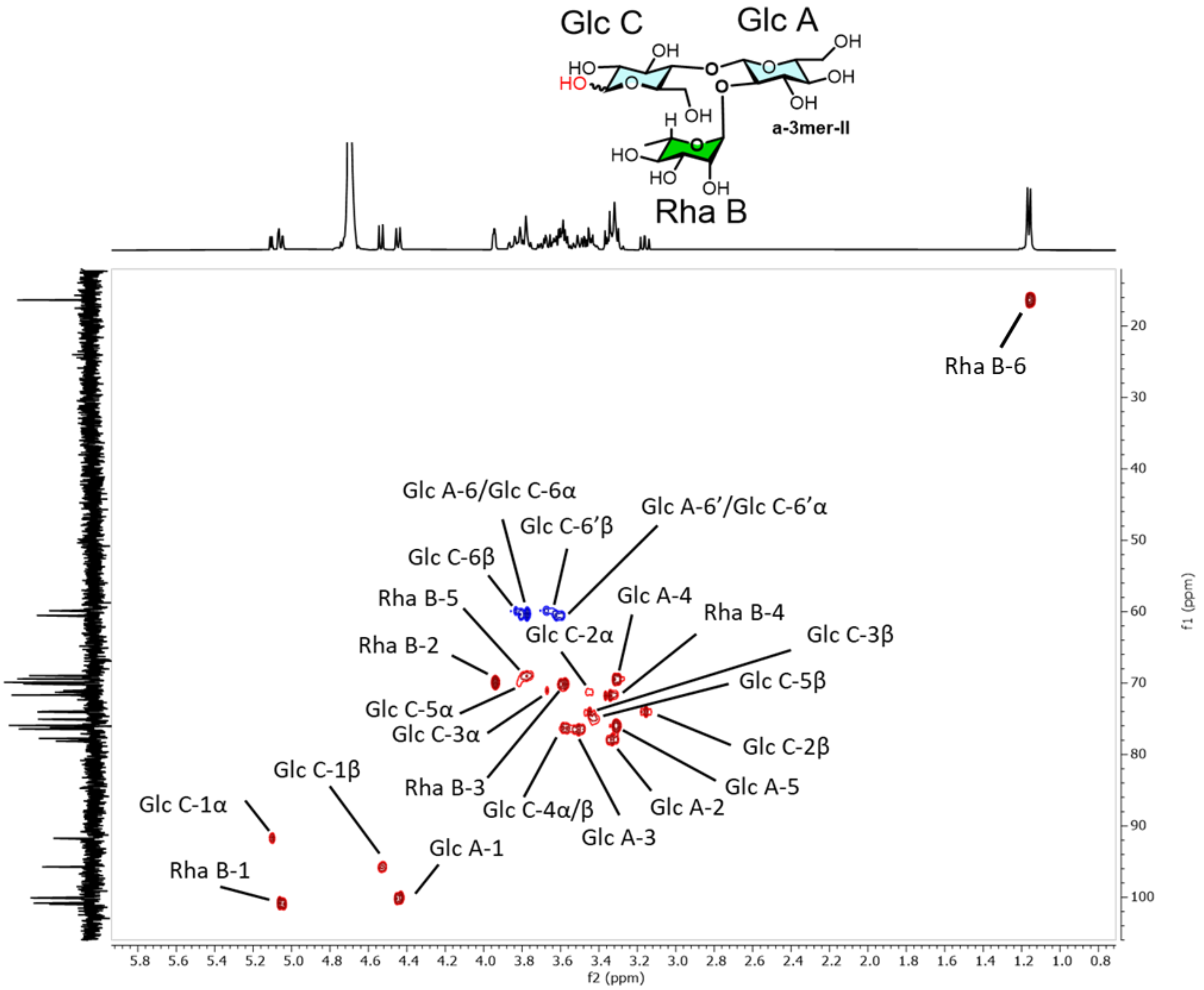

Figure S23 HSQC NMR (D_2O) of **a-3mer-II** with assignments.

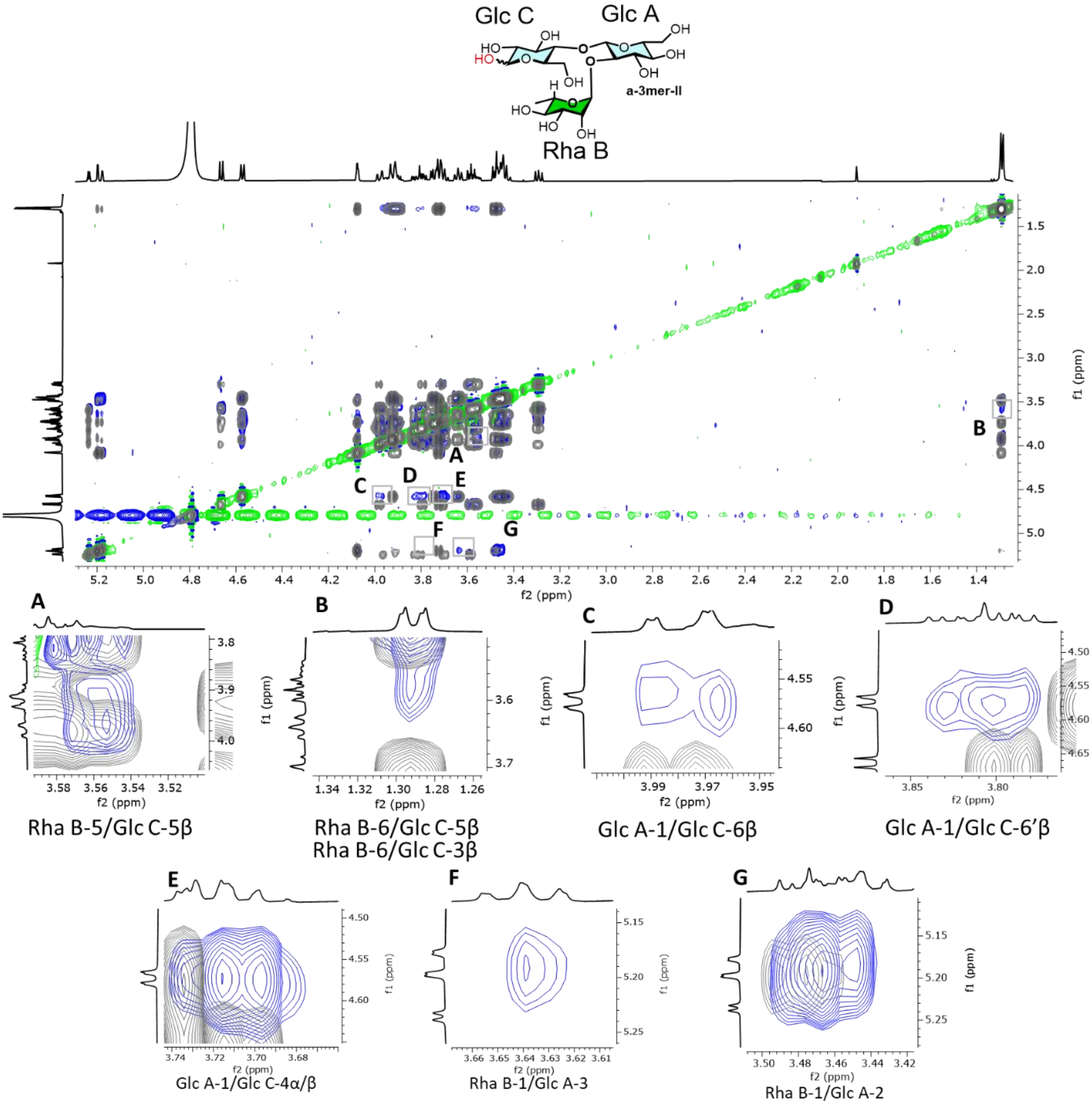

Figure S24 Superimposed 2D NOESY (green-blue, 600 MHz, d8 800 ms, 293 K, D_2O) of **a-3mer-II** with assignments and 2D TOCSY spectrum (gray, 600 MHz, d9 200 ms, D_2O).

6.3 NMR characterization of a-3mer-IV

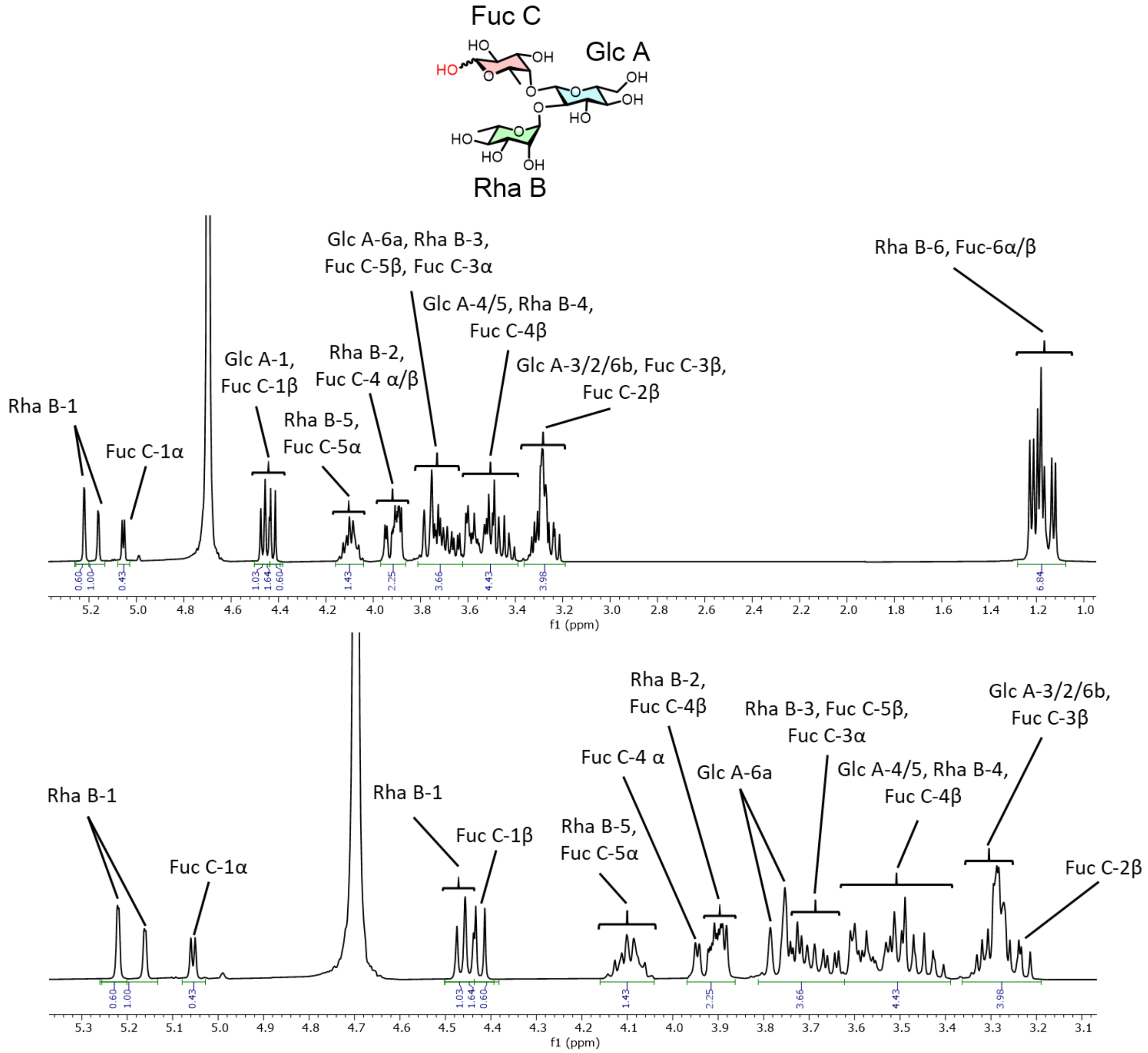

Figure S25 ^{1}H NMR (400 MHz, D_2O) of **a-3mer-IV** with assignments.

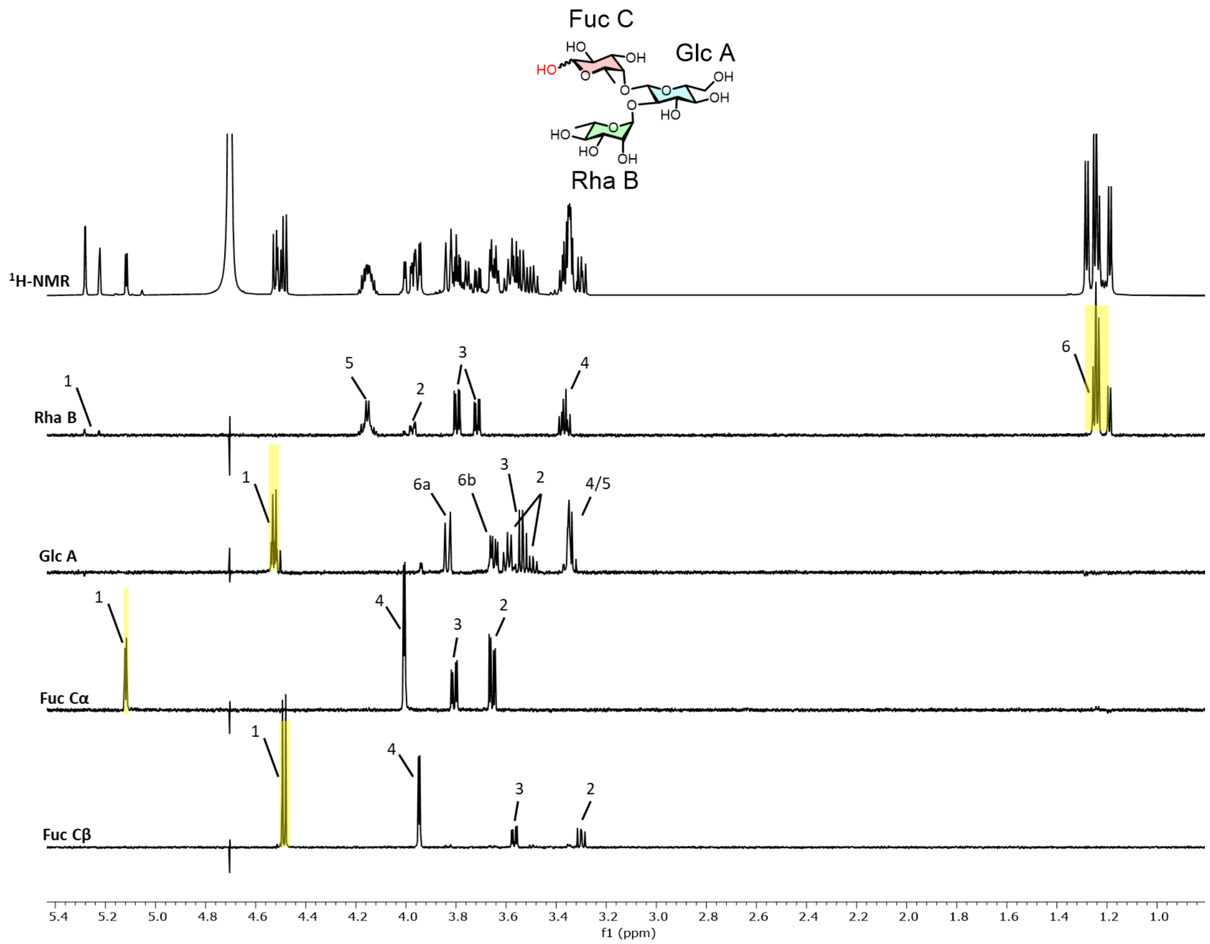

Figure S26 1D TOCSY (600 MHz, d9 200 ms, D_2O) of **a-3mer-IV** with assignments. Resonances chosen for selective excitation are highlighted in yellow.

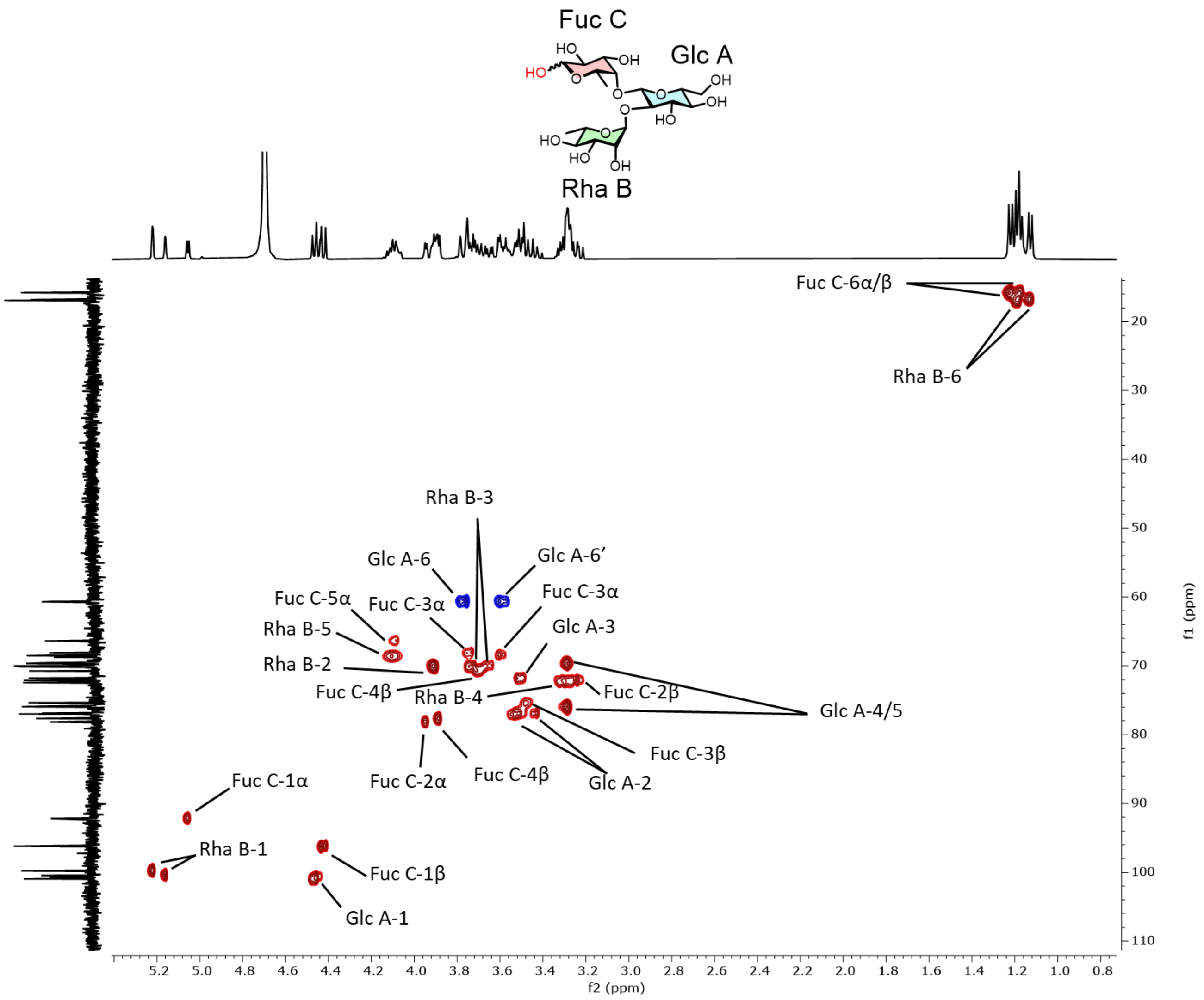

Figure S27 HSQC NMR (D_2O) of **a-3mer-IV** with assignments.

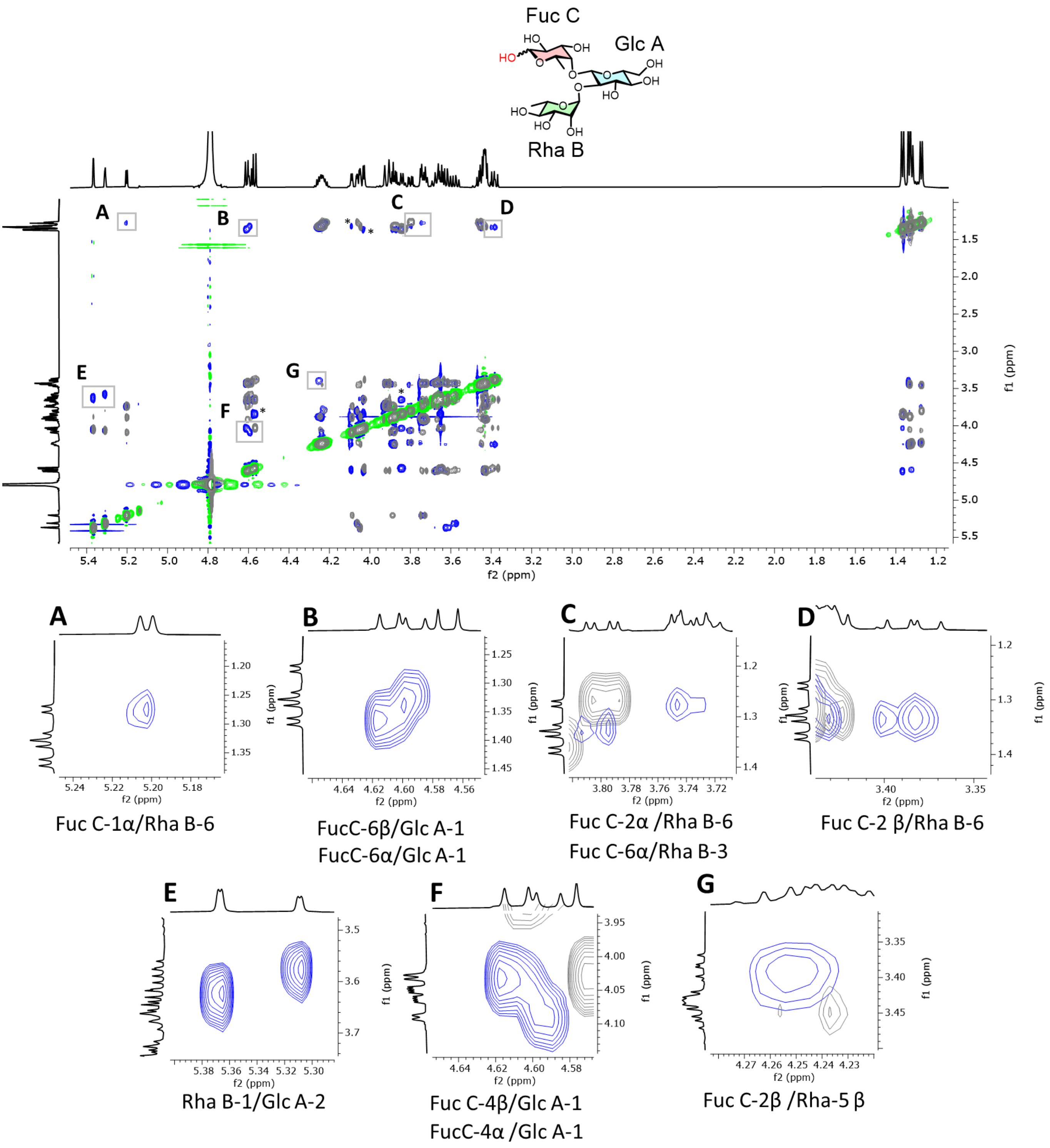

Figure S28 Superimposed 2D NOESY (green-blue, 600 MHz, d8 800 ms, 293 K, D_2O) of **a-3mer-IV** with assignments and 2D TOCSY spectrum (gray, 600 MHz, d9 200 ms, D_2O).

6.4 NMR characterization of a-5mer-IV

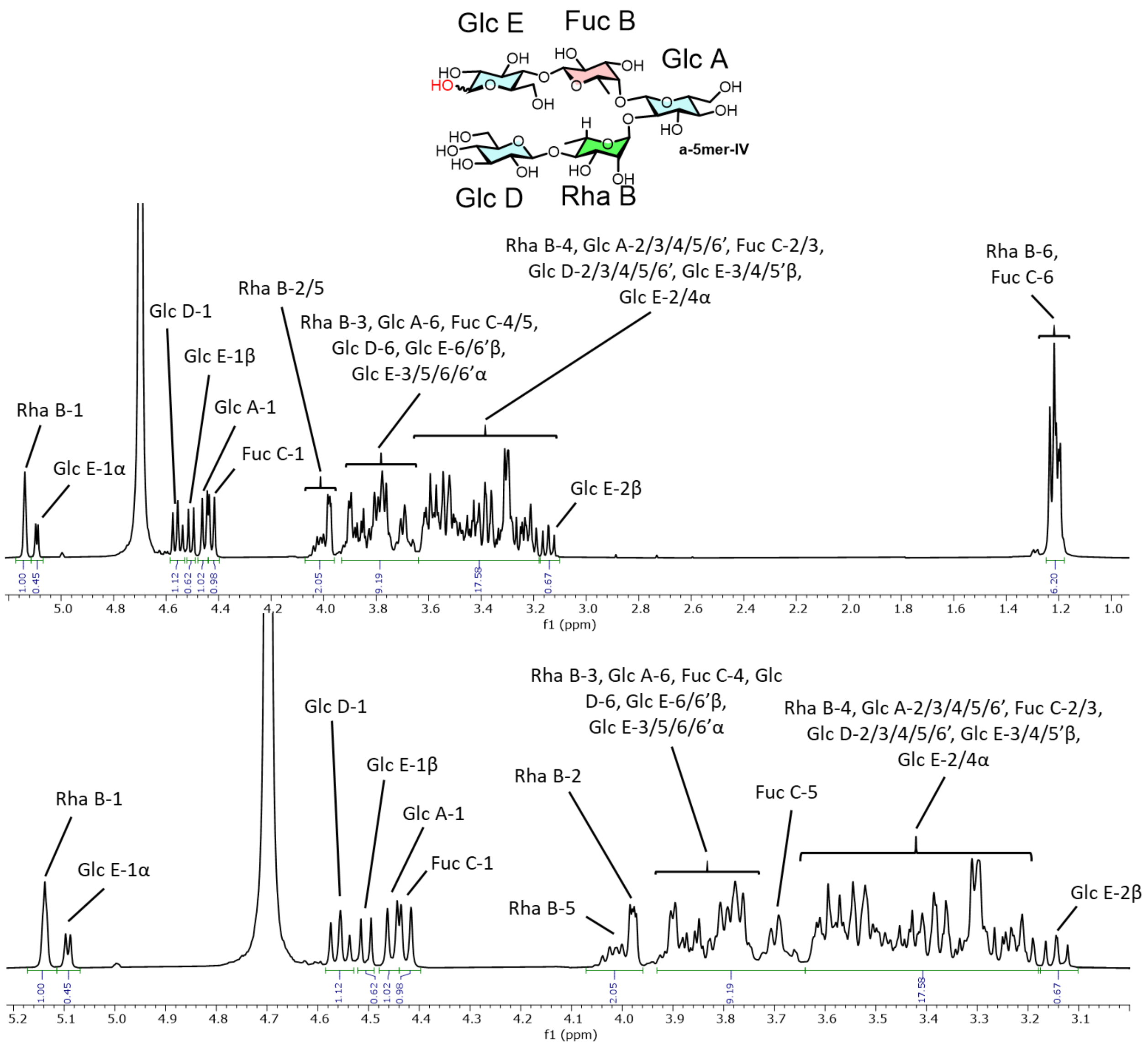

Figure S29 ^{1}H NMR (400 MHz, D_2O) of **a-5mer-IV** with assignments.

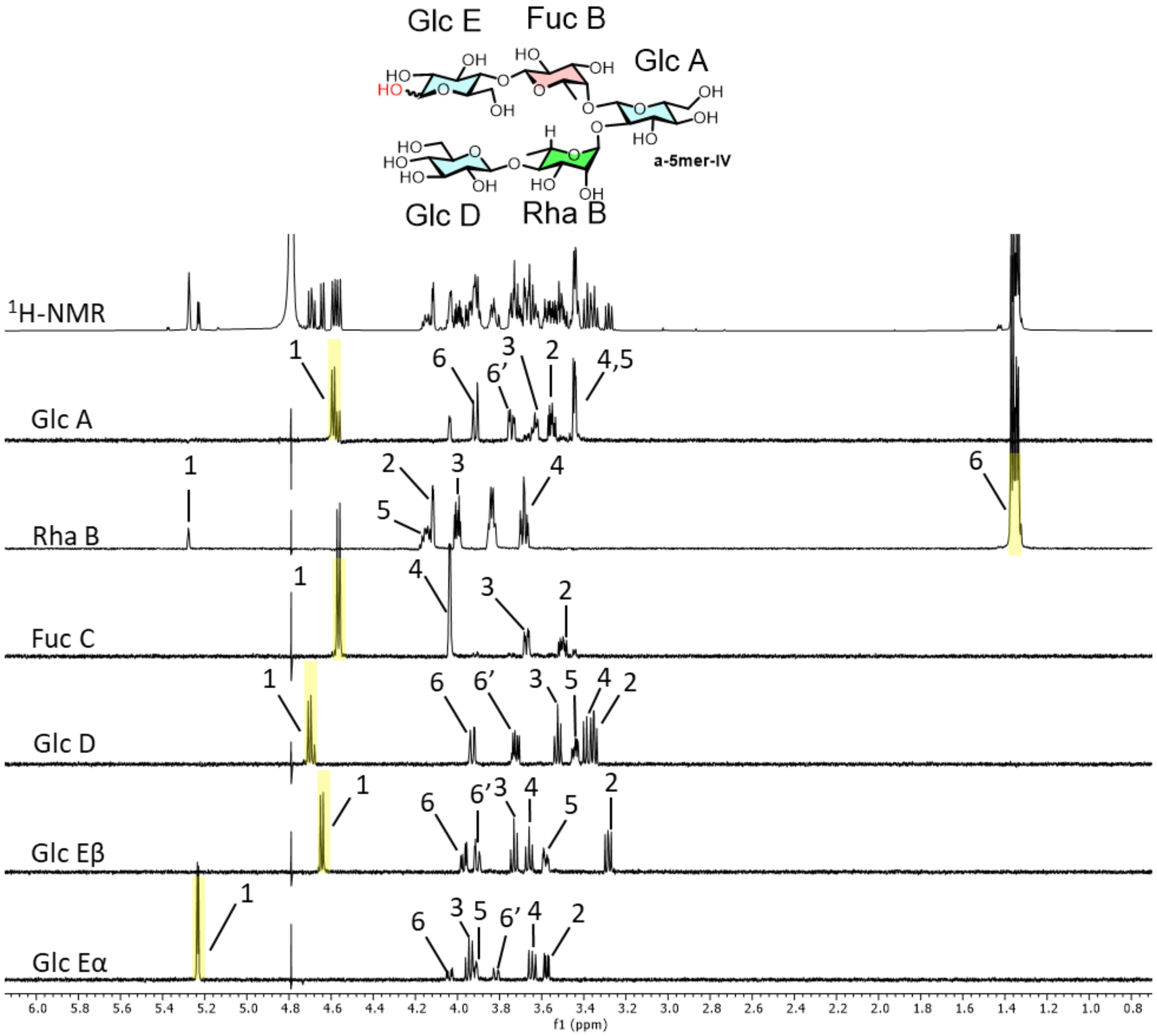

Figure S30 1D TOCSY (600 MHz, d9 200 ms, D_2O) of **a-5mer-IV** with assignments. Resonances chosen for selective excitation are highlighted in yellow.

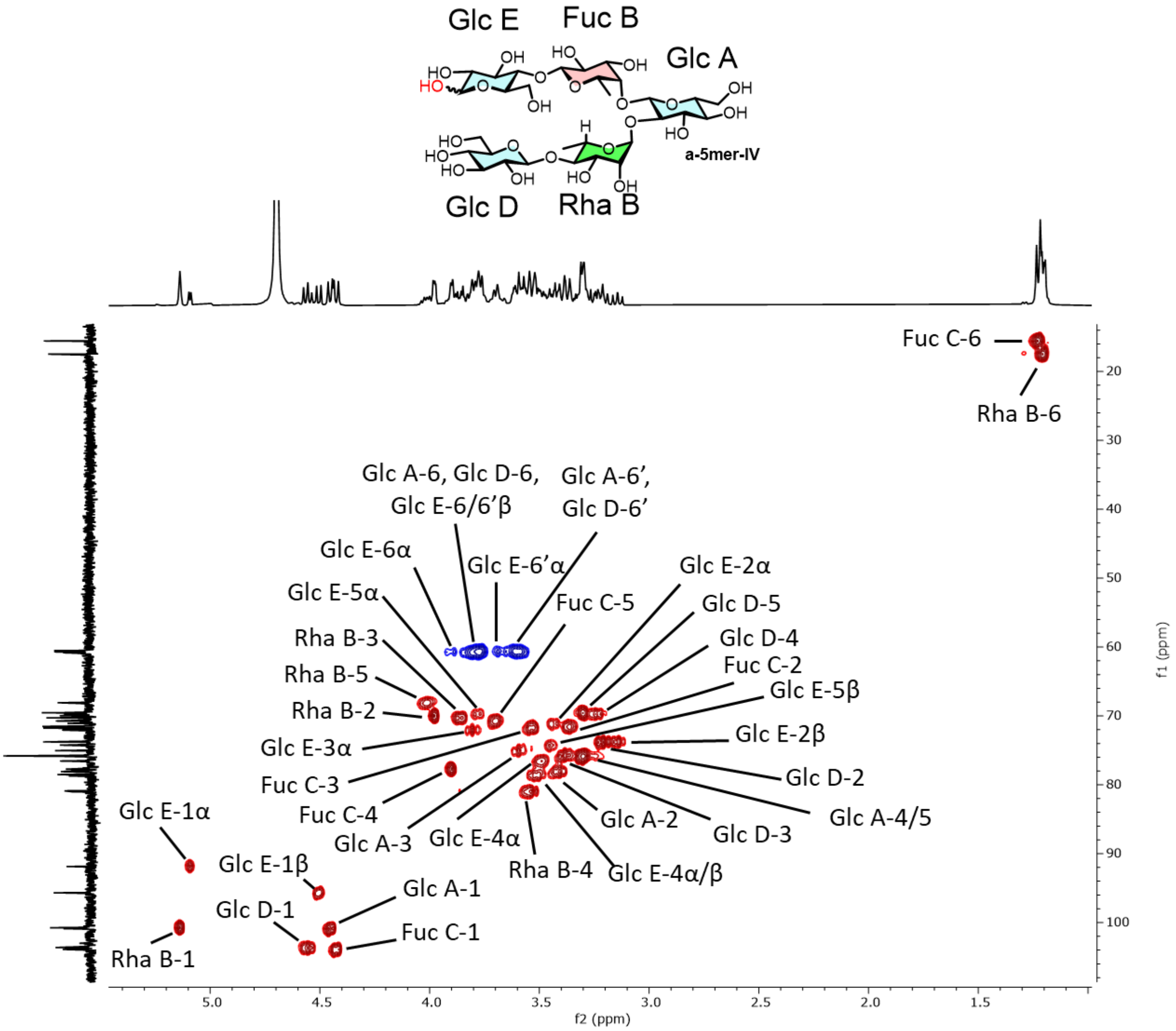

Figure S31 HSQC NMR (D_2O) of **a-5mer-IV** with assignments.

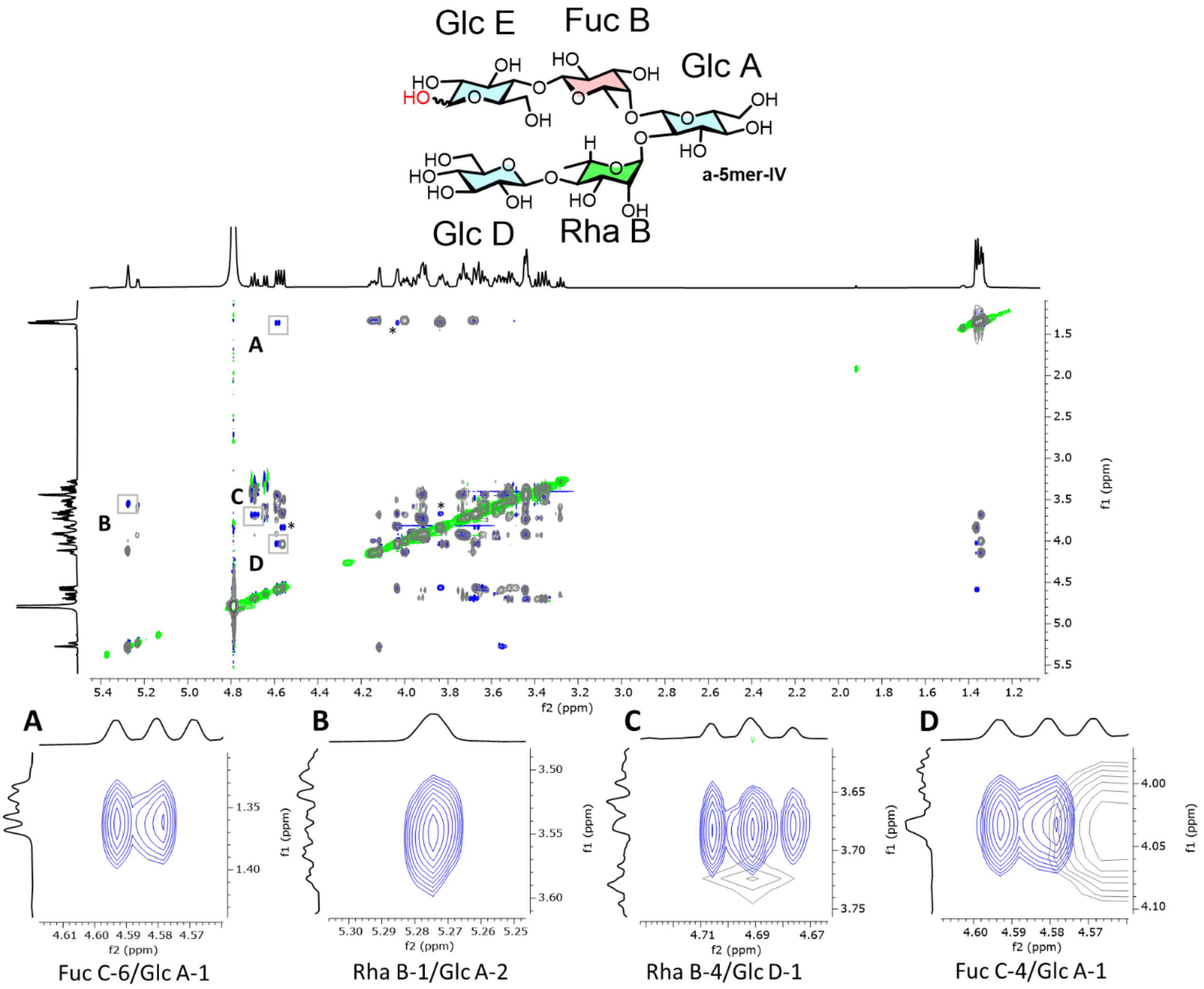

Figure S32 Superimposed 2D ROESY (green-blue, 600 MHz, p15 300 ms, 293 K, D_2O) of **a-5mer-IV** with assignments and 2D TOCSY spectrum (gray, 700 MHz, d9 200 ms, D_2O).

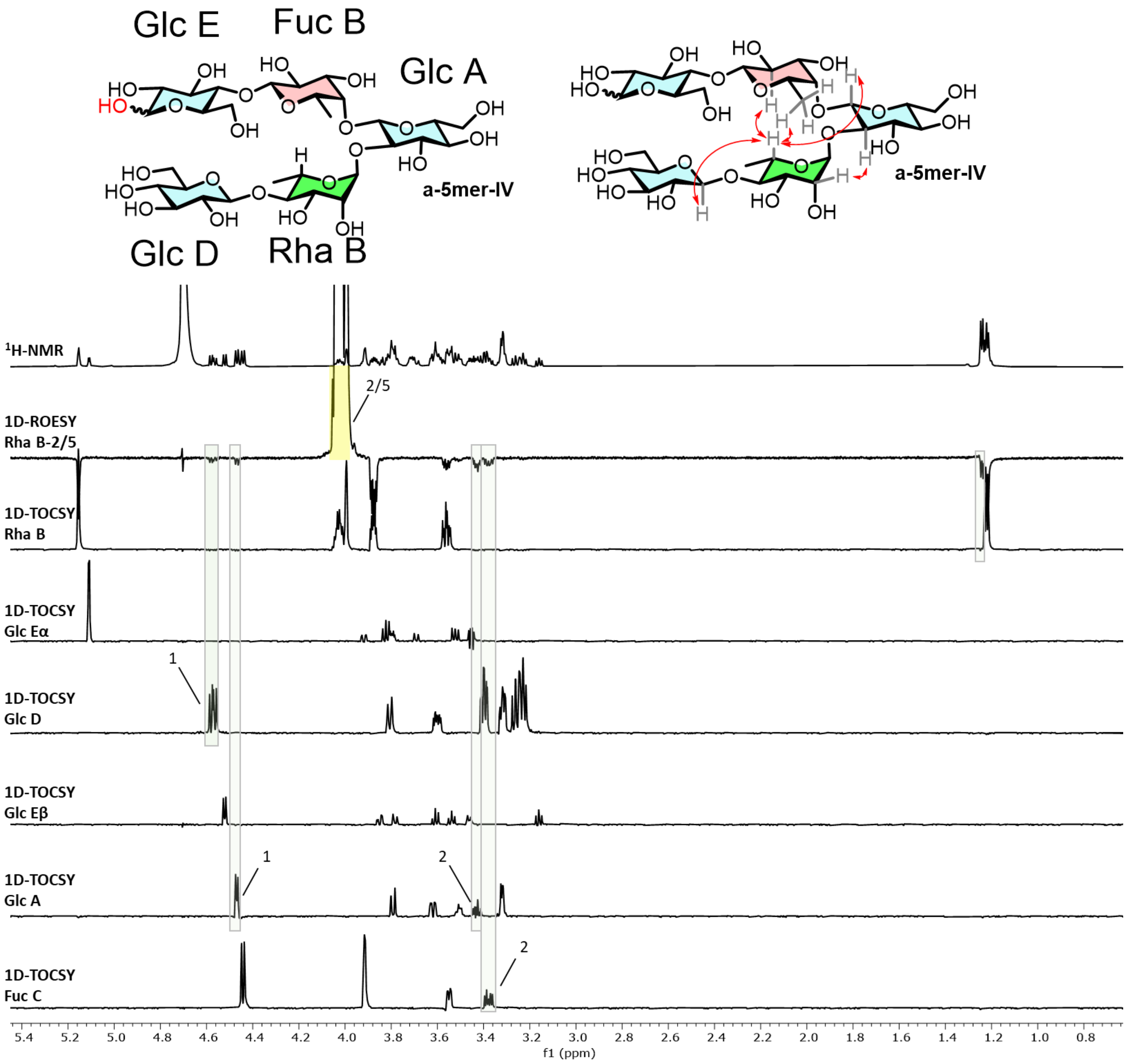

Figure S33 Overlay of 1D ROESY (700 MHz, p15 300 ms, 293 K, D_2O) and 1D TOCSY of **a-5mer-IV**. The 1D ROESY was obtained by selective excitation of the Rha-5 & 2 resonances (δ 3.97 – 4.05 ppm) highlighted with a yellow box. The NOE between Glc D-1/Rha B-5, Glc A-1/Rha B-5, Glc A-2/Rha B-2, Fuc C-2/Rha B-5 and Fuc C-6/Rha B-5 is highlighted with gray boxes.

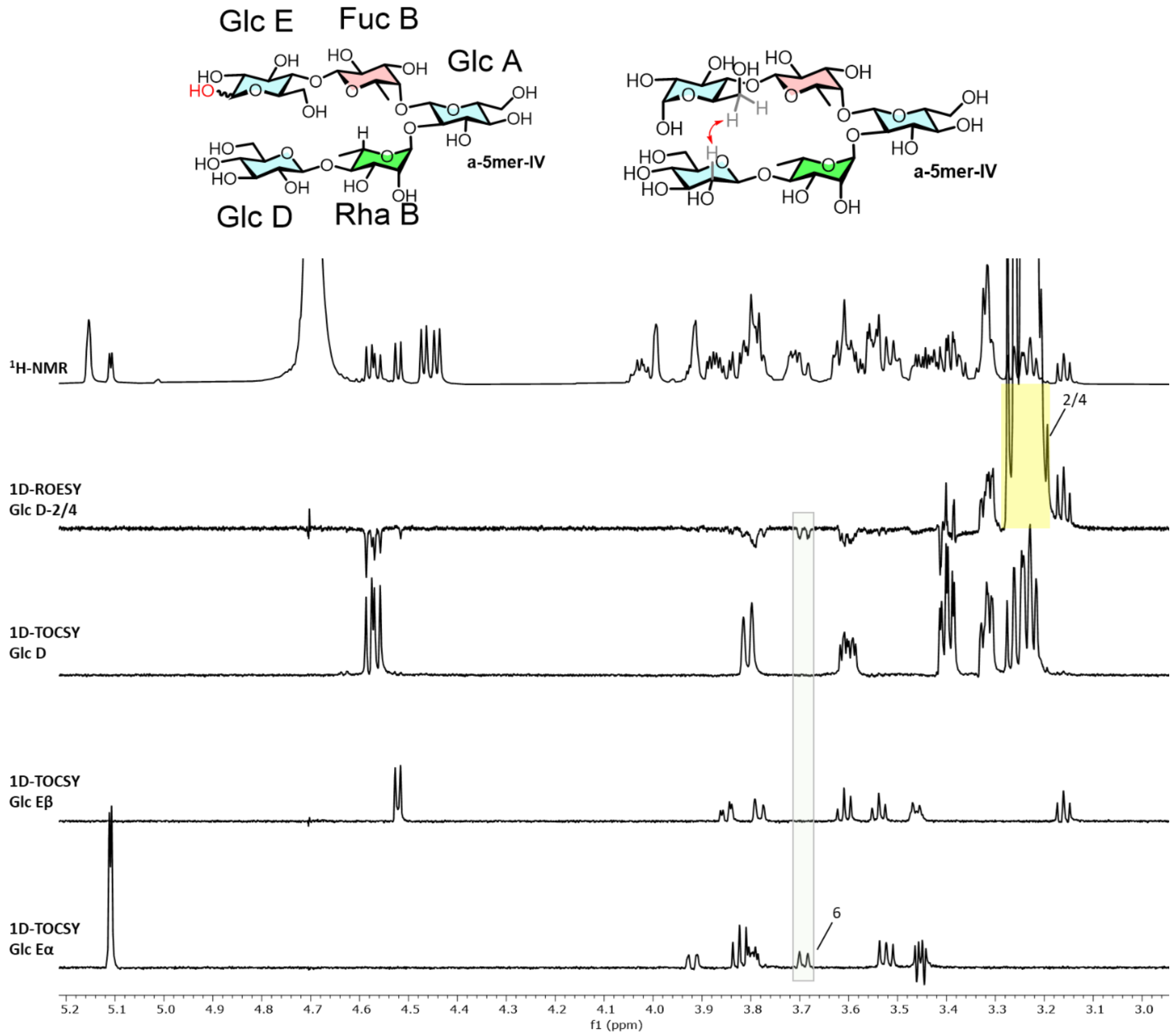

Figure S34 Overlay of 1D ROESY (700 MHz, p15 300 ms, 293 K, D_2O) and 1D TOCSY of **a-5mer-IV**. The 1D ROESY was obtained by selective excitation of the Glc-2 & 4 resonances (δ 3.20 – 3.24 ppm) highlighted with a yellow box. The NOE between Glc D-2/Glc E-6α is highlighted with a gray box.

6.5 NMR characterization of a-9mer-II

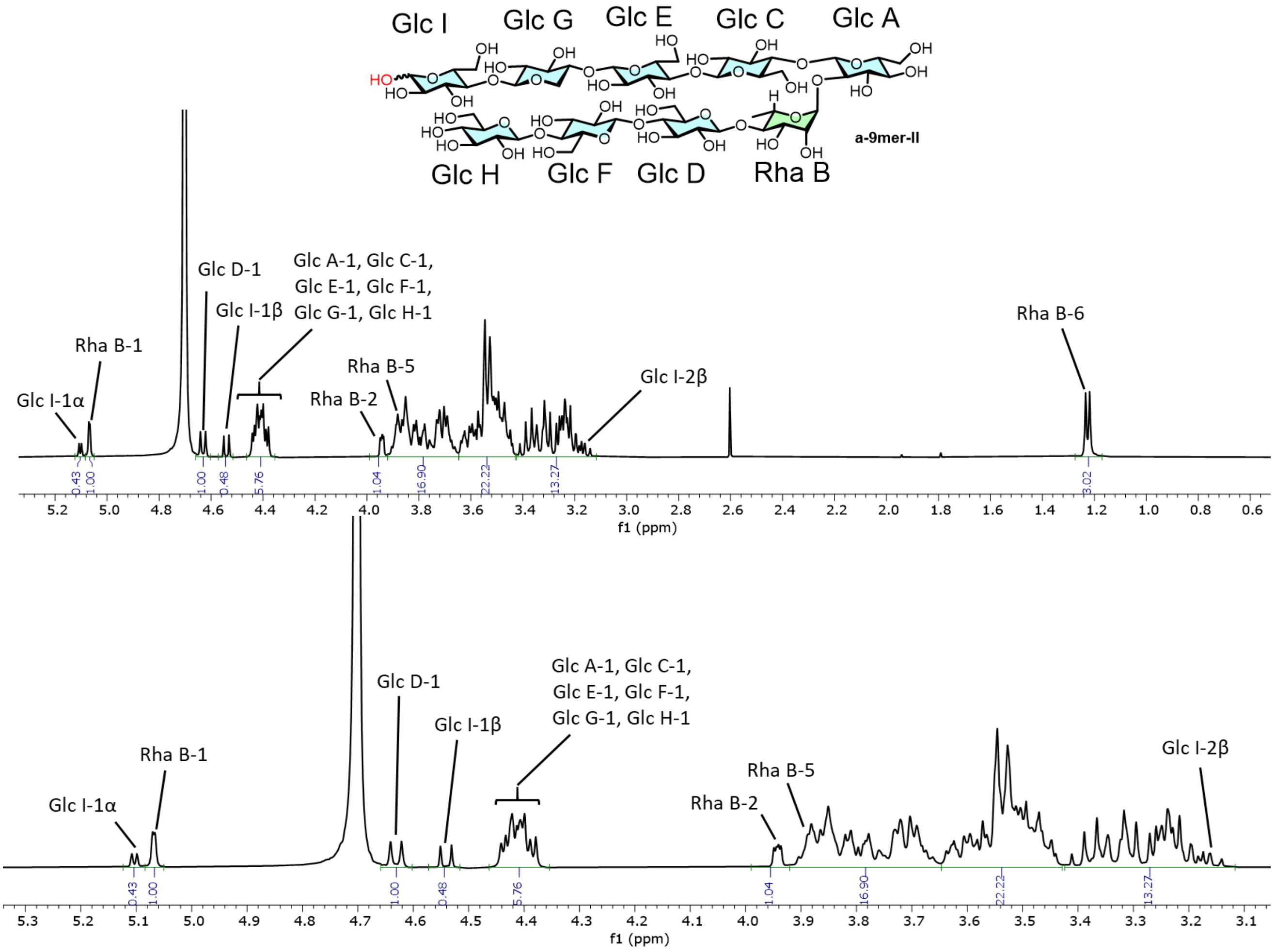

Figure S35 ^{1}H NMR (400 MHz, D_2O) of **a-9mer-II** with assignments.

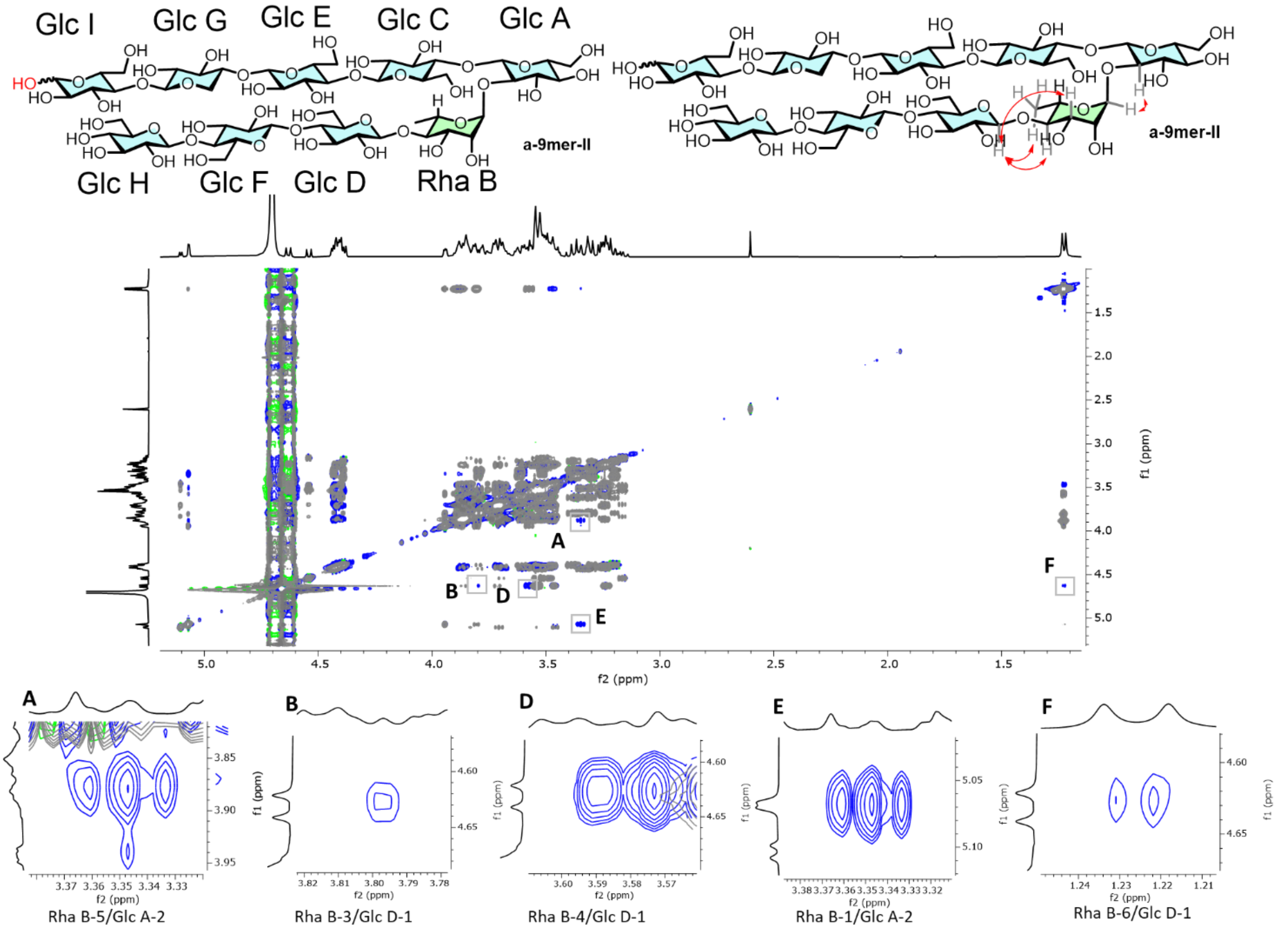

Figure S36 Superimposed 2D NOESY (green-blue, 600 MHz, d8 400 ms, 293 K, D_2O) of **a-9mer-II** with assignments and 2D TOCSY spectrum (gray, 600 MHz, d9 80 ms, D_2O).

6.6 NMR characterization of a-9mer-IV

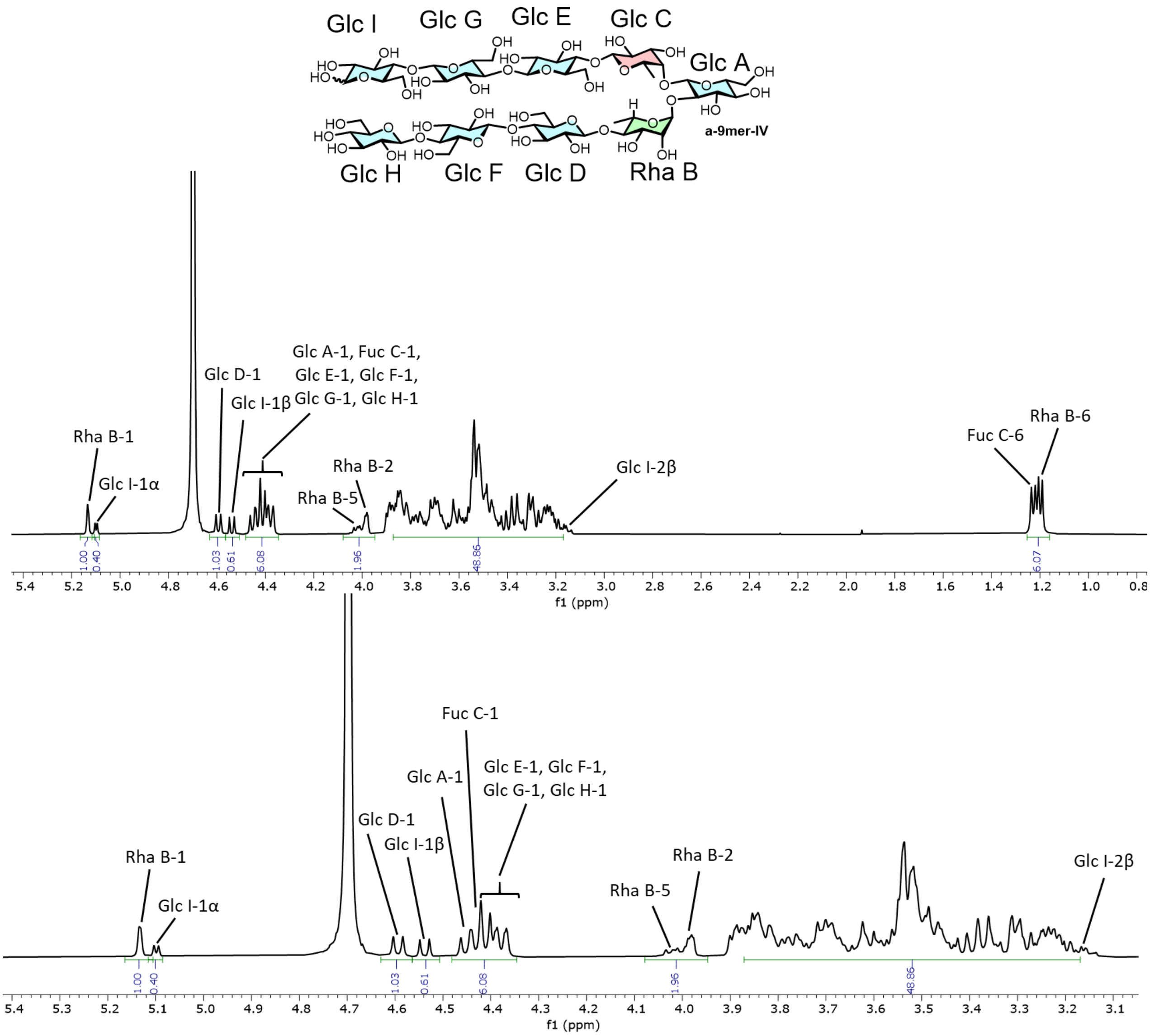

Figure S37 ^{1}H NMR (400 MHz, D_2O) of **a-9mer-IV** with assignments.

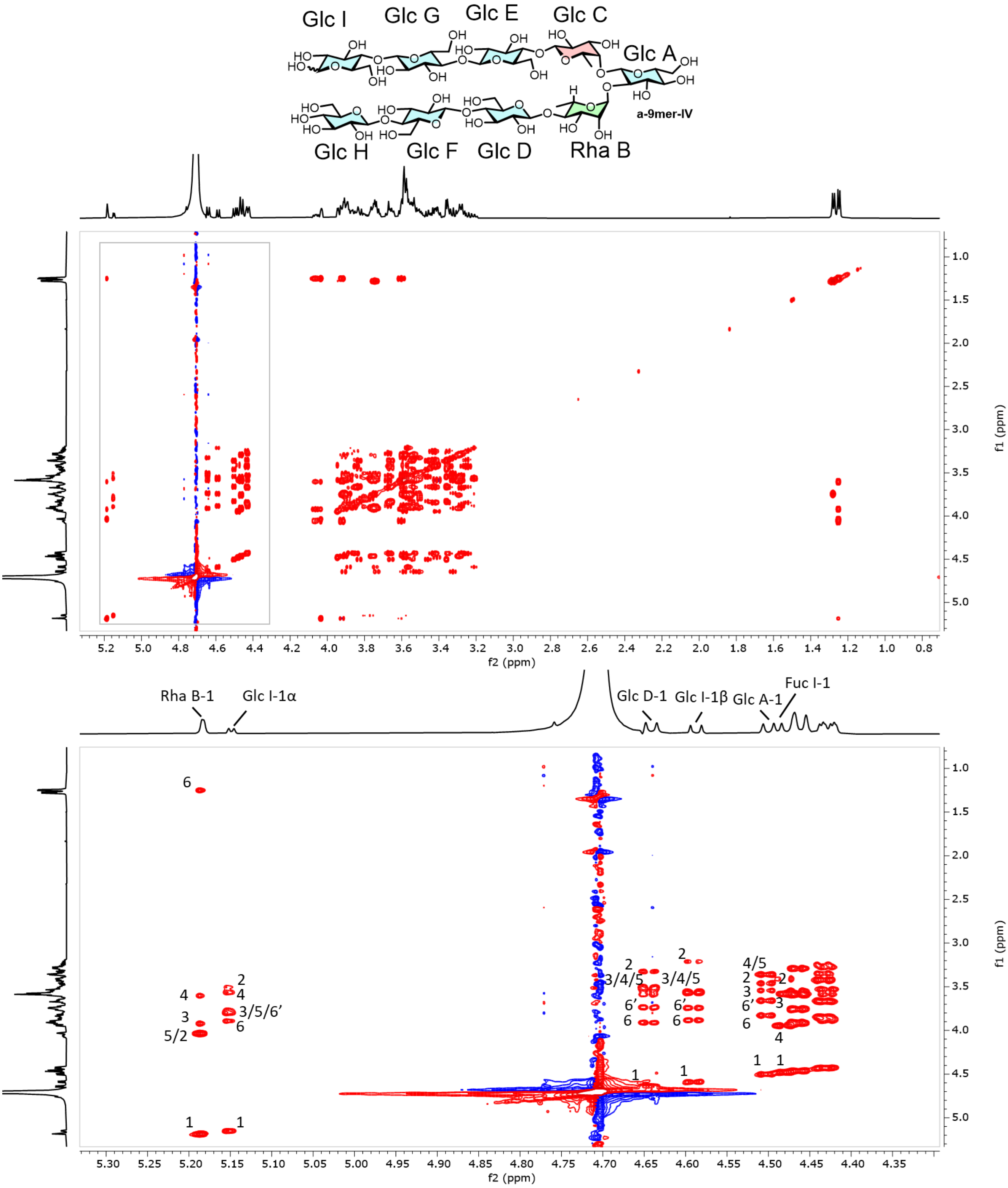

Figure S38 2D TOCSY (600 MHz, d9 80 ms, D_2O) of **a-9mer-IV** with assignments.

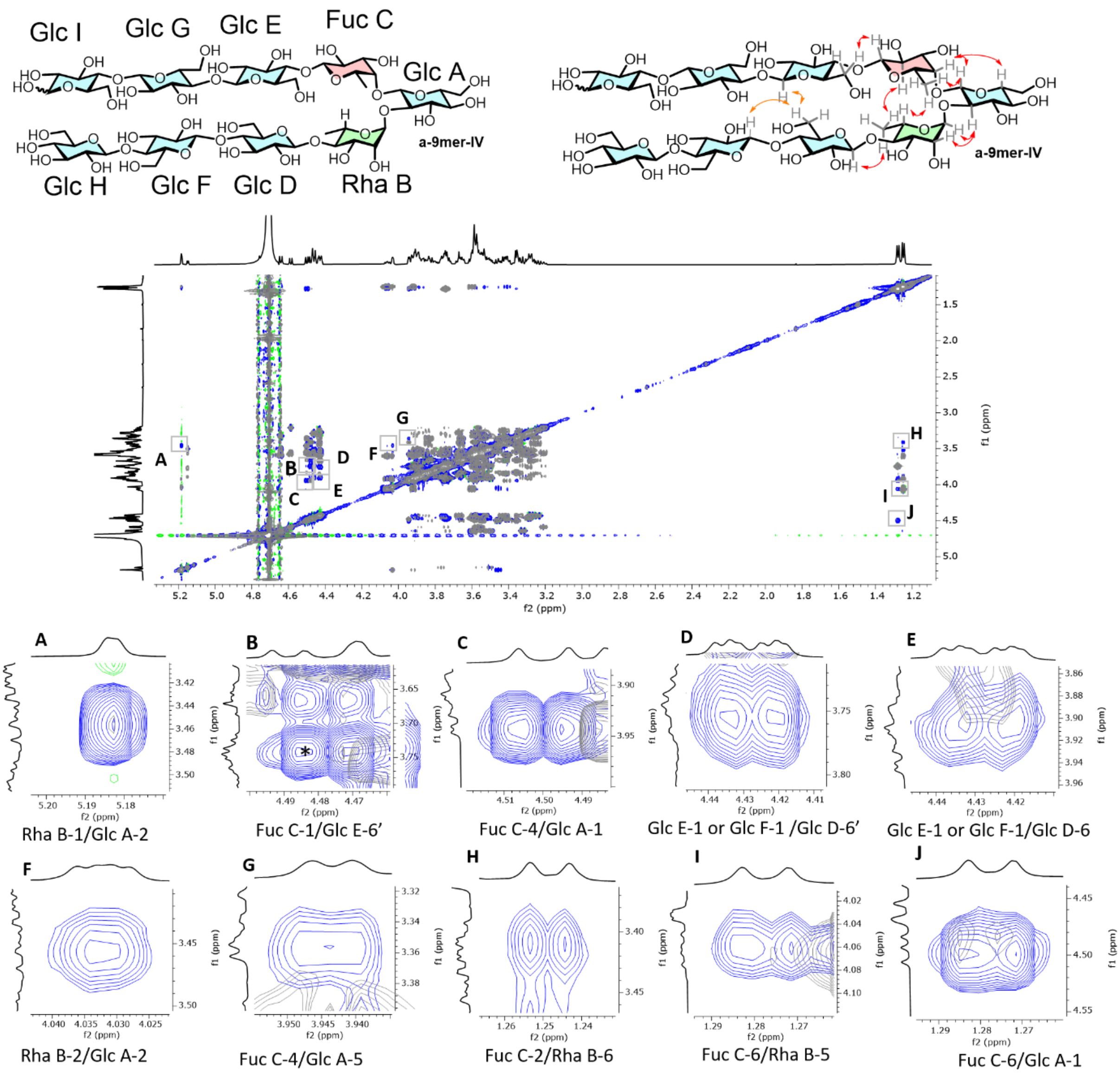

Figure S39 Superimposed 2D NOESY (green-blue, 600 MHz, d8 600 ms, 293 K, D_2O) of **a-9mer-IV** with assignments and 2D TOCSY spectrum (gray, 600 MHz, d9 80 ms, D_2O).

7 SAXS analysis

7.1 General materials and methods

X-ray scattering experiments were performed at the D2AM beamline of the European Synchrotron Radiation Facility (ESRF). Samples of **a-9mer-II**, **a-9mer-IV** and **ttt-15mer-IV** at concentrations 0.5 % wt at 25 °C were sealed in glass capillaries and mounted on a motorized sample exchanger. They were exposed to monochromatic X-rays of 15.8 eV (λ = 0.7847 Å). The scattering intensity was measured using two-dimensional pixel detectors (XPAD-WOS and D5). The data processing was performed using pyFAI software. Intensity and Rg analysis were done using Gnuplot software.

7.2 Conformational grouping and SAXS curve calculation

To group conformations for SAXS analysis, a window-based approach was applied using 1 ns windows along the trajectory. For each window, averaged radius of gyration (Rg) was calculated, which helped reduce the effect of short-timescale fluctuations. This averaging strategy reflects the idea that conformational states-especially in glycan-rich systems-are better represented as dynamics ensembles rather than rigid structures.

Based on the distribution of average Rg values (as summarized in Table S25), conformations were categorized into two representative states: folded conformation A and unfolded conformation B. All trajectory window assigned to the same Rg-defined group were concatenated, and a single SAXS curve was calculated for each conformational state using the combined frames. In this work was studied combined models considering only two conformations A and B.

Table S25. Rg intervals (nm) used for conformational grouping of method 1 considering two conformations

	**A**	**B**
**a-9mer-II**	Rg < 0.79	Rg > 0.79
**a-9mer-IV**	Rg < 0.79	Rg > 0.79
**ttt-15mer-IV**	Rg < 0.98	Rg > 0.98

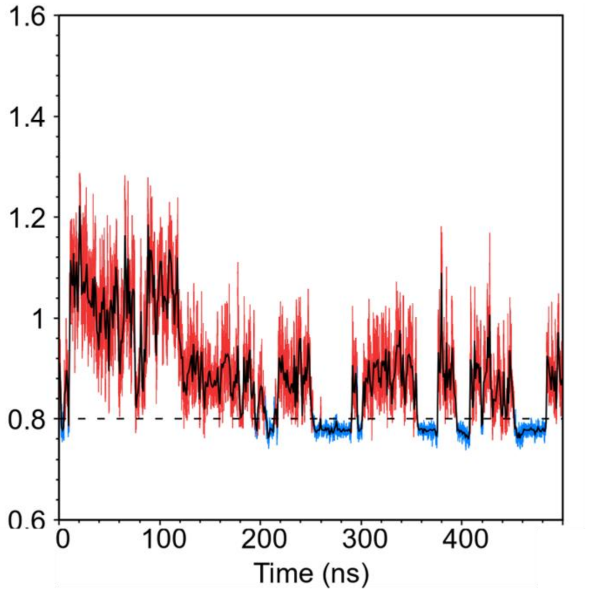

Figure S40 Radius of gyration profile and conformational state classification for **a-9mer-II** system.

Figure S40 shows the superposition of the average Rg values per window and the full Rg time series for the **a-9mer-II** system as an example; the threshold used to define the Rg categories are indicated with dashed line on the average Rg profile.

7.3 SAXS calculation

Explicit-solvent SAXS calculations were performed based on a modified version[17,18] of GROMACS 2022.2 (GROMACS-SWAXS)[19]. Details of explicit-solvent SAXS calculations are presented in a previous publication.[20] Here, in this modified Gromacs code, the solvent molecules in the solvation layer were considered in the SAXS intensity calculation, in contrast to the common approach where only the solute molecules are used for the SAXS intensity simulations. This explicit solvent model can provide realistic simulated intensities, as the density fluctuation of water molecules around the solute certainly contributes to the overall SAXS intensities. A spatial envelope was built around the hairpin at a distance of 0.7 nm. The water subtraction was carried out using > 1000 simulation frames of the pure-water simulation box. The atomic form factors were approximated by $f(q) = c + \sum_{k=1}^{4} a_k \exp[-b_k(q/4\pi)^2]$, where the values ak, bk and c are the Cromer-Mann parameters[21]. The orientational average was carried out using 500 q-vectors for each absolute value of q, and the solvent electron density was corrected to the experimental value of 334 e/nm^3.

7.4 SAXS fitting

To fit the experimental SAXS data, we performed a linear combination of the predicted SAXS curves corresponding to different Rg-defined conformational states. The Kratky plot, defined as $q^2I(q)$ versus q, where I(q) is the scattering intensity and q is the momentum transfer, enhances sensitivity to structural compactness and flexibility. It is particularly suitable for analyzing glycan systems with folded and unfolded conformation, as it accentuates deviations from ideal chain behavior and facilitates quantitative comparison between experimental and predicted SAXS profiles. The fitting was based on minimizing the squared difference in Kratky-

transformation intensities (I^K) between the experimental data and the model, using the q-range from 0 to 0.6 Å^{-1} for **a-9mer-II** and **a-9mer-IV**, and from 0 to 0.4 Å^{-1} for **ttt-15mer-IV**. The fitting function was defined as:

$I^K = q^2 \cdot I(q)$

$\chi^2(b,c) = [I^K_{exp}(q) - (a_1 \cdot b \cdot I^K_1(q) + a_2 \cdot c \cdot I^K_2\ (q) + a_3 \cdot (1 - b - c) \cdot I^K_3(q))]^2$

where:

$I^K_{exp}(q)$ is the experimental Kratky intensity at scattering vector q;

$I^K_1(q)$, $I^K_3(q)$ and $I^K_3(q)$ are the predicted Kratky SAXS curves for conformational states 1, 2, and 3;

a_1, a_2 and a_3 are given scaling factor for conformational states 1, 2, and 3, determined based on the plateau intensities in the low-q region of the SAXS profiles before fitting;

b and c are the fitting parameters representing the fractional contributions of states 1 and 2, respectively, and the contribution of the state 3 is given by 1 - b - c.

The fitting was constrained such that $0 \leq b \leq 1$, $0 \leq c \leq 1$ and $b + c \leq 1$. The optimization was performed using scipy.optimize.minimize.

To study the contribution of different conformational states to the overall shape of the designed glycans, a combinatorial approach was explored in combination with fitting of experimental SAXS data. To evaluate how deeply this conformational space could be analyzed using this methodology, two methods were employed. The conformational ensemble was simplified into two main states: the folded conformation (A) and the unfolded conformation (B).

7.5 Experimental Rg calculation

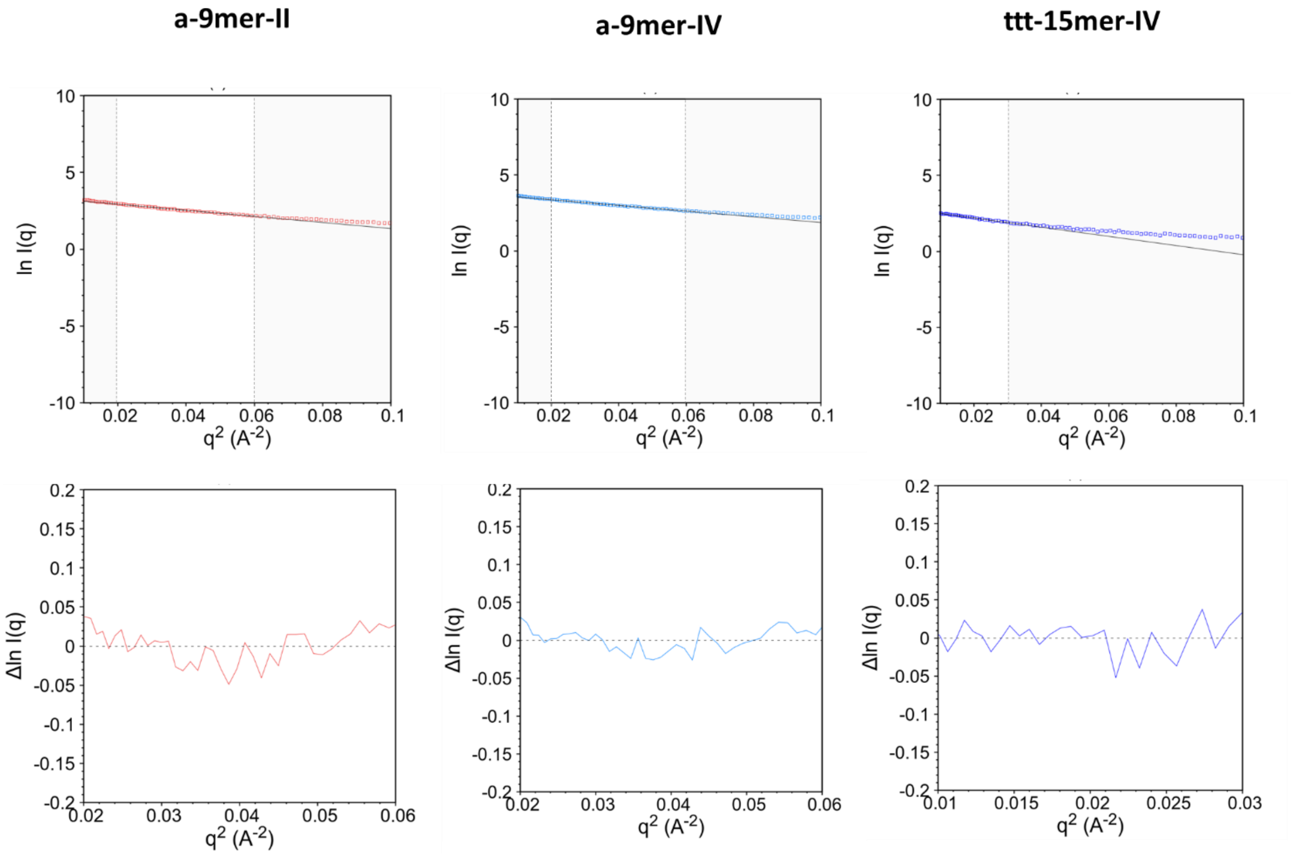

Figure S41. Guinier plots and residuals of **a-9mer-II**, **a-9mer-IV** and **ttt-15mer-IV**

Table S26. Curve fitting of natural logarithm of I(q) vs. q^2 plots of Guinier law-SAXS measurements of **a-9mer-II**, **a-9mer-IV** and **ttt-15mer-IV**

	**a-9mer-II**		**a-9mer-IV**		**ttt-15mer-IV**	
	**Value**	**Error**	**Value**	**Error**	**Value**	**Error**
**m**	-19.9	0.29	-18.8	0.19	-30.2	0.64
**n**	3.33	0.01	3.74	0.01	2.80	0.01
**R_g (Å)**	7.72	0.05	7.50	0.03	9.52	0,10
**RMSE**	0.018		0.062		0.020	

7.6 Conformational analysis of a-9mer-II

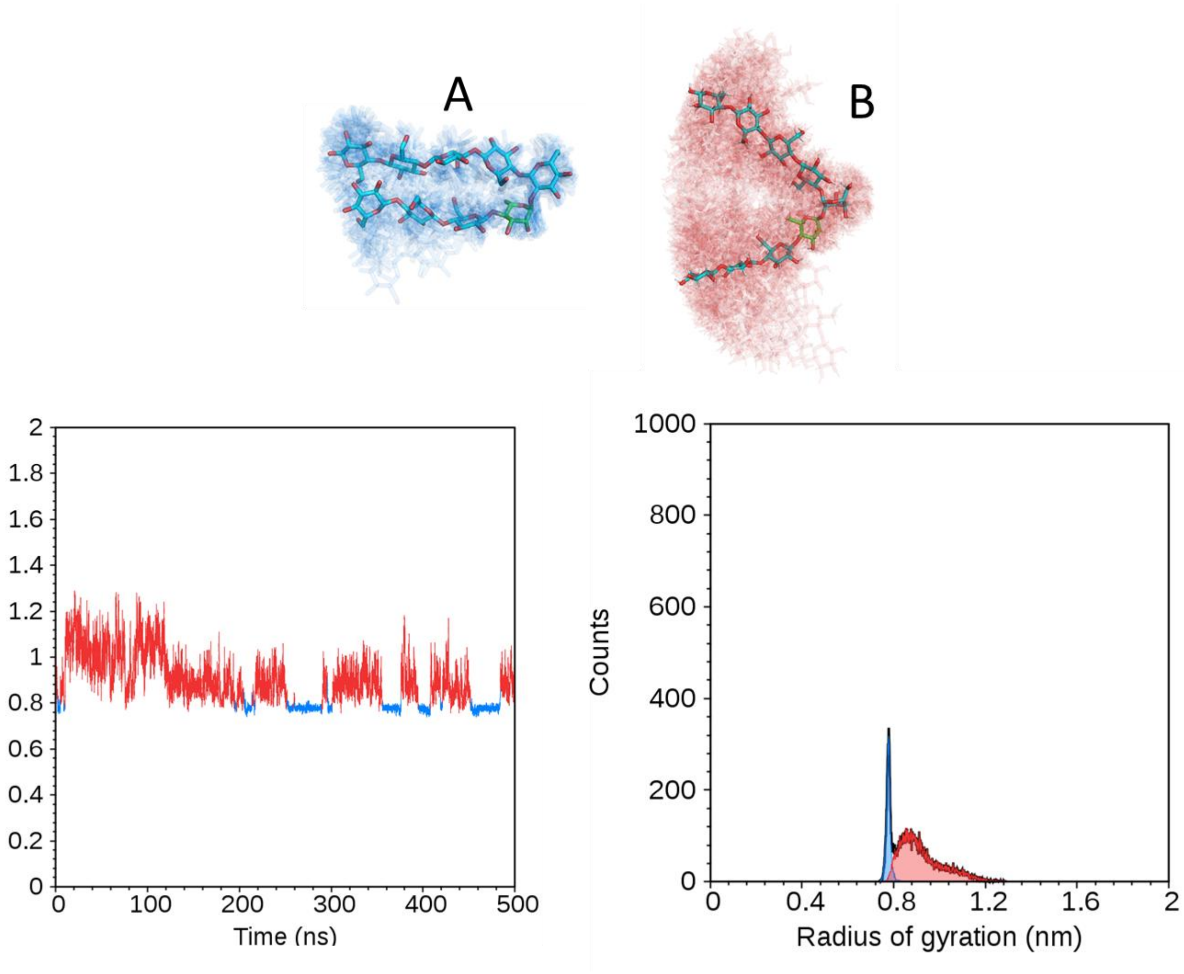

Figure S42. Conformational states mapped by radius of gyration: Distributions and alignments

Table S27. Relative abundance of molecular conformations of **a-9mer-II** from MD simulations

	**A**	**B**
**Percentage**	25.0	75.0

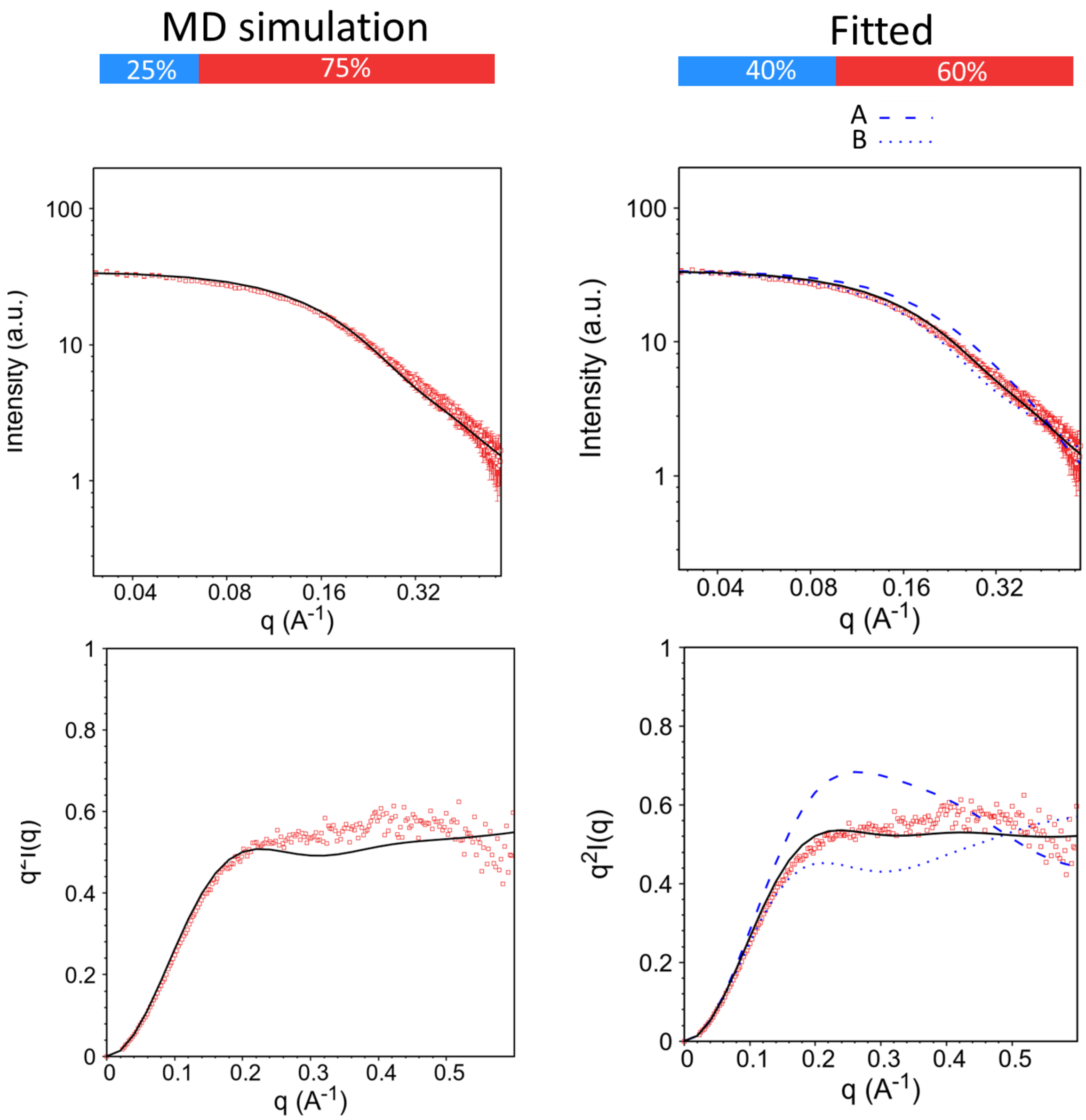

Figure S43. Comparison of experimental and predicted SAXS profiles and Kratky plots of **a-9mer-II**: individual conformations (dashed) and combined model (solid).

7.7 Conformational analysis of a-9mer-IV

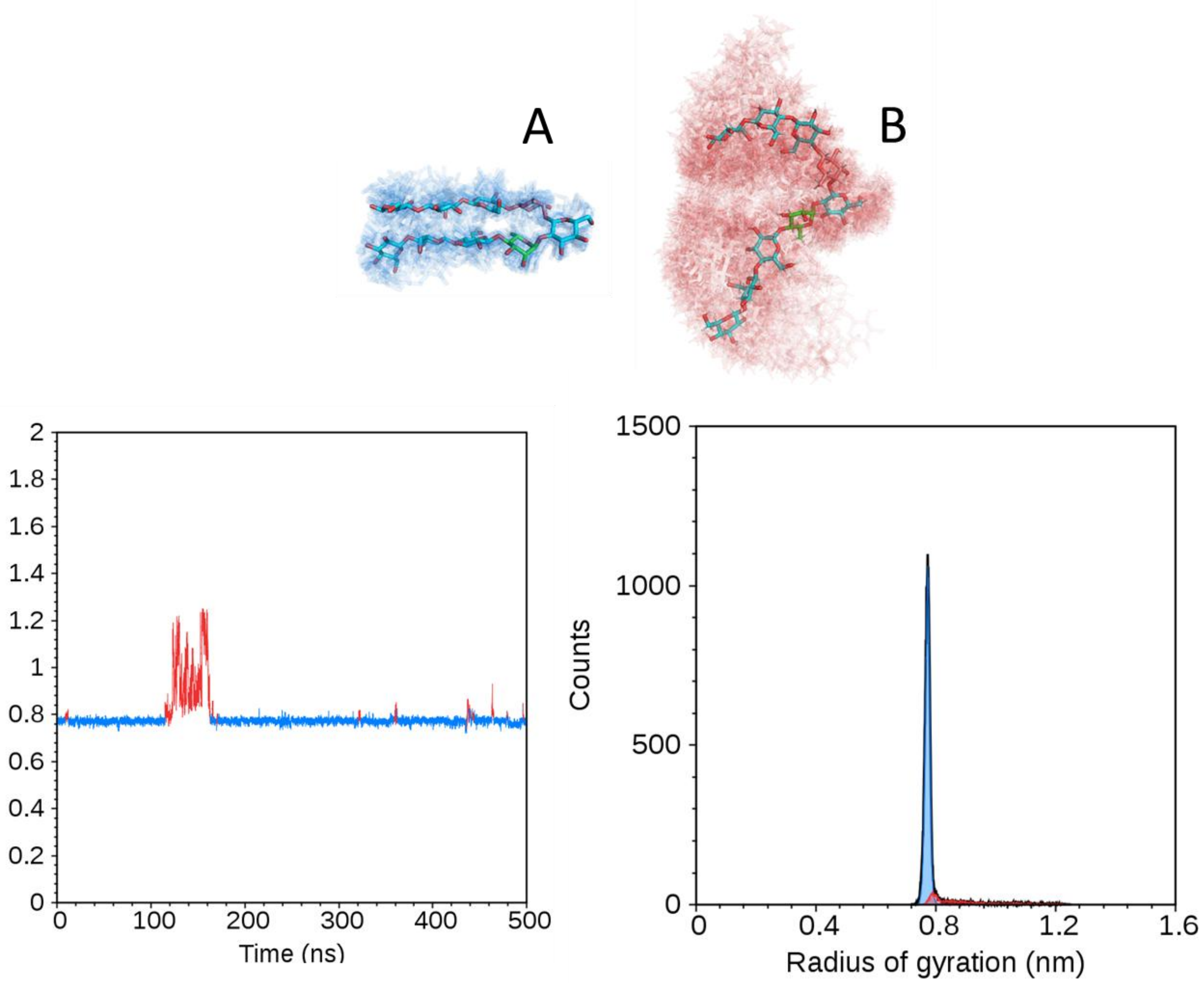

Figure S44. Conformational states mapped by radius of gyration: Distributions and alignments

Table S28. Relative abundance of molecular conformations of **a-9mer-IV** from MD simulations

	**A**	**B**
**Percentage**	87.0	13.0

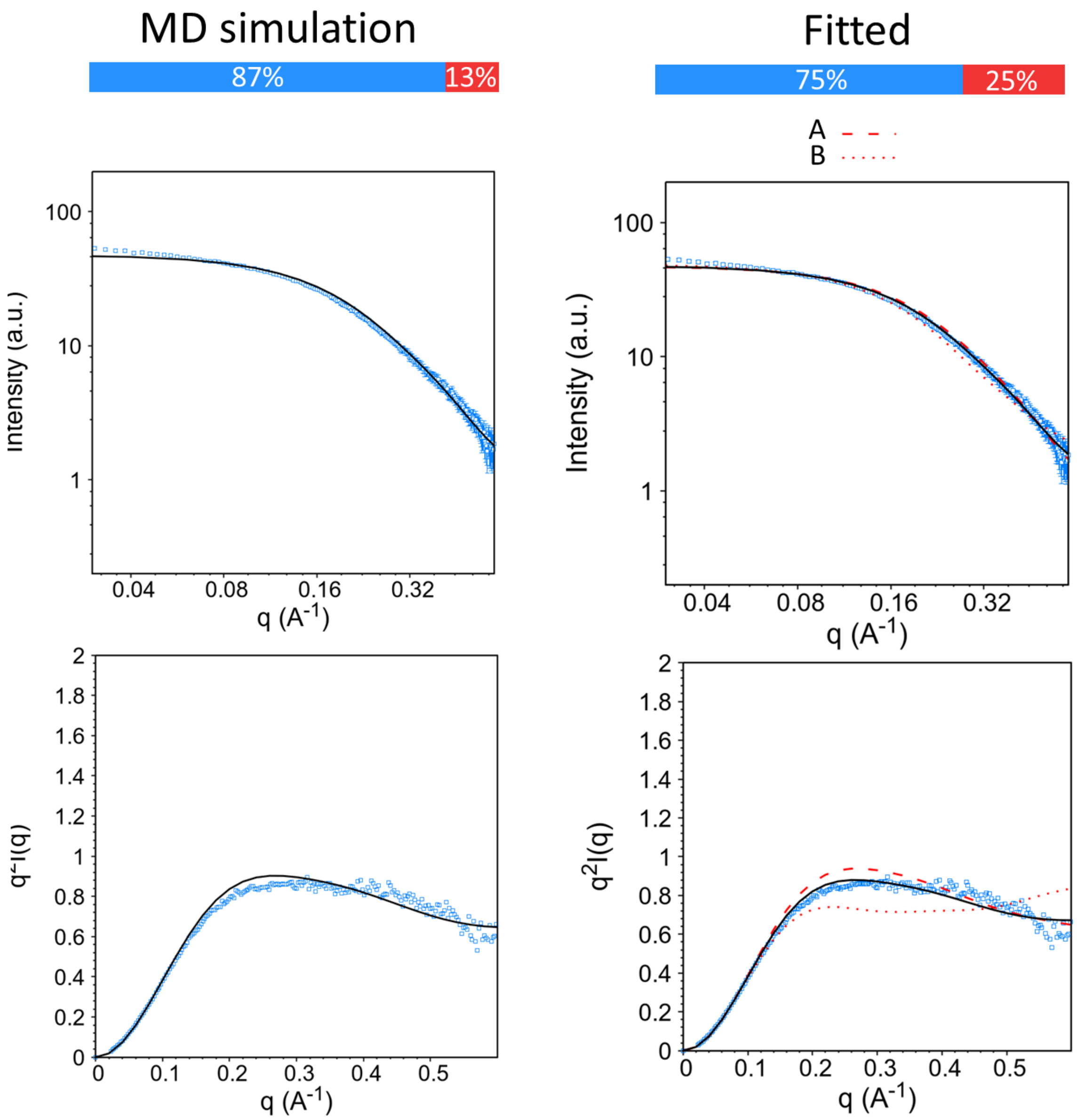

Figure S45. Comparison of experimental and predicted SAXS profiles and Kratky plots of a-**9mer-IV**: individual conformations (dashed) and combined model (solid)

7.8 Conformational analysis of ttt-15mer-IV

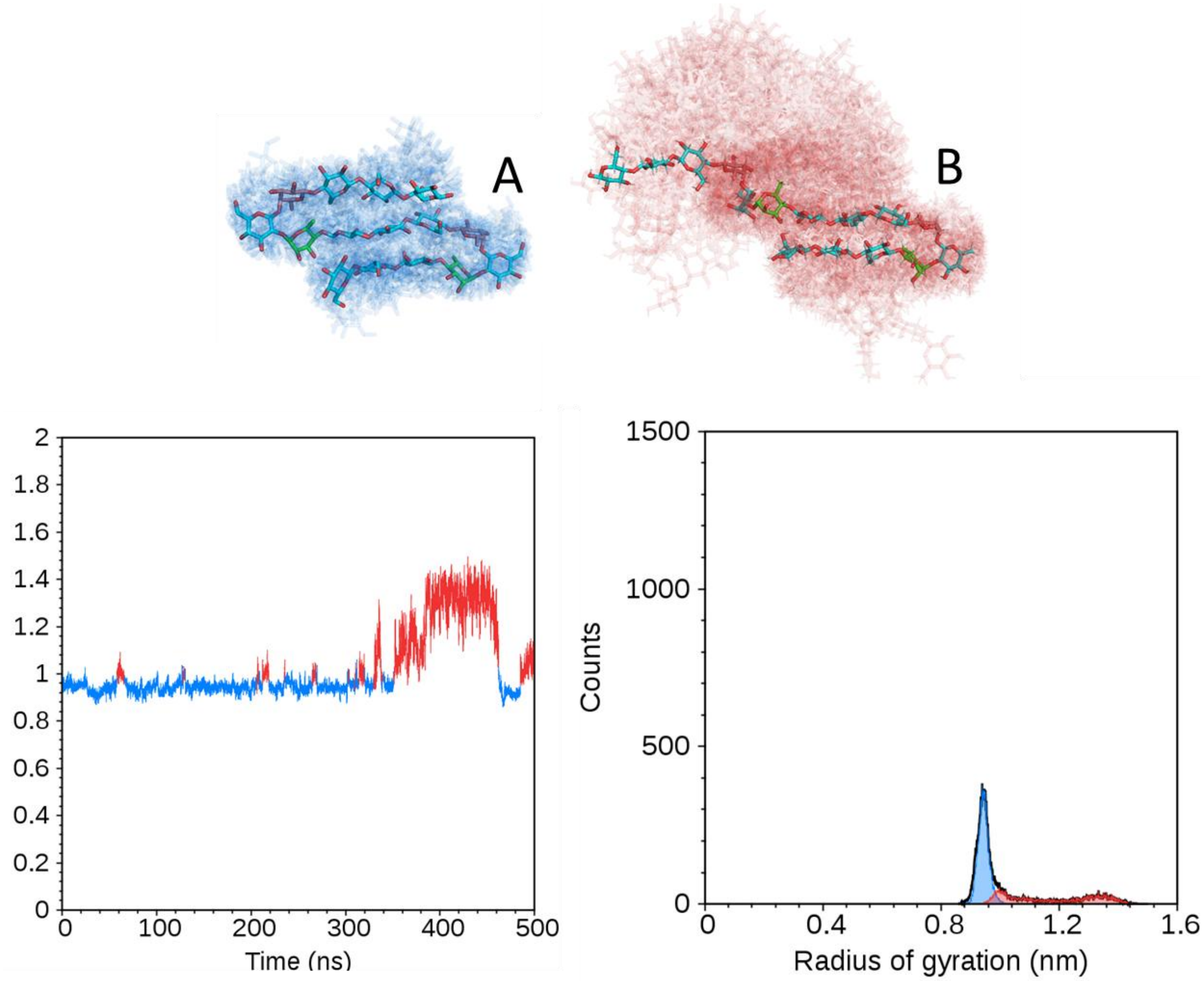

Figure S46. Conformational states mapped by radius of gyration: Distributions and alignments

Table S29. Relative abundance of molecular conformations of **a-9mer-IV** from MD simulations

	A	B
**Percentage**	67.0	33.0

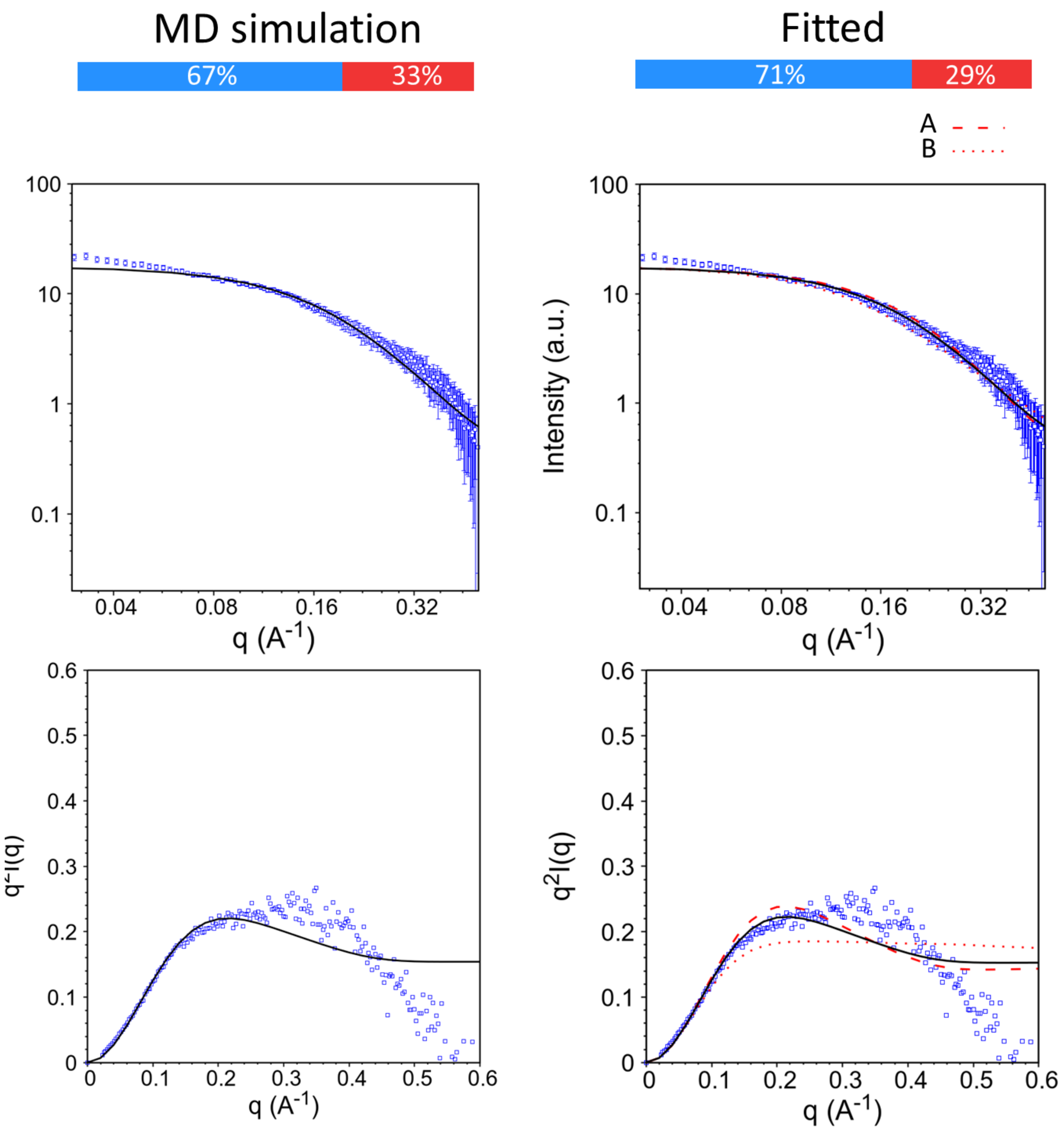

Figure S47. Comparison of experimental and predicted SAXS profiles and Kratky plots of **a-9mer-IV**: individual conformations (dashed) and combined model (solid).

8 References

1. Guberman, M. & Seeberger, P. H. Automated Glycan Assembly: A Perspective. *J. Am. Chem. Soc.* **141**, 5581–5592 (2019).
2. Dallabernardina, P., Schuhmacher, F., Seeberger, P. H. & Pfrengle, F. Mixed-Linkage Glucan Oligosaccharides Produced by Automated Glycan Assembly Serve as Tools To Determine the Substrate Specificity of Lichenase. *Chem. – Eur. J.* **23**, 3191–3196 (2017).
3. Fittolani, G. *et al.* Synthesis of a glycan hairpin. *Nat. Chem.* **15**, 1461–1469 (2023).
4. Le Mai Hoang, K. *et al.* Traceless Photolabile Linker Expedites the Chemical Synthesis of Complex Oligosaccharides by Automated Glycan Assembly. *J. Am. Chem. Soc.* **141**, 9079–9086 (2019).
5. Delbianco, M. *et al.* Well-Defined Oligo- and Polysaccharides as Ideal Probes for Structural Studies. *J. Am. Chem. Soc.* **140**, 5421–5426 (2018).
6. Smid, P., De Ruiter, G. A., Van Der Marel, G. A., Rombouts, F. M. & Van Boom, J. H. Iodonium-Ion Assisted Stereospecific Glycosylation: Synthesis of Oligosaccharides Containing α(1-4)-Linked L-Fucopyranosyl Units. *J. Carbohydr. Chem.* **10**, 833–849 (1991).
7. Kirschner, K. N. *et al.* GLYCAM06: A generalizable biomolecular force field. Carbohydrates. *J. Comput. Chem.* **29**, 622–655 (2008).
8. Sauter, J. & Grafmüller, A. Predicting the Chemical Potential and Osmotic Pressure of Polysaccharide Solutions by Molecular Simulations. *J. Chem. Theory Comput.* **12**, 4375–4384 (2016).
9. Mahoney, M. W. & Jorgensen, W. L. A five-site model for liquid water and the reproduction of the density anomaly by rigid, nonpolarizable potential functions. *J. Chem. Phys.* **112**, 8910–8922 (2000).
10. Hoover, W. G. Canonical dynamics: Equilibrium phase-space distributions. *Phys. Rev. A* **31**, 1695–1697 (1985).

11. Nosé, S. A unified formulation of the constant temperature molecular dynamics methods. *J. Chem. Phys.* **81**, 511–519 (1984).

12. Parrinello, M. & Rahman, A. Polymorphic transitions in single crystals: A new molecular dynamics method. *J. Appl. Phys.* **52**, 7182–7190.

13. Parrinello, M. & Rahman, A. Crystal Structure and Pair Potentials: A Molecular-Dynamics Study. *Phys. Rev. Lett.* **45**, 1196–1199 (1980).

14. Darden, T., York, D. & Pedersen, L. Particle mesh Ewald: An N·log(N) method for Ewald sums in large systems. *J. Chem. Phys.* **98**, 10089–10092 (1993).

15. Hess, B., Bekker, H. & Berendsen, H. J. C. 3 LINCS: a linear constraint solver for molecular simulations. *J. Comput. Chem.* **18**, 1463–1472.

16. Miyamoto, S. & Kollman, P. A. Settle: An analytical version of the SHAKE and RATTLE algorithm for rigid water models. *J. Comput. Chem.* **13**, 952–962 (1992).

17. Chen, P. & Hub, J. S. Validating Solution Ensembles from Molecular Dynamics Simulation by Wide-Angle X-ray Scattering Data. *Biophys. J.* **107**, 435–447 (2014).

18. Hub, J. S. Interpreting solution X-ray scattering data using molecular simulations. *Curr. Opin. Struct. Biol.* **49**, 18–26 (2018).

19. Knight, C. J. & Hub, J. S. WAXSiS: a web server for the calculation of SAXS/WAXS curves based on explicit-solvent molecular dynamics. *Nucleic Acids Res.* **43**, W225–W230 (2015).

20. Chatzimagas, L. & Hub, J. S. Predicting solution scattering patterns with explicit-solvent molecular simulations. *ArXiv Prepr. ArXiv220404961* (2022).

21. Doyle, P. T. & Turner, P. S. Relativistic Hartree-Fock X-ray and electron scattering factors. *Cryst. Phys. Diffr. Theor. Gen. Crystallogr.* **24**, 390–397 (1968).
